# Modular Synthesis of New Metalloid-Substituted Olefins from Diboryl(Silyl)Ethenes via Suzuki–Miyaura Reactions

**DOI:** 10.3390/ijms252212208

**Published:** 2024-11-14

**Authors:** Tomasz Sokolnicki, Kinga Stefanowska-Kątna, Agnieszka Czapik, Jędrzej Walkowiak, Adrian Franczyk

**Affiliations:** 1 Center for Advanced Technologies, Adam Mickiewicz University, Uniwersytetu Poznanskiego 10, 61-614 Poznan, Poland; 2 Faculty of Chemistry, Adam Mickiewicz University, Uniwersytetu Poznanskiego 8, 61-614 Poznan, Poland; agnieszka.czapik@amu.edu.pl

**Keywords:** (*E*)-1-silyl-1,2-diboryl-ethenes, aryl iodides, (*E*)-(2-iodovinyl)benzenes, Suzuki–Miyaura cross-coupling, substituted olefins, building blocks

## Abstract

A novel approach towards synthesizing new metalloid-substituted olefins has been accomplished by transforming (*E*)-1,2-diboryl-1-silylethenes through two consecutive Suzuki–Miyaura coupling reactions. This methodology provides an effective and selective way to obtain new, structurally different products, such as (*E*)-1-silyl-1-boryl-2-arylethens, (1*E,3E*)-1-silyl-1-boryl-2-alkenylethens, and (*E*)-1-silyl-1-aryl_1_-2-aryl_2_ethenes, which are difficult to synthesize through hydrometallation reactions and related processes. Due to the presence of reactive motifs (silyl group, Bpin moiety, and C_sp2_-H bond) in the structure of the final products, these molecules might be considered powerful building blocks in modern chemistry. With the aid of demetallation and cross-coupling reactions, they might be further functionalized into several invaluable chemicals, i.e., tetrasubstituted olefins (anti-cancer drugs, fluorescence materials), compounds with high π-conjugation, and polymers.

## 1. Introduction

Olefins are one of the most important structural motifs, which are commonly found in many organic compounds, including acyclic, cyclic, and heterocyclic systems [1,2]. Differently substituted C=C bonds are prevalent in numerous natural products [3], pharmaceuticals [4,5,6], and organic materials [7,8]. On the other hand, olefins can serve as building blocks in a variety of chemical transformations, including epoxidation, aziridination, cyclopropanation, hydrogenation, hydroelementation, transmetallation, Diels–Alder reactions, and many more, giving access to highly functionalized products [9,10]. Since the geometry of double bonds has a profound impact on the product properties (e.g., biological or optical activity), the control of regio- and stereoselectivity is critical when planning a synthetic method.

Among the many traditional methods for the construction of substituted olefins, such as the dehydrohalogenation of haloalkanes, dehydration of alcohols, carbonyl group transformations (e.g., Wittig reaction and its variations, McMurry coupling), and olefin metathesis, the application of alkenes bearing metal(loid) moieties in cross-coupling reactions has sparked a growing interest in recent years. This method, with a proper selection of reagents, catalysts, and their addition sequence, has emerged as an efficient and highly selective way to produce a wide array of peripherally expanded olefins in a regio- and stereodefined manner.

To date, several methods have been developed for the synthesis of metal(loid)-substituted starting materials, leading to precursors with different numbers of carbon-metal(loid) bonds [M = B, Si, Sn, and other]. Olefins equipped with boron and/or silicon groups are especially appealing because of their high activity in the carbon-carbon bond forming reactions, such as Suzuki–Miyaura or Hiyama–Denmark cross-coupling, respectively. These olefins are exceptionally appealing in the stereoselective preparation of diverse tri- and tetrasubstituted olefins with high application potential in both academia and industry.

In recent years, the cross-coupling-based methodology has experienced a renaissance, and many articles have been published in this field. For example, it has been reported that tri-substituted alkenes with one metalloid (M = B or Si) group can easily be obtained by catalytic carboboration [11,12,13]/carbosilylation of internal alkynes [14,15,16] or diarylation of borylalkynes [17,18,19], giving a useful structural pattern for the synthesis of tetrasubstituted olefins in combination with cross-coupling procedures.

Another approach involves the application of disubstituted alkenes with two identical metalloid groups (M_1_ = M_2_ = B or Si). Such precursors can be synthesized either by the dimetalation of alkynes [20,21,22] or carbometallation of metalloid-alkynes [23], leading to dimetalated intermediates, which can be further modified with two identical or different halides via one- or two-step cross-coupling procedures.

Fewer studies have been devoted to the synthesis of trimetalated compounds, which might be explained by the difficulties in selectively differentiating between three reactive carbon-metal bonds to enable all three cross-coupling reactions. To the best of our knowledge, only three articles on the synthesis of triborylated alkenes exist. Ozerov [24] and Marder [25,26] independently reported the preparation of alkenes bearing three Bpin moieties, while the Srebnik [27] and Nishihara [28] groups published the synthesis of triborylated alkenes, where one Bpin group located in a *trans* position to aryl moiety was replaced by a B(mida) group (mida = *N*-methyliminodiacetate). Nevertheless, it must be noted that the obtained triborylated products were susceptible to only one Suzuki–Miyaura reaction, leading to diborylated products with cross-coupled groups positioned *trans* to either the aryl or B(mida) group, respectively. An effective procedure for the synthesis of alkenes with three boryl groups of diversified reactivities has been, for the first time, achieved by the Tsuchimoto group. The authors demonstrated that products obtained by the platinum-catalyzed diboration of alkynyl−Bdan compounds with B_2_(pin)_2_ can be further cross-coupled regioselectively with three different aryl halides in a stepwise manner to yield tetrasubstituted alkenes [29]. The key aspect of this finding was the application of the Bdan group (dan = 1,8-diaminonaphthyl) as a masked boryl group, sparsely reactive under Suzuki–Miyaura reaction conditions, that was converted into a reactive Bpin group before the final cross-coupling.

Another excellent synthetic opportunity for metalloid-functionalized olefins involves alkenes bearing both boron and silicon moieties in one molecule. The combined reactivity of both metalloids might be applicable in further derivatization to produce all-carbon substituted olefins, and more sophisticated architectures. The most convenient method to access such compounds is probably the silaboration of alkynes. Numerous articles on the efficient synthesis of these trifunctional building blocks have been published, leading to products with different stereoselectivities [30,31,32,33,34,35,36,37]. However, due to the high steric hindrance around double bonds caused by carbon substituents, the modification of boryl(silyl)alkenes to tetrasubstituted olefins has remained a substantial challenge [30,31,32,33,34,35,36,37]. One of the very few examples of such modification was proposed by the Nishihara group. The authors presented highly selective, transition metal-catalyzed silaboration of alkynylboronates [38,39] and diboration of alkynylsilanes [39] as effective ways to obtain a series of *gem*- and *vic*-diborylated vinylsilanes. These trimetalated olefins were then employed as powerful precursors for the synthesis of tetra-arylated olefins by stereoselective Suzuki−Miyaura reactions. The developed reaction conditions permitted discrimination between the two boryl moieties, and allowed a pallet of products with high selectivity and good group tolerance to be obtained.

**Scheme 1:**
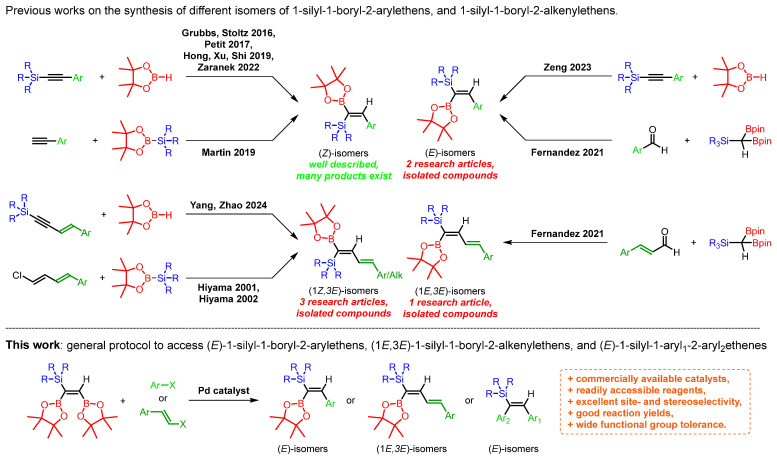
Different approaches to the synthesis of 1-boryl-1-silyl-2-substituted-ethenes [41,42,43,44,45,46,47,48,49,50].

*Vic*-diborylated vinylsilanes can also be effectively obtained by the *cis*-hydrosilylation of borylalkynes, serving as a potent scaffold for the synthesis of 1-boryl-1-silyl-2-substituted ethenes, and 1-silyl-1-aryl_1_-2-aryl_2_ethenes, both of which are difficult to achieve in hydroelementation reactions [40]. Scheme 1 presents the synthesis methods of 1-boryl-1-silylethenes described in the literature. It is worth emphasizing that, while the stereoselective synthesis of (*Z*)-1-boryl-1-silyl-2-arylethenes (mainly via hydroboration of alkynylsilanes [41,42,43,44] or silaboration of terminal alkynes [45]) is well documented, only one publication described the formation of *E* isomers through Cr-catalyzed *cis*-selective hydroboration of silylalkynes [46], while another published article furnished a mixture of *E*/*Z* isomers (Scheme 1) [47]. Also, compounds with structural motifs of (1*E*,3*E*)-1,1-borylsilylated conjugated diene have been scarcely reported. To the best of our knowledge, the synthesis of two molecules via a boron-Wittig reaction of diborylsilylmethide lithium salts LiC(Bpin)_2_(SiR_3_) with *α, β*-unsaturated aldehydes has recently been described by Fernandez [47]. However, in the reported protocol a mixture of diastereomers 1*E*,3*E*:1*Z*,3*E* = 68:32 (SiR_3_ = SiMe_3_), and 1*E*,3*E*:1*Z*,3*E* = 85:15 (SiR_3_ = SiMe_2_*t*-Bu) were obtained. Complementary work on the synthesis of (1*Z*,3*E*)-1-boryl-1-silyl-4-alkyl-buta-1,3-diene through the treatment of alkylidene-type lithium carbenoids with silylboranes was previously reported by Hiyama, but only one compound was obtained [48,49]. One compound was also recently synthesized via the cobalt-catalyzed hydroboration of 1-trimethylsilyl-1,3-enyne with pinacolborane HBpin (Scheme 1) [50].

Despite the publication of numerous reports, new synthetic methodologies that allow for more flexible structural modifications—leading to new interesting structures, such as the aforementioned 1-boryl-1-silyl-2-aryl(alkenyl)-ethenes, and silylated unsymmetrically substituted ethenes—are still in high demand. Herein, we address current needs with a new approach towards metalloid-functionalized ethenes via a sequence of two consecutive Suzuki–Miyaura coupling reactions. Due to the presence of C(sp^2^)-H, C(sp^2^)-silyl, and C(sp^2^)-boryl moieties in the molecules of the products—reactive in Suzuki–Miyaura reactions, and potentially in Heck and Hiyama cross-coupling reactions—the synthesized compounds can be regarded as attractive precursors of various valuable tri- and tetrasubstituted all-carbon olefins, such as natural products, anti-cancer drugs (tamoxifen and its derivatives), organic luminogens with many practical applications, or polymers.

## 2. Results and Discussion

Recently, our group has been interested in the synthesis of building blocks containing boryl and silyl moieties [51,52,53,54,55,56]. In our previous work, we demonstrated the facile protocol for the preparation of boryl(silyl)alkenes through directed *cis*-hydrosilylation of a structurally different, wide group of borylalkynes with various silanes [40]. Through the proper selection of catalysts, reagents, and reaction conditions, the designed methodology yields products with boron and silicon groups of *gem* or *trans* topology. Particular interest was stimulated by the product of the reaction between ethyne-1,2-diyl bis(boronic acid pinacol ester) and various Si-H donors such as triethylsilane, benzyldimethylsilane or others, providing building blocks **1a** and **1b** with two boryl groups, silyl moiety, and C(sp^2^)-H bond. Theoretically, these compounds can be further converted into tetrasubstituted olefins with the aid of cross-couplings, including Suzuki–Miyaura, Hiyama, and Heck reactions, and also in other processes, such as demetallation protocols, hydrogenation, and many electrophilic reactions.

**Scheme 2:**
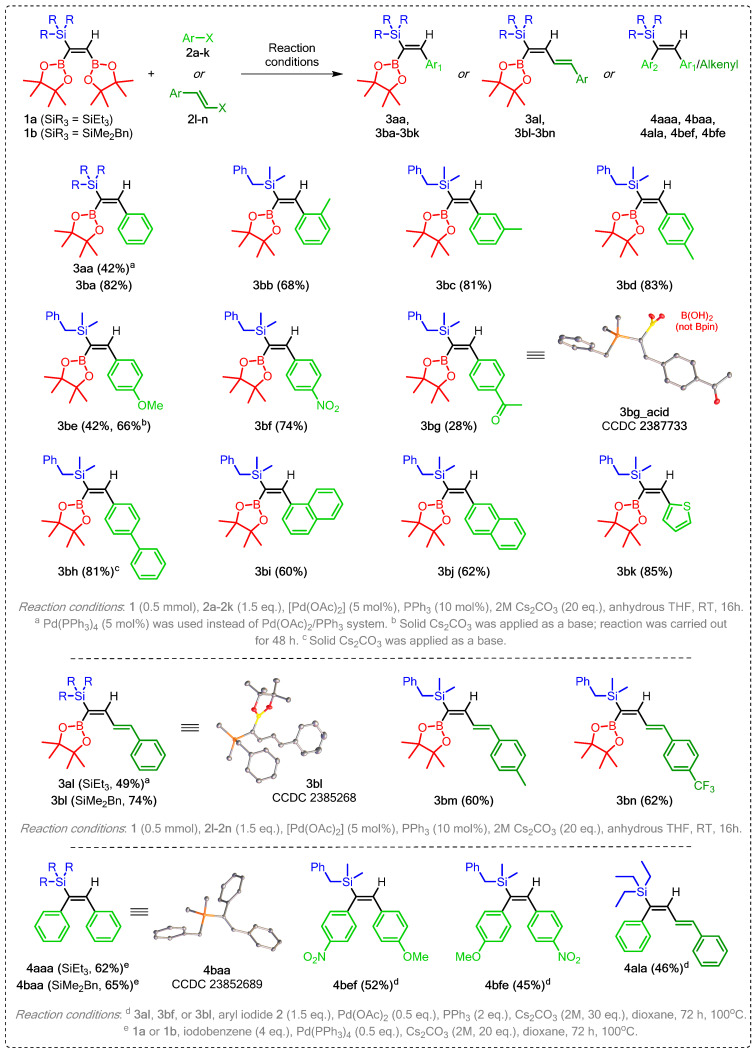
Modification of boryl(silyl)ethenes towards metalloid-substituted olefins via Suzuki–Miyaura reactions.

We commenced the studies by verifying that compounds **1a**–**b** can selectively react in two consecutive Suzuki–Miyaura reactions, discriminating between the Bpin groups which are present in the structure of the starting material (Scheme 2). We started our experimental work by evaluating the reaction of compound **1a** as a model substrate with iodobenzene in the presence of tetrakis(triphenylphosphine)palladium(0) Pd(PPh_3_)_4_ as a catalyst and 2M aqueous solution of cesium carbonate as the base. A check of the reaction carried out at room temperature after 16 h revealed the total conversion of the olefin, with the exclusive formation of product **3aa** (detailed reaction optimization is provided in the Supplementary File, Table S1). Further NMR elucidation identified this compound as a structure with a phenyl group installed *trans* in relation to the SiEt_3_ moiety, which, after extraction and column chromatography, was isolated in a 42% yield. Taking into account low yield, we switched to a mix of palladium(II) acetate and triphenylphosphine, according to the modified procedure reported by Fernandez [57]. The combination of Pd(OAc)_2_ with PPh_3_ proved to be an effective catalytic system for the control cross-coupling reaction between substrate **1b,** containing the SiMe_2_Bn unit, and iodobenzene, giving an 82% yield of product **3ba**, higher than in the case of the initially applied Pd(PPh_3_)_4_ catalyst. With robust conditions for substitution of the Bpin unit in the *trans* position to the silyl group, we next investigated the generality of this transformation by testing various aromatic iodides with different steric and electronic properties.

When 3-iodo- and 4-iodotoluenes were utilized, the corresponding products **3bc** and **3bd** were obtained in good yields (81% and 83%). However, the reaction carried out using 2-iodotoluene **2b,** due to the steric hindrance of the substrate, resulted in a slightly lower yield of 68%. Further experiments revealed a change in reaction performance, depending on whether iodides with strongly electron-donating or electron-withdrawing groups were implemented. A reaction conducted between **1b** and 1-iodo-4-nitrobenzene **2f** afforded product **3bf** in 74% yield, while a reaction of the starting diboryl(silyl)ethene with 4-iodoanisole **2e** produced product **3be** in only 42% yield. In this case, we noticed that reaction effectiveness could be improved by the application of solid cesium carbonate, instead of an aqueous solution of the base and by increasing the reaction time (48h vs. 16h), leading to a 66% yield in the final product. The reaction with 4′-iodoacetophenone **2g** proceeded less productively (28%), due to the formation of unidentified by-products (possibly created in the base-catalyzed aldol condensation).

**Figure 1:**
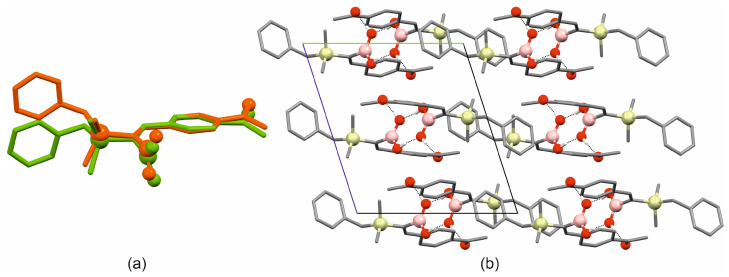
(**a**) Comparison of the molecular structure of symmetrically independent molecules of compound **3bg_acid**. (**b**) Molecular arrangement in crystal structure, view along a-axis. Dashed lines represent interactions, hydrogen atoms are omitted for clarity, and the non-C and non-H atoms are shown as balls (please see also Figure S99, and File S1).

It is worth mentioning that the crystallization of compound **3bg** resulted in the formation of crystals. However, subsequent analysis revealed that the compound had undergone a transformation, resulting in the crystals containing boronic acid **3bg_acid**. The compound crystallized with two symmetrically independent molecules in asymmetric units, each displaying slightly different conformations (see Figure 1 and Tables S2 and S3).

In the crystal structure, symmetrically dependent molecules are connected into centrosymmetric dimers via O-H···O hydrogen bonds between acidic groups. These dimers interact with each other by O-H···O hydrogen bonds, in which the acceptor is an aldehyde oxygen atom, resulting in the formation of bands. This interaction is reinforced by π···π interactions. The crystal structure reveals the phenomenon of molecular sorting, whereby symmetrically independent molecules form alternating layers.

Next, it was found that polycyclic aromatic hydrocarbons with isolated or fused rings were also good reactants under the designed conditions. The reaction of **1b** with 4-iodobiphenyl **1h** yielded product **3bh** in 81%, while experiments in the presence of 1-iodonaphtalene and 2-iodonaphtalene provided the expected isomers **3bi** and **3bj** in 60% and 62% yield, respectively. This strategy was also compatible with 2-iodothiophene, leading to the corresponding product **3bk,** with an incorporated heterocyclic unit in a very good yield of 85%. However, no product formation was observed in the reaction with 4-iodopyridine, implying that nitrogen-based heterocycles are unsuitable in this protocol. Of the 12 compounds, 10 were obtained for the first time (Scheme 2). The synthesized compounds were fully characterized by ^1^H, ^13^C, ^11^B, ^29^Si NMR, GC-MS, and elemental analysis (NMR and GC-MS data are provided in the Supplementary File, Figure S1–S98). This developed protocol is one of the first methods to provide a selective route for the exclusive formation of (*E*)-1-boryl-1-silyl-2-arylethenes with good reaction yields, and excellent functional group tolerance.

**Figure 2:**
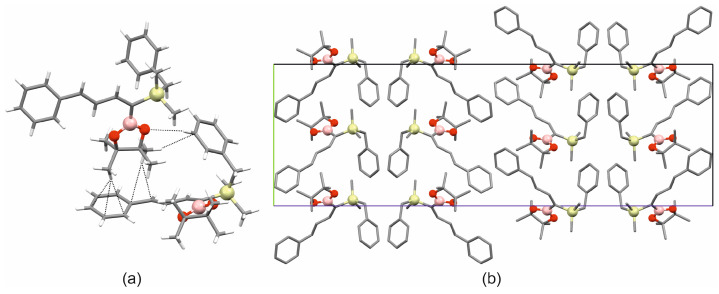
(**a**) Intermolecular interaction in the crystal structure of compound **3bl**. Dashed lines represent interactions. (**b**) Molecular arrangement viewed along a-axis (hydrogen atoms omitted for clarity). The non-C and non-H atoms are shown as balls (please see also Figure S100 and File S2).

In our further studies, we examined whether the designed catalytic system is also capable of catalyzing the reaction between model substrates **1** and alkenyl iodides. Indeed, (*E*)-(2-iodovinyl)benzene **2l** was selectively coupled with retention of configuration, providing products **3al** and **3bl** with acceptable and good yields of 49% and 74%, respectively. Additionally, the crystal structure of compound **3bl** was determined (Figure 2). The conformation of the molecule is consistent with the predictions. The phenyl substituent, attached to the silicon atom, is folded and the molecule is bent. No hydrogen bonds are observed between the molecules due to the absence of the donors and acceptors typically involved in these interactions. However, methyl groups act as donors of C-H···π type interactions, with the aromatic systems of phenyl rings acting as their acceptors (Figure 2). Additionally, *edge*-*to*-*face* type interactions between phenyl rings contribute to the stability of the crystal structure.

The successful reactions leading to products with alkenyl units bearing electron-donating methyl group **3bm**, as well as electron-withdrawing trifluoromethyl unit **3bn**, suggest that this procedure can be expanded to a selection of similar substrates with substituents of different structures and electronic properties. This results in the formation of a variety of new and interesting products featuring a (1*E*,3*E*)-1-boryl-1-silyl-4-substituted-buta-1,3-diene framework. The expected products **3bn** and **3bm,** with 1*E*,3*E* stereoselectivity, were isolated in decent yields of 60% and 62%, respectively.

Next, our research focused on the modification of the second Bpin moiety present in the structures of 1,1-borylsilylated products, obtained in the first step. For this purpose, selected compounds **3al**, **3be**, and **3bf** were reacted with the excess of aryl halides **2a**, **2f**, and **2e,** respectively (1.5 eq.). A few optimization tests showed that higher concentrations of catalytic system components—Pd(OAc)_2_ (0.5 eq.), PPh_3_ (2 eq.), Cs_2_CO_3_ (30 eq.) —along with harsher reaction conditions (higher temperature: 100 °C vs. room temperature, longer reaction time: 72 h vs. 16 h) were required to activate the Bpin unit in the *gem* position related to silyl group, ensuring the total consumption of the substrates **3**. Under the specified reaction conditions, all three 1,1-borylsilylethens underwent Suzuki–Miyaura cross-coupling reactions, leading to (1*E*,3*E*)-1-silyl-1,4-diarylbuta-1,3-diene **4ala** in 46% yield, and two (*E*)-silylated ethenes with different aryl moieties, **4bef** (52%), and **4bfe** (45%). The developed procedure, which combines two consecutive Suzuki–Miyaura reactions with two different halides, constitutes a convenient circumvention for the synthesis of silylated olefins, which are difficult to obtain via classical hydrosilylation of unsymmetrically decorated C(sp^2^)=C(sp^2^) bonds, due to chemo- and/or regioselectivity challenges.

This protocol also allows for the modification of two Bpin groups of **1** in one transformation. For this purpose, Pd(PPh_3_)_4_-catalyzed reactions were conducted between **1a** or **1b** and a 4-fold excess of iodobenzene **2a** in the presence of Cs_2_CO_3_ (20 eq.). After 72 h of reaction, carried out at 100 °C under vigorous mixing, the expected products **4aaa** and **4baa** were isolated in good yields (62% and 65%, respectively). Additionally, crystallographic analysis confirmed the molecular structure of the compound **4baa**.

**Figure 3:**
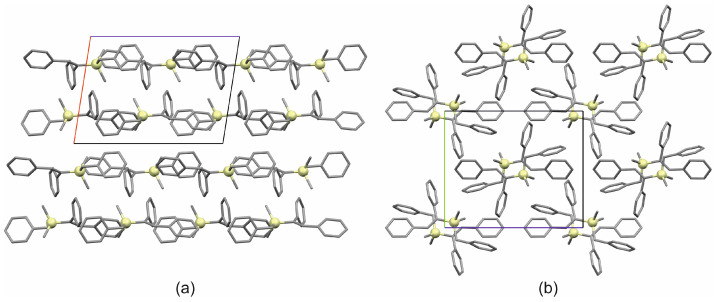
Molecular packing in the crystal structure of compound **4baa** (**a**) view along b-axis and (**b**) view along the a-axis. The hydrogen atoms are omitted for clarity, and Si-atoms are shown as balls (please see also Figure S101 and File S3).

The molecule lacks both classical donors and acceptors of hydrogen bonds. Instead, it is characterized by a high degree of aromaticity, with C-H...π interactions playing a pivotal role in stabilizing both the molecular structure and the crystal structure. The packing of molecules in the crystal is illustrated in Figure 3. This approach can be further expanded to other starting materials, offering a promising alternative for hydrosilylation reactions.

## 3. Materials and Methods

### 3.1. Materials

1,2-Bis(4,4,5,5-tetramethyl-1,3,2-dioxaborolan-2-yl)ethyne (97%, Sigma-Aldrich), triethylsilane (97%, Sigma-Aldrich, Poznan, Poland), benzyldimethylsilane (98%, Fluorochem, Hadfield, UK), Platinum(0)-1,3-divinyl-1,1,3,3-tetramethyl-disiloxane (Karstedt’s catalyst, solution in xylene, Pt 2%, Sigma-Aldrich), tetrakis(triphenylphosphine)palladium(0) (99%, Sigma-Aldrich), palladium(II) acetate (97%, Fluorochem), triphenylphosphine (99%, abcr, Karlsruhe, Germany), iodobenzene **2a** (98%, Thermo Scientific, Kandel, Germany), 2-iodotoluene **2b** (98%, Sigma-Aldrich), 3-iodotoluene **2c** (99%, Sigma-Aldrich), 4-iodotoluene **2d** (99%, Sigma-Aldrich), 4-iodoanisole **2e** (98%, AmBeed, Arlington Heights, IL, USA), 1-iodo-4-nitrobenzene **2f** (98%, Sigma-Aldrich), 4′-Iodoacetophenone **2g** (97%, Sigma-Aldrich), 4-iodobiphenyl **2h** (97%, Sigma-Aldrich), 1-iodonaphthalene **2i** (97%, Sigma-Aldrich), 2-iodonaphthalene **2j** (99%, Sigma-Aldrich), 2-iodothiophene **2k** (98%, Sigma-Aldrich), cesium carbonate (99.9%, Thermo Scientific), and chloroform-d (99.96 atom% D, Sigma-Aldrich) were used in this study.

Compounds **1a** and **1b** were synthesized following the synthetic procedures described in one of our previous works [40].

(*E*)-(2-iodovinyl)benzene **2l**, (*E*)-1-(2-iodovinyl)-4-methylbenzene **2m**, and (*E*)-1-(2-iodovinyl)-4-(trifluoromethyl)benzene **2n** were obtained according to a procedure reported in the literature [58].

Toluene and tetrahydrofuran were dried using standard procedures, deoxygenated, and stored over molecular sieves 4 Å under an argon atmosphere (toluene). Argon (99,999%) was purchased from Linde. Silica gel [MN-Kieselgel 60, 0.04–0.063 mm (230–400 mesh ASTM; Sigma-Aldrich)] was used as received.

### 3.2. Methods

^1^H, ^11^B, ^13^C, ^19^F, and ^29^Si NMR spectra were recorded at 25 °C using Bruker Ultra Shield 300 MHz and Bruker Ascend 400 MHz NANOBAY spectrometers (Bruker, Billerica, MA, USA). CDCl3 was used as a solvent, and chemical shifts are reported in ppm with reference to the residual portion solvent peak for ^1^H and ^13^C NMR, to TMS for ^29^Si NMR, and to BF_3_-Et_2_O for ^11^B NMR. The multiplicities were reported as follows: singlet (s), doublet (d), triplet (t), quartet (q), doublet of doublets (dd), and multiplet (m).

GC-MS analyses were performed on a Bruker 450-GC with a 30 m Varian DB-5 0.25 mm capillary column and a Scion SQ-MS mass spectrometry detector. Two temperature programs were used: (a) 80 °C (3 min), 10 °C/min, 250 °C (30 min), and (b) 150 °C (3 min), 10 °C/min, 280 °C (44.5 min).

Elemental analyses were carried out on a Vario EL III analyzer. The content of hydrogen and carbon was obtained as percentage data.

### 3.3. General Procedures

#### 3.3.1. General Procedure for the Synthesis of **3aa**, **3al**, and **3ba**–**3bn** via Suzuki–Miyaura Cross-Coupling of 1b with Aryl **2a**–**2k** or Alkenyl Iodides **2l**–**2n**

Pd(OAc)_2_ (5 mol%, 0,025 mmol, 5.6 mg) and PPh_3_ (10 mol%, 0,05 mmol, 13.2 mg) were introduced to a Schlenk vessel equipped with a stirring bar, and the typical evacuation/backfill cycle was performed three times. Then, anhydrous THF (5 mL), **1b** (0.5 mmol, 214.1 mg), and aryl iodide **2a**–**2k** or (*E*)-(2-iodovinyl)benzenes **2l**–**2n** (1.05 eq., 0.525 mmol) were added under an argon atmosphere, and the mixture was stirred until complete dissolution of the reagents. Finally, the solution of Cs_2_CO_3_ was injected (2M, 20 eq., 5 mL), and the biphasic system obtained was stirred vigorously at room temperature for 16 h. The conversion of reagents was determined by GC-MS analysis. The post-reaction mixture was then extracted with *n*-hexane twice, followed by drying of combined organic layers over anhydrous Na_2_SO_4_. The pure products were isolated by silica gel column chromatography, giving products **3ba**–**3bn**.

The products **3aa** and **3al** were synthesized from **1a** (0.5 mmol, 197.1 mg), and iodobenzene **2a** (1.05 eq., 0.525 mmol, 59 μL) or (*E*)-(2-iodovinyl)benzene **2l** (1.05 eq., 0.525 mmol, 121 mg), respectively, according to the procedure described above; however, Pd(PPh_3_)_4_ (5 mol%, 0.025 mmol, 28.8 mg) served as a catalyst instead of Pd(OAc)_2_ with PPh_3_.

#### 3.3.2. General Procedure for the Synthesis of **4ala**, **4bef**, and **4bfe** via Suzuki-Miyaura Cross-Coupling of **3al**, **3be**, and **3bf** with Aryl Iodides *2a, 2f, or 2a Respectively*

Pd(OAc)_2_ (0.5 eq., 0,125 mmol, 28.1 mg) and PPh_3_ (2 eq., 0.5 mmol, 131 mg) were introduced to a Schlenk vessel equipped with a stirring bar, and the evacuation/backfill cycle was performed three times. Then, anhydrous 1,4-dioxane (1.5 mL), **3al**, **3be**, or **3bf** (0.25 mmol) dissolved in 1,4-dioxane (1.5 mL), and aryl iodide **2a**, **2f**, or **2a**, respectively (1.5 eq., 0.375 mmol), were added under an argon atmosphere, and the mixture was stirred until complete dissolution of the reagents. Finally, the solution of Cs_2_CO_3_ was injected (2M, 30 eq., 3.8 mL), and the biphasic system obtained was stirred vigorously at 100 °C for 72 h. The further synthetic procedure follows the methodology described in Section 3.3.1.

#### 3.3.3. General Procedure for the Synthesis of **4aaa** and **4baa** via Suzuki–Miyaura Cross-Coupling of **1a** or **1b** with Iodobenzene **2a** (Functionalization of Both Bpin Groups in One Step)

Pd(PPh_3_)_4_ (0.25 eq., 0.0625 mmol, 72.2 mg) was introduced to the Schlenk vessel equipped with a stirring bar, and the evacuation/backfill cycle was performed three times. Then, anhydrous 1,4-dioxane (2.5 mL), **1a** (0.5 mmol, 98.5 mg) or **1b** (0.25 mmol, 107 mg) and iodobenzene **2a** (1.5 eq., 0.375 mmol) were added under an argon atmosphere, and the mixture was stirred until complete dissolution of the reagents. Finally, the solution of Cs_2_CO_3_ was injected (2 M, 20 eq., 2.5 mL), and the biphasic system obtained was stirred vigorously at 100 °C for 72 h. The further synthetic procedure follows the methodology described in Section 3.3.1.

## 4. Conclusions

In summary, trimetalated (*E*)-1-silyl-1,2-diborylethenes, easily accessible by catalytic hydrosilylation of borylalkynes, were employed as powerful building blocks for the preparation of a series of metalloid-functionalized olefins via palladium-catalyzed Suzuki–Miyaura cross-coupling reactions. The developed methodology permits the discrimination of the two boryl groups, thus offering practical, stereoselective access to three classes of products, such as (*E*)-1-silyl-1-boryl-2-arylethenes (12 synthesized compounds), (1*E*,3*E*)-1-silyl-1-boryl-2-alkenylethenes (four compounds), and (*E*)-1-silyl-1-aryl_1_/alkenyl-2-aryl_2_ethenes (three compounds: Ar_1_ ≠ Ar_2_, and two compounds: Ar_1_ = Ar_2_). It is worth pointing out that 17 of 21 products are new, and have never been reported. Various functional groups in the structure of aryl/alkenyl iodide are compatible with this protocol. The obtained products build a new class of reagents, which, in the case of the development of C-Si activation methods for such congested substrates, can potentially be further transformed into tri- and tetrasubstituted olefins, as well as halogenated derivatives.

## Data Availability

The data supporting this article have been included as part of the Supplementary Information.

## Supplementary Materials

The following supporting information can be downloaded at: https://www.mdpi.com/article/10.3390/ijms252212208/s1. References [40,46,59,60,61,62,63,64,65] are cited in the Supplementary Materials.
