# Supplementary material for: Modular Synthesis of New Metalloid-Substituted Olefins from Diboryl(Silyl)Ethenes via Suzuki–Miyaura Reactions
Source: Int J Mol Sci. 2024 Nov 14;25(22):12208. doi: 10.3390/ijms252212208 (PMC11595092; doi:10.3390/ijms252212208)

checkCIF/PLATON report

Structure factors have been supplied for datablock(s) 3bg

THIS REPORT IS FOR GUIDANCE ONLY. IF USED AS PART OF A REVIEW PROCEDURE FOR PUBLICATION, IT SHOULD NOT REPLACE THE EXPERTISE OF AN EXPERIENCED CRYSTALLOGRAPHIC REFEREE.

No syntax errors found. CIF dictionary Interpreting this report

Datablock: 3bg

```
Bond precision:   C-C = 0.0019 A          Wavelength=1.54178

Cell:           a=11.8512(4)      b=11.9282(5)      c=13.3864(4)
                alpha=71.741(2)   beta=80.284(2)    gamma=85.293(2)
Temperature:    100 K
```

	Calculated	Reported
Volume	1770.43(11)	1770.43(11)
Space group	P -1	P -1
Hall group	-P 1	-P 1
Moiety formula	C19 H23 B O3 Si	C19 H23 B O3 Si
Sum formula	C19 H23 B O3 Si	C19 H23 B O3 Si
Mr	338.27	338.27
Dx,g cm-3	1.269	1.269
Z	4	4
Mu (mm-1)	1.276	1.276
F000	720.0	720.0
F000'	722.93	
h,k,lmax	14,14,16	14,14,16
Nref	6727	6709
Tmin,Tmax	0.807,0.847	0.694,0.753
Tmin'	0.795	

```
Correction method= # Reported T Limits: Tmin=0.694 Tmax=0.753
AbsCorr = MULTI-SCAN

Data completeness= 0.997           Theta(max)= 69.979

R(reflections)= 0.0336( 6081)              wR2(reflections)=
                                           0.1036( 6709)
S = 1.070                 Npar= 443
```

The following ALERTS were generated. Each ALERT has the format
test-name_ALERT_alert-type_alert-level.
Click on the hyperlinks for more details of the test.

Alert level B

```
PLAT110_ALERT_2_B ADDSYM Detects Potential Lattice Translation ...          ? Check
PLAT112_ALERT_2_B ADDSYM Detects New (Pseudo) Symm. Elem          B       100 %Fit
PLAT113_ALERT_2_B ADDSYM Suggests Possible Pseudo/New  Space Group        P-1 Check
              Check Model Parameter Symmetry for Reflection Data Support
```

Alert level C

```
PLAT911_ALERT_3_C Missing FCF Refl Between Thmin & STh/L=    0.600         10 Report
                6 -8  1,   1 -4  1,   1 -2  1,   5 -2  1,  -5  9  1,   4-12  2,
                2 -3  2,   1 -9  3,   3  4  3,  -1 14  5,
```

Alert level G

```
PLAT007_ALERT_5_G Number of Unrefined Donor-H Atoms .............           4 Report
              H1A   H2A   H1B   H2B
PLAT116_ALERT_2_G ADDSYM Included (Pseudo) Lattice Translation ...     Please Check
PLAT154_ALERT_1_G The s.u.'s on the Cell Angles are Equal ..(Note)      0.002 Degree
PLAT432_ALERT_2_G Short Inter X...Y Contact  O1A      ..C9B      .       2.98 Ang.
                                                      x,y,z  =      1_555 Check
PLAT432_ALERT_2_G Short Inter X...Y Contact  O2B      ..C9A      .       2.92 Ang.
                                                      x,y,z  =      1_555 Check
PLAT720_ALERT_4_G Number of Unusual/Non-Standard Labels .........           2 Note
              H2AA    H2BA
PLAT912_ALERT_4_G Missing # of FCF Reflections Above STh/L=  0.600          8 Note
PLAT969_ALERT_5_G The 'Henn et al.' R-Factor-gap value ..........       5.975 Note
              Predicted wR2: Based on SigI**2  1.73 or SHELX Weight  9.68
PLAT978_ALERT_2_G Number C-C Bonds with Positive Residual Density.          7 Info
```

0 **ALERT level A** = Most likely a serious problem - resolve or explain
3 **ALERT level B** = A potentially serious problem, consider carefully
1 **ALERT level C** = Check. Ensure it is not caused by an omission or oversight
9 **ALERT level G** = General information/check it is not something unexpected

1 ALERT type 1 CIF construction/syntax error, inconsistent or missing data
7 ALERT type 2 Indicator that the structure model may be wrong or deficient
1 ALERT type 3 Indicator that the structure quality may be low
2 ALERT type 4 Improvement, methodology, query or suggestion
2 ALERT type 5 Informative message, check

It is advisable to attempt to resolve as many as possible of the alerts in all categories. Often the minor alerts point to easily fixed oversights, errors and omissions in your CIF or refinement strategy, so attention to these fine details can be worthwhile. In order to resolve some of the more serious problems it may be necessary to carry out additional measurements or structure refinements. However, the purpose of your study may justify the reported deviations and the more serious of these should normally be commented upon in the discussion or experimental section of a paper or in the "special_details" fields of the CIF. checkCIF was carefully designed to identify outliers and unusual parameters, but every test has its limitations and alerts that are not important in a particular case may appear. Conversely, the absence of alerts does not guarantee there are no aspects of the results needing attention. It is up to the individual to critically assess their own results and, if necessary, seek expert advice.

Publication of your CIF in IUCr journals

A basic structural check has been run on your CIF. These basic checks will be run on all CIFs submitted for publication in IUCr journals (*Acta Crystallographica*, *Journal of Applied Crystallography*, *Journal of Synchrotron Radiation*); however, if you intend to submit to *Acta Crystallographica Section C* or *E* or *IUCrData*, you should make sure that full publication checks are run on the final version of your CIF prior to submission.

Publication of your CIF in other journals

Please refer to the *Notes for Authors* of the relevant journal for any special instructions relating to CIF submission.

PLATON version of 22/08/2024; check.def file version of 21/08/2024

Datablock 3bg - ellipsoid plot

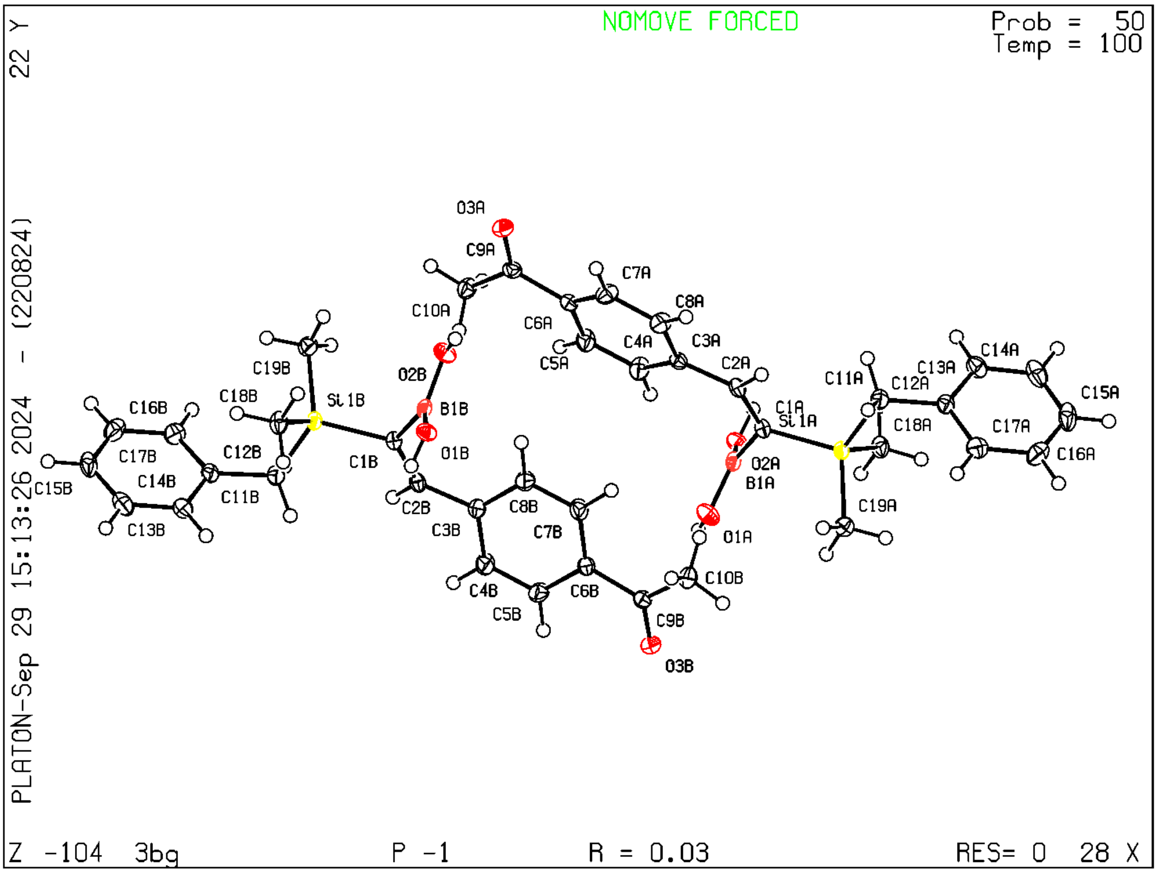

Supplement: Supplementary file 1 [file ijms-25-12208-s001.zip › File S1.pdf]
