# Supplementary material for: Modular Synthesis of New Metalloid-Substituted Olefins from Diboryl(Silyl)Ethenes via Suzuki–Miyaura Reactions
Source: Int J Mol Sci. 2024 Nov 14;25(22):12208. doi: 10.3390/ijms252212208 (PMC11595092; doi:10.3390/ijms252212208)

checkCIF/PLATON report

Structure factors have been supplied for datablock(s) 4baa

THIS REPORT IS FOR GUIDANCE ONLY. IF USED AS PART OF A REVIEW PROCEDURE FOR PUBLICATION, IT SHOULD NOT REPLACE THE EXPERTISE OF AN EXPERIENCED CRYSTALLOGRAPHIC REFEREE.

No syntax errors found. CIF dictionary Interpreting this report

Datablock: 4baa

Bond precision:	C-C = 0.0017 A		Wavelength=1.54178
Cell:	a=10.5454(2)	b=12.0637(2)	c=14.5360(3)
	alpha=90	beta=98.923(1)	gamma=90
Temperature:	100 K		

	Calculated	Reported
Volume	1826.84(6)	1826.84(6)
Space group	P 21/c	P 1 21/c 1
Hall group	-P 2ybc	-P 2ybc
Moiety formula	C23 H24 Si	C23 H24 Si
Sum formula	C23 H24 Si	C23 H24 Si
Mr	328.51	328.51
Dx,g cm-3	1.194	1.194
Z	4	4
Mu (mm-1)	1.106	1.106
F000	704.0	704.0
F000’	706.60	
h,k,lmax	13,14,17	13,14,17
Nref	3610	3602
Tmin,Tmax	0.842,0.915	0.671,0.754
Tmin’	0.710	

Correction method= # Reported T Limits: Tmin=0.671 Tmax=0.754
AbsCorr = MULTI-SCAN

Data completeness= 0.998 Theta(max)= 72.483

R(reflections)= 0.0301(3320) wR2(reflections)= 0.0799(3602)

S = 1.038 Npar= 219

```
The following ALERTS were generated. Each ALERT has the format
       test-name_ALERT_alert-type_alert-level.
Click on the hyperlinks for more details of the test.
```

Alert level G

```
PLAT912_ALERT_4_G Missing # of FCF Reflections Above STh/L=  0.600          7 Note
PLAT969_ALERT_5_G The 'Henn et al.' R-Factor-gap value ..........      4.767 Note
              Predicted wR2: Based on SigI**2  1.68 or SHELX Weight  7.70
PLAT978_ALERT_2_G Number C-C Bonds with Positive Residual Density.         21 Info
```

```
   0 ALERT level A = Most likely a serious problem - resolve or explain
   0 ALERT level B = A potentially serious problem, consider carefully
   0 ALERT level C = Check. Ensure it is not caused by an omission or oversight
   3 ALERT level G = General information/check it is not something unexpected

   0 ALERT type 1 CIF construction/syntax error, inconsistent or missing data
   1 ALERT type 2 Indicator that the structure model may be wrong or deficient
   0 ALERT type 3 Indicator that the structure quality may be low
   1 ALERT type 4 Improvement, methodology, query or suggestion
   1 ALERT type 5 Informative message, check
```

It is advisable to attempt to resolve as many as possible of the alerts in all categories. Often the minor alerts point to easily fixed oversights, errors and omissions in your CIF or refinement strategy, so attention to these fine details can be worthwhile. In order to resolve some of the more serious problems it may be necessary to carry out additional measurements or structure refinements. However, the purpose of your study may justify the reported deviations and the more serious of these should normally be commented upon in the discussion or experimental section of a paper or in the "special_details" fields of the CIF. checkCIF was carefully designed to identify outliers and unusual parameters, but every test has its limitations and alerts that are not important in a particular case may appear. Conversely, the absence of alerts does not guarantee there are no aspects of the results needing attention. It is up to the individual to critically assess their own results and, if necessary, seek expert advice.

Publication of your CIF in IUCr journals

A basic structural check has been run on your CIF. These basic checks will be run on all CIFs submitted for publication in IUCr journals (*Acta Crystallographica*, *Journal of Applied Crystallography*, *Journal of Synchrotron Radiation*); however, if you intend to submit to *Acta Crystallographica Section C* or *E* or *IUCrData*, you should make sure that full publication checks are run on the final version of your CIF prior to submission.

Publication of your CIF in other journals

Please refer to the *Notes for Authors* of the relevant journal for any special instructions relating to CIF submission.

PLATON version of 22/08/2024; check.def file version of 21/08/2024

Datablock 4baa - ellipsoid plot

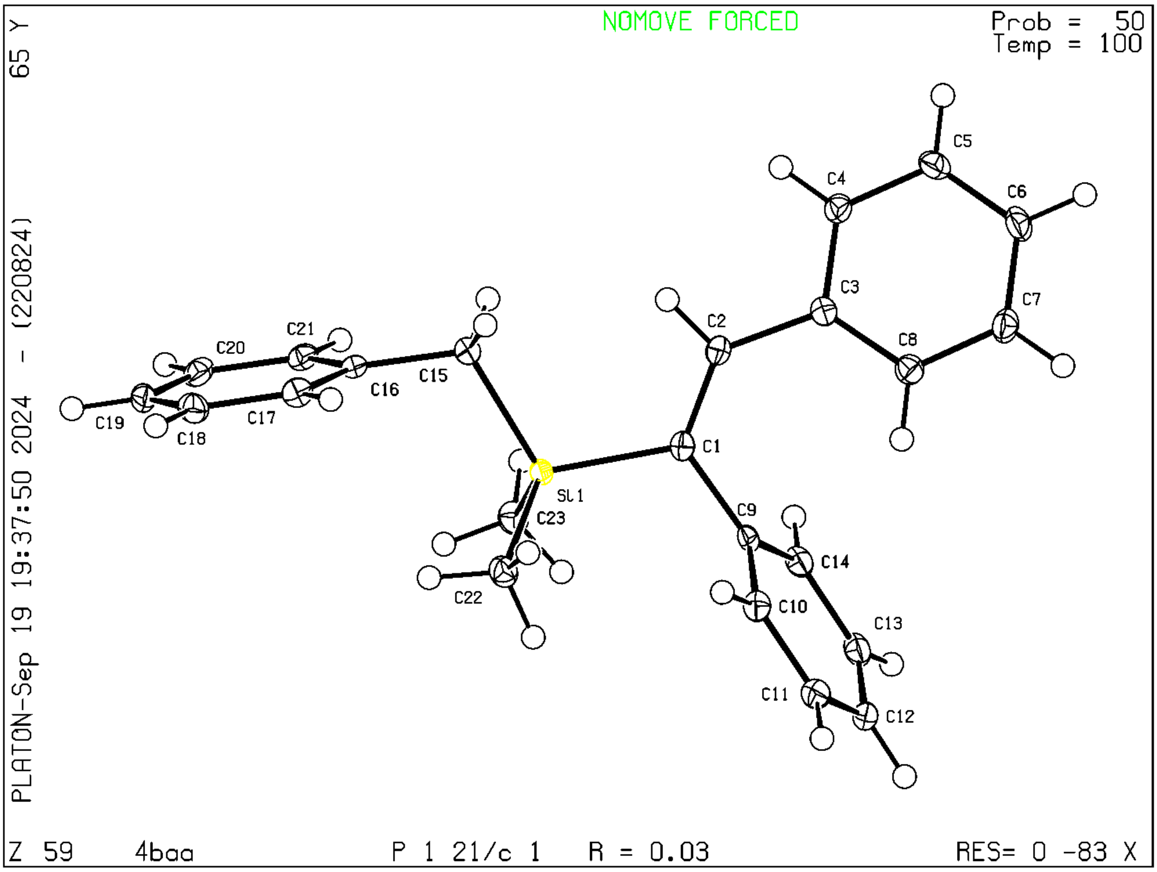

Supplement: Supplementary file 1 [file ijms-25-12208-s001.zip › File S3.pdf]
