# Supplementary material for: Modular Synthesis of New Metalloid-Substituted Olefins from Diboryl(Silyl)Ethenes via Suzuki–Miyaura Reactions
Source: Int J Mol Sci. 2024 Nov 14;25(22):12208. doi: 10.3390/ijms252212208 (PMC11595092; doi:10.3390/ijms252212208)
Supplement: Supplementary file 1 [file ijms-25-12208-s001.zip › ijms-3267152-supplementary.pdf]

Supporting Information

Modular synthesis of new metalloid-substituted olefins from diboryl(silyl)ethenes *via* Suzuki-Miyaura reactions

Tomasz Sokolnicki [1], Kinga Stefanowska-Kątna [1], Agnieszka Czapik [2], Jędrzej Walkowiak [1], and Adrian Franczyk [1,*]

[1] Center for Advanced Technologies, Adam Mickiewicz University, Uniwersytetu Poznanskiego 10, 61-614 Poznan, Poland; adrian.franczyk@amu.edu.pl

[2] Faculty of Chemistry, Adam Mickiewicz University, Uniwersytetu Poznanskiego 8, 61-614 Poznan

* adrian.franczyk@amu.edu.pl

Outline

1. Optimization of reaction conditions S2

2. NMR characterization of products S3

3. NMR spectra of products S9

4. X-ray crystallography S58

5. References S62

1. Optimization of reaction conditions

Table S1. The optimization of reaction conditions in the model experiment between (*E*)-1,2-diboryl-1-silylethenes (**1a** or **1b**) and iodobenzene (**2a**).

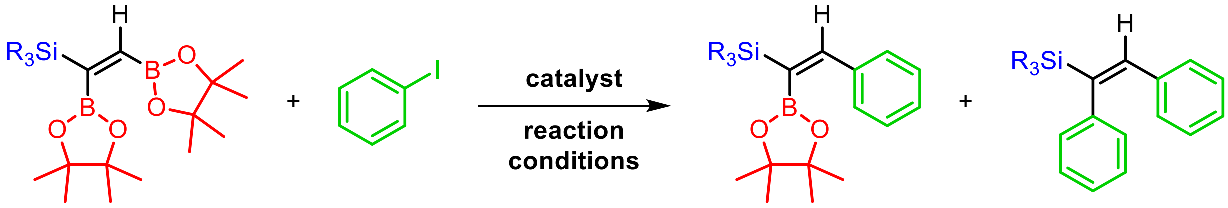

1a (SiR3 = SiEt3),
1b (SiR3 = SiMe2Bn)

2a
(1 eq.)

Lp.	Substrate	Catalyst	Reaction conditions	Selectivity 3aa:4aaa [%][a]
1.	1a	$Pd(PPh_3)_4$ (0.1 eq.)	50 °C, 24 h	64:35
2.	1a	$Pd(PPh_3)_4$ (5 mol%)	50 °C, 24 h	81:19
3.	1a	$Pd(PPh_3)_4$ (2.5 mol%)	50 °C, 24 h	86:14
4.	1a	$Pd(PPh_3)_4$ (2.5 mol%)	40 °C, 24 h	85:15
**5.**	**1a**	**$Pd(PPh_3)_4$ (2.5 mol%)**	**Room temperature, 24 h**	**95:5**
**6.**	**1b**	**$Pd(OAc)_2$ (5 mol%), PPh_3 (10 mol%)**	**Room temperature, 24 h**	**96:4[b]**
7.	1b	$Pd(OAc)_2$ (5 mol%), XPhos (10 mol%)	Room temperature, 24 h	90:10
8.	1b	$Pd(OAc)_2$ (5 mol%), SPhos (10 mol%)	Room temperature, 24 h	94:6
9.[c]	1a	$Pd(PPh_3)_4$ (0.2 eq.)	80 °C, 96 h	17:83
10.[c]	1a	$Pd(PPh_3)_4$ (0.1 eq.)	100 °C, 48 h	32:68 (2:98) [d]
**11.[c]**	**1a**	**$Pd(PPh_3)_4$ (0.25 eq.)**	**100 °C, 72 h**	**3:97**
12.[c]	1a	$Pd(PPh_3)_4$ (0.3 eq.)	70 °C, 72 h	37:63
13.[c]	1b	$Pd(OAc)_2$ (0.25 eq.), PPh_3 (0.5 eq.)	100 °C, 44 h	27:73
14.[c]	1b	$Pd(OAc)_2$ (0.5 eq.), PPh_3 (1 eq.)	100 °C, 72 h	23:77
15.[c]	1b	$Pd(OAc)_2$ (0.25 eq.), PPh_3 (1 eq.)	100 °C, 96 h	11:89
**16.[c]**	**1b**	**$Pd(OAc)_2$ (0.5 eq.), PPh_3 (2 eq.)**	**100 °C, 96 h**	**1:99**

All reactions were carried out in Schlenk vessels in anhydrous THF (0.1 M) under inert atmosphere using Cs_2CO_3 (20 eq.) as a base.

[a] Determined by GC and GC-MS analyses.

[b] The same yield was achieved, when the reaction was performed for 16 hours.

[c] 4 eq. of iodobenzene were used.

[d] After 48 h, the next portion of catalyst (0.15 eq.) was added, and the reaction was stirred another 24 h.

2. NMR characterization of products

(*E*)-Triethyl(2-phenyl-1-(4,4,5,5-tetramethyl-1,3,2-dioxaborolan-2-yl)vinyl)silane (3aa)

^{1}H NMR ($CDCl_3$, 300 MHz, δ, ppm): 7.38 – 7.29 (m, 2H), 7.24 – 7.12 (m, 4H), 1.20 (s, 12H), 0.92 (t, *J* = 7.9 Hz, 9H), 0.69 – 0.58 (q, *J* = 7.9 Hz, 6H).

^{13}C NMR ($CDCl_3$, 101 MHz, δ, ppm): 151.3, 140.8, 128.1, 128.0, 127.8, 83.5, 25.3, 7.6, 3.8. Signal from carbon atom SiBC= is not observed.

^{29}Si NMR ($CDCl_3$, 79 MHz, δ, ppm): 4.00.

MS (EI) [m/z (%)]: 316(20), 314(17), 235(21), 234(100), 232(31), 232(40), 231(20), 131(32), 130(31), 83(61), 55(21).

Isolated by column chromatography using hexane:ethyl acetate = 99:1 (yield 42%, yellow oil).

The NMR spectra are in agreement with the literature [40, 46].

(*E*)-benzyldimethyl(2-phenyl-1-(4,4,5,5-tetramethyl-1,3,2-dioxaborolan-2-yl)vinyl)silane (3ba)

^{1}H NMR ($CDCl_3$, 300 MHz, δ, ppm): 7.47 – 7.11 (m, 11H, overlapped singlet and multiplet), 2.36 (s, 2H), 1.36 (s, 12H), 0.21 (s, 6H).

^{13}C NMR ($CDCl_3$, 75 MHz, δ, ppm): 151.5, 140.4, 140.2, 128.5, 128.2, 128.1, 128.0, 127.9, 124.0, 83.6, 26.2, 25.2, -3.1. Signal from carbon atom SiBC= is not observed.

^{11}B NMR ($CDCl_3$, 96 MHz, δ, ppm): 32.45. **^{29}Si NMR** ($CDCl_3$, 79 MHz, δ, ppm): -1.74.

MS (EI) [m/z (%)]: 363.1 (M^+, -15.3, 1.5), 288.2 (17.3), 287.0 (81.6), 285.9 (18.7), 205.0 (77.7), 203.8 (19.1), 188.9 (45.8), 186.9 (39.1), 158.9 (26.5), 144.9 (36.9), 134.9 (42.8), 120.9 (35.4), 84.1 (18.3), 83.0 (100.0), 68.9 (15.3), 55.0 (51.7).

Elem. Anal. calcd for $C_{23}H_{31}BO_2Si$: C, 73.01; H, 8.26; found C, 73.35; H, 8.29.

Isolated by column chromatography using hexane:ethyl acetate = 99:1 (yield 82%, yellow oil).

The NMR spectra are in agreement with the literature [40].

(*E*)-Benzyldimethyl(1-(4,4,5,5-tetramethyl-1,3,2-dioxaborolan-2-yl)-2-(*o*-tolyl)vinyl)silane (3bb)

^{1}H NMR ($CDCl_3$, 300 MHz, δ, ppm): 7.41 – 7.31 (m, 2H), 7.24 – 7.16 (m, 3H), 7.15 – 7.05 (m, 6H), 2.29 (s, 2H), 2.26 (s, 3H), 1.23 (s, 12H), 0.16 (s, 6H).

^{13}C NMR ($CDCl_3$, 101 MHz, δ, ppm): 150.8, 140.3, 140.1, 135.8, 129.8, 128.6, 128.2, 127.9, 127.9, 125.5, 124.0, 83.5, 26.4, 25.1, 19.8, -3.0. Signal from carbon atom SiBC= is not observed.

^{11}B NMR ($CDCl_3$, 128 MHz, δ, ppm): 32.82. **^{29}Si NMR** ($CDCl_3$, 80 MHz, δ, ppm): -2.14.

MS (EI) [m/z (%)]: 302(44), 300(50), 219(100), 218(96), 176(57), 174(48), 83(65), 55(25).

Elem. Anal. calcd for $C_{24}H_{33}BO_2Si$: C, 73.46; H, 8.48; found C, 73.49; H, 8.48.

Isolated by column chromatography using hexane:ethyl acetate = 99:1 (yield 68%, yellow oil.

The compound **3bb** has been synthesized for the first time.

(*E*)-Benzyldimethyl(1-(4,4,5,5-tetramethyl-1,3,2-dioxaborolan-2-yl)-2-(*m*-tolyl)vinyl)silane (3bc)

^{1}H NMR ($CDCl_3$, 400 MHz, δ, ppm): 7.26 – 7.16 (m, 6H), 7.11 – 7.04 (m, 4H), 2.35 (s, 3H), 2.30 (s, 2H), 1.32 (s, 12H), 0.15 (s, 6H).

^{13}C NMR ($CDCl_3$, 101 MHz, δ, ppm): 151.7, 140.5, 140.3, 137.6, 128.8, 128.6, 128.4, 128.2, 128.1, 125.3, 124.0, 83.6, 26.3, 25.3, 21.5, -3.0. Signal from carbon atom SiBC= is not observed.

^{11}B NMR ($CDCl_3$, 128 MHz, δ, ppm): 32.27. **^{29}Si NMR** ($CDCl_3$, 80 MHz, δ, ppm): -1.83.

MS (EI) [m/z (%)]: 301(30), 220(100), 219(28), 218(68), 217(23), 176(27), 175(30), 174(22), 160(37), 158(27), 83(60), 56(31).

Elem. Anal. calcd for $C_{22}H_{33}BO_2Si$: C, 73.46; H, 8.48; found C, 73.43; H, 8.48.

Isolated by column chromatography using hexane:ethyl acetate = 99:1 (yield 81%, yellow oil).

The compound **3bc** has been synthesized for the first time.

(*E*)-Benzyldimethyl(1-(4,4,5,5-tetramethyl-1,3,2-dioxaborolan-2-yl)-2-(*p*-tolyl)vinyl)silane (3bd)
^{1}**H NMR** ($CDCl_3$, 300 MHz, δ, ppm): 7.31 (d, *J* = 8.1 Hz, 2H), 7.24 – 7.16 (m, 4H), 7.13 – 7.04 (m, 7H), 2.34 (s, 3H), 2.29 (s, 2H), 1.33 (s, 12H), 0.15 (s, 6H).
^{13}**C NMR** ($CDCl_3$, 101 MHz, δ, ppm): 151.5, 140.3, 137.9, 137.7, 128.8, 128.6, 128.2, 128.0, 124.0, 83.6, 26.3, 25.3, 21.4, -3.0. Signal from carbon atom SiBC= is not observed.
^{11}**B NMR** ($CDCl_3$, 128 MHz, δ, ppm): 32.56. ^{29}**Si NMR** ($CDCl_3$, 80 MHz, δ, ppm): -1.79.
MS (EI) [m/z (%)]: 301(15), 283(11), 219(25), 218(9), 202(29), 201(100), 91(8), 83(9), 55(14), 43(6).
Elem. Anal. calcd for $C_{24}H_{33}BO_2Si$: C, 73.46; H, 8.48; found C, 73.43; H, 8.47.
Isolated by column chromatography using hexane:ethyl acetate = 99:1 (yield 83%, yellow oil).
The compound **3bd** has been synthesized for the first time.

(*E*)-Benzyl(2-(4-methoxyphenyl)-1-(4,4,5,5-tetramethyl-1,3,2-dioxaborolan-2-yl)vinyl)dimethylsilane (3be)
^{1}**H NMR** ($CDCl_3$, 300 MHz, δ, ppm): 7.40 – 7.33 (m, 2H), 7.24 – 7.12 (m, 3H), 7.10 – 7.03 (m, 3H), 6.86 – 6.80 (m, 2H), 3.81 (s, 3H), 2.29 (s, 2H), 1.33 (s, 12H), 0.14 (s, 6H).
^{13}**C NMR** ($CDCl_3$, 101 MHz, δ, ppm): 159.7, 151.2, 140.4, 133.3, 129.5, 128.6, 128.2, 124.0, 113.5, 83.6, 55.4, 26.4, 25.3, -3.0. Signal from carbon atom SiBC= is not observed.
^{11}**B NMR** ($CDCl_3$, 128 MHz, δ, ppm): 31.88. ^{29}**Si NMR** ($CDCl_3$, 80 MHz, δ, ppm): -1.78.
MS (EI) [m/z (%)]: 318(57), 316(57), 236(100), 234(60), 194(23), 193(30), 191(55), 176(49), 174(42), 166(26), 164(22), 92(31), 90(20), 84(29), 83(48), 55(38).
Elem. Anal. calcd for $C_{24}H_{33}BO_3Si$: C, 70.58; H, 8.14; found C, 70.59; H, 8.14.
Isolated by column chromatography using gradient elution with hexane → hexane:ethyl acetate = 97:3 (yield 66%, yellow oil).
The compound **3be** has been synthesized for the first time.

(*E*)-Benzyldimethyl(2-(4-nitrophenyl)-1-(4,4,5,5-tetramethyl-1,3,2-dioxaborolan-2-yl)vinyl)silane (3bf)
^{1}**H NMR** ($CDCl_3$, 300 MHz, δ, ppm): 8.19 – 8.13 (m, 2H), 7.55 – 7.48 (m, 2H), 7.24 – 7.14 (m, 3H), 7.10 – 7.02 (m, 3H), 2.29 (s, 2H), 1.30 (s, 12H), 0.17 (s, 6H).
^{13}**C NMR** ($CDCl_3$, 101 MHz, δ, ppm): 148.7, 147.2, 146.7, 139.7, 128.6, 128.6, 128.3, 124.3, 123.6, 84.1, 26.0, 25.2, -3.2. Signal from carbon atom SiBC= is not observed.
^{11}**B NMR** ($CDCl_3$, 128 MHz, δ, ppm): 31.39. ^{29}**Si NMR** ($CDCl_3$, 80 MHz, δ, ppm): -1.02.
MS (EI) [m/z (%)]: 331(16), 250(11), 249(9), 84(19), 83(100), 55(24).
Elem. Anal. calcd for $C_{23}H_{30}BNO_4Si$: C, 65.25; H, 7.14; found C, 65.26; H, 7.14.
Isolated by column chromatography using hexane:ethyl acetate = 99:1 (yield 74%, yellow oil).
The compound **3bf** has been synthesized for the first time.

(*E*)-1-(4-(2-(Benzyldimethylsilyl)-2-(4,4,5,5-tetramethyl-1,3,2-dioxaborolan-2-yl)vinyl)phenyl)ethan-1-one (3bg)
^{1}**H NMR** ($CDCl_3$, 300 MHz, δ, ppm): 7.92 – 7.87 (m, 2H), 7.50 – 7.45 (m, 2H), 7.24 – 7.16 (m, 3H), 7.09 – 7.02 (m, 3H), 2.60 (s, 3H), 2.29 (s, 2H), 1.31 (s, 12H), 0.16 (s, 6H).
^{13}**C NMR** ($CDCl_3$, 101 MHz, δ, ppm): 197.9, 150.0, 144.9, 139.9, 136.3, 128.6, 128.4, 128.2, 128.1, 124.1, 83.9, 26.8, 26.1, 25.3, -3.1. Signal from carbon atom SiBC= is not observed.
^{11}**B NMR** ($CDCl_3$, 128 MHz, δ, ppm): 32.74. ^{29}**Si NMR** ($CDCl_3$, 80 MHz, δ, ppm): -1.34.
MS (EI) [m/z (%)]: 329(20), 328(47), 248(84), 246(100), 188(21), 91(22), 84(22), 83(92), 56(53), 55(40).
Elem. Anal. calcd for $C_{25}H_{33}BO_3Si$: C, 71.42; H, 7.91; found C, 71.43; H, 7.91.
Isolated by column chromatography using hexane:ethyl acetate = 99:1 (yield 28%, yellow oil.
The compound **3bg** has been synthesized for the first time.

(*E*)-(2-([1,1'-Biphenyl]-4-yl)-1-(4,4,5,5-tetramethyl-1,3,2-dioxaborolan-2-yl)vinyl)(benzyl)dimethylsilane (3bh)

^{1}H NMR ($CDCl_3$, 300 MHz, δ, ppm): 7.50 – 7.46 (m, 2H), 7.44 – 7.40 (m, 2H), 7.38 – 7.29 (m, 4H), 7.25 – 7.19 (m, 1H), 7.13 – 7.06 (m, 3H), 6.97 – 6.92 (m, 3H), 2.18 (s, 2H), 1.21 (s, 12H), 0.04 (s, 6H).

^{13}C NMR ($CDCl_3$, 101 MHz, δ, ppm): 151.0, 141.0, 140.8, 140.2, 139.4, 128.9, 128.6, 128.5, 128.2, 127.5, 127.2, 126.9, 124.1, 83.7, 26.3, 25.3, -3.0. Signal from carbon atom SiBC= is not observed.

^{11}B NMR ($CDCl_3$, 128 MHz, δ, ppm): 31.76. **^{29}Si NMR** ($CDCl_3$, 80 MHz, δ, ppm): -1.64.

MS (EI) [m/z (%)]: 364(54), 362(51), 283(34), 281(100), 281(37), 238(47), 237(37), 222(30), 220(25), 127(22), 91(30), 84(83), 83(24), 56(63), 55(50).

Elem. Anal. calcd for $C_{29}H_{35}BO_2Si$: C, 76.64; H, 7.76; found C, 76.62; H, 7.76.

Isolated by column chromatography using hexane:ethyl acetate = 99:1 (yield 81%, yellow oil).

The compound **3bh** has been synthesized for the first time.

(*E*)-Benzyldimethyl(2-(naphthalen-1-yl)-1-(4,4,5,5-tetramethyl-1,3,2-dioxaborolan-2-yl)vinyl)silane (3bi)

^{1}H NMR ($CDCl_3$, 300 MHz, δ, ppm): 8.04 – 7.87 (m, 4H), 7.69 – 7.63 (m, 1H), 7.63 – 7.57 (m, 2H), 7.56 – 7.51 (m, 1H), 7.39 – 7.33 (m, 2H), 7.25 – 7.18 (m, 2H), 2.49 (s, 2H), 1.27 (s, 12H), 0.37 (s, 6H).

^{13}C NMR ($CDCl_3$, 101 MHz, δ, ppm): 150.1, 140.3, 138.7, 133.4, 131.4, 128.7, 128.3, 128.3, 126.0, 125.8, 125.6, 125.2, 125.0, 124.1, 83.5, 27.1, 26.4, 25.0, -2.9. Signal from carbon atom SiBC= is not observed.

^{11}B NMR ($CDCl_3$, 128 MHz, δ, ppm): 30.95. **^{29}Si NMR** ($CDCl_3$, 80 MHz, δ, ppm): -2.22.

MS (EI) [m/z (%)]: 338(54), 337(45), 255(74), 254(100), 252(42), 237(84), 222(45), 212(49), 211(79), 196(52), 154(42), 153(45), 84(45), 83(89), 56(48).

Elem. Anal. calcd for $C_{27}H_{33}BO_2Si$: C, 75.69; H, 7.76; found C, 75.71; H, 7.76.

Isolated by column chromatography using hexane:ethyl acetate = 99:1 (yield 60%, yellow oil).

The compound **3bi** has been synthesized for the first time.

(*E*)-Benzyldimethyl(2-(naphthalen-2-yl)-1-(4,4,5,5-tetramethyl-1,3,2-dioxaborolan-2-yl)vinyl)silane (3bj)

^{1}H NMR ($CDCl_3$, 300 MHz, δ, ppm): 7.59 – 7.46 (m, 5H), 7.26 (dd, *J* = 8.4, 1.7 Hz, 1H), 7.18 – 7.14 (m, 2H), 7.06 (s, 1H), 6.97 – 6.89 (m, 3H), 6.82 – 6.75 (m, 4H), 2.04 (s, 2H), 1.03 (s, 12H), -0.10 (s, 6H).

^{13}C NMR ($CDCl_3$, 101 MHz, δ, ppm): 151.5, 140.2, 138.2, 133.4, 133.2, 128.6, 128.2, 128.2, 127.8, 127.7, 127.1, 126.2, 126.1, 126.1, 124.1, 83.8, 27.1, 26.3, 25.3, -3.0. Signal from carbon atom SiBC= is not observed.

^{11}B NMR ($CDCl_3$, 128 MHz, δ, ppm): 32.80. **^{29}Si NMR** ($CDCl_3$, 80 MHz, δ, ppm): -1.66.

MS (EI) [m/z (%)]: 338(32), 337(20), 336(41), 256(100), 255(48), 254(55), 238(17), 212(30), 211(24), 196(25), 83(40), 83(29), 55(26).

Elem. Anal. calcd for $C_{27}H_{33}BO_2Si$: C, 75.69; H, 7.76; found C, 75.70; H, 7.76.

Isolated by column chromatography using hexane:ethyl acetate = 99:1 (yield 62%, yellow oil).

The compound **3bj** has been synthesized for the first time.

(*E*)-Benzyldimethyl(1-(4,4,5,5-tetramethyl-1,3,2-dioxaborolan-2-yl)-2-(thiophen-2-yl)vinyl)silane (3bk)

^{1}H NMR ($CDCl_3$, 300 MHz, δ, ppm): 7.26 – 7.18 (m, 4H), 7.15 – 7.01 (m, 5H), 6.97 (dd, *J* = 5.0, 3.6 Hz, 1H), 2.29 (s, 2H), 1.39 (s, 12H), 0.14 (s, 6H).

^{13}C NMR ($CDCl_3$, 101 MHz, δ, ppm): 145.0, 142.3, 140.2, 128.5, 128.4, 128.2, 127.2, 126.4, 124.0, 83.8, 26.2, 25.5, -3.0. Signal from carbon atom SiBC= is not observed.

^{11}B NMR ($CDCl_3$, 128 MHz, δ, ppm): 31.86. **^{29}Si NMR** ($CDCl_3$, 80 MHz, δ, ppm): -0.95.

MS (EI) [m/z (%)]: 294(46), 293(56), 213(24), 211(91), 211(37), 151(38), 141(33), 127(49), 126(43), 91(30), 84(23), 83(100), 55(32), 55(25).

Elem. Anal. calcd for $C_{21}H_{29}BO_2SSi$: C, 65.61; H, 7.60; found C, 65.63; H, 7.60.

Isolated by column chromatography using hexane:ethyl acetate = 99:1 (yield 85%, yellow oil).

The compound **3bk** has been synthesized for the first time.

Triethyl((1*E*,3*E*)-4-phenyl-1-(4,4,5,5-tetramethyl-1,3,2-dioxaborolan-2-yl)buta-1,3-dien-1-yl)silane (3al)

^{1}H NMR ($CDCl_3$, 400 MHz, δ, ppm): 7.49 – 7.24 (m, 7H), 7.15 (d, *J* = 10.6 Hz, 1H), 6.69 (d, *J* = 15.5 Hz, 1H), 1.39 (s, 12H), 1.01 (t, *J* = 7.9 Hz, 9H), 0.74 (q, *J* = 7.8 Hz, 6H).

^{13}C NMR ($CDCl_3$, 101 MHz, δ, ppm): 154.7, 137.6, 135.8, 130.9, 128.7, 127.9, 126.9, 83.2, 25.2, 7.7, 3.7. Signal from carbon atom SiBC= is not observed.

^{11}B NMR ($CDCl_3$, 128 MHz, δ, ppm): δ 31.66. **^{29}Si NMR** ($CDCl_3$, 79 MHz, δ, ppm): 3.71.

MS (EI) [m/z (%)]: 342(26), 287(13), 260(100), 202(21), 186(16), 158(27), 132(19), 83(74), 70(17), 55(68).

Elem. Anal. calcd for $C_{22}H_{35}BO_2Si$: C, 71.34; H, 9.52; found C, 71.36; H, 9.52.

Isolated by column chromatography using hexane:ethyl acetate = 99:1 (yield 49%, yellow oil).

The compound **3al** has been synthesized for the first time.

Benzyldimethyl((1*E*,3*E*)-4-phenyl-1-(4,4,5,5-tetramethyl-1,3,2-dioxaborolan-2-yl)buta-1,3-dien-1-yl)silane (3bl)

^{1}H NMR ($CDCl_3$, 300 MHz, δ, ppm): 7.36 – 7.25 (m, 3H), 7.22 – 7.16 (m, 2H), 7.13 – 7.02 (m, 4H), 6.94 – 6.85 (m, 4H), 6.48 (d, *J* = 15.5 Hz, 1H), 2.12 (s, 2H), 1.21 (s, 12H), -0.05 (s, 6H).

^{13}C NMR ($CDCl_3$, 75 MHz, δ, ppm): 155.2, 140.7, 137.4, 136.6, 130.7, 128.8, 128.5, 128.1, 128.1, 127.0, 123.9, 83.3, 26.3, 25.2, -3.1. Signal from carbon atom SiBC= is not observed.

^{11}B NMR ($CDCl_3$, 128 MHz, δ, ppm): 31.20. **^{29}Si NMR** ($CDCl_3$, 80 MHz, δ, ppm): -1.91. **MS (EI)** [m/z (%)]: 313(49), 232(17), 231(100), 230(26), 213(16), 187(25), 171(22), 145(22), 91(36), 83(95), 75(16), 59(15), 55(44).

Elem. Anal. calcd for $C_{25}H_{33}BO_2Si$: C, 74.25; H, 8.22; found C, 74.23; H, 8.21.

Isolated by column chromatography using hexane:ethyl acetate = 99:1 (yield 74%, yellowish crystals).

The compound **3bl** has been synthesized for the first time.

Benzyldimethyl((1*E*,3*E*)-1-(4,4,5,5-tetramethyl-1,3,2-dioxaborolan-2-yl)-4-(p-tolyl)buta-1,3-dien-1-yl)silane (3bm)

^{1}H NMR ($CDCl_3$, 300 MHz, δ, ppm): 7.48 – 7.38 (m, 1H), 7.33 (d, *J* = 8.2 Hz, 2H), 7.25 – 7.13 (m, 5H), 7.10 – 7.03 (m, 4H), 6.63 (d, *J* = 15.5 Hz, 1H), 2.37 (s, 3H), 2.28 (s, 2H), 1.37 (s, 12H), 0.11 (s, 6H).

^{13}C NMR ($CDCl_3$, 75 MHz, δ, ppm): 155.4, 140.7, 138.1, 136.6, 134.7, 129.8, 129.5, 128.5, 128.1, 126.9, 123.9, 83.2, 26.3, 25.2, 21.4, -3.1. Signal from carbon atom SiBC= is not observed.

^{11}B NMR ($CDCl_3$, 128 MHz, δ, ppm): 31.41. **^{29}Si NMR** ($CDCl_3$, 80 MHz, δ, ppm): -1.96.

MS (EI) [m/z (%)]: 327(45), 326(35), 245(100), 244(87), 203(31), 202(41), 201(28), 144(25), 143(28), 92(34), 84(57), 83(46), 56(49), 55(27).

Elem. Anal. calcd for $C_{25}H_{35}BO_2Si$: C, 74.63; H, 8.43; found C, 74.60; H, 8.42.

Isolated by column chromatography using hexane:ethyl acetate = 99:1 (yield 60%, yellow oil).

The compound **3bm** has been synthesized for the first time.

Benzyldimethyl((1*E*,3*E*)-1-(4,4,5,5-tetramethyl-1,3,2-dioxaborolan-2-yl)-4-(4-(trifluoromethyl)phenyl)buta-1,3-dien-1-yl)silane (3bn)

^{1}H NMR ($CDCl_3$, 300 MHz, δ, ppm): 7.59 (d, *J* = 8.3 Hz, 2H), 7.54 – 7.47 (m, 3H), 7.23 – 7.18 (m, 2H), 7.09 – 7.00 (m, 4H), 6.64 (d, *J* = 15.5 Hz, 1H), 2.28 (s, 2H), 1.37 (s, 12H), 0.11 (s, 6H).

^{13}C NMR ($CDCl_3$, 101 MHz, δ, ppm): 154.3, 141.0 (d, J = 1.3 Hz), 140.5, 134.6, 132.8, 129.6 (q, *J* = 32.4 Hz), 128.5, 128.2, 127.0, 125.7 (q, *J* = 3.6 Hz), 125.1 (q, *J* = 272.9 Hz), 124.0, 83.5, 26.2, 25.2, -3.1. Signal from carbon atom SiBC= is not observed.

^{11}B NMR ($CDCl_3$, 128 MHz, δ, ppm): 30.84. **^{29}Si NMR** ($CDCl_3$, 80 MHz, δ, ppm): -1.74.

^{19}F NMR ($CDCl_3$, 377 MHz, δ, ppm): -62.52. **MS (EI)** [m/z (%)]: 381(6), 381(8), 299(19), 299(11), 85(8), 83(100), 55(23).

Elem. Anal. calcd for $C_{26}H_{32}BF_3O_2Si$: C, 66.10; H, 6.83; found C, 66.08; H, 6.83.

Isolated by column chromatography using hexane:ethyl acetate = 200:1 (yield 62%, yellow oil).

The compound **3bn** has been synthesized for the first time.

(*E*)-(1,2-diphenylvinyl)triethylsilane (4aaa)

^{1}H NMR ($CDCl_3$, 300 MHz, δ, ppm): 7.19 (dd, *J* = 22.8, 7.4 Hz, 3H), 7.03 (dd, *J* = 5.0, 1.8 Hz, 3H), 6.98 – 6.85 (m, 4H), 6.72 (s, 1H), 0.91 (t, *J* = 7.8 Hz, 9H), 0.60 (q, *J* = 7.8 Hz, 6H). **^{13}C NMR** ($CDCl_3$, 101 MHz, δ, ppm): 144.3, 143.3, 138.9, 137.6, 129.7, 128.7, 128.0, 127.4, 127.1, 125.7, 7.5, 2.9.

^{29}Si NMR ($CDCl_3$, 80 MHz, δ, ppm): 3.09.

Isolated by column chromatography using hexane:ethyl acetate = 99:1 (yield 62%, colorless oil.

The NMR spectra are in agreement with the literature [59].

(*E*)-benzyl(1,2-diphenylvinyl)dimethylsilane (4baa)

^{1}H NMR ($CDCl_3$, 300 MHz, δ, ppm): 7.47 (t, *J* = 7.4 Hz, 2H), 7.40 – 7.34 (m, 3H), 7.30 – 7.22 (m, 4H), 7.20 – 7.13 (m, 4H), 7.10 (dd, *J* = 6.6, 3.0 Hz, 2H), 6.94 (s, 1H), 2.37 (s, 2H), 0.26 (s, 6H).

^{13}C NMR ($CDCl_3$, 101 MHz, δ, ppm): 145.4, 142.6, 139.9, 138.8, 137.3, 129.6, 128.8, 128.5, 128.3, 128.1, 127.7, 127.3, 125.9, 124.2, 25.1, -3.4.

^{29}Si NMR ($CDCl_3$, 80 MHz, δ, ppm): -2.89.

Isolated by column chromatography using hexane:ethyl acetate = 99:1 (yield 65%, white crystals). The NMR spectra are in agreement with the literature [60].

(*E*)-Benzyl(2-(4-methoxyphenyl)-1-(4-nitrophenyl)vinyl)dimethylsilane (4bef)

^{1}H NMR ($CDCl_3$, 300 MHz, δ, ppm): 8.20 – 8.14 (m, 2H), 7.24 – 7.18 (m, 2H), 7.13 – 7.07 (m, 3H), 7.02 – 6.97 (m, 2H), 6.86 – 6.80 (m, 3H), 6.70 – 6.63 (m, 2H), 3.74 (s, 3H), 2.21 (s, 2H), 0.12 (s, 6H).

^{13}C NMR ($CDCl_3$, 101 MHz, δ, ppm): 159.3, 151.3, 146.2, 140.8, 139.6, 139.3, 131.0, 129.2, 128.8, 128.5, 128.4, 124.5, 124.2, 113.7, 55.3, 25.4, -3.2.

^{29}Si NMR ($CDCl_3$, 80 MHz, δ, ppm): -2.24.

MS (EI) [m/z (%)]: 314(25), 313(91), 312(100), 298(13), 266(15), 207(15), 204(16), 189(15), 180(15), 165(37), 165(17), 164(20), 92(27), 59(36), 58(24).

Elem. Anal. calcd for $C_{22}H_{25}NO_3Si$: C, 71.43; H, 6.24; found C, 71.44; H, 6.24.

Isolated by column chromatography using gradient elution with hexane → hexane:ethyl acetate = 97:3 (yield 52%, yellow oil).

The compound **4bef** has been synthesized for the first time.

(*E*)-Benzyl(1-(4-methoxyphenyl)-2-(4-nitrophenyl)vinyl)dimethylsilane (4bfe)

^{1}H NMR ($CDCl_3$, 300 MHz, δ, ppm): 8.02 – 7.90 (m, 2H), 7.25 – 7.14 (m, 3H), 7.12 – 7.05 (m, 3H), 7.02 – 6.97 (m, 2H), 6.86 (d, J = 1.1 Hz, 3H), 6.78 (s, 1H), 3.83 (s, 3H), 2.21 (s, 2H), 0.13 (s, 6H).

^{13}C NMR ($CDCl_3$, 101 MHz, δ, ppm): 158.4, 150.7, 146.3, 144.0, 139.5, 136.8, 133.4, 130.1, 128.5, 128.5, 128.4, 124.4, 123.4, 114.6, 55.3, 25.0, -3.4.

^{29}Si NMR ($CDCl_3$, 80 MHz, δ, ppm): -2.34.

MS (EI) [m/z (%)]: 313(91), 312(35), 268(24), 267(86), 266(28), 252(34), 251(31), 165(100), 164(82), 122(39), 120(30), 92(37), 91(47), 59(49), 58(42).

Elem. Anal. calcd for $C_{24}H_{25}NO_3Si$: C, 71.43; H, 6.24; found C, 71.42; H, 6.23.

Isolated by column chromatography using gradient elution with hexane → hexane:ethyl acetate = 97:3 (yield 45%, yellow oil).

The compound **4bfe** has been synthesized for the first time.

((1*E*,3*E*)-1,4-Diphenylbuta-1,3-dien-1-yl)triethylsilane (4ala)

^{1}H NMR ($CDCl_3$, 400 MHz, δ, ppm): 7.35 – 7.30 (m, 2H), 7.27 – 7.12 (m, 6H), 7.08 – 6.97 (m, 2H), 6.79 – 6.55 (m, 3H), 0.93 (t, *J* = 7.9 Hz, 9H), 0.63 (q, *J* = 7.8 Hz, 6H).

^{13}C NMR ($CDCl_3$, 101 MHz, δ, ppm) δ 145.1, 142.9, 140.1, 137.5, 134.0, 128.6, 128.2, 128.1, 127.7, 126.7, 126.4, 125.8, 7.5, 3.1.

^{29}Si NMR ($CDCl_3$, 80 MHz, δ, ppm): 2.24.

MS (EI) [m/z (%)]: 320(49), 291(60), 263(20), 204(80), 159(19), 135(38), 115(31), 107(46), 87(61), 59(100).

Elem. Anal. calcd for $C_{22}H_{28}Si$: C, 82.43; H, 8.80; found C, 82.40; H, 9.40.

Isolated by column chromatography using hexane:ethyl acetate = 99:1 (yield 46%, yellow oil).

The compound **4ala** has been synthesized for the first time.

3. NMR spectra of products

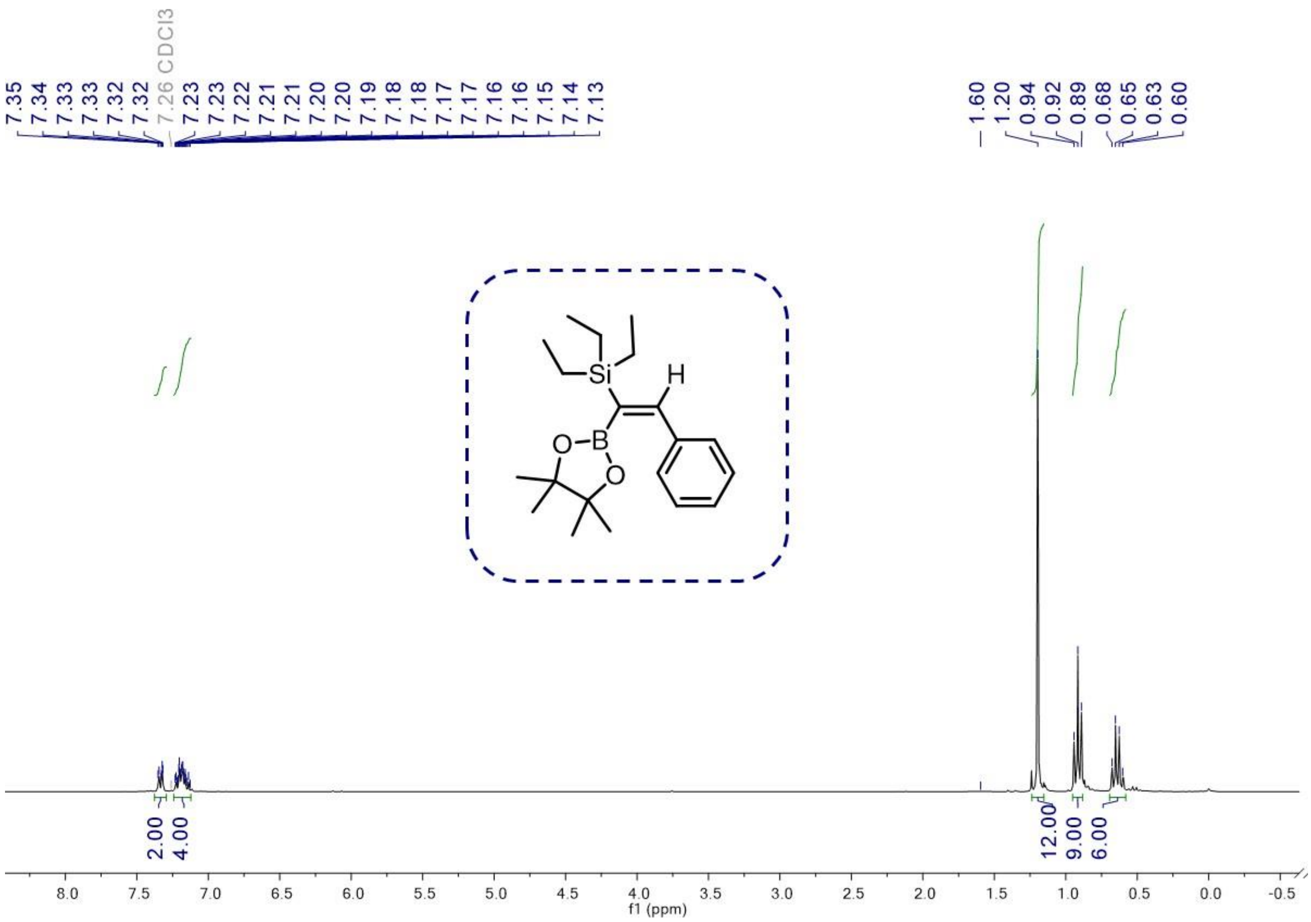

Figure S1. ^{1}H NMR spectrum of compound **3aa**.

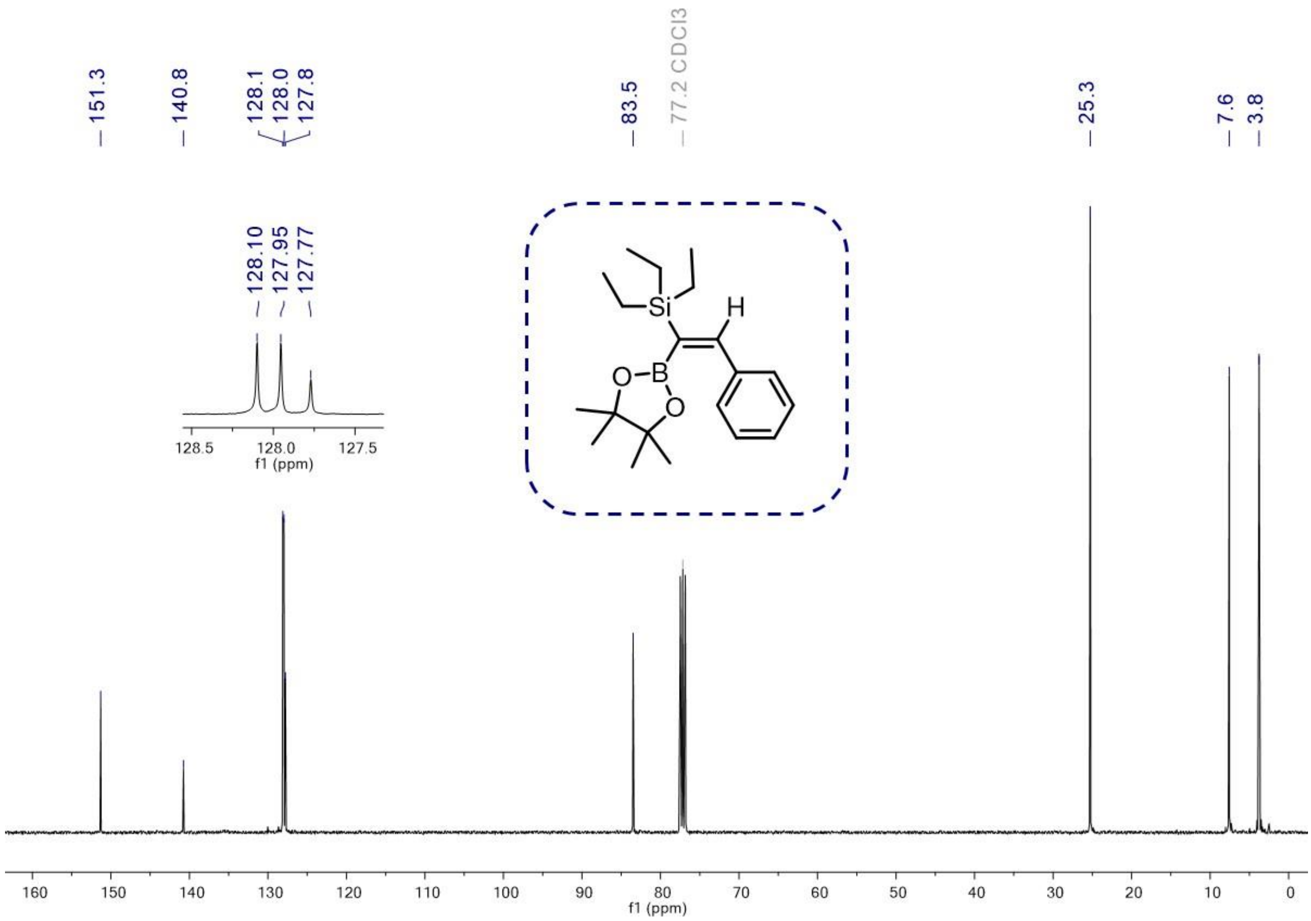

Figure S2. ^{13}C NMR spectrum of compound **3aa**.

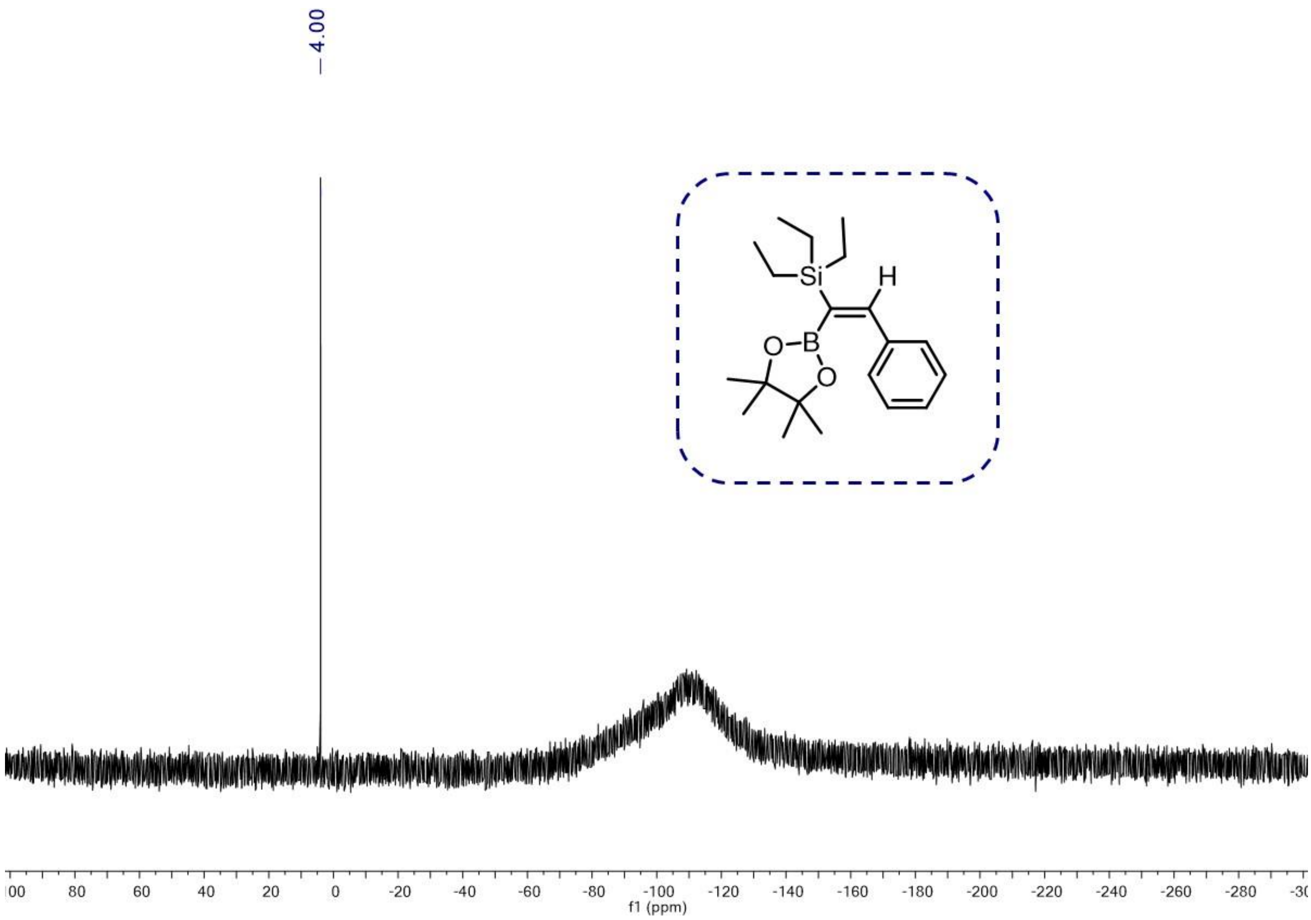

Figure S3. ^{29}Si NMR spectrum of compound **3aa**.

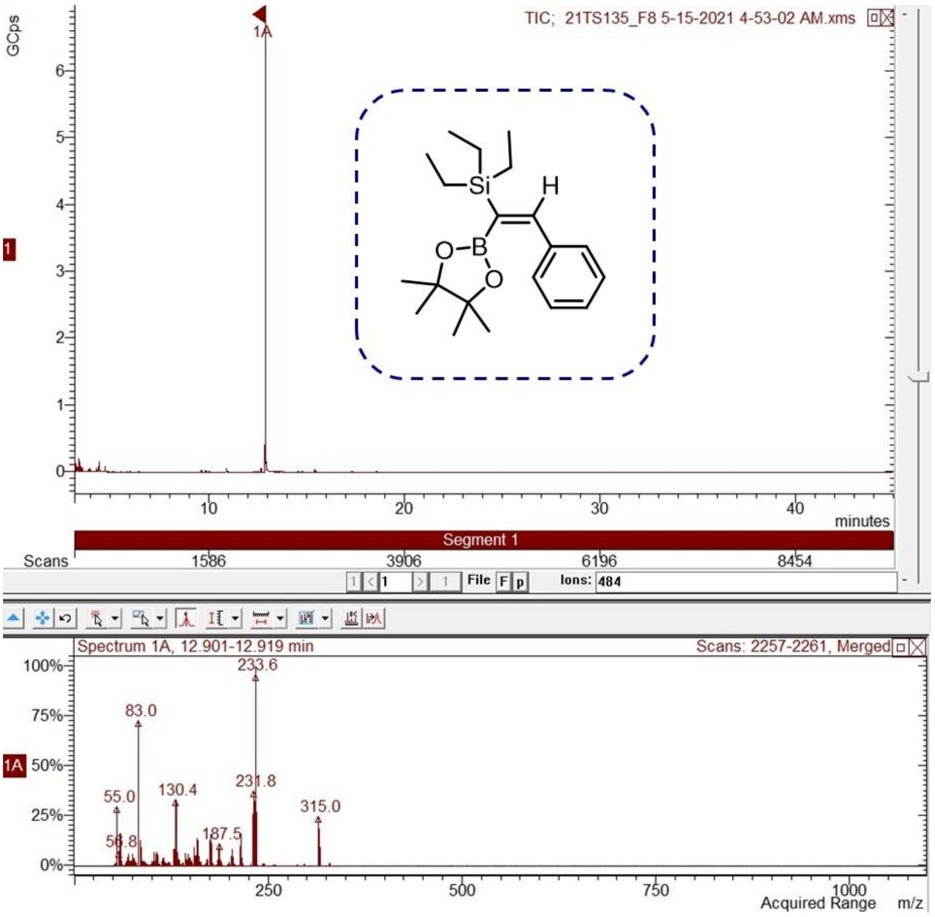

Figure S4. GC-MS image of compound **3aa**.

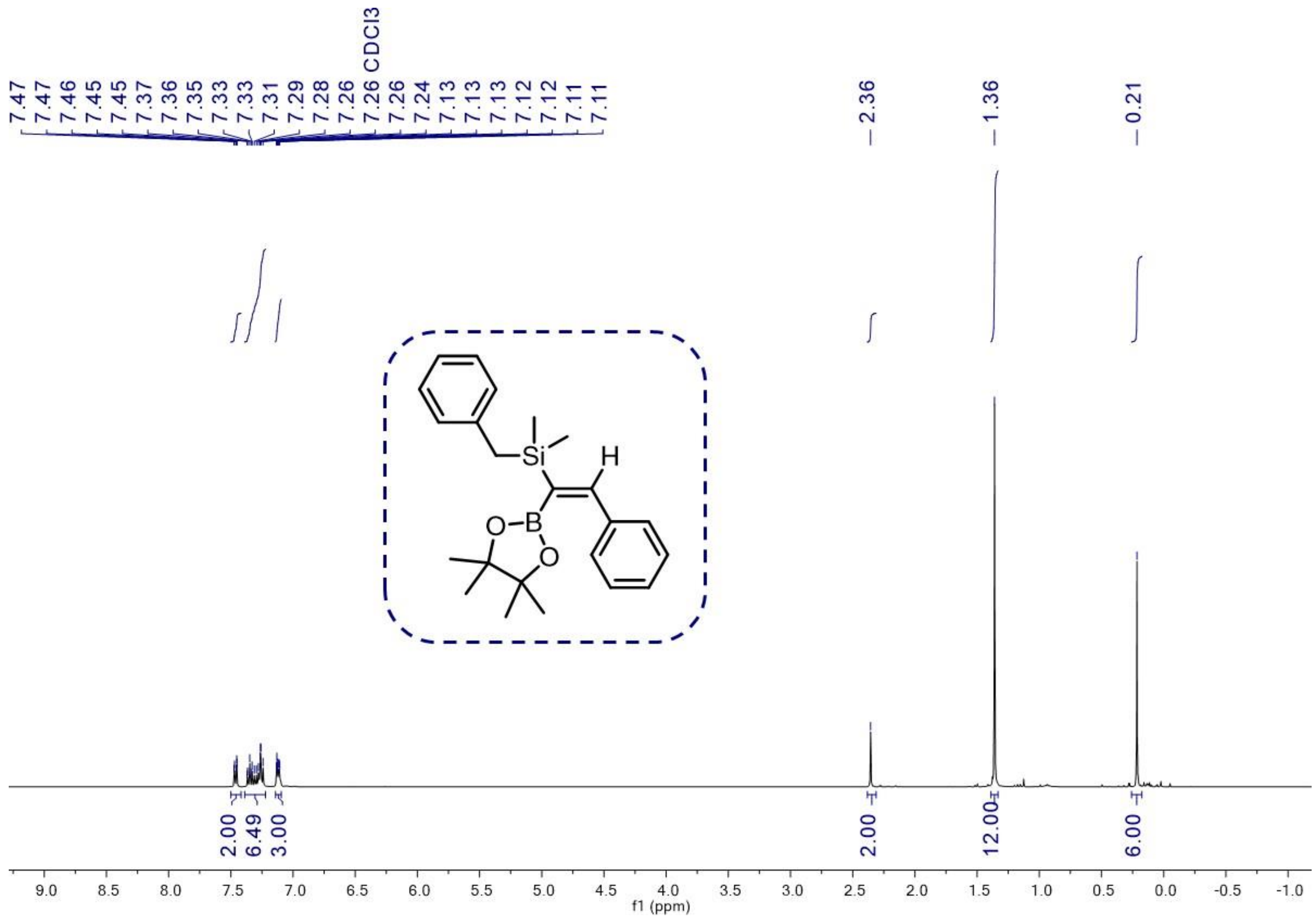

Figure S5. ^{1}H NMR spectrum of compound **3ba**.

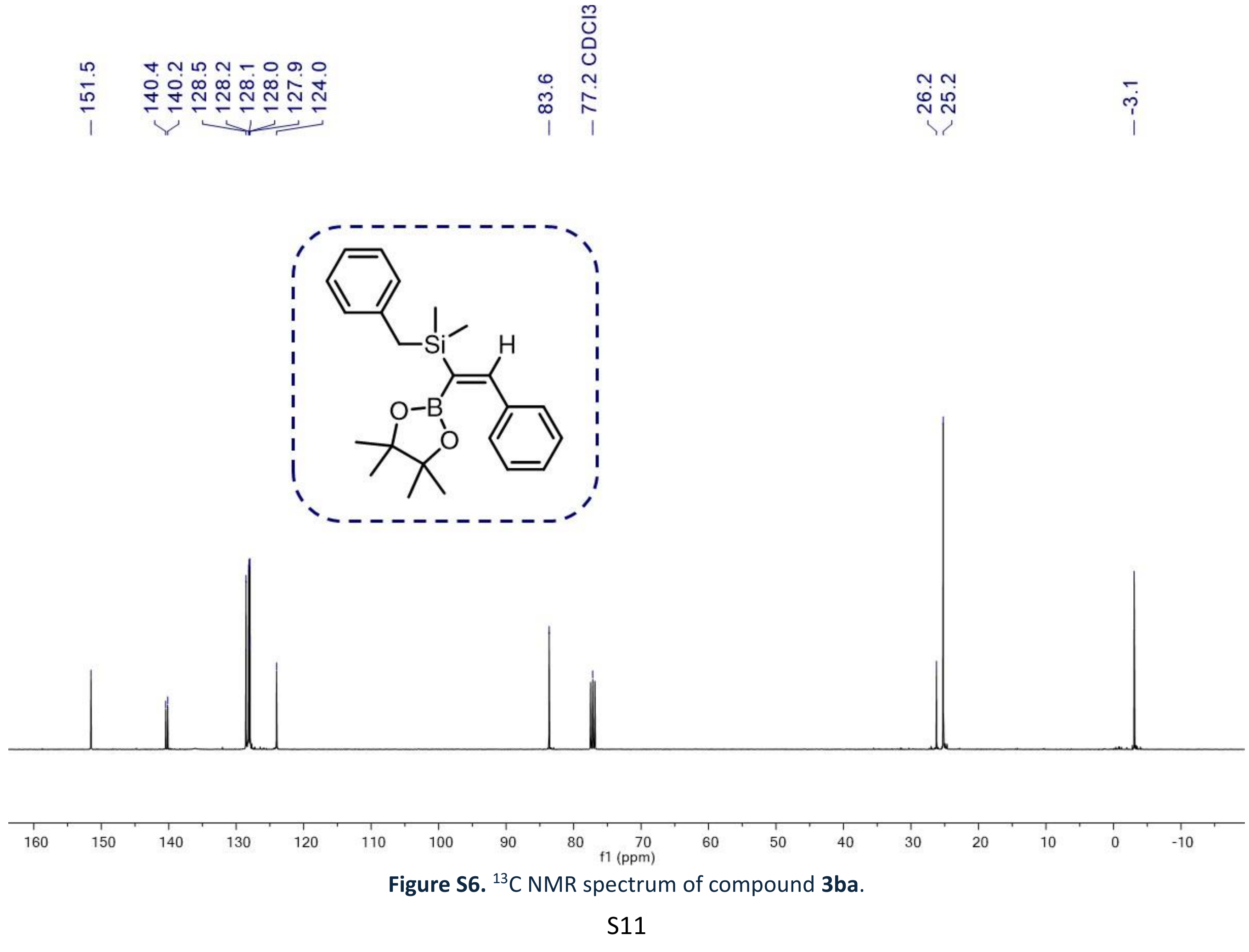

Figure S6. ^{13}C NMR spectrum of compound **3ba**.

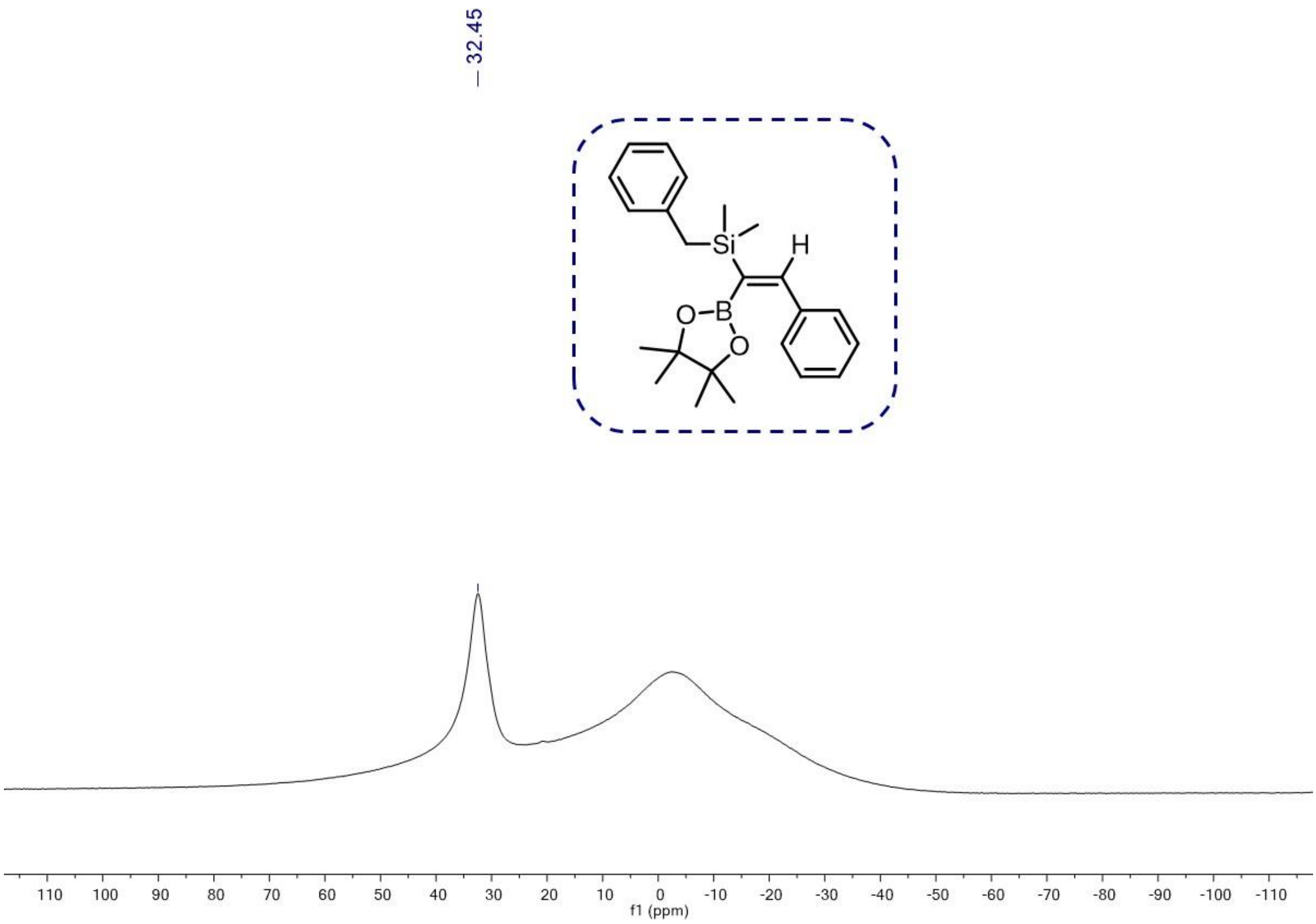

Figure S7. ^{11}B NMR spectrum of compound **3ba**.

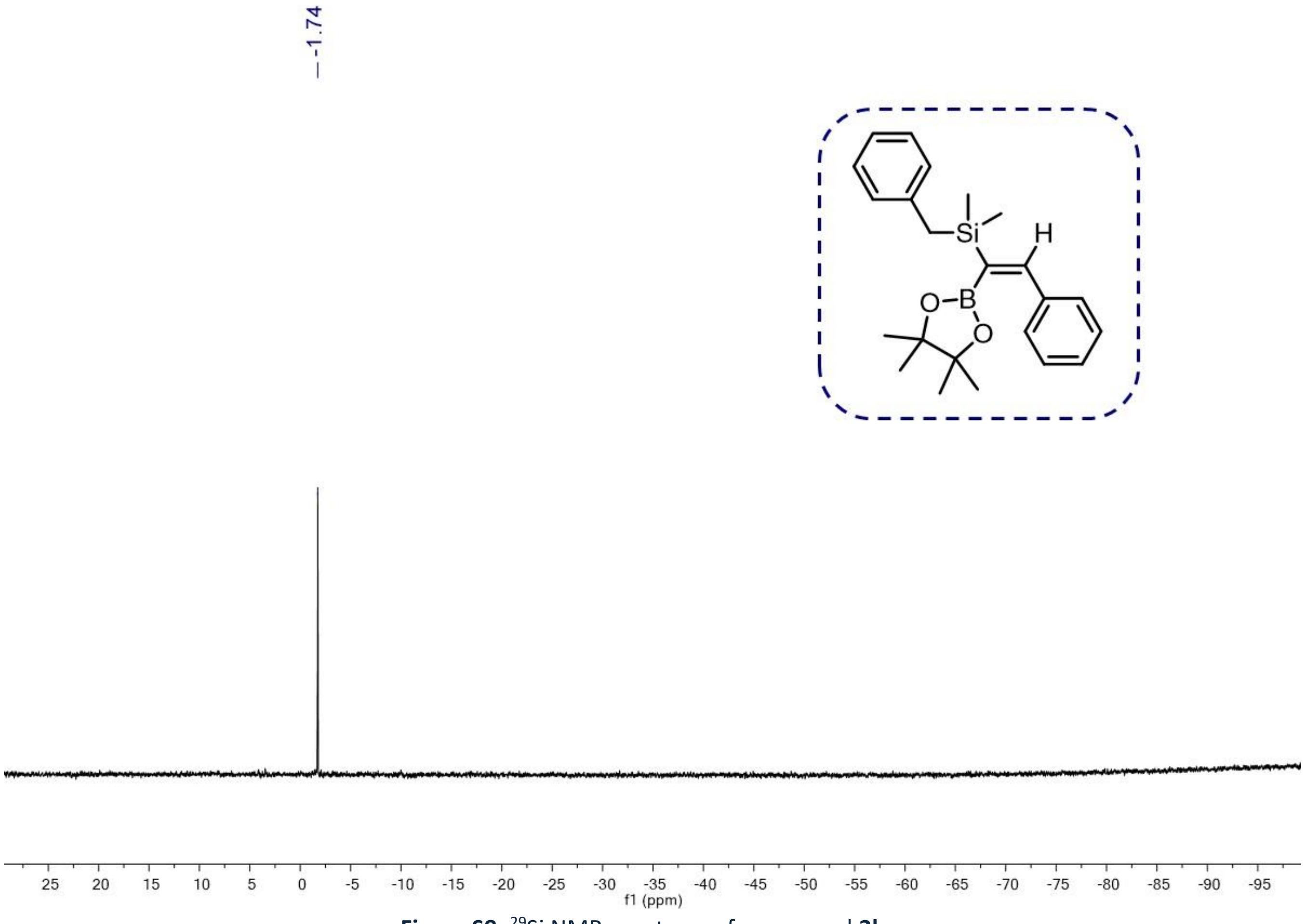

Figure S8. ^{29}Si NMR spectrum of compound **3ba**.

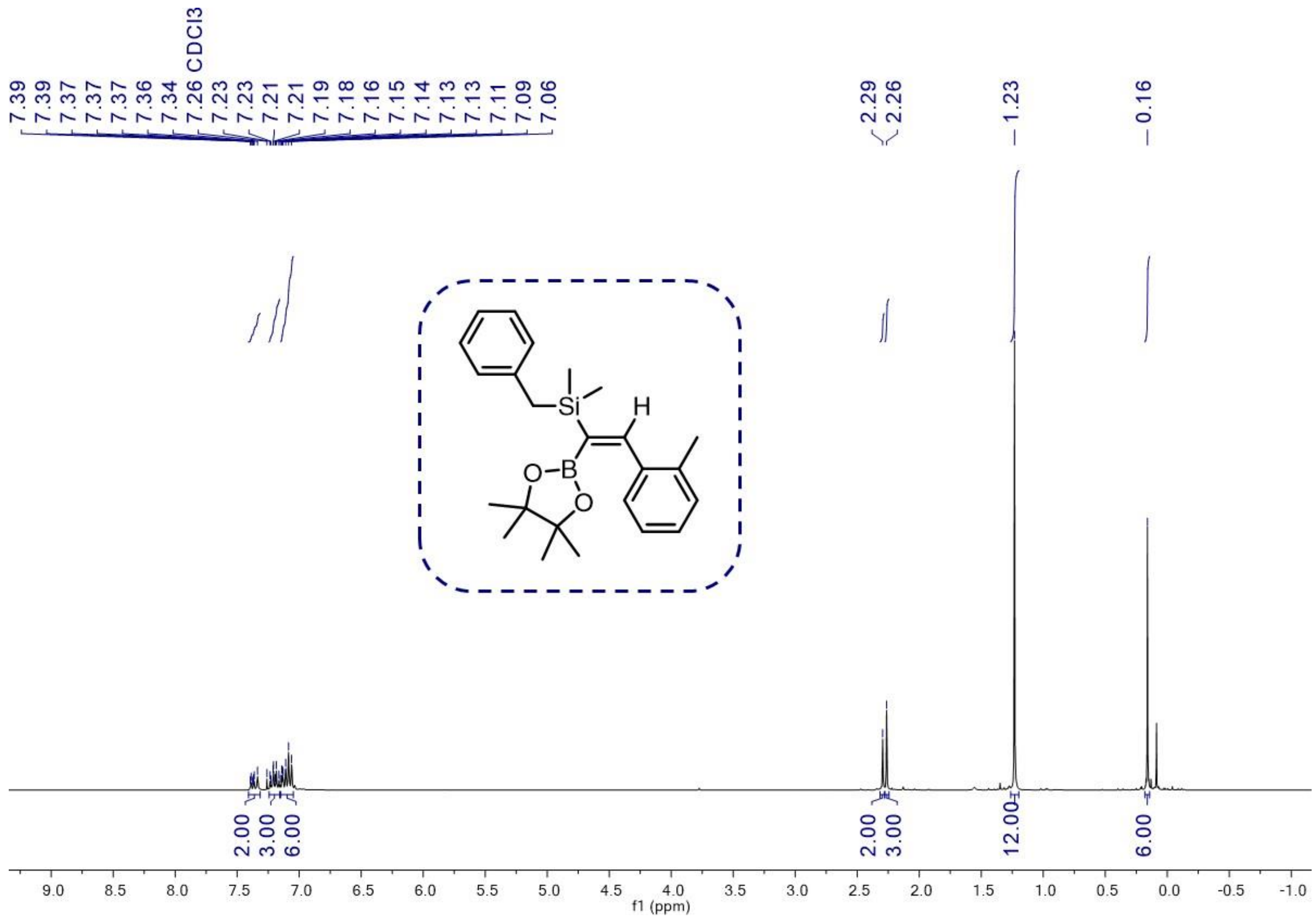

Figure S9. ^{1}H NMR spectrum of compound **3bb**.

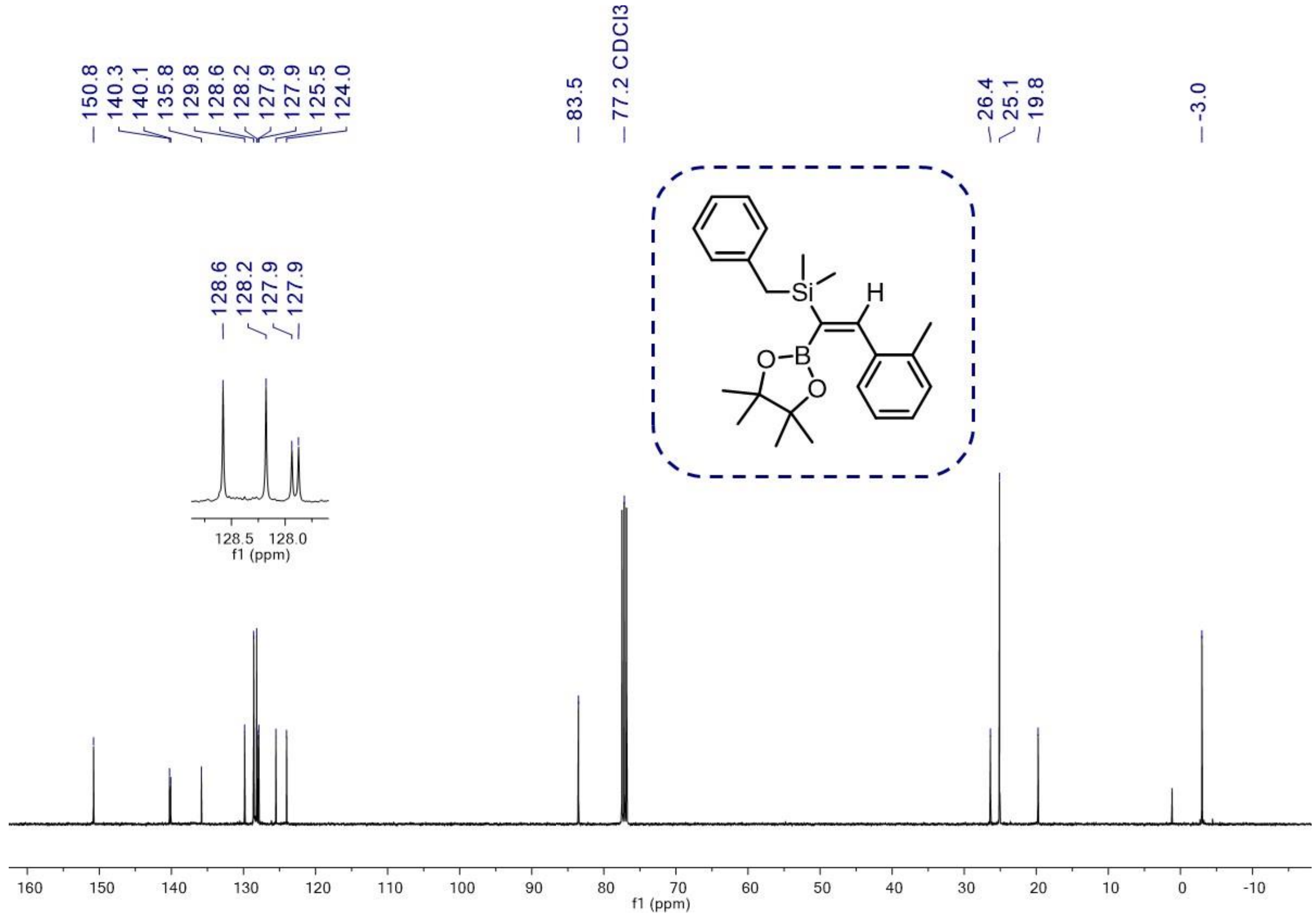

Figure S10. ^{13}C NMR spectrum of compound **3bb**.

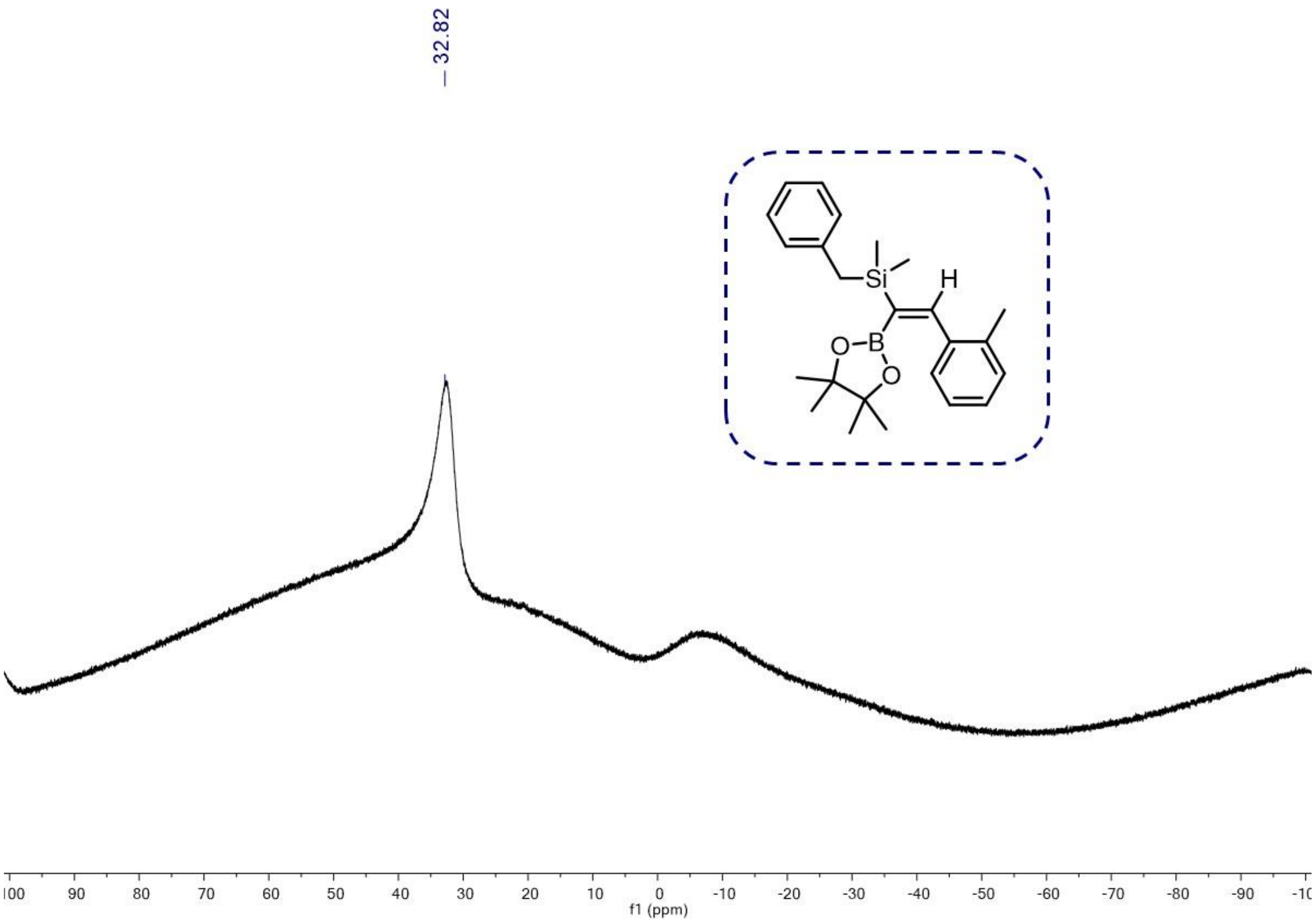

Figure S11. ^{11}B NMR spectrum of compound **3bb**.

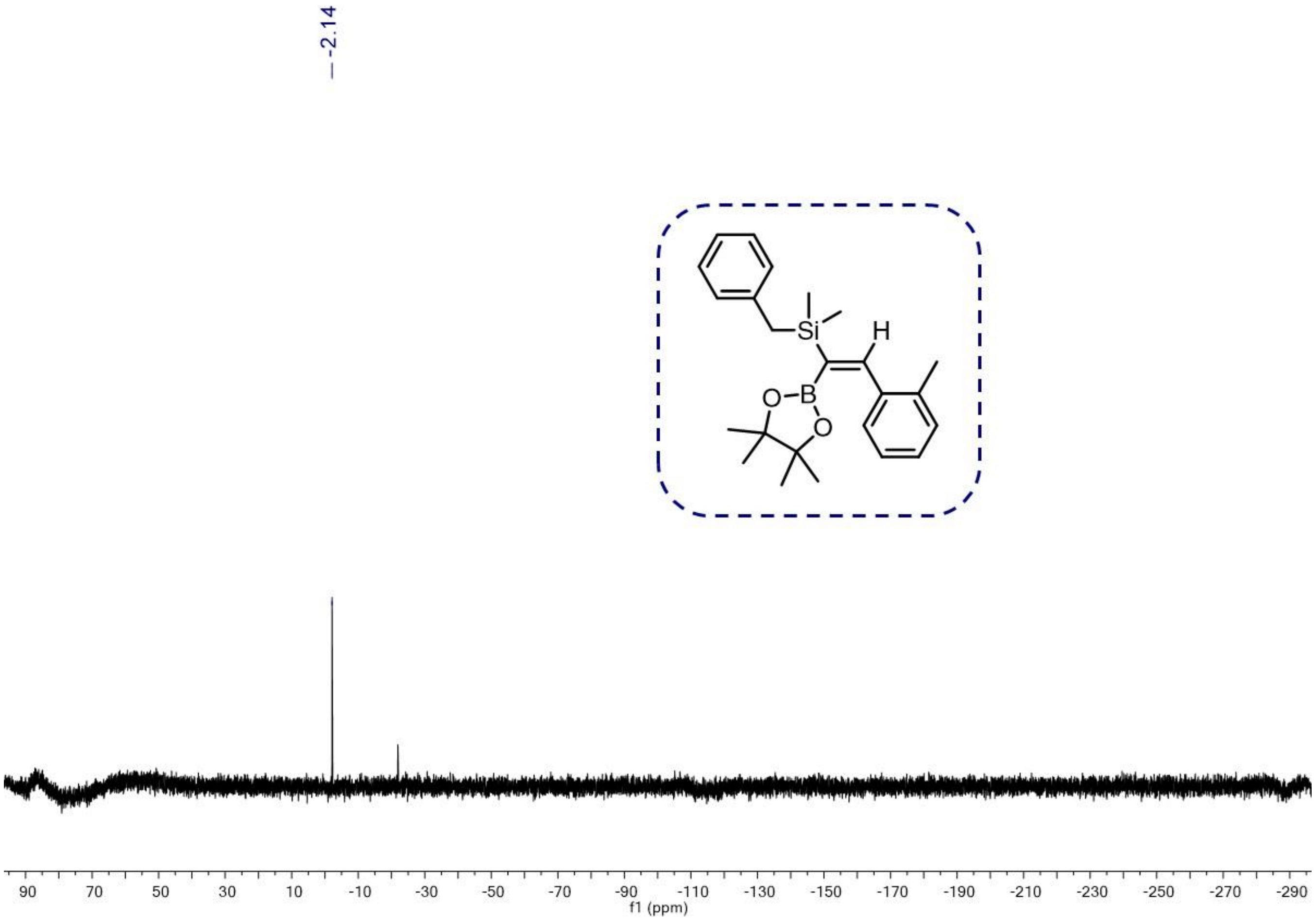

Figure S12. ^{29}Si NMR spectrum of compound **3bb**.

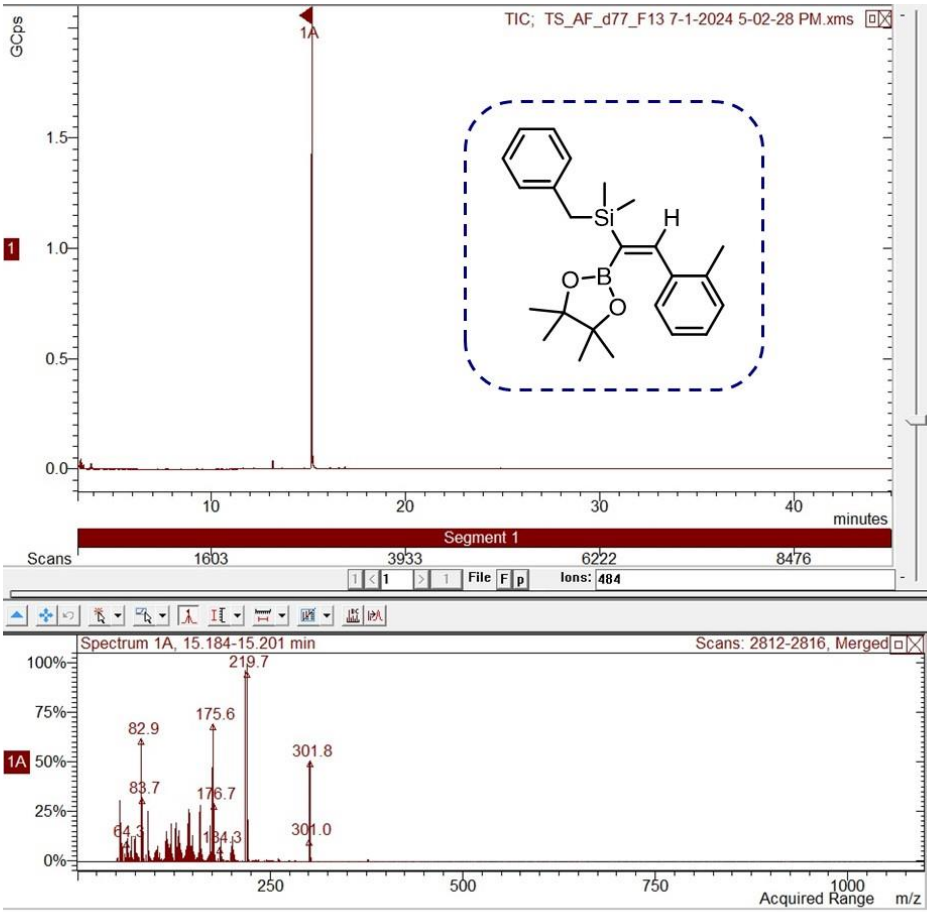

Figure S13. GC-MS image of compound **3bb**.

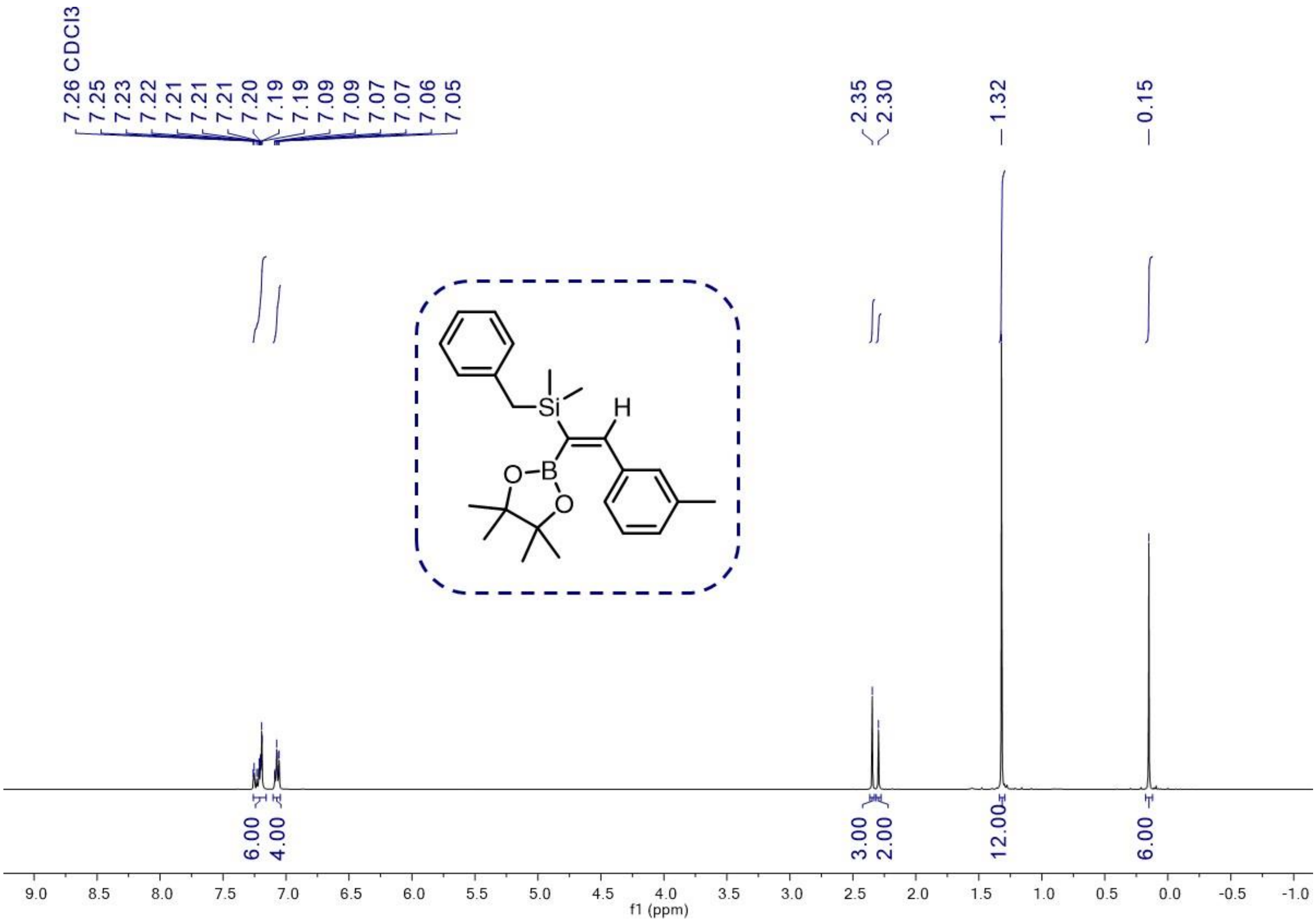

Figure S14. ^{1}H NMR spectrum of compound **3bc**.

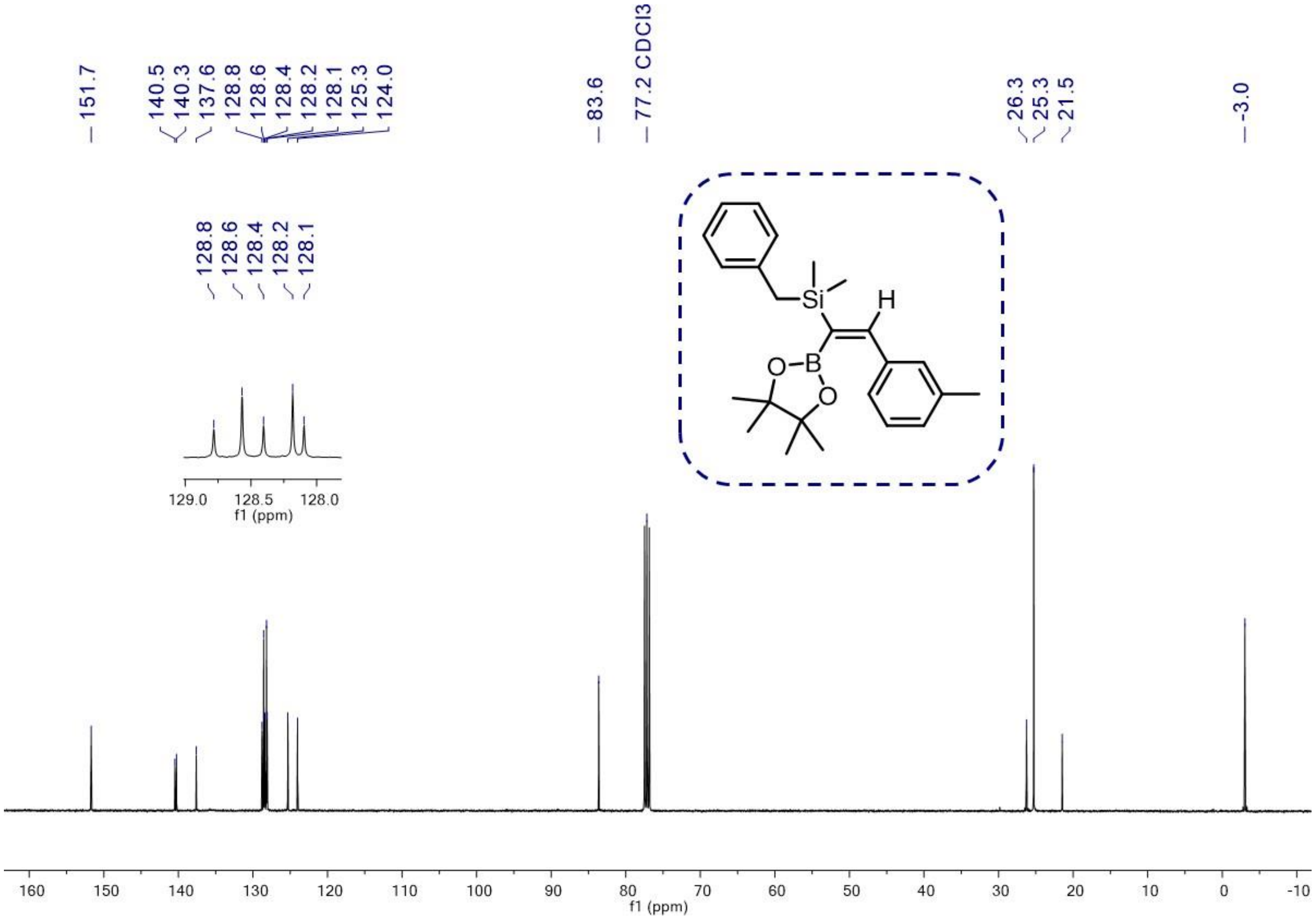

Figure S15. ^{13}C NMR spectrum of compound **3bc**.

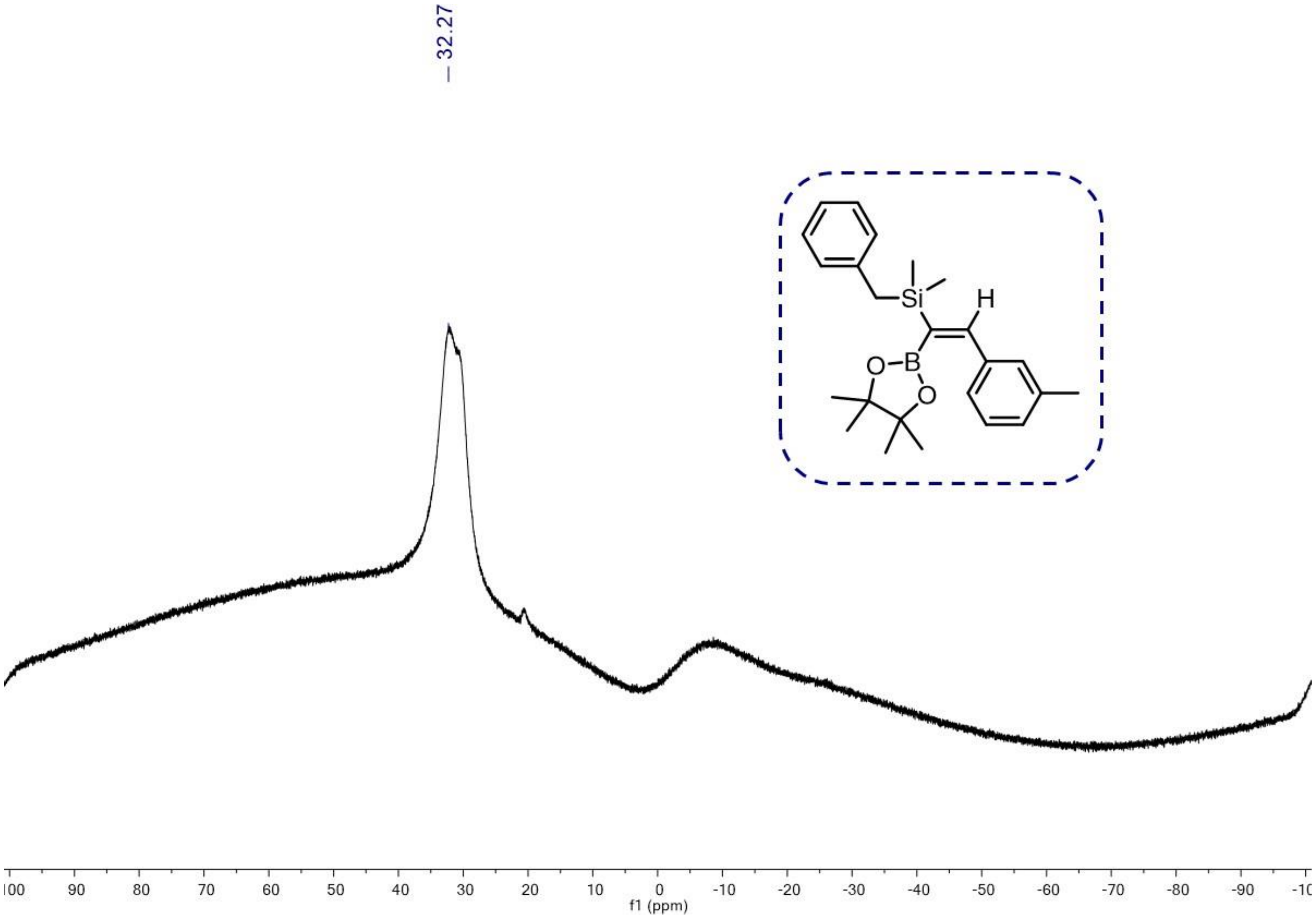

Figure S16. ^{11}B NMR spectrum of compound **3bc**.

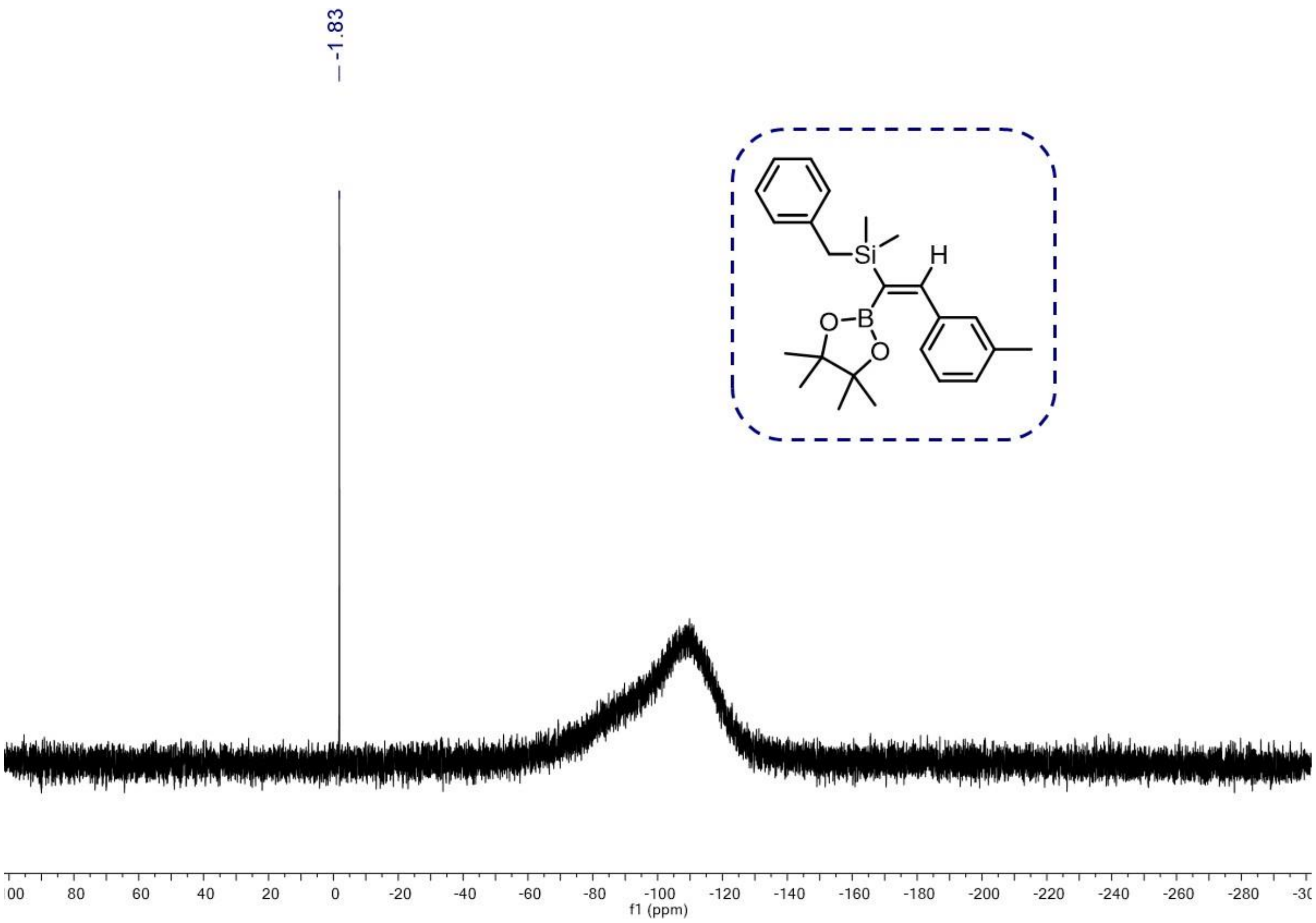

Figure S17. ^{29}Si NMR spectrum of compound **3bc**.

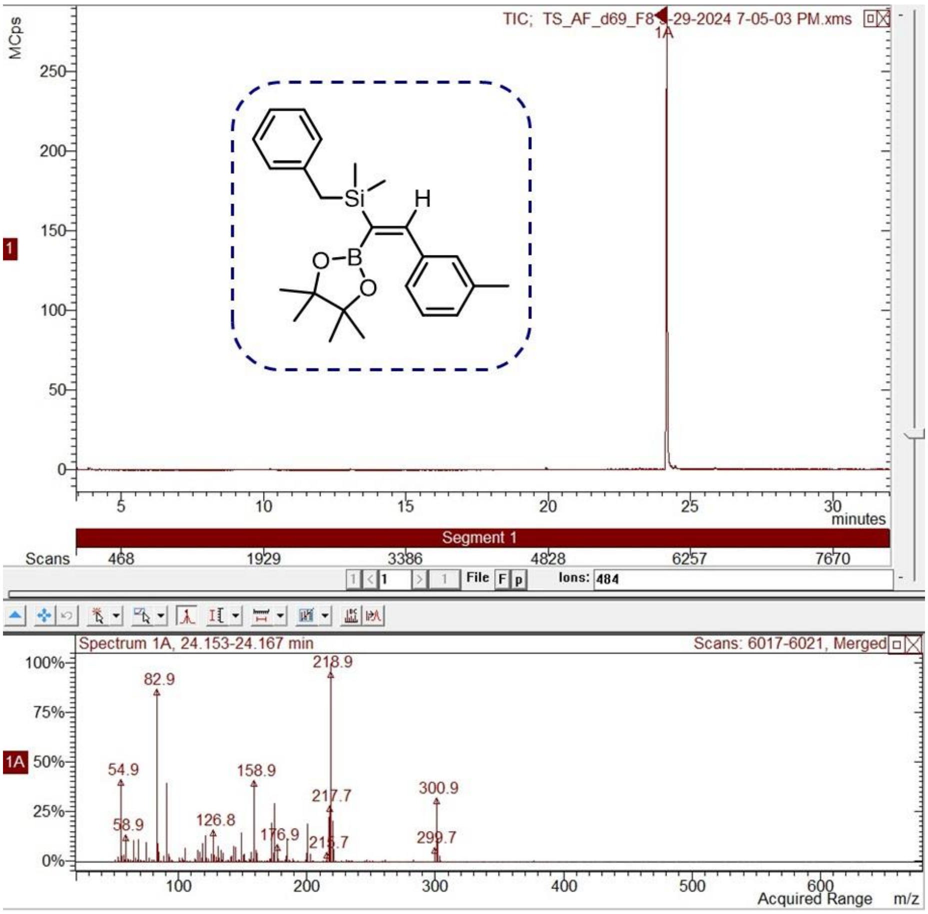

Figure S18. GC-MS image spectrum of compound **3bc**.

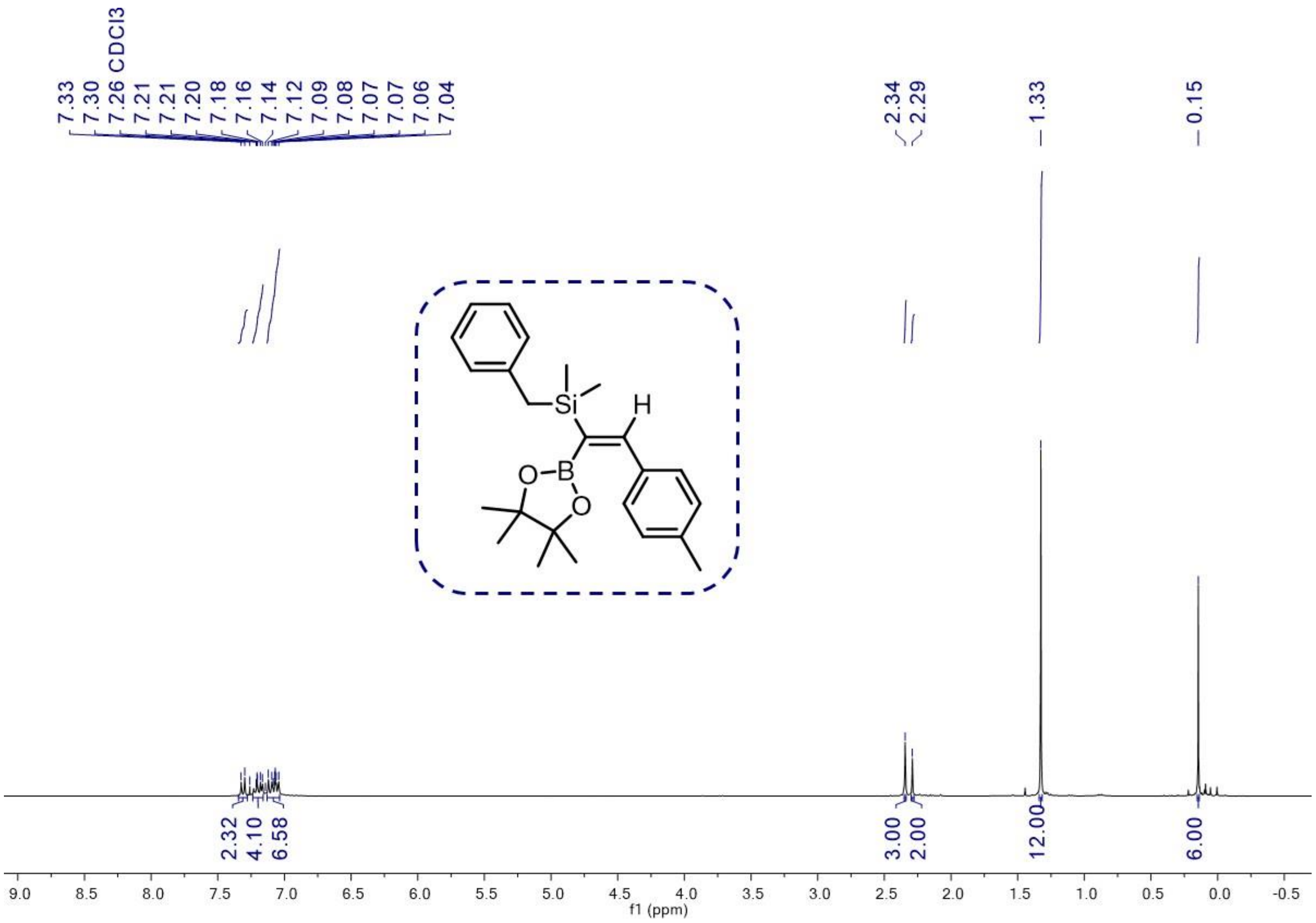

Figure S19. ^{1}H NMR spectrum of compound **3bd**.

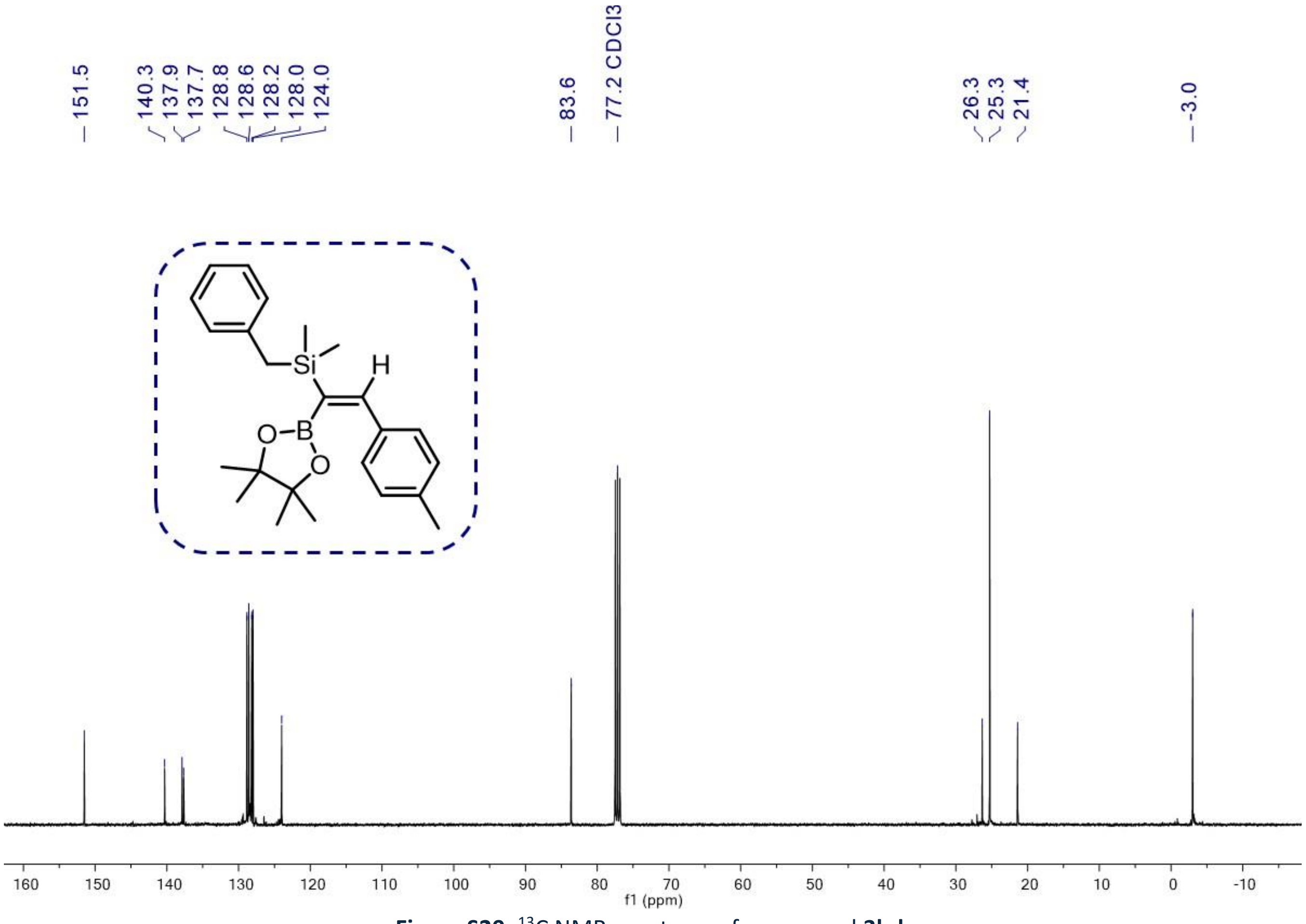

Figure S20. ^{13}C NMR spectrum of compound **3bd**.

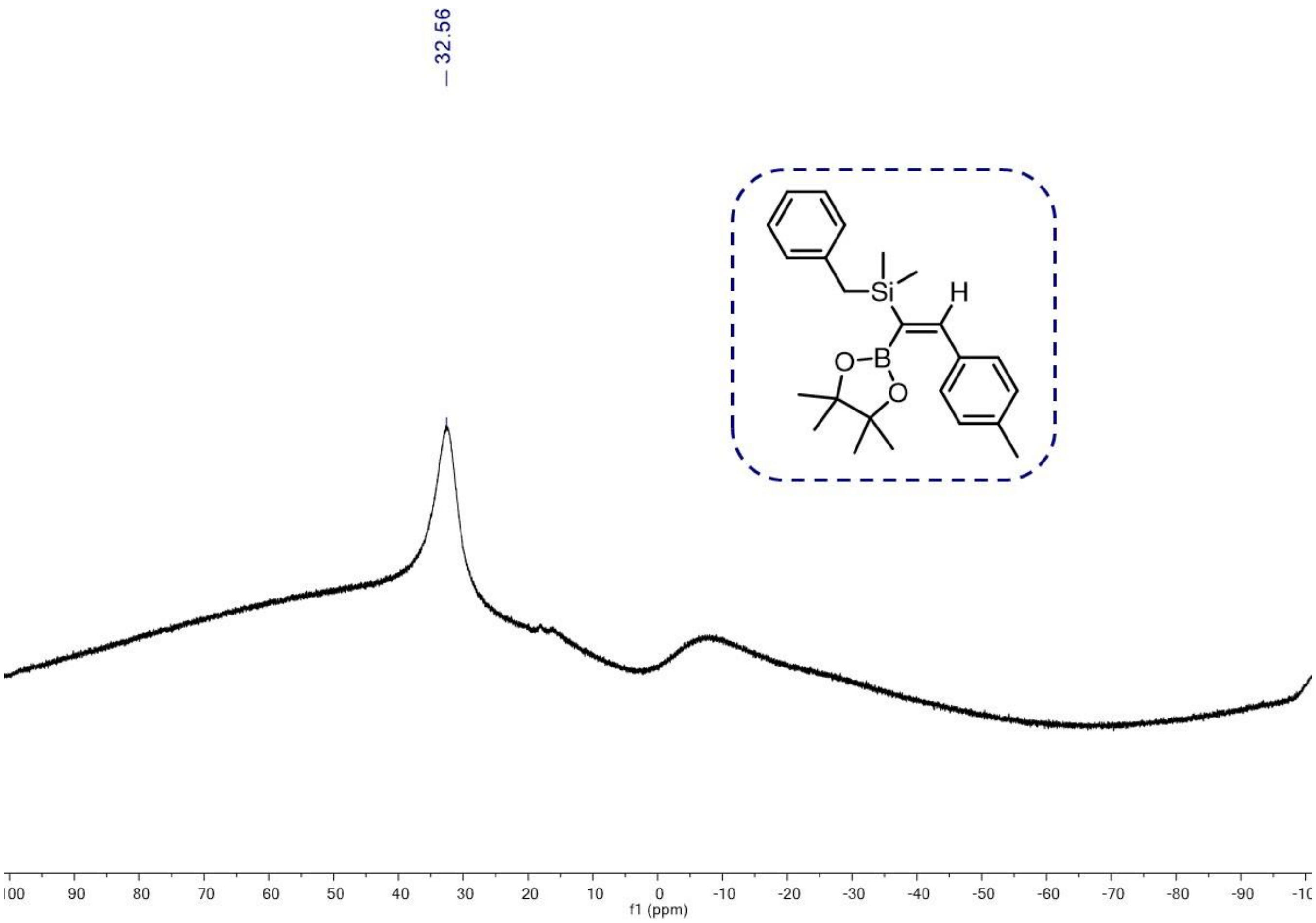

Figure S21. ^{11}B NMR spectrum of compound **3bd**.

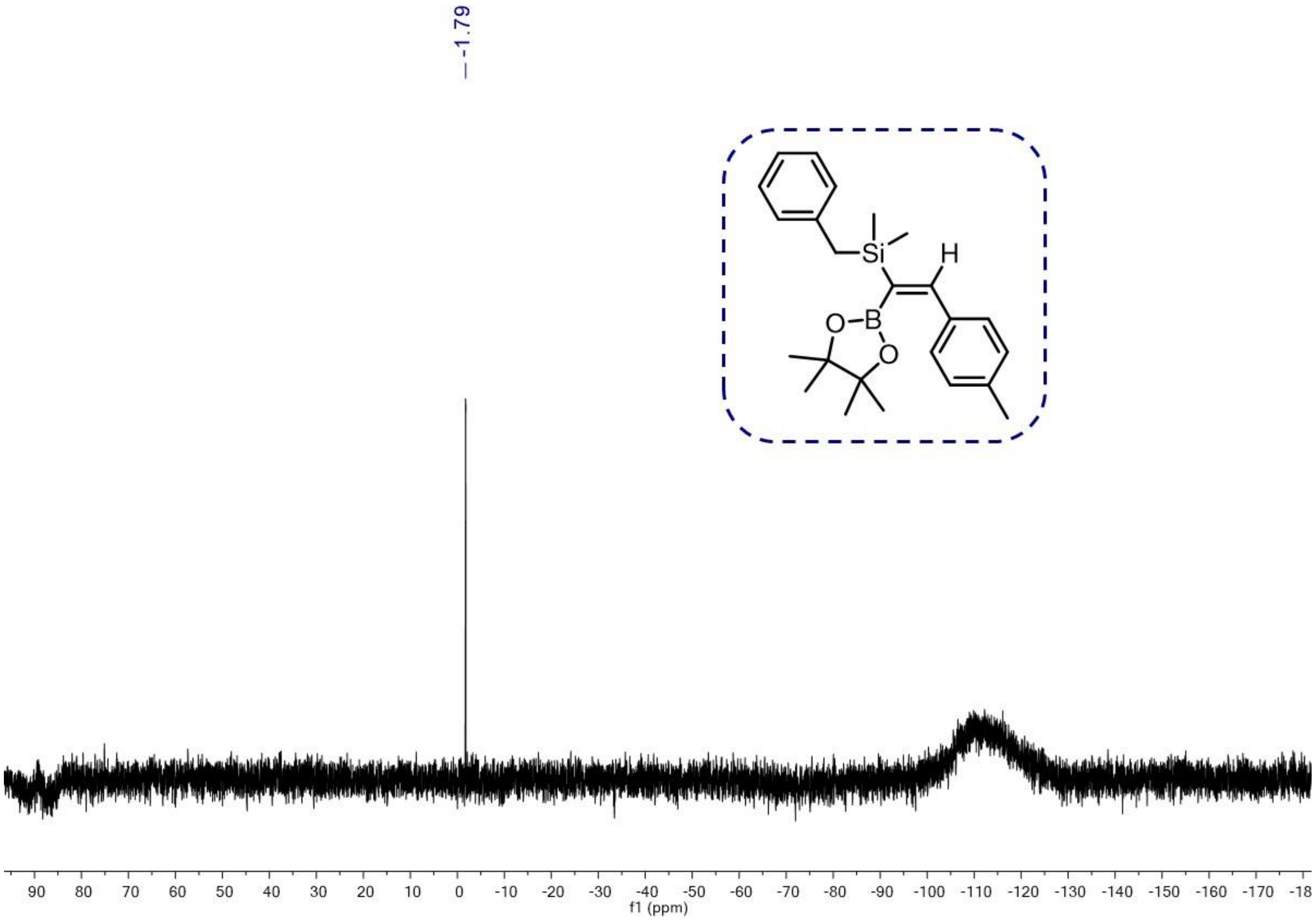

Figure S22. ^{29}Si NMR spectrum of compound **3bd**.

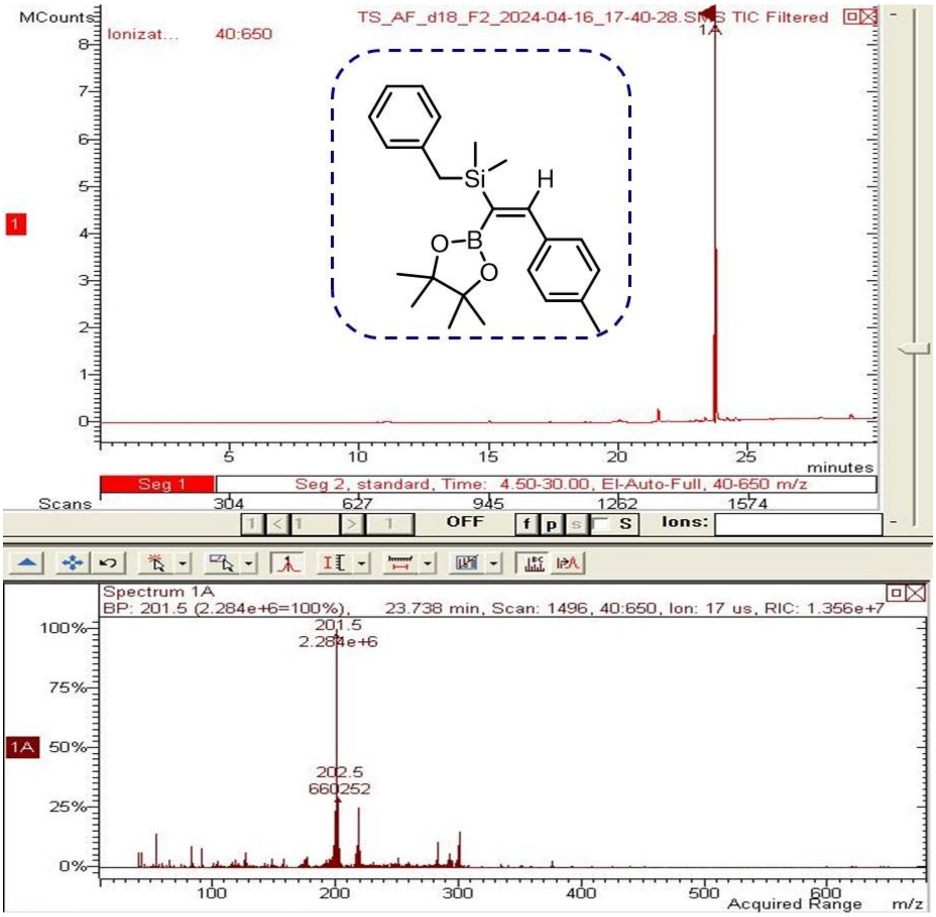

Figure S23. ^{29}Si NMR spectrum of compound **3bd**.

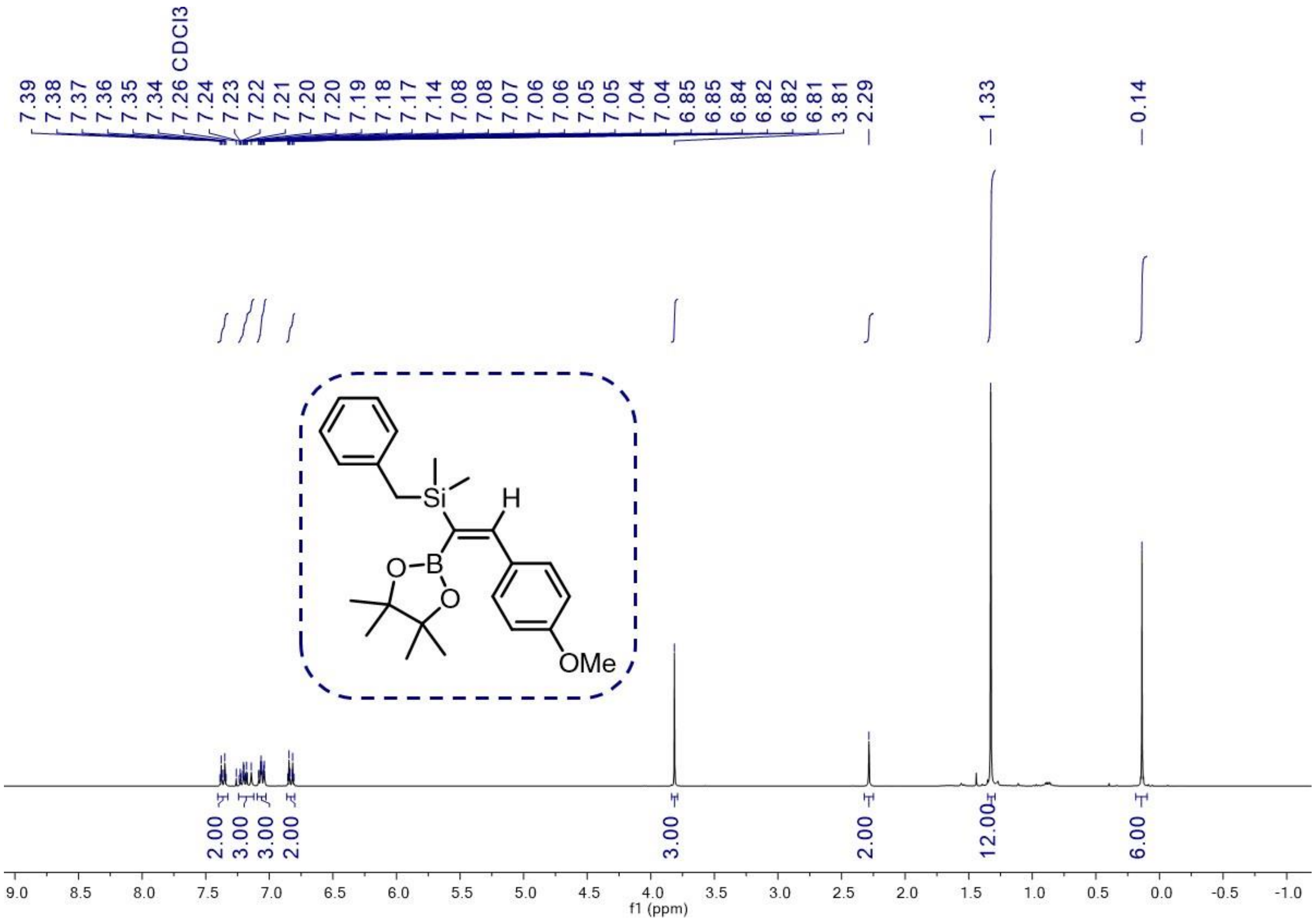

Figure S24. ^{1}H NMR spectrum of compound **3be**.

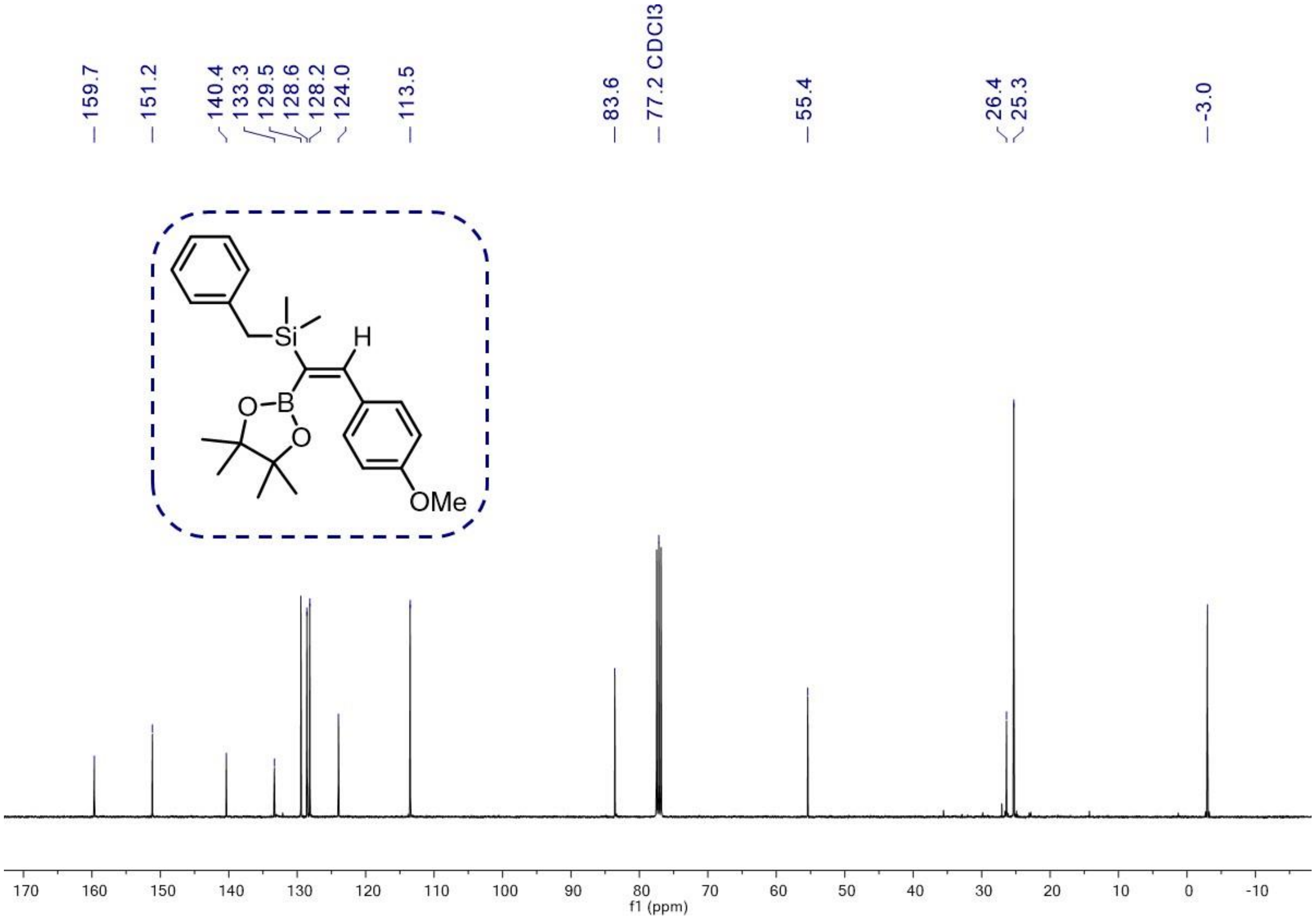

Figure S25. ^{13}C NMR spectrum of compound **3be**.

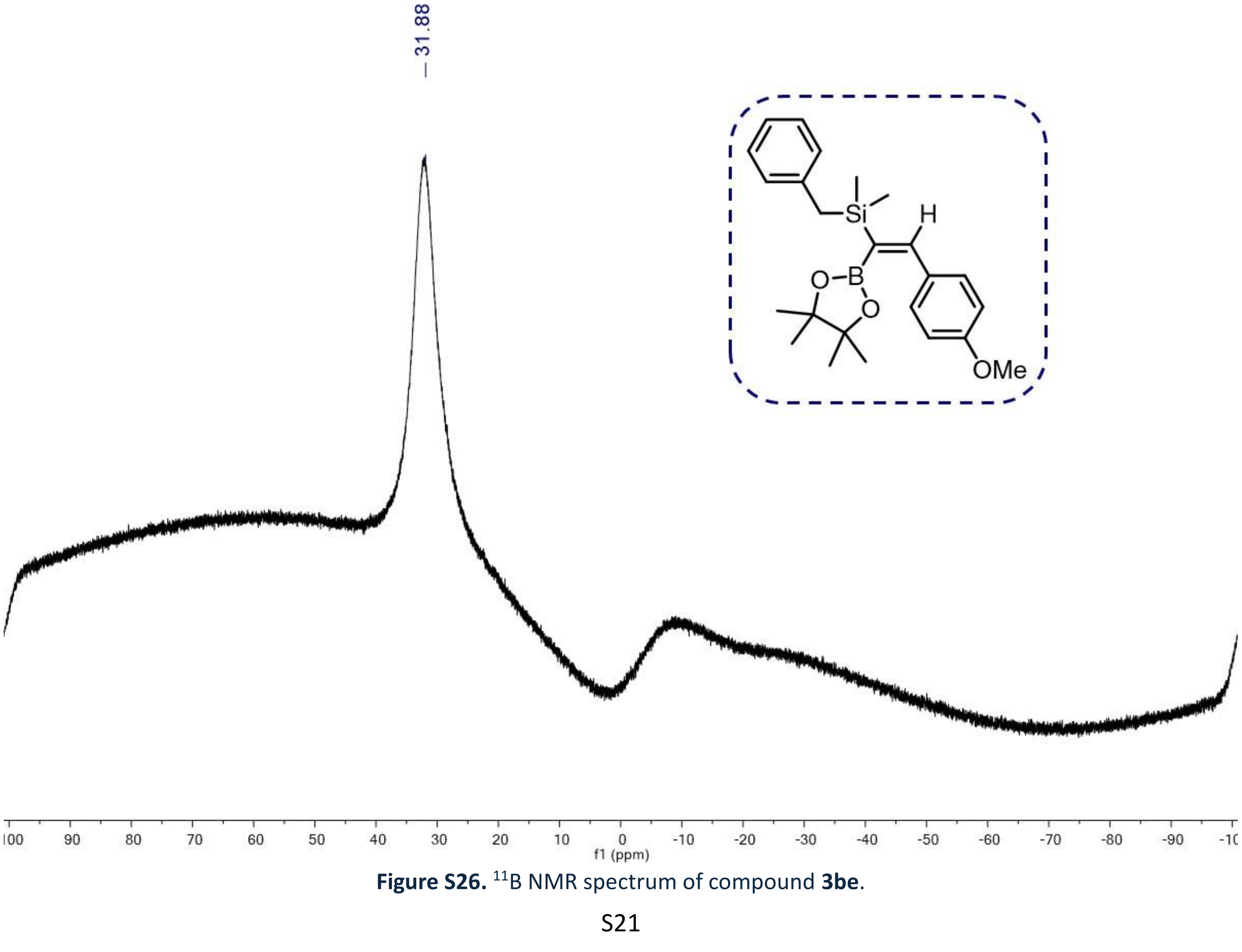

Figure S26. ^{11}B NMR spectrum of compound **3be**.

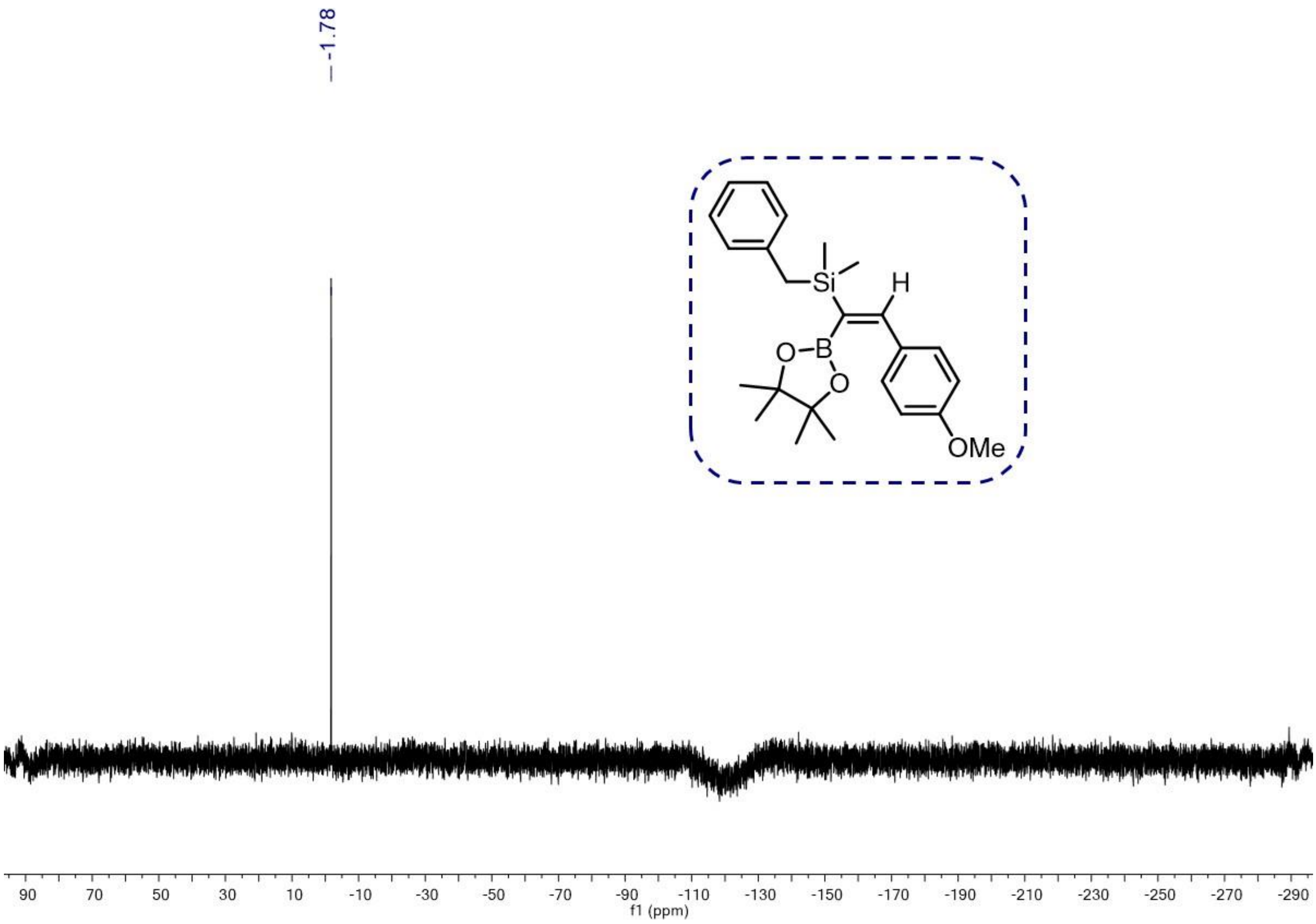

Figure S27. ^{29}Si NMR spectrum of compound **3be**.

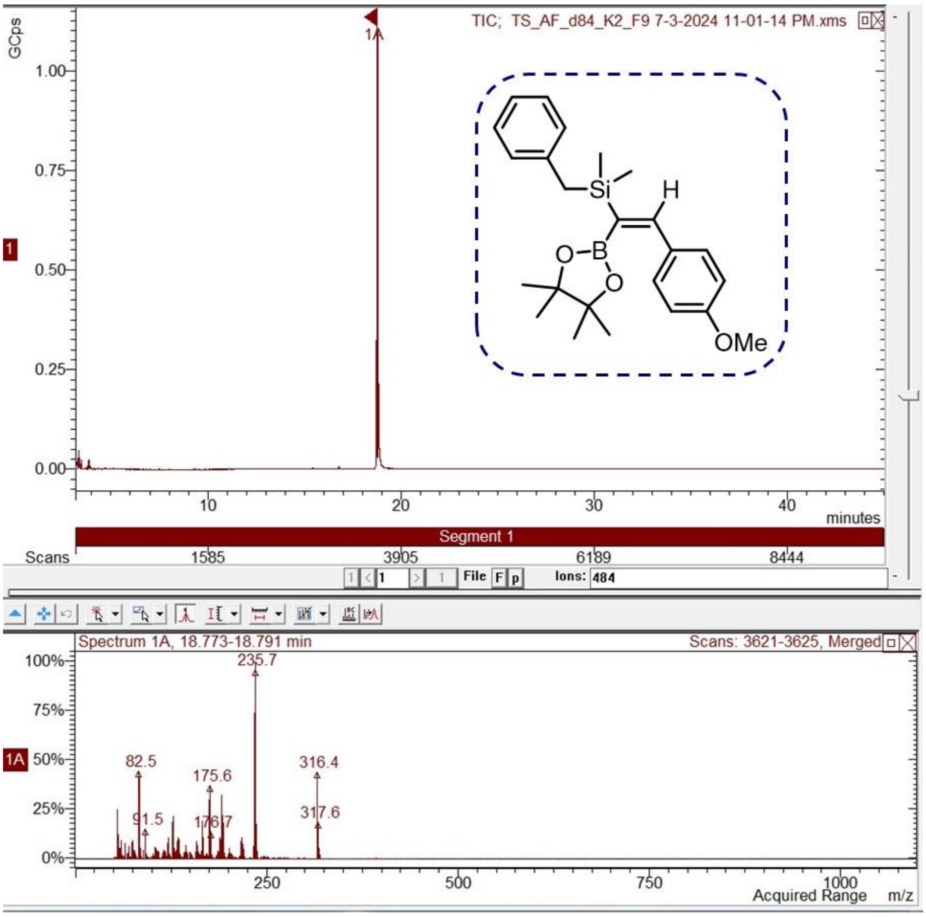

Figure S28. GC-MS image of compound **3be**.

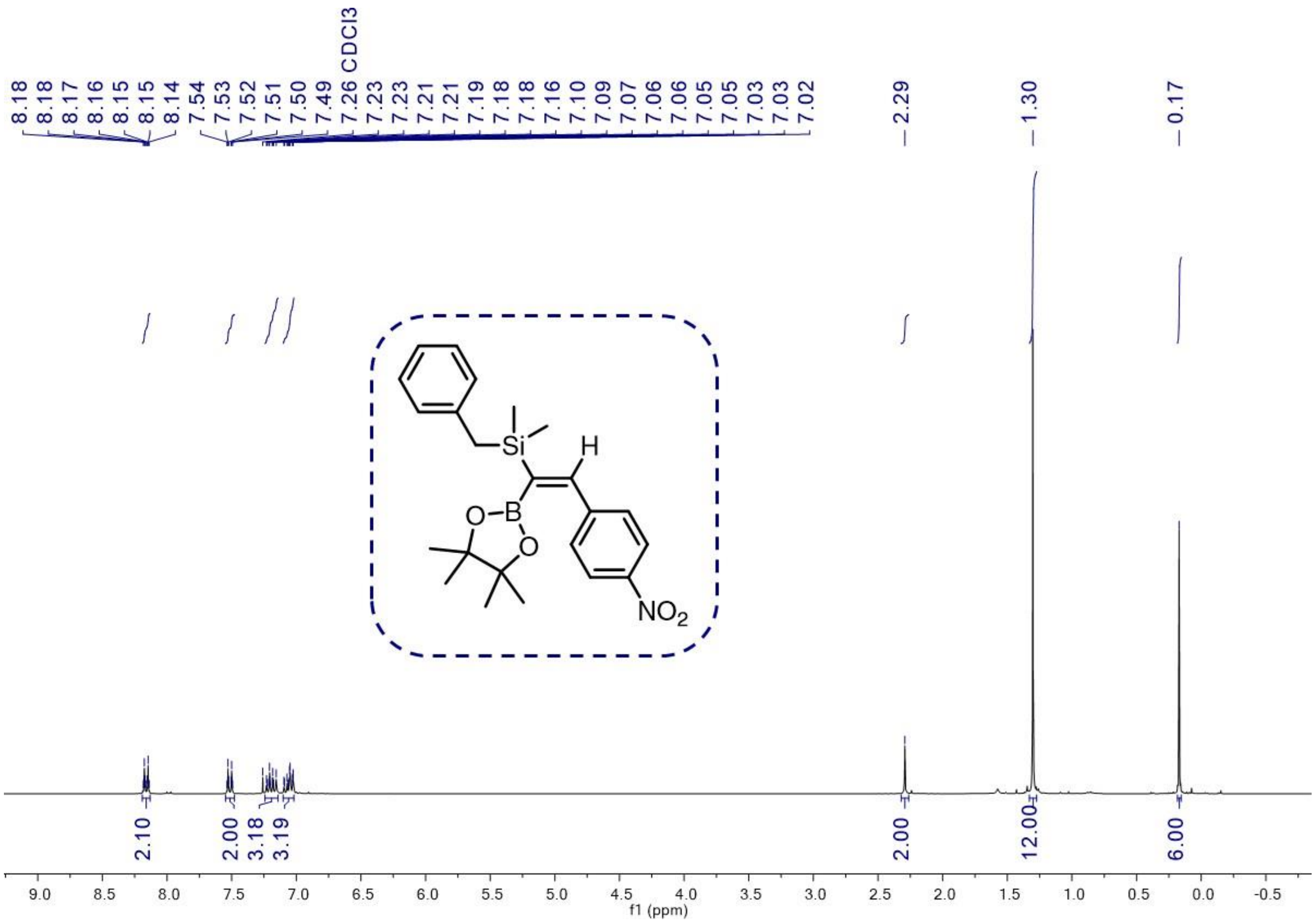

Figure S29. ^{1}H NMR spectrum of compound **3bf**.

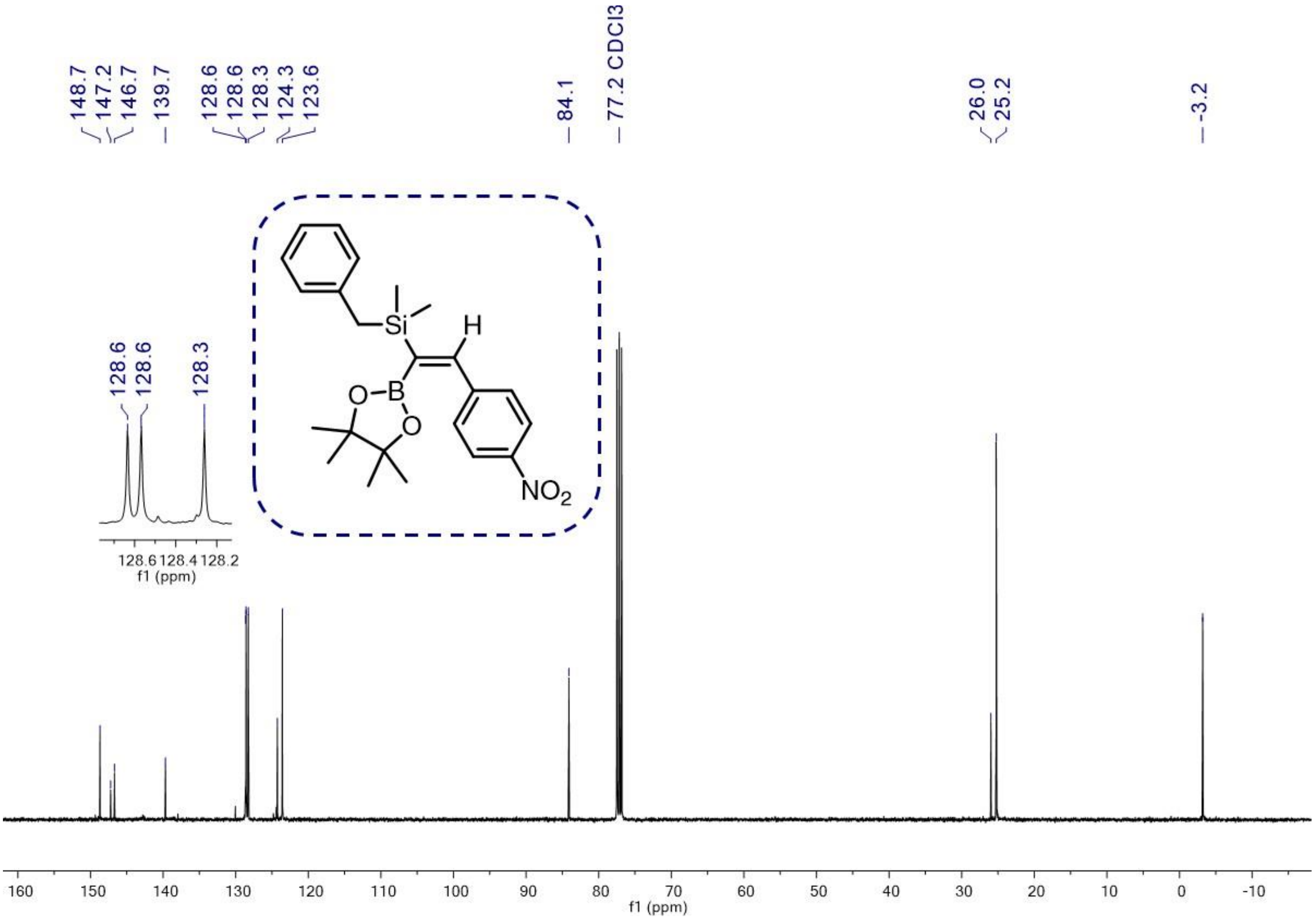

Figure S30. ^{13}C NMR spectrum of compound **3bf**.

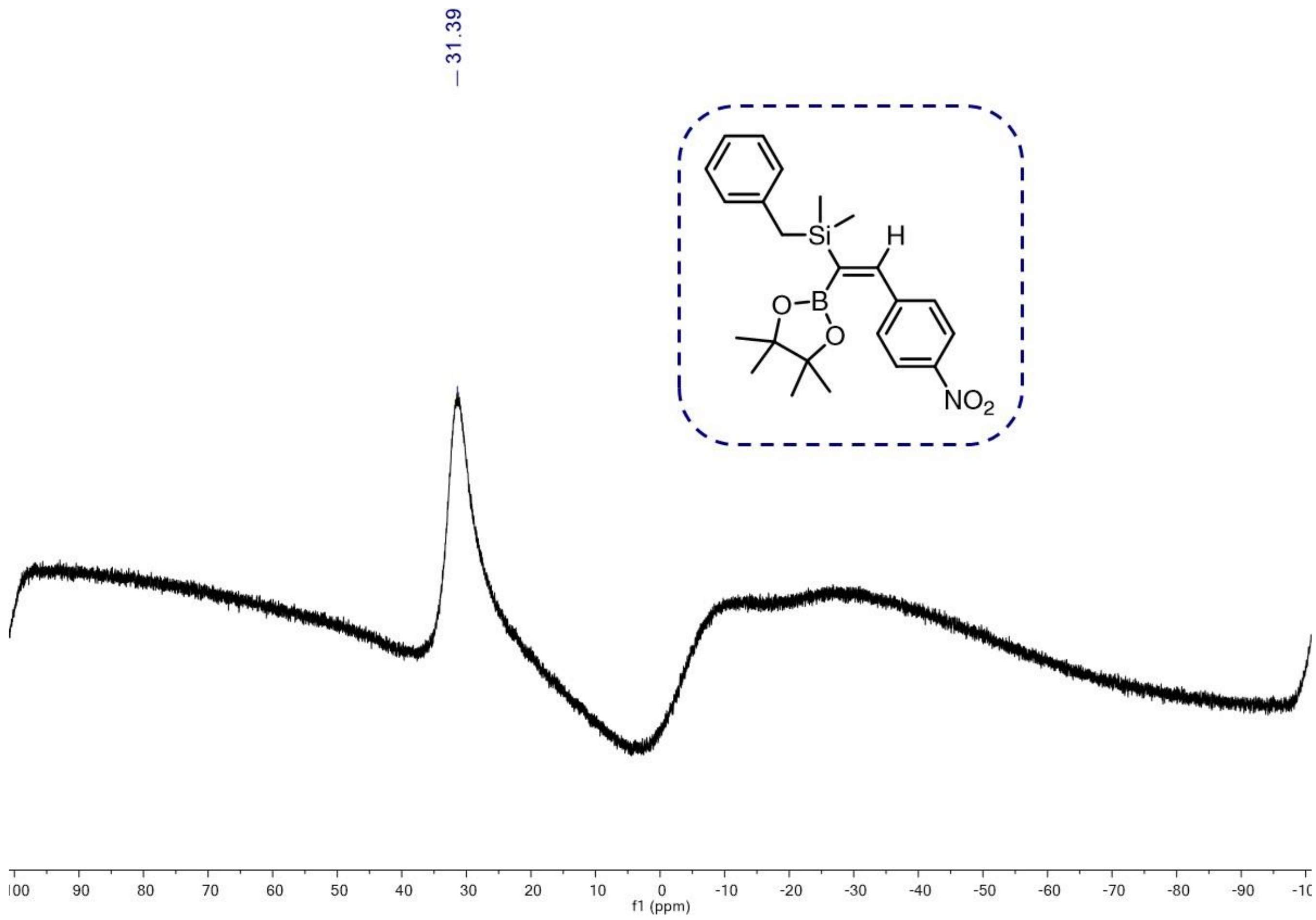

Figure S31. ^{11}B NMR spectrum of compound **3bf**.

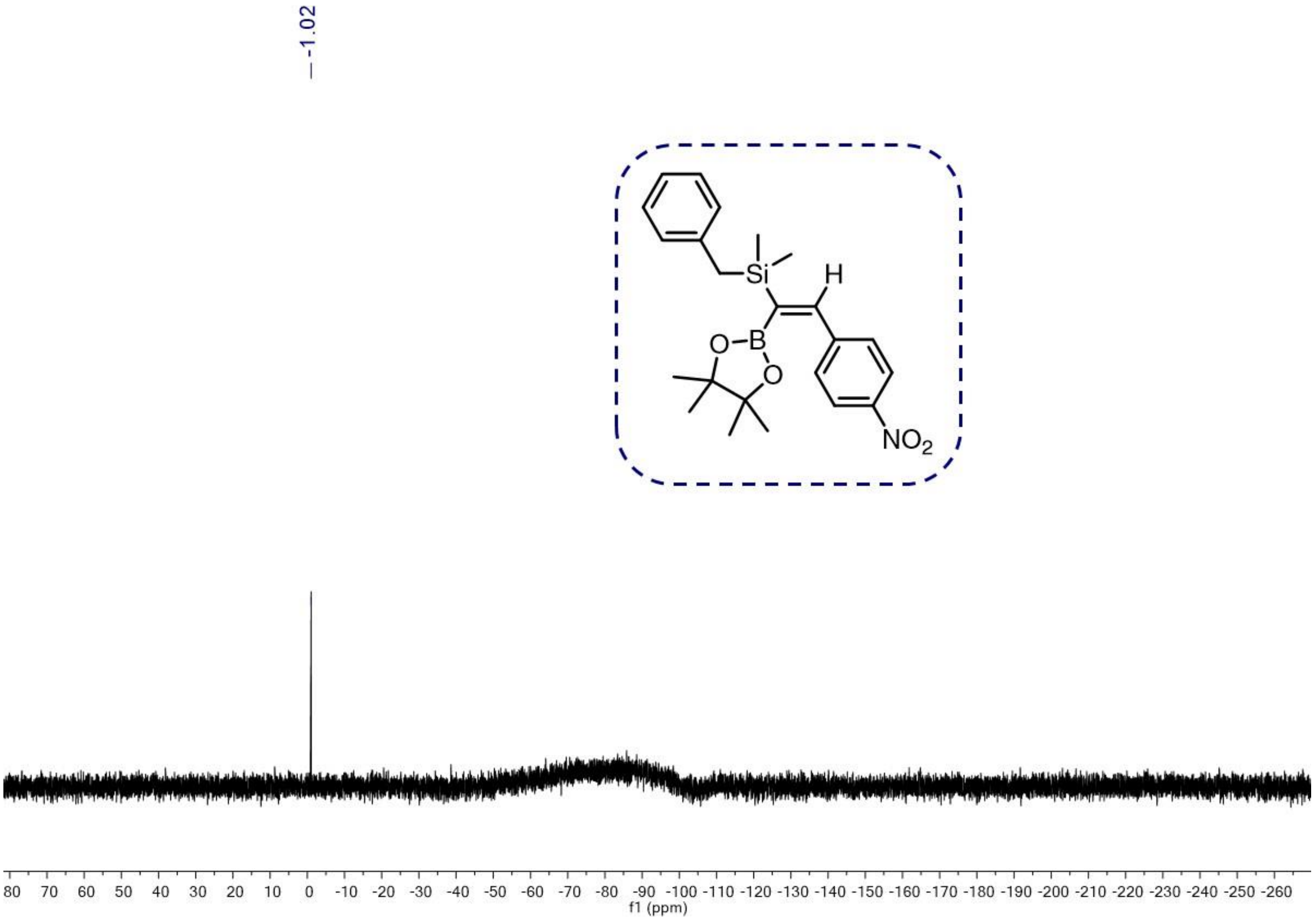

Figure S32. ^{29}Si NMR spectrum of compound **3bf**.

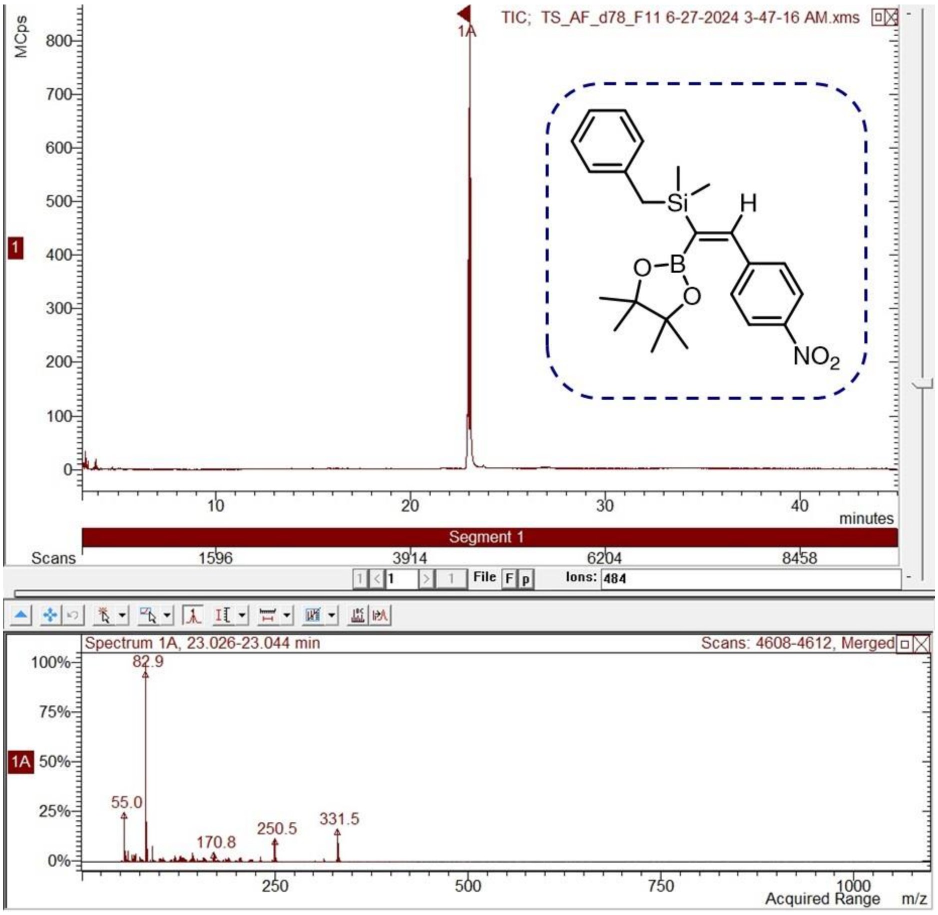

Figure S33. GC-MS image of compound **3bf**.

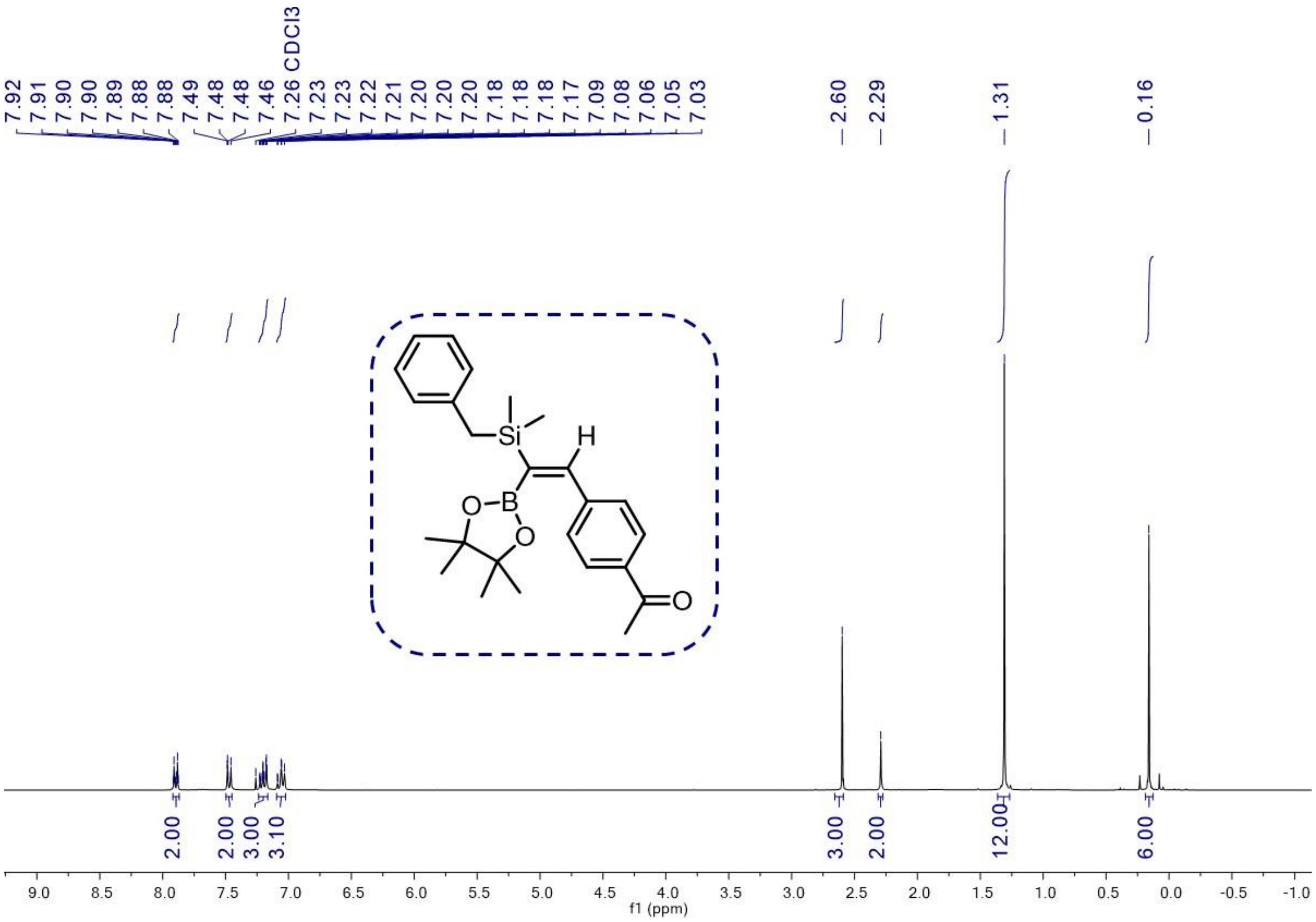

Figure S34. ^{1}H NMR spectrum of compound **3bg**.

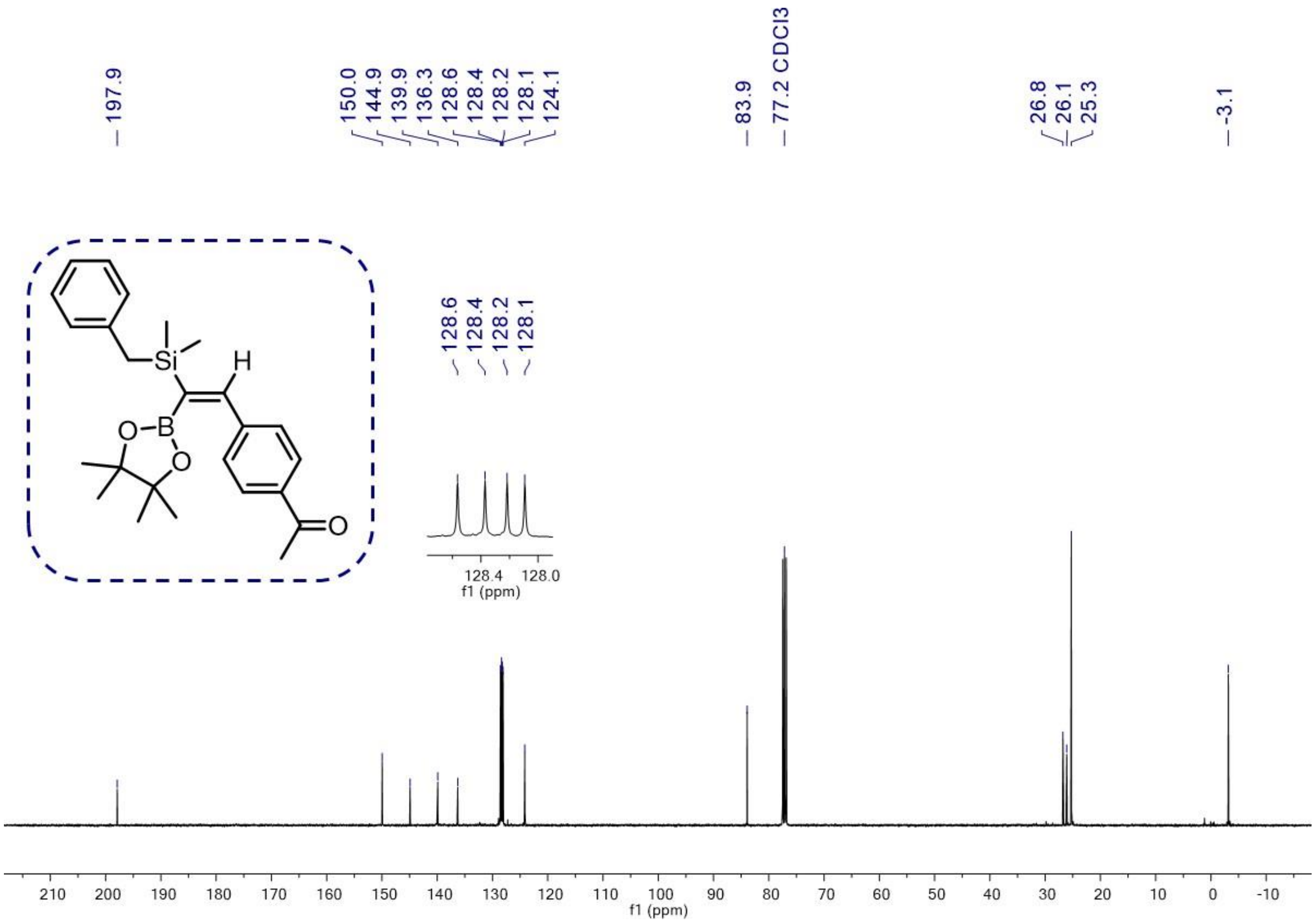

Figure S35. ^{13}C NMR spectrum of compound **3bg**.

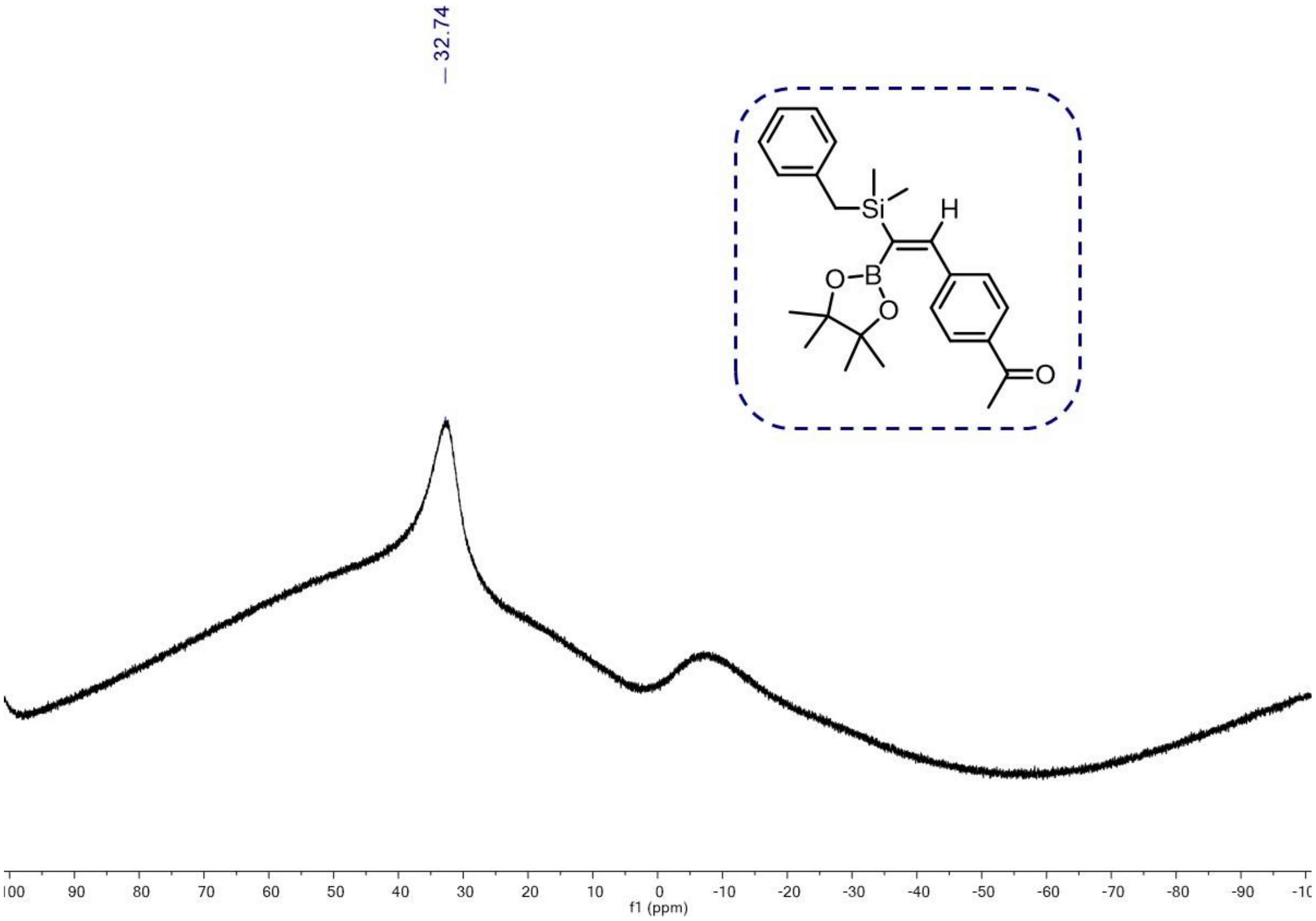

Figure S36. ^{11}B NMR spectrum of compound **3bg**.

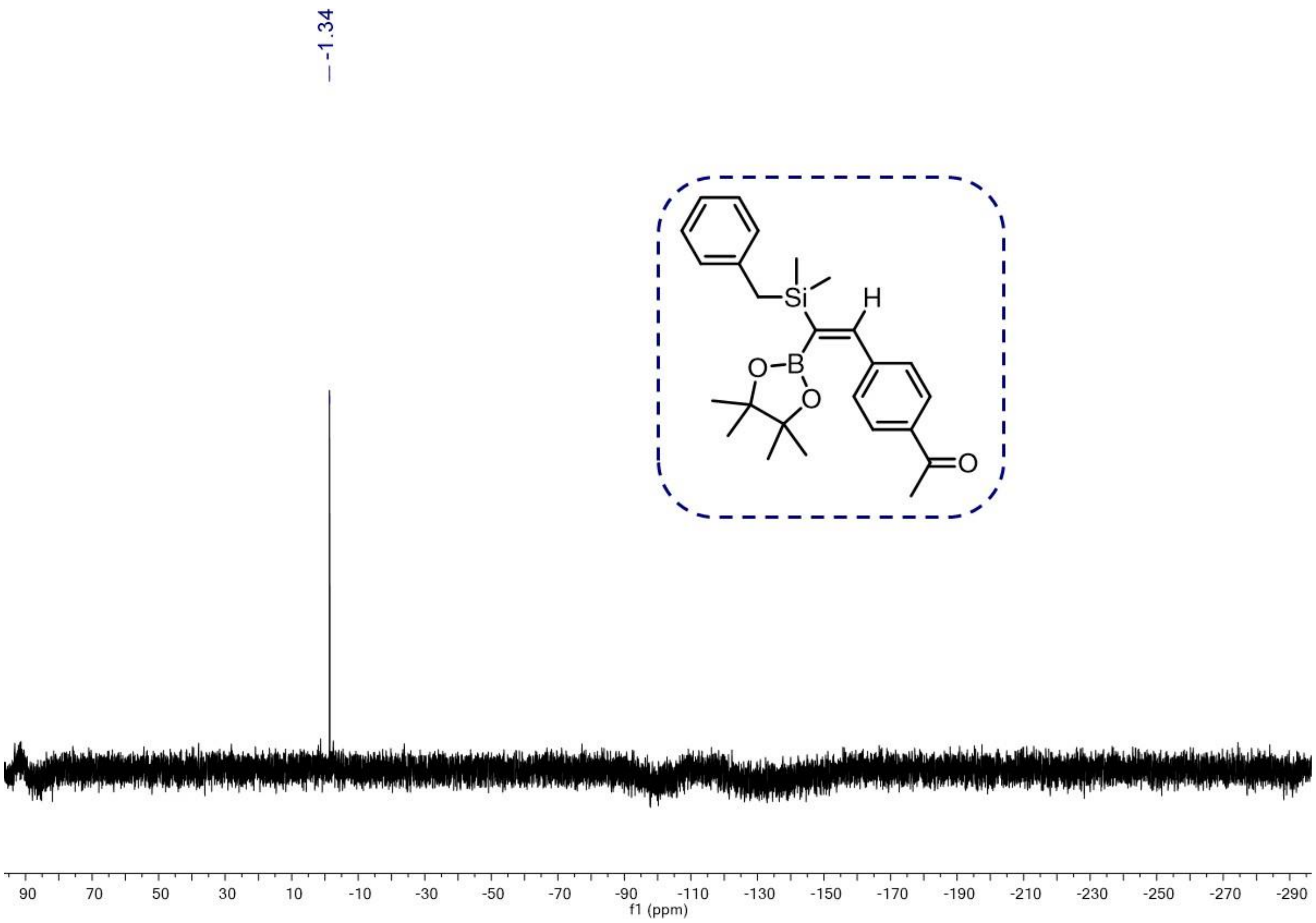

Figure S37. ^{29}Si NMR spectrum of compound **3bg**.

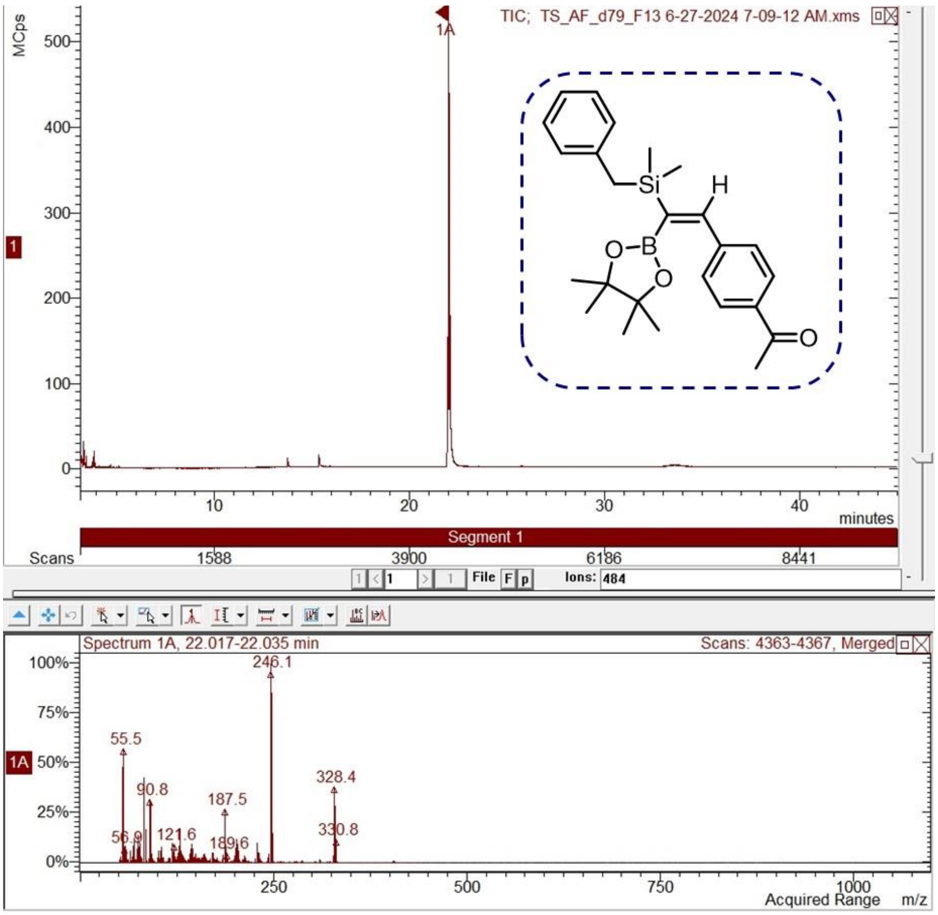

Figure S38. GC-MS image of compound **3bg**.

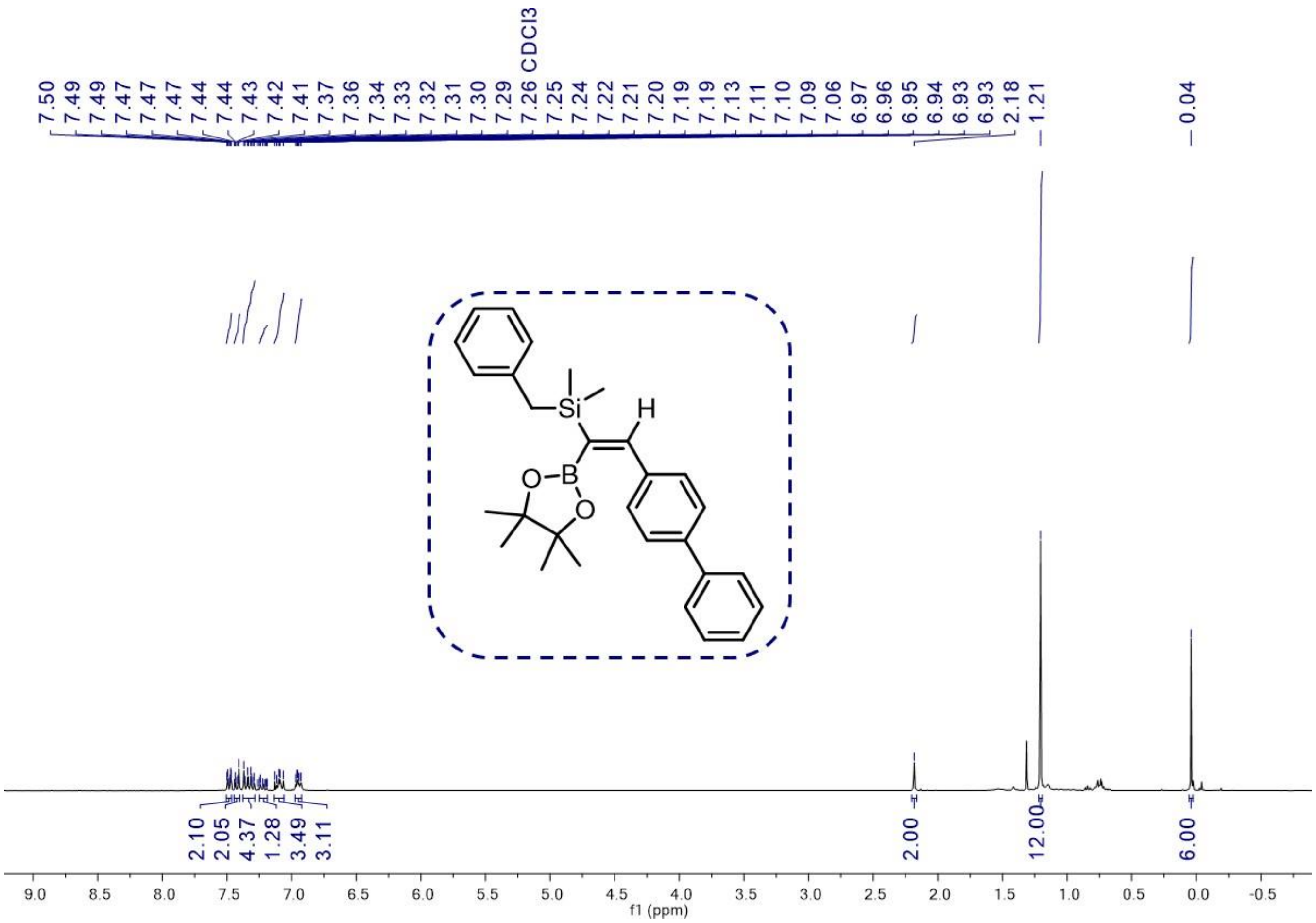

Figure S39. ^{1}H NMR spectrum of compound **3bh**.

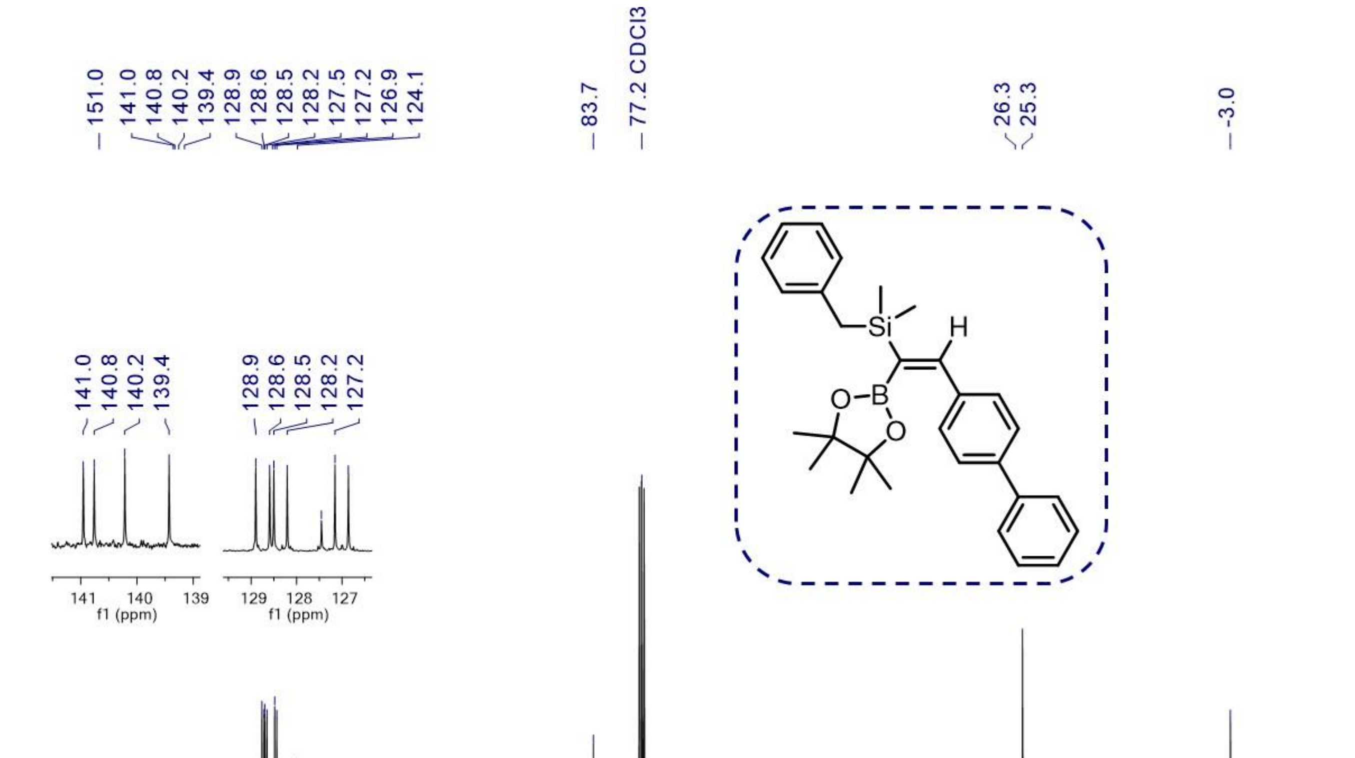

Figure S40. ^{13}C NMR spectrum of compound **3bh**.

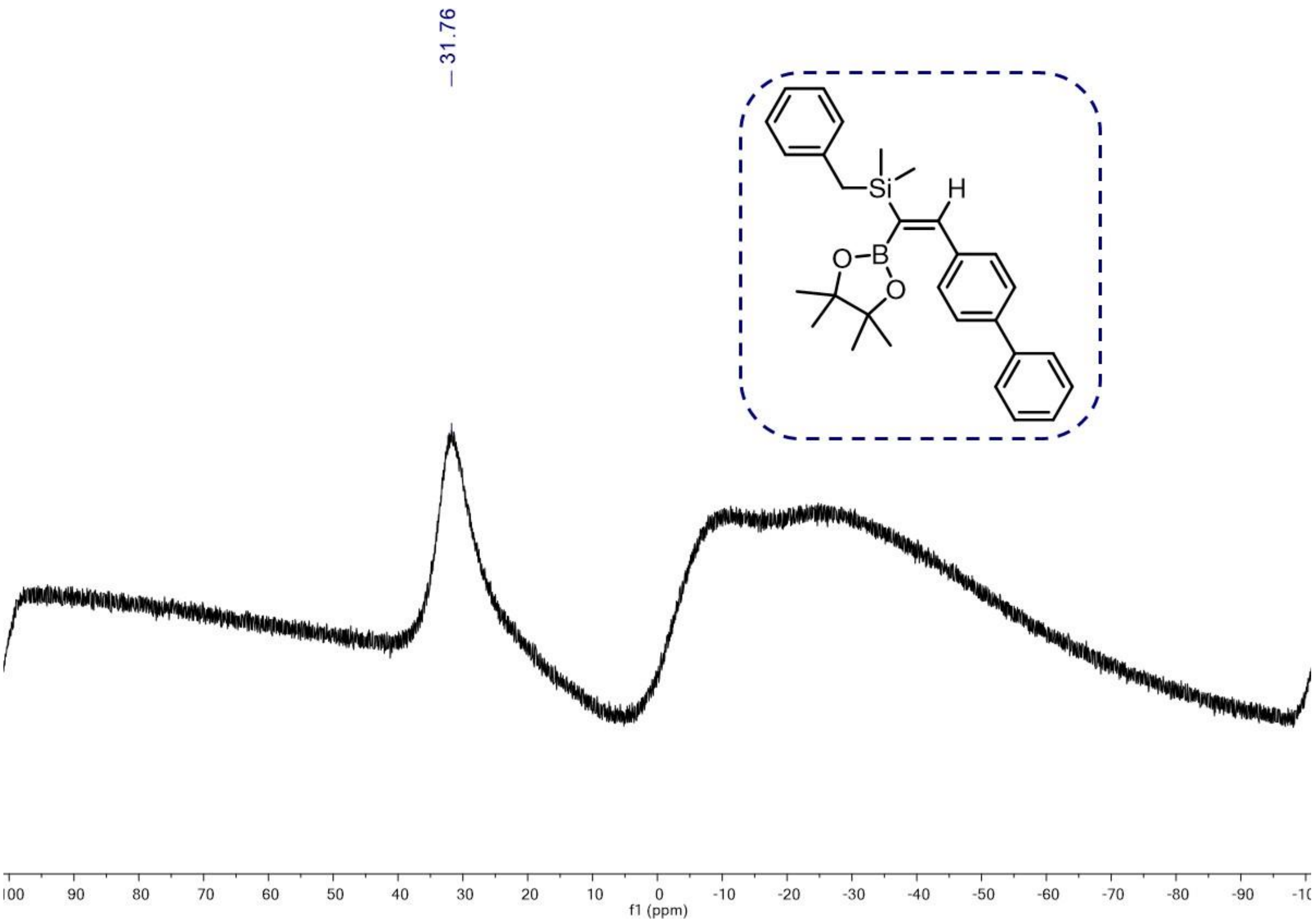

Figure S41. ^{11}B NMR spectrum of compound **3bh**.

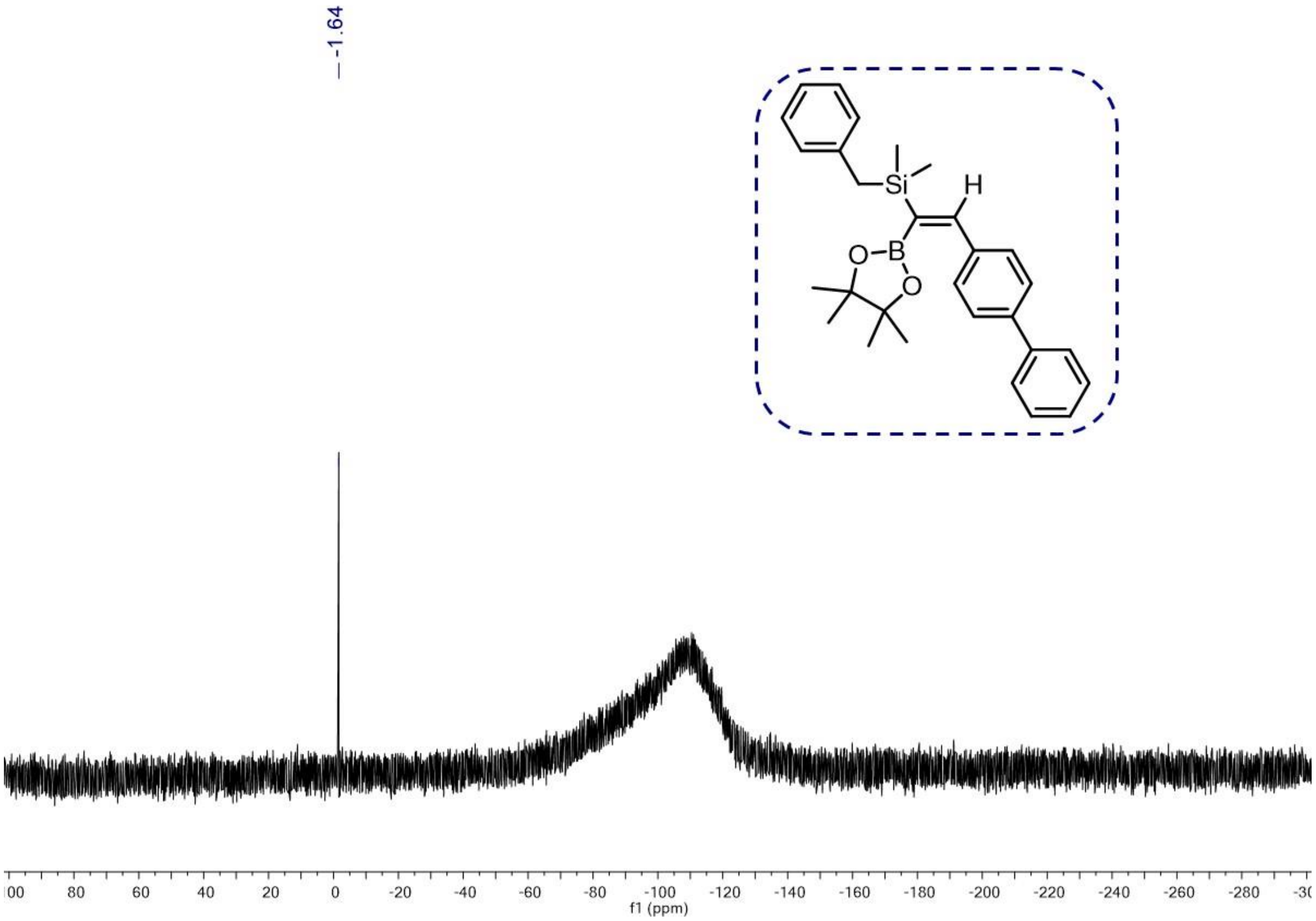

Figure S42. ^{29}Si NMR spectrum of compound **3bh**.

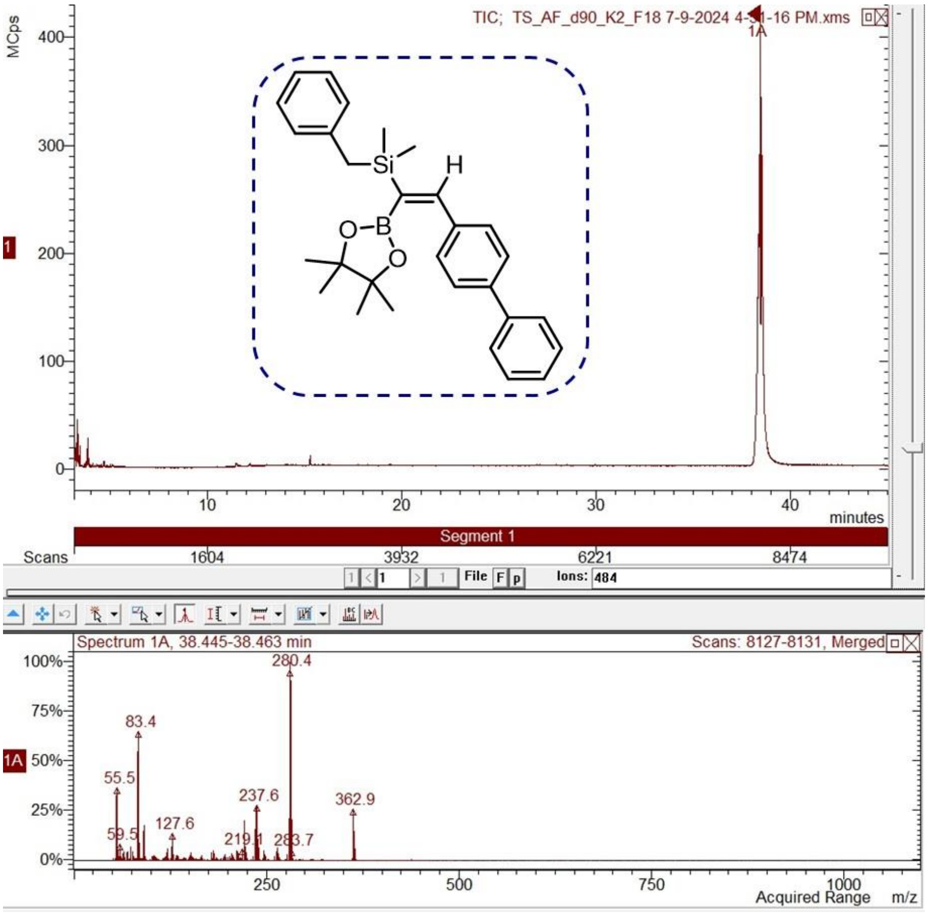

Figure S43. GC-MS image of compound **3bh**.

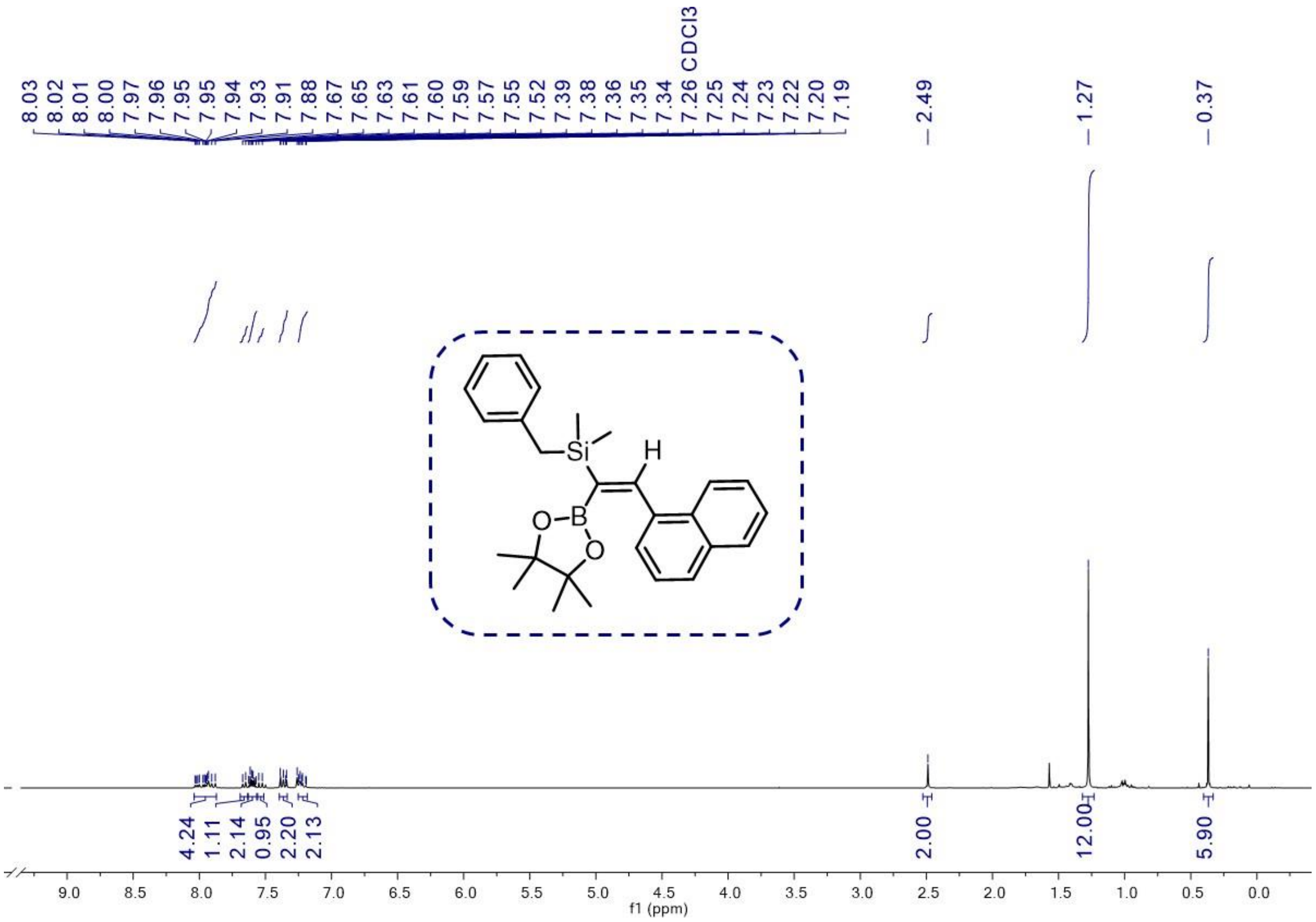

Figure S44. ^{1}H NMR spectrum of compound **3bi**.

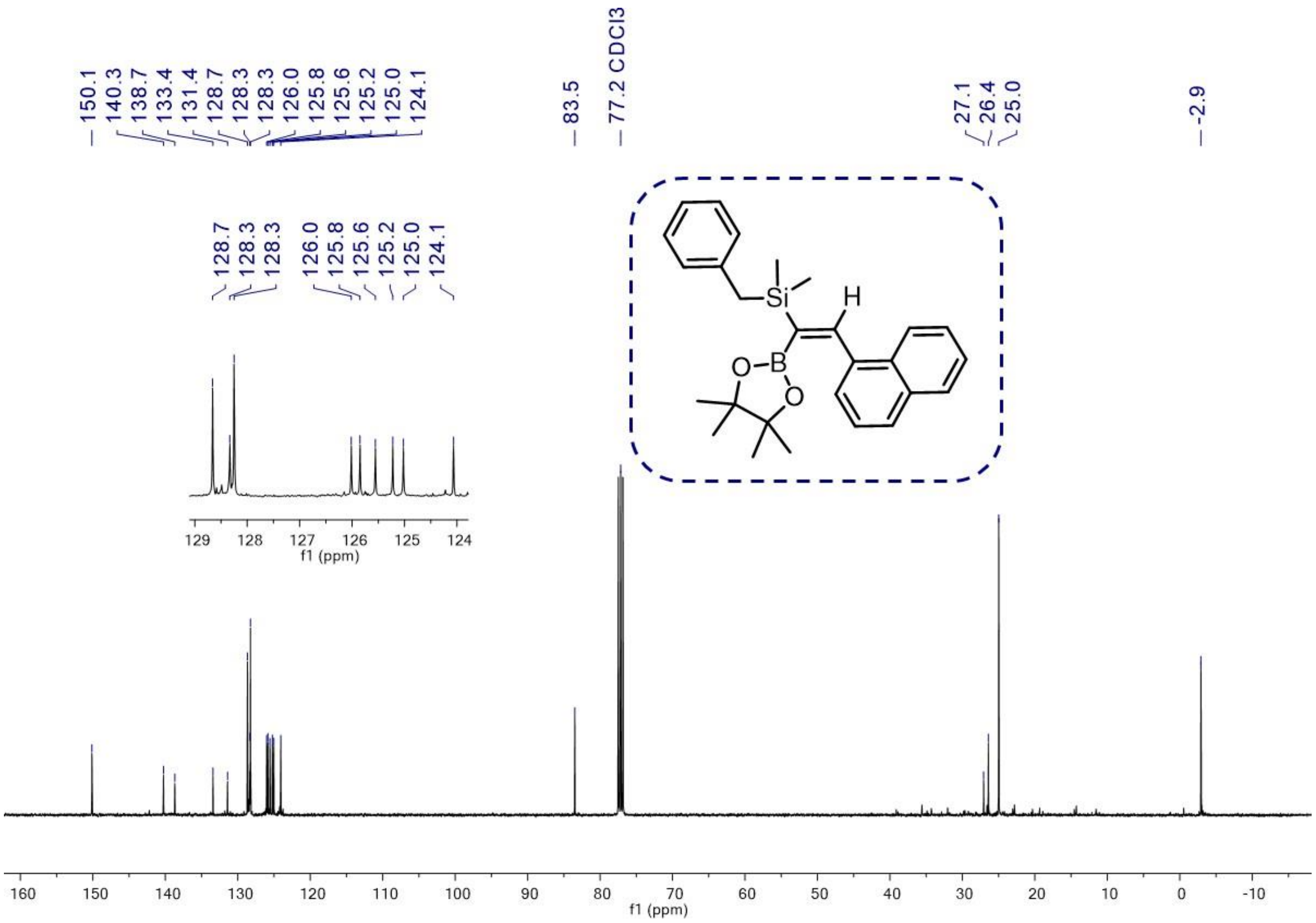

Figure S45. ^{13}C NMR spectrum of compound **3bi**.

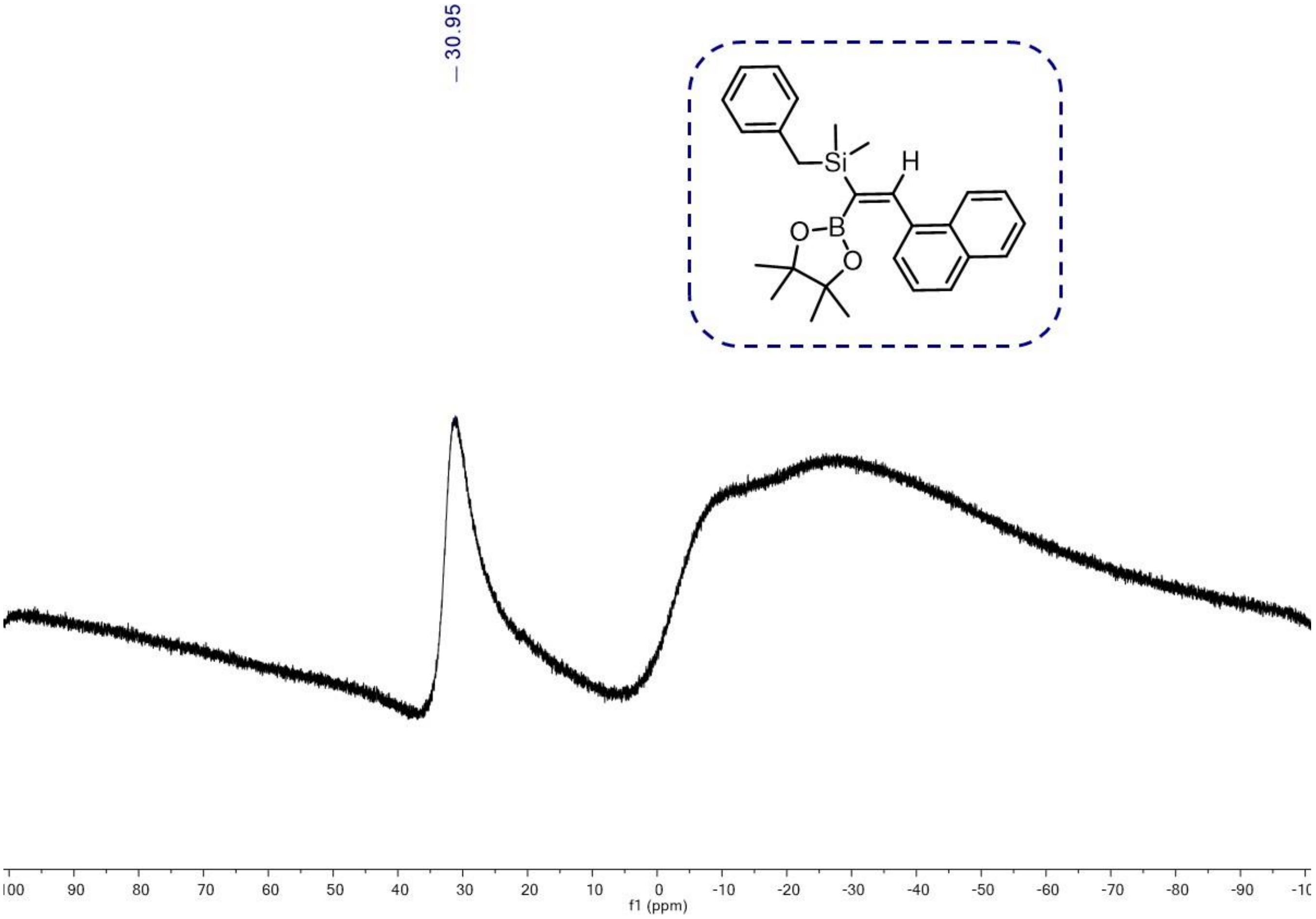

Figure S46. ^{11}B NMR spectrum of compound **3bi**.

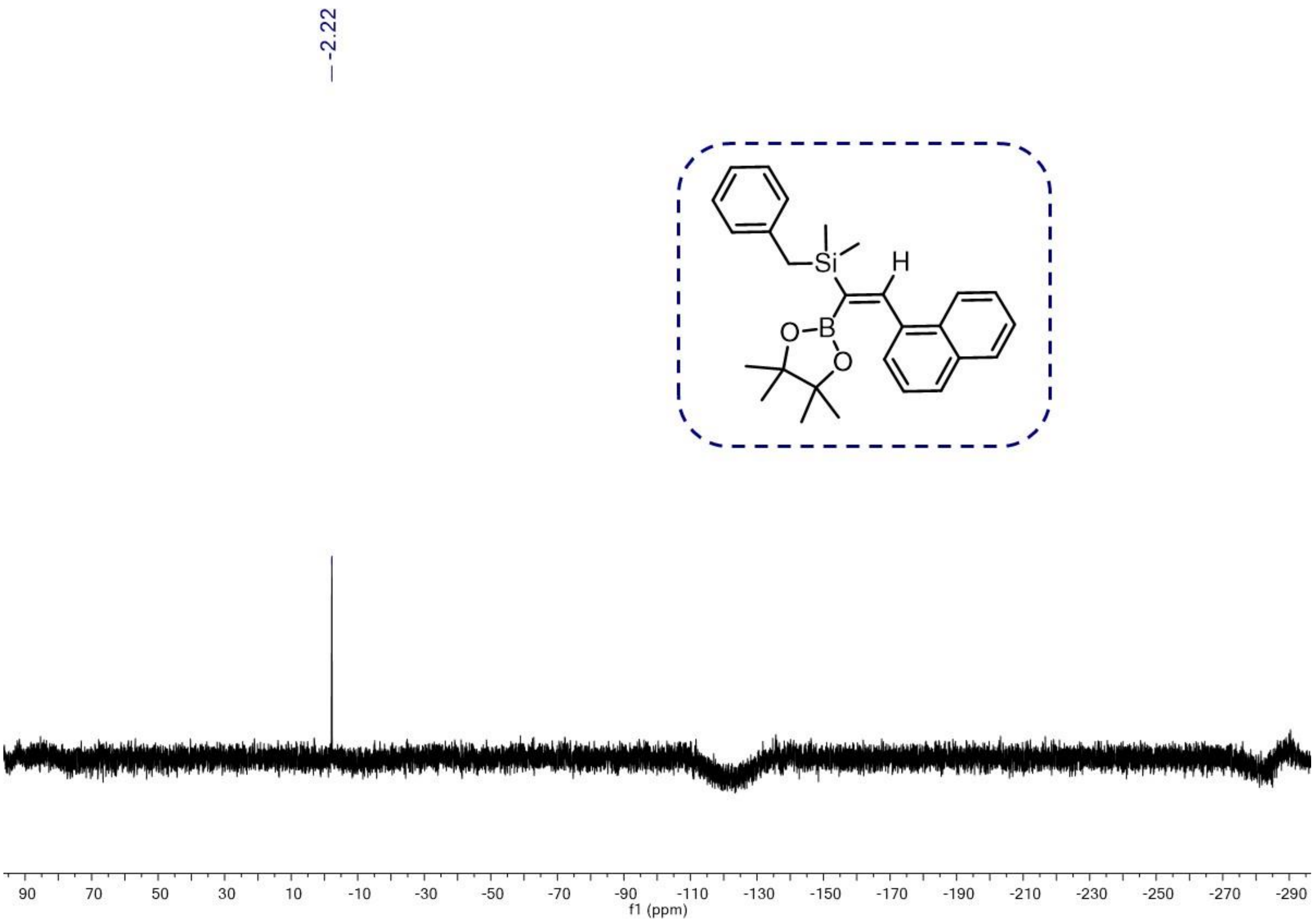

Figure S47. ^{29}Si NMR spectrum of compound **3bi**.

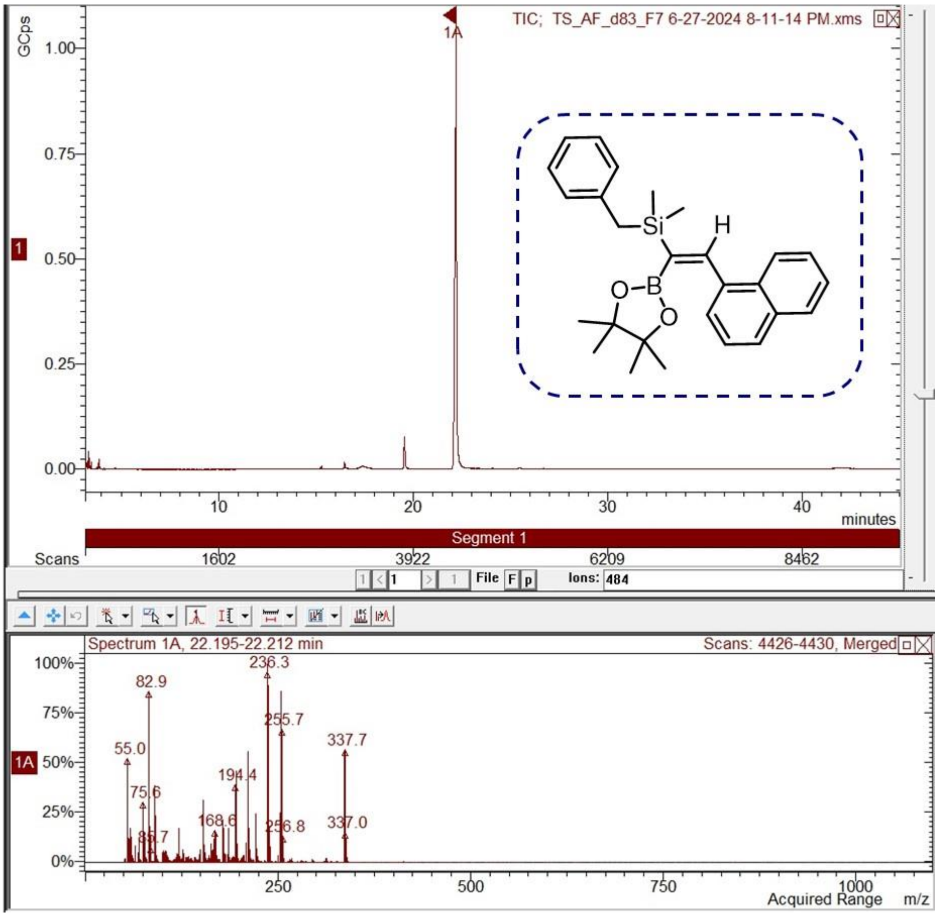

Figure S48. GC-MS image of compound **3bi**.

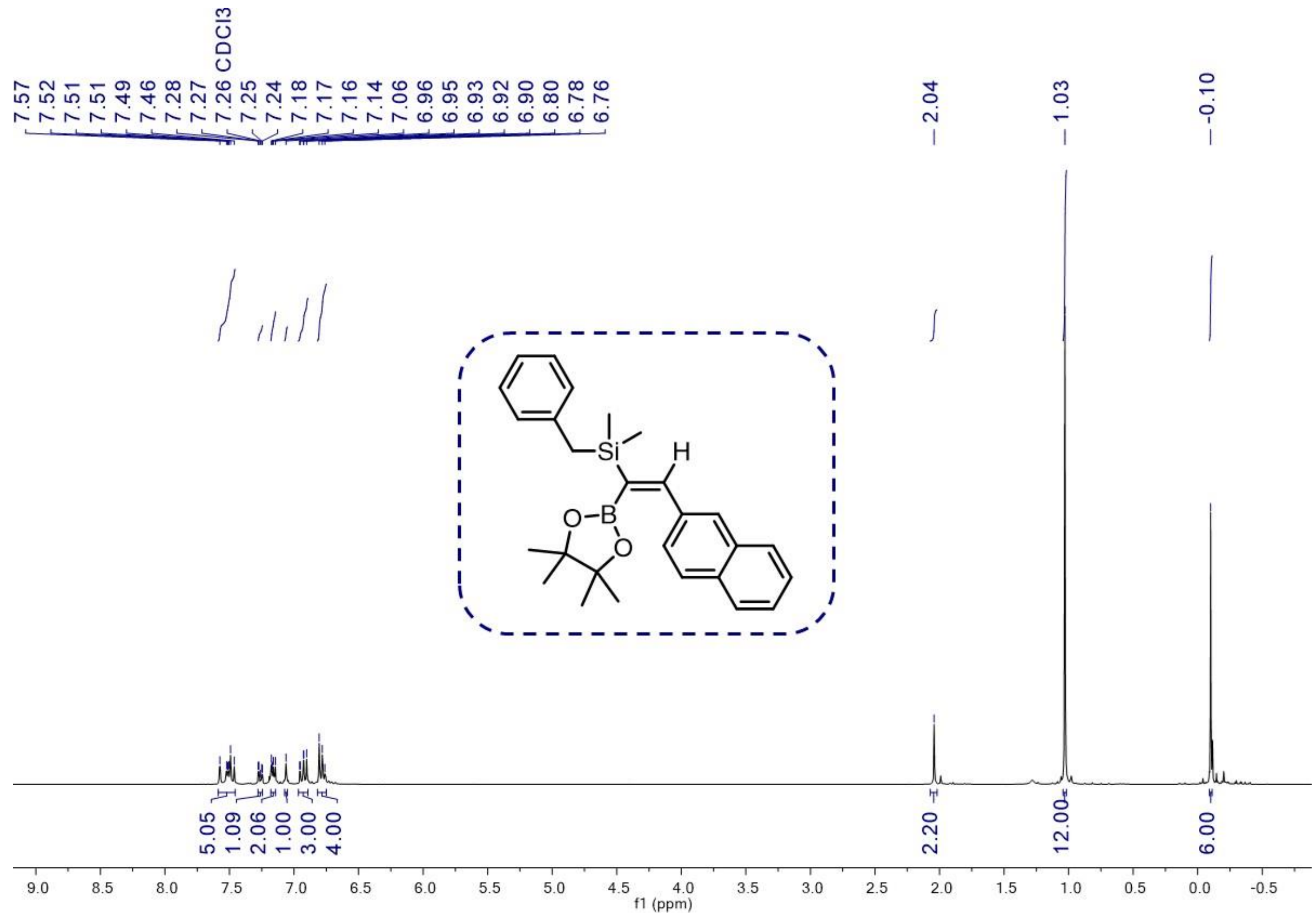

Figure S49. ^{1}H NMR spectrum of compound **3bj**.

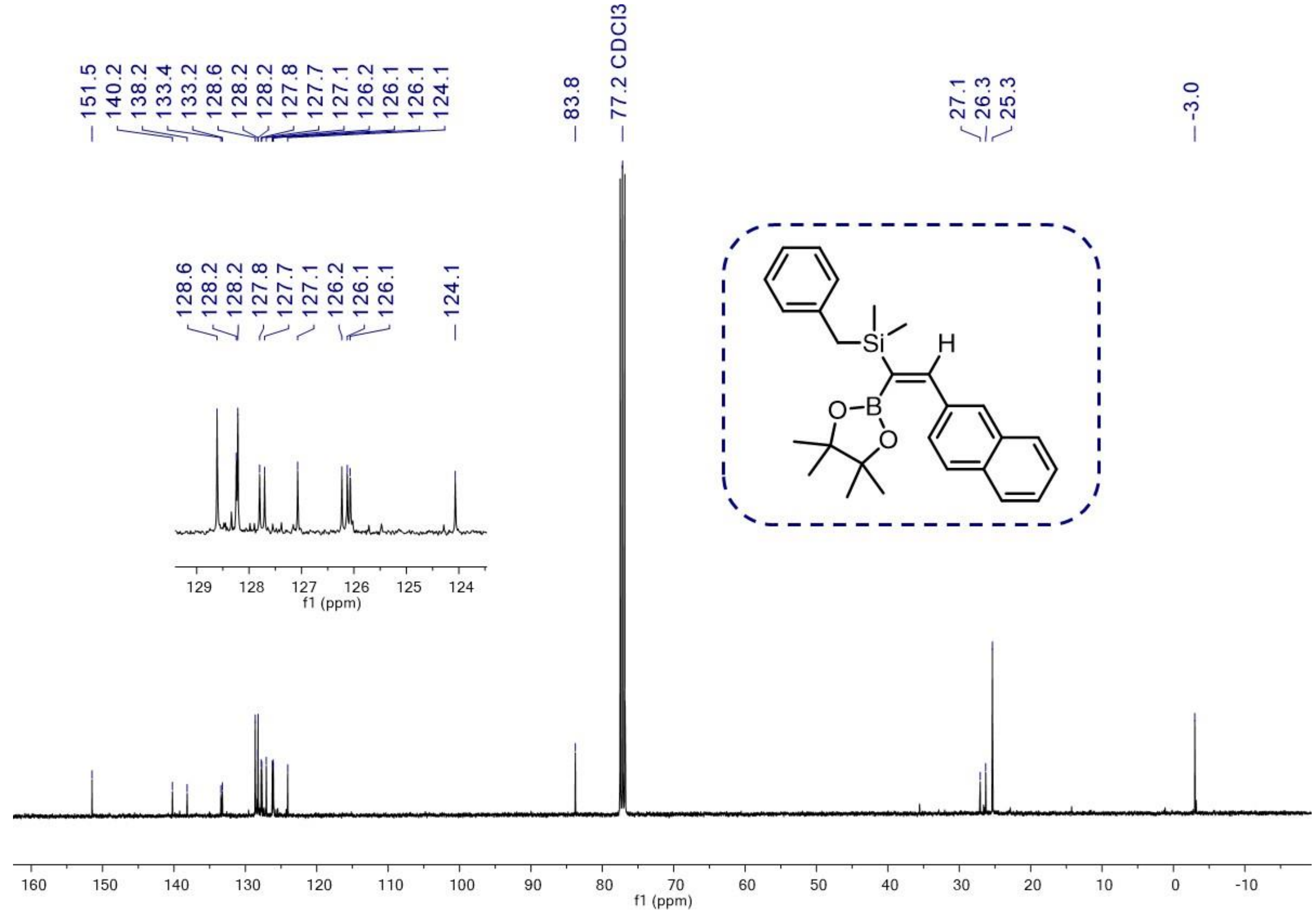

Figure S50. ^{13}C NMR spectrum of compound **3bj**.

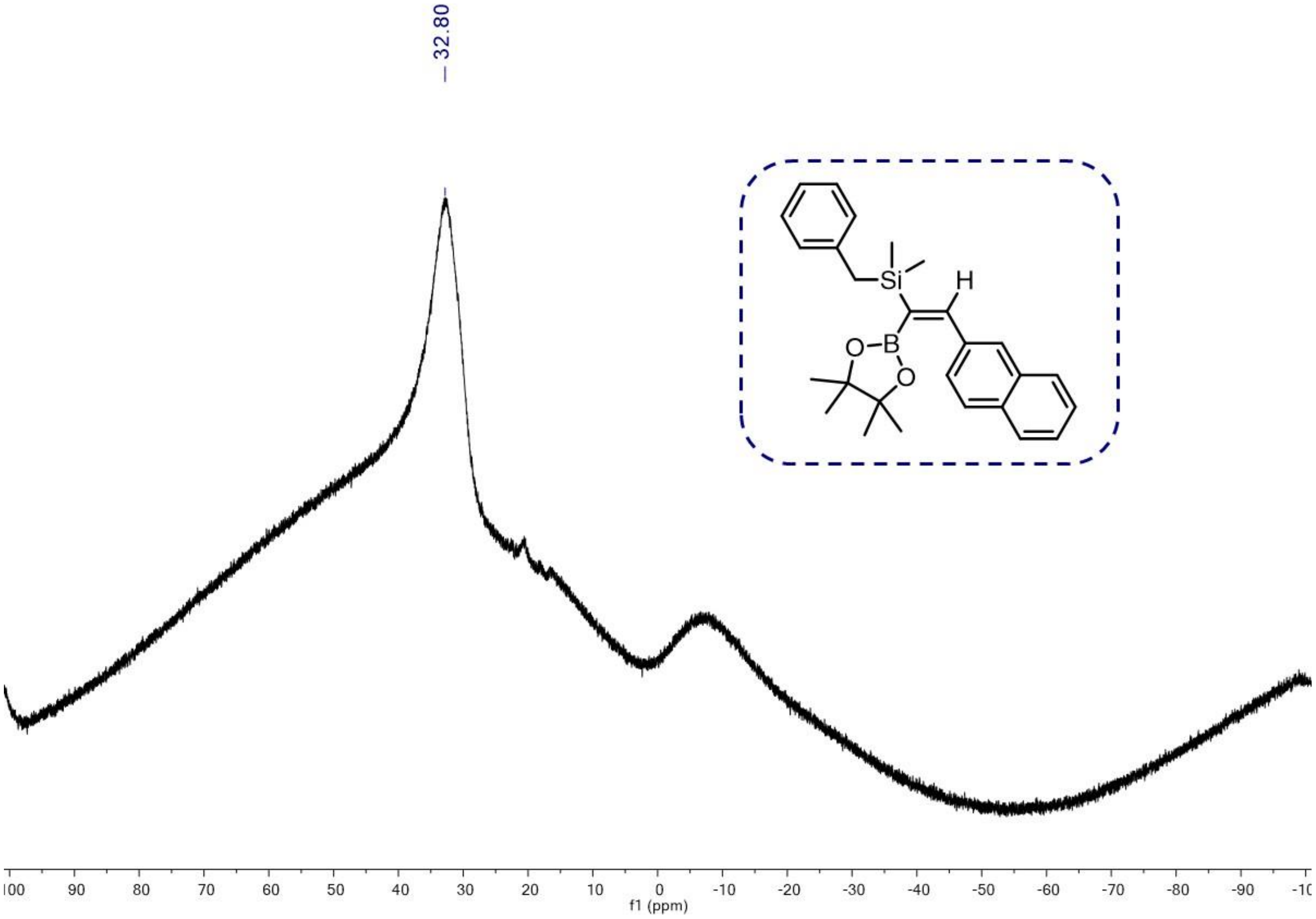

Figure S51. ^{11}B NMR spectrum of compound **3bj**.

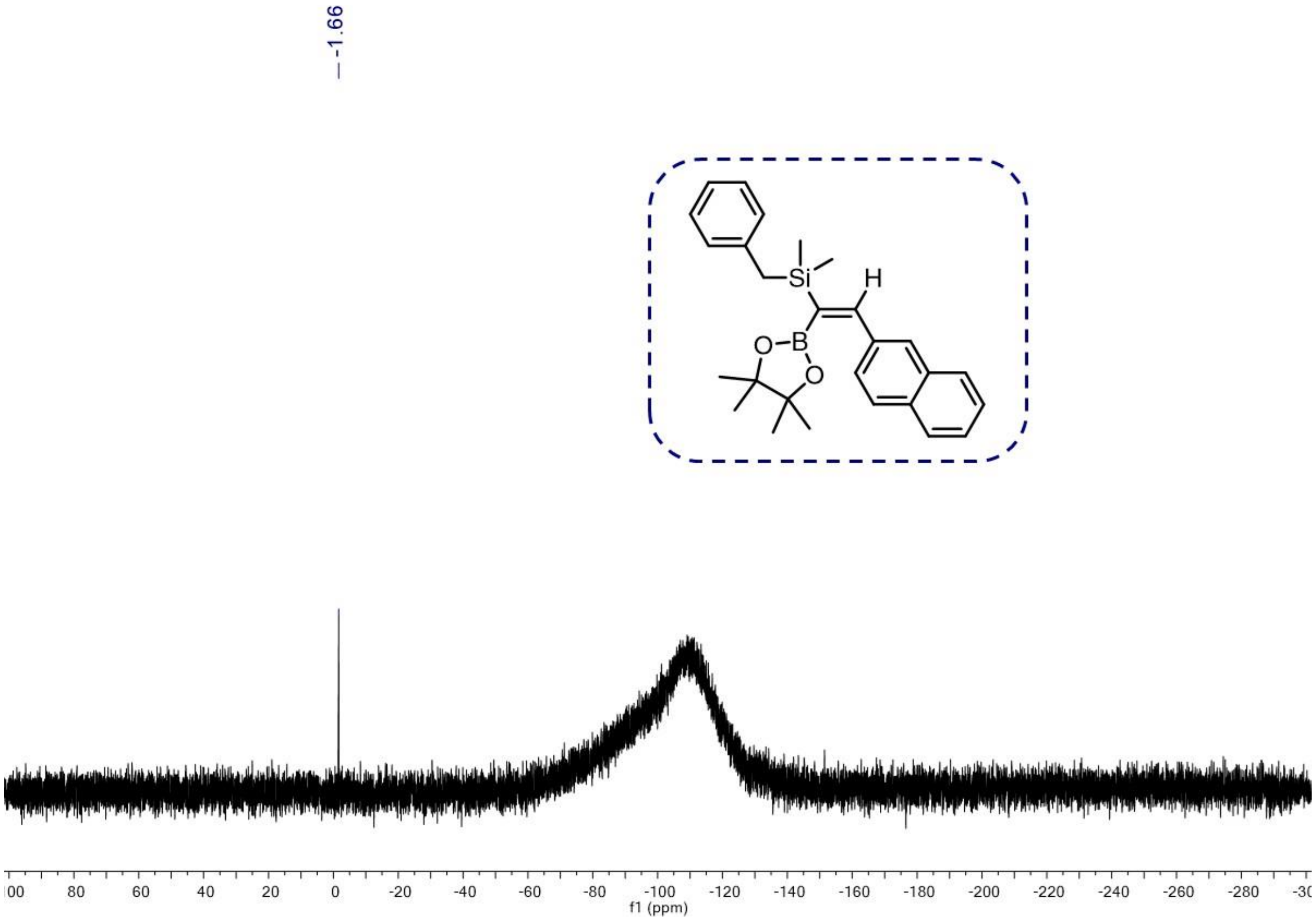

Figure S52. ^{29}Si NMR spectrum of compound **3bj**.

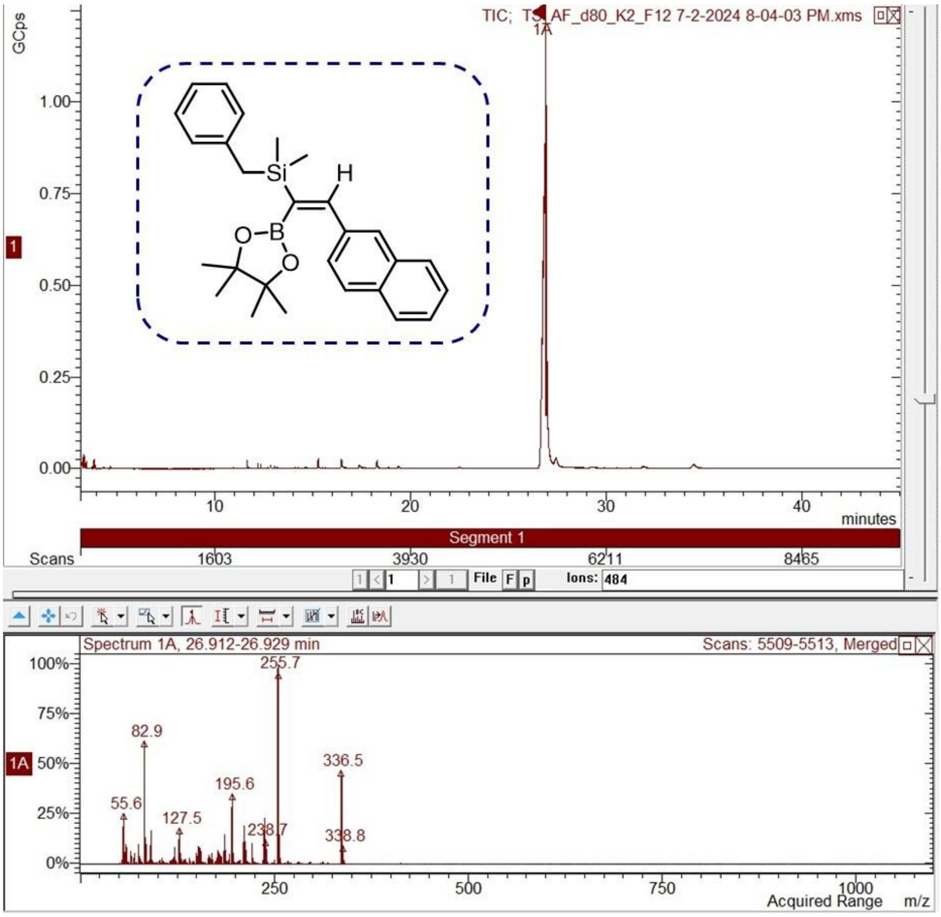

Figure S53. GC-MS image of compound **3bj**.

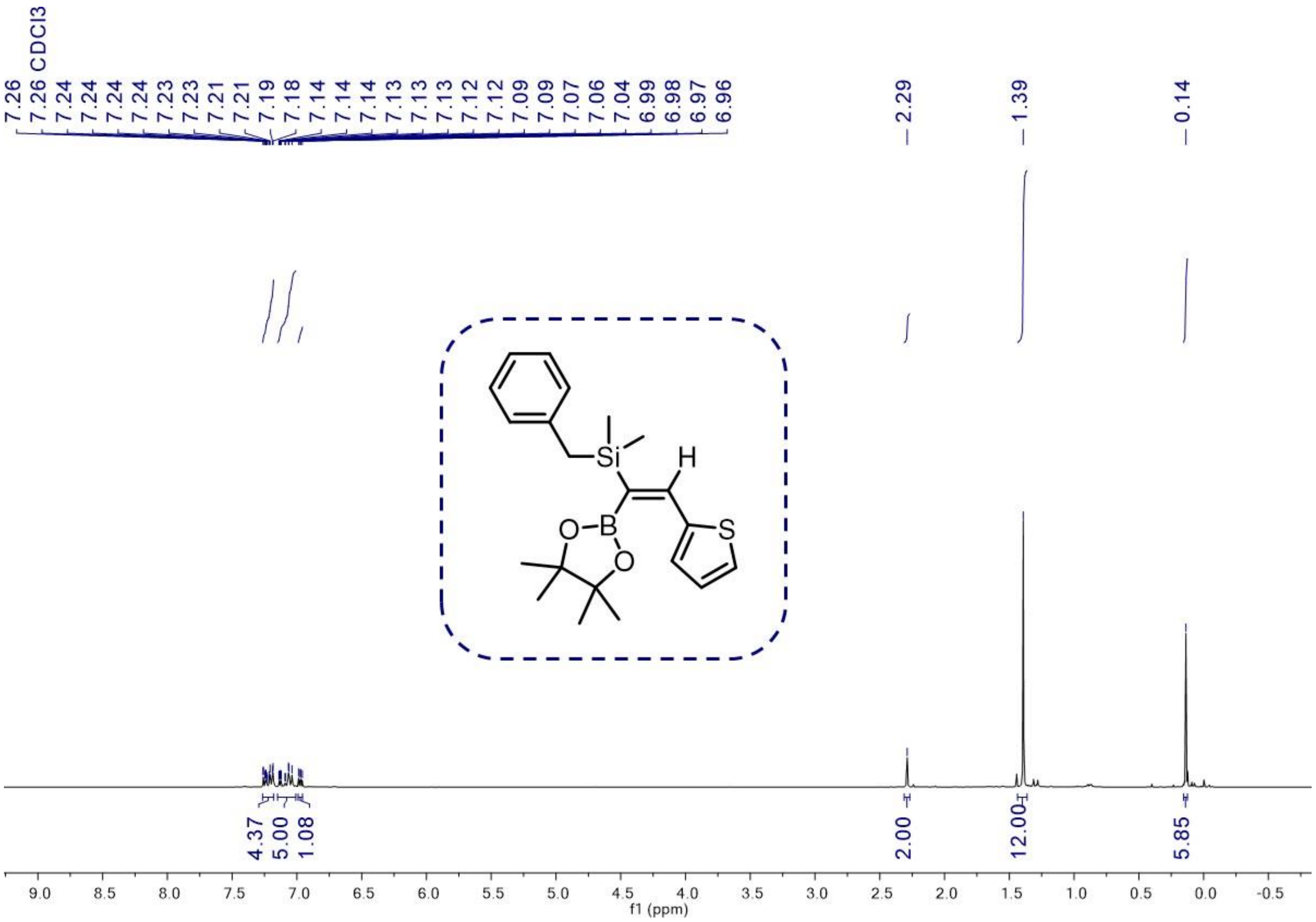

Figure S54. ^{1}H NMR spectrum of compound **3bk**.

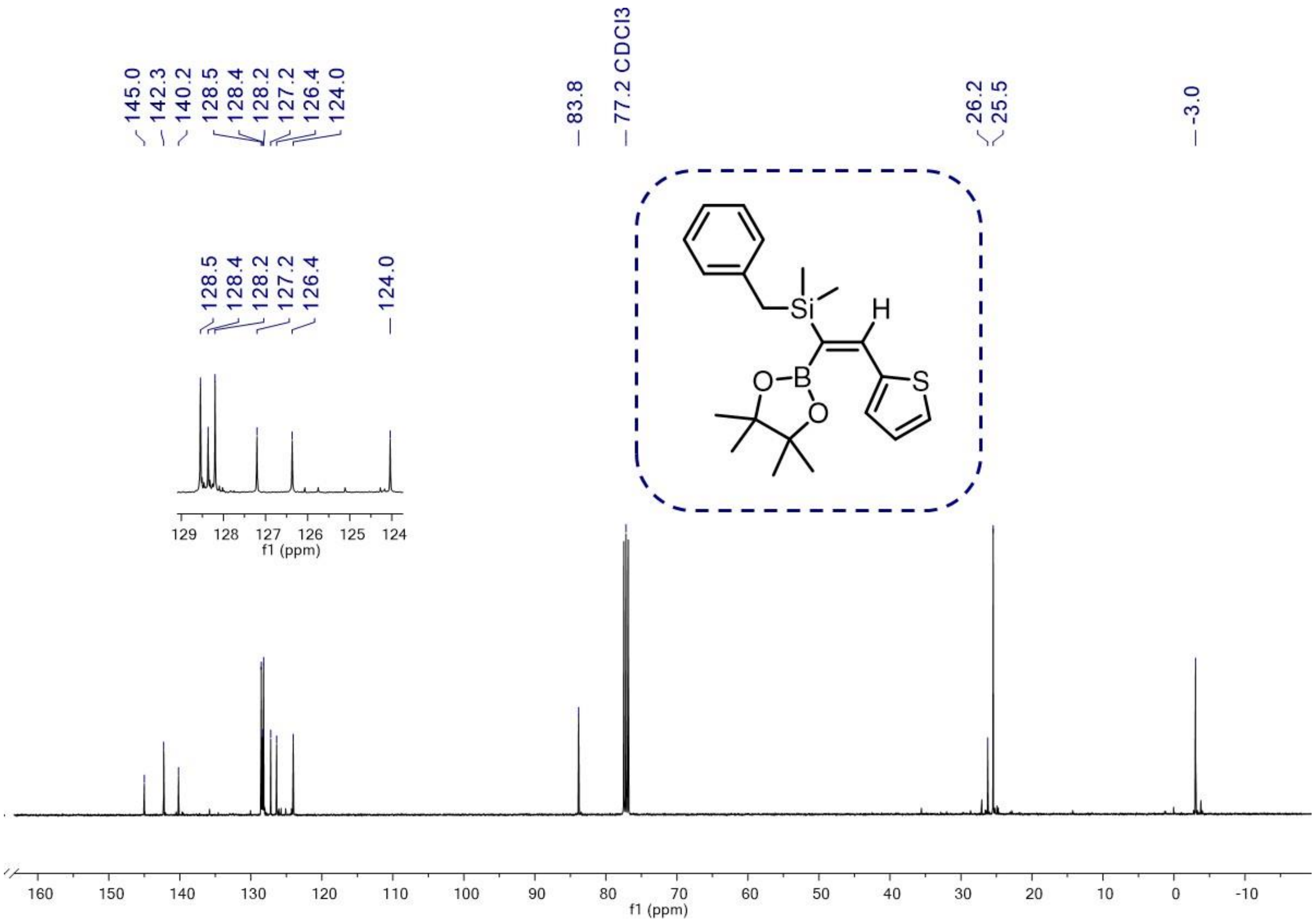

Figure S55. ^{13}C NMR spectrum of compound **3bk**.

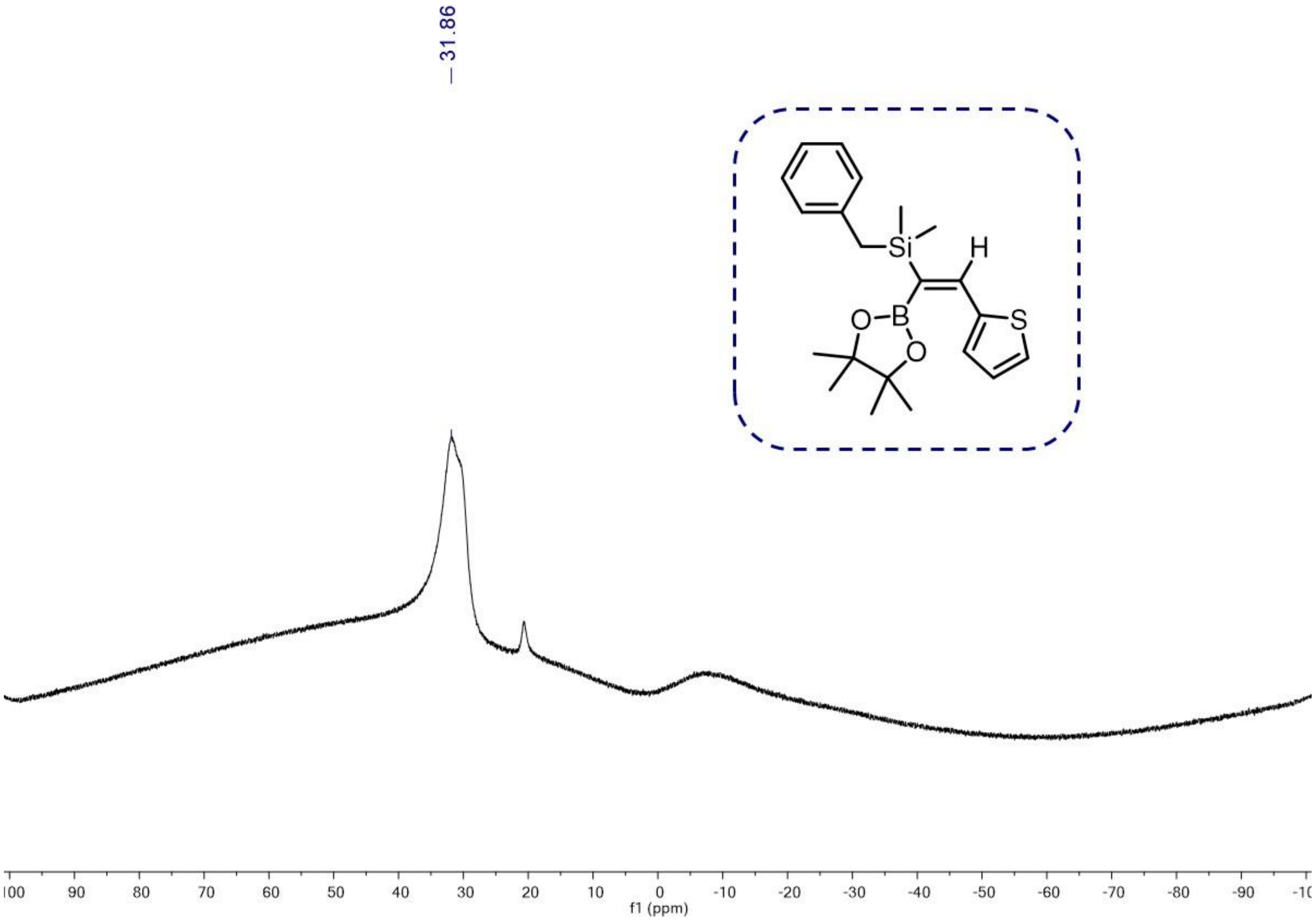

Figure S56. ^{11}B NMR spectrum of compound **3bk**.

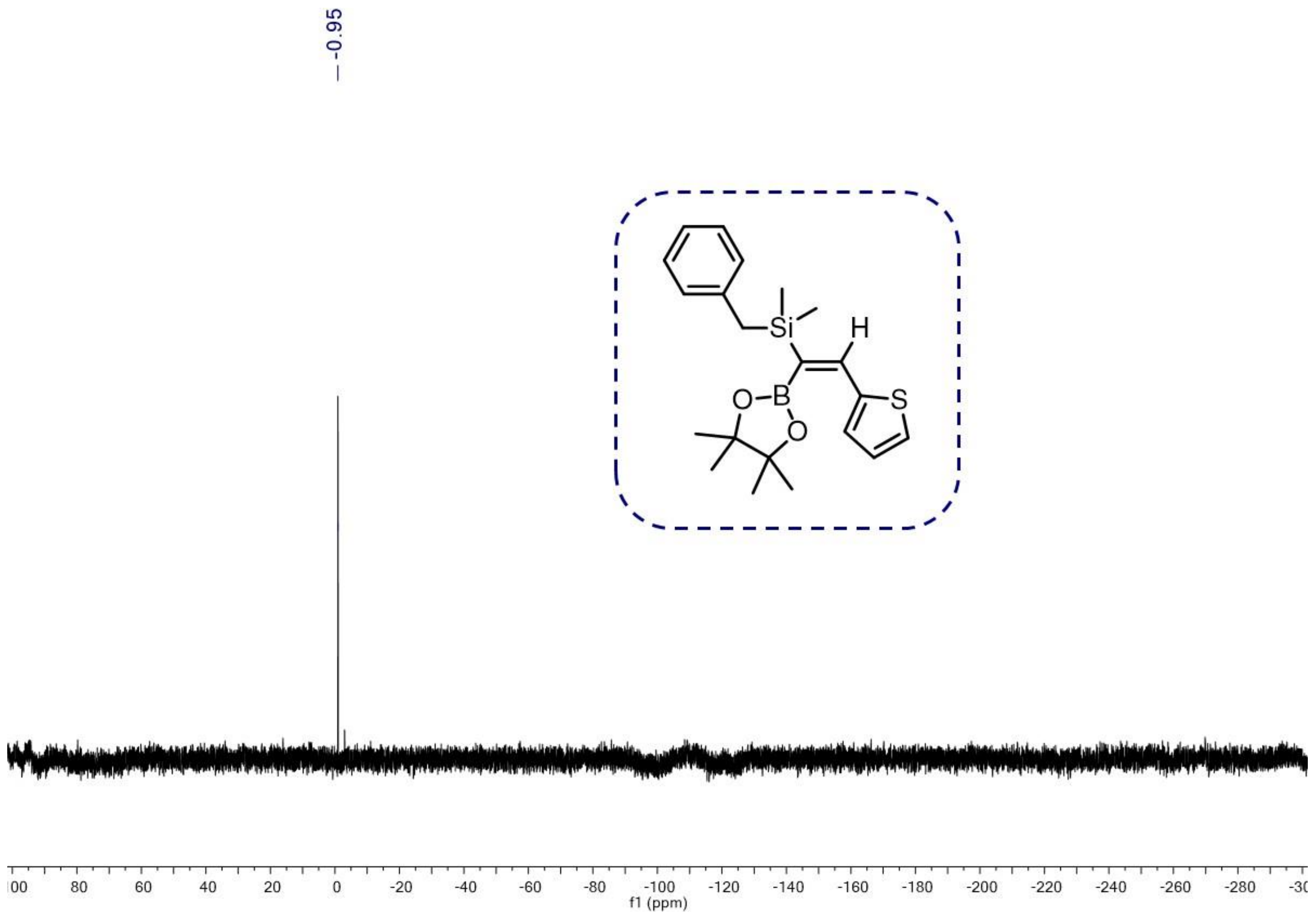

Figure S57. ^{29}Si NMR spectrum of compound **3bk**.

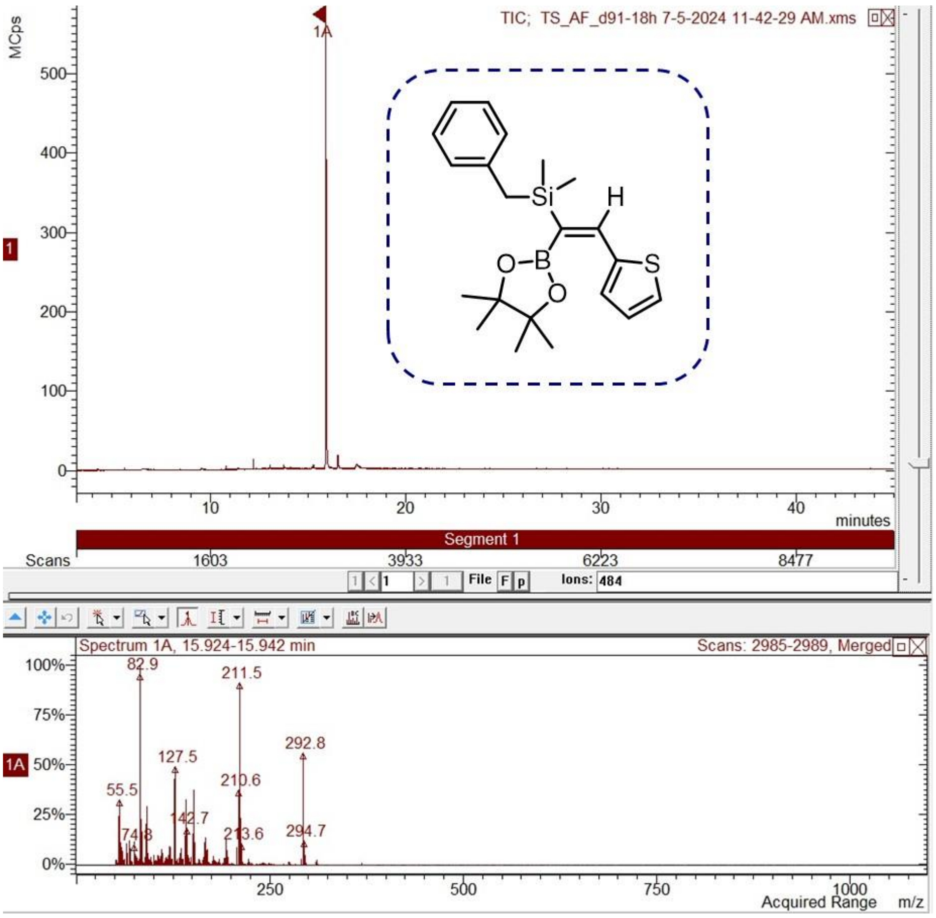

Figure S58. ^{29}Si NMR spectrum of compound **3bk**.

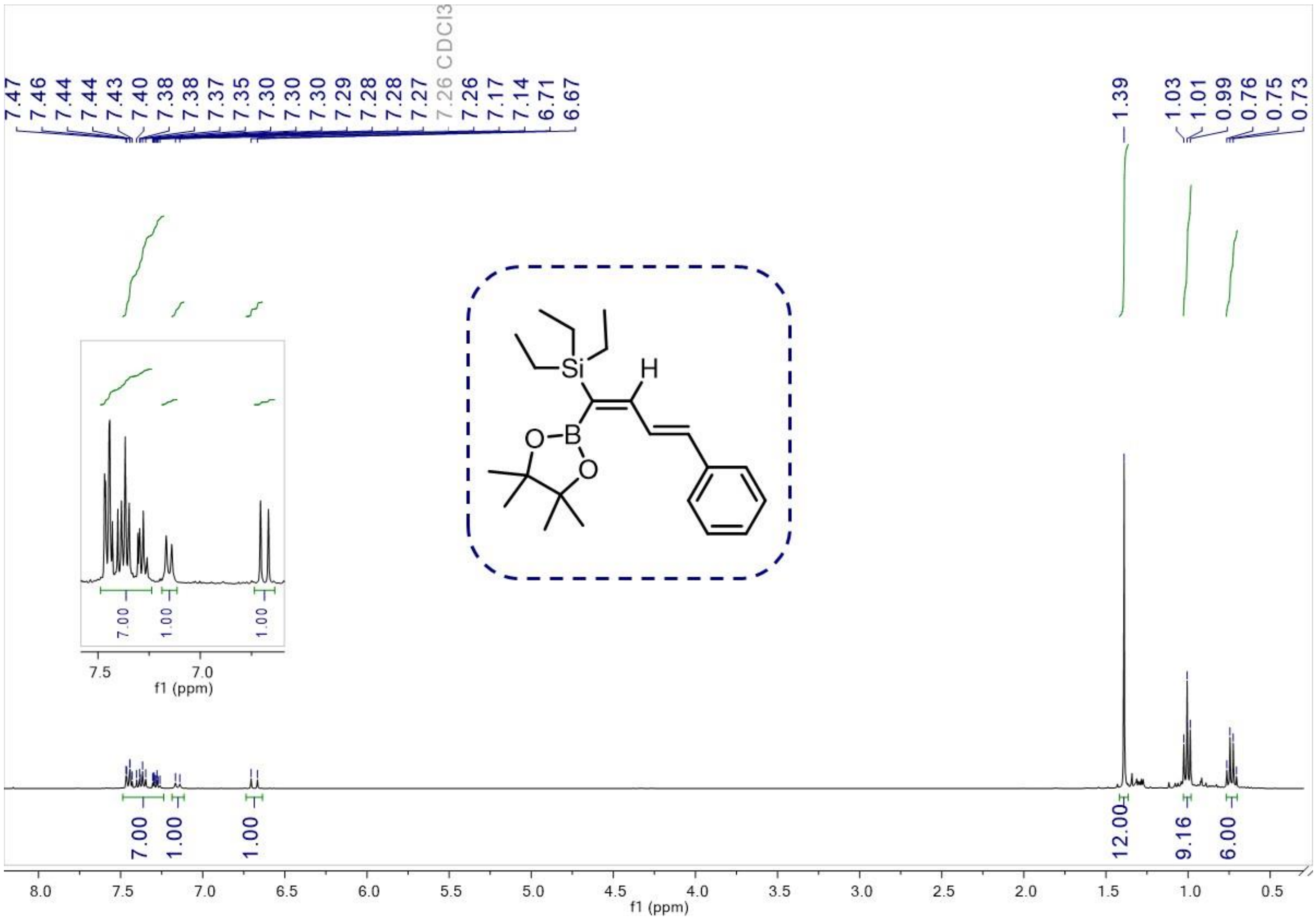

Figure S59. ^{1}H NMR spectrum of compound **3al**.

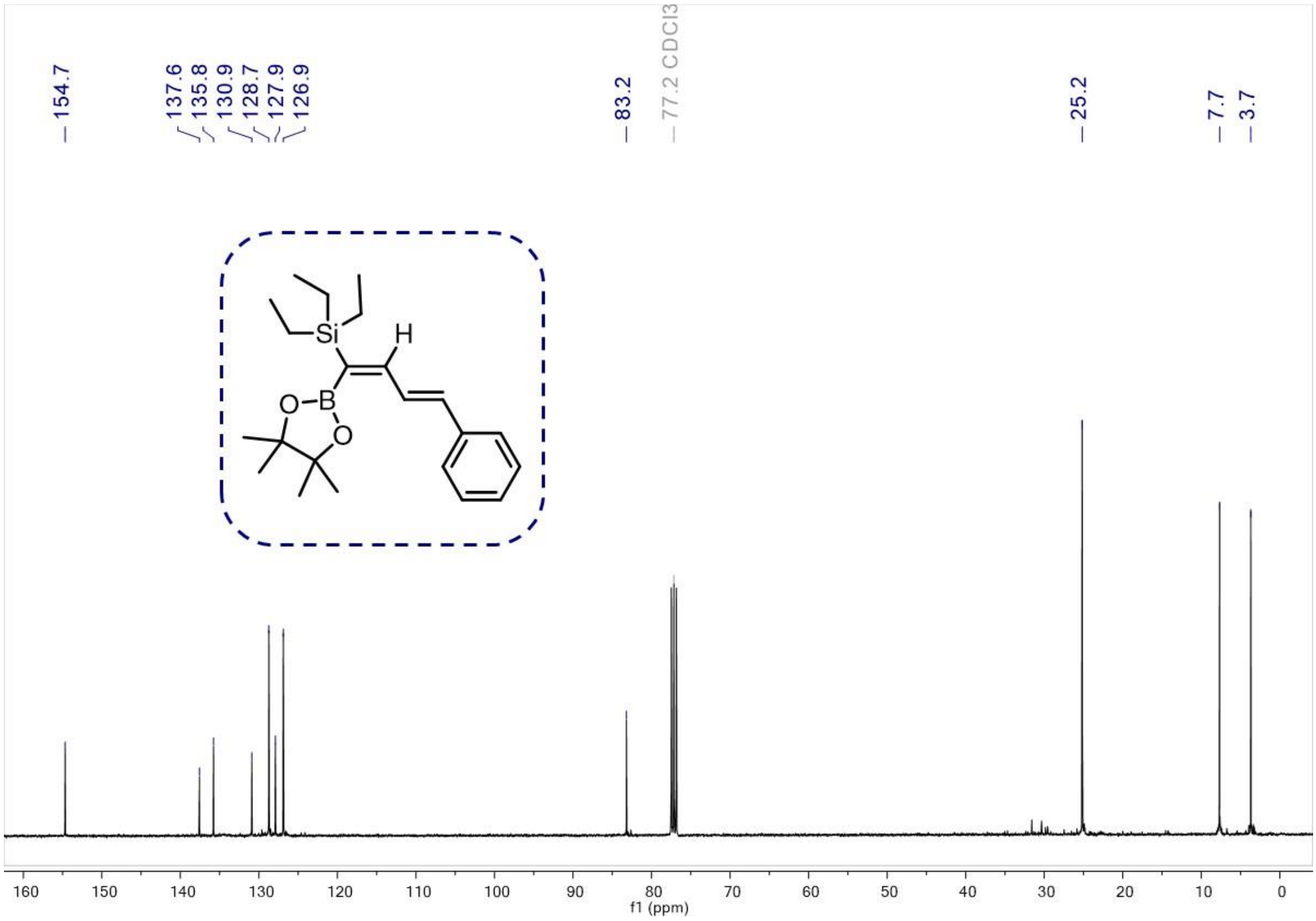

Figure S60. ^{13}C NMR spectrum of compound **3al**.

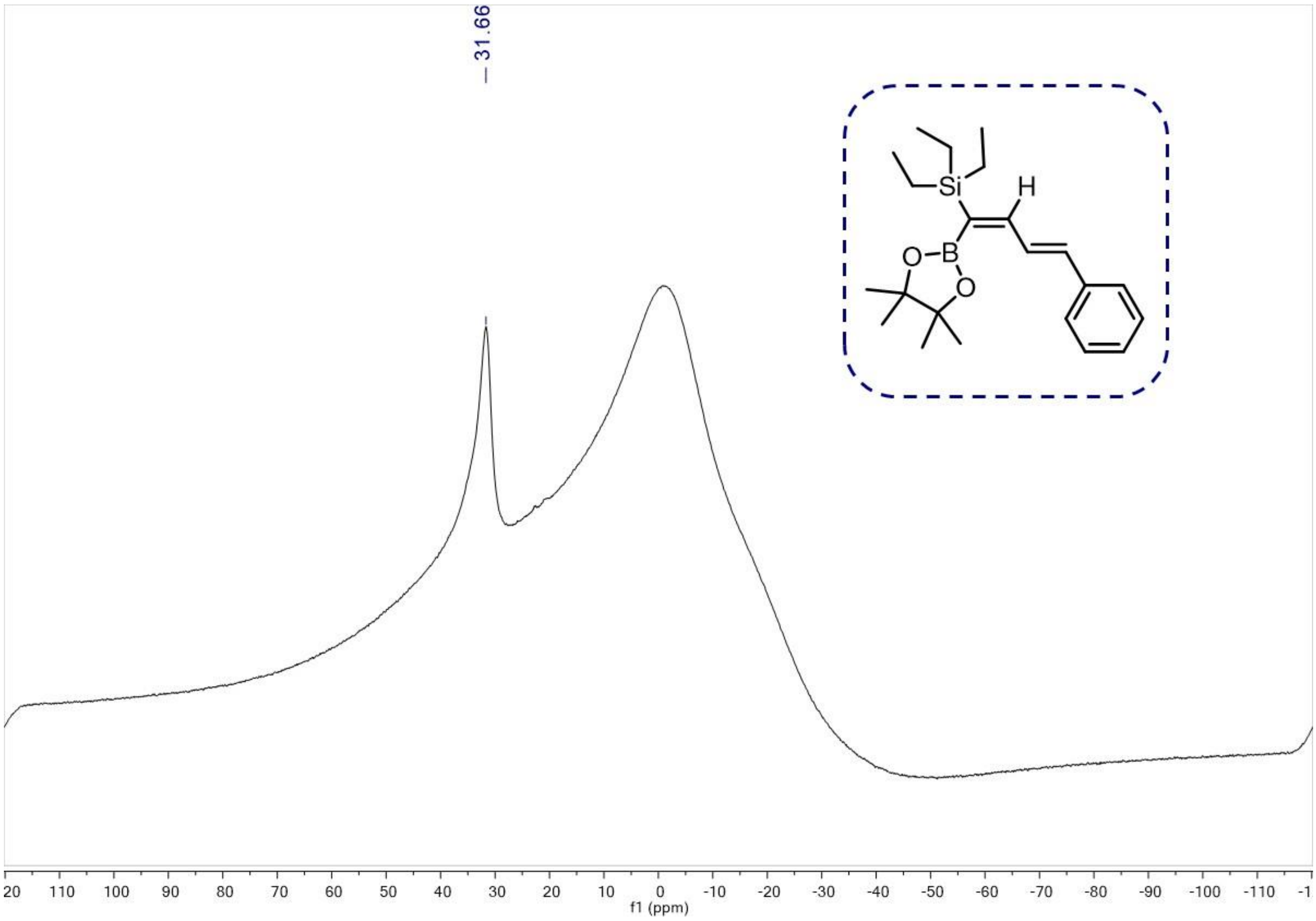

Figure S61. ^{11}B NMR spectrum of compound **3al**.

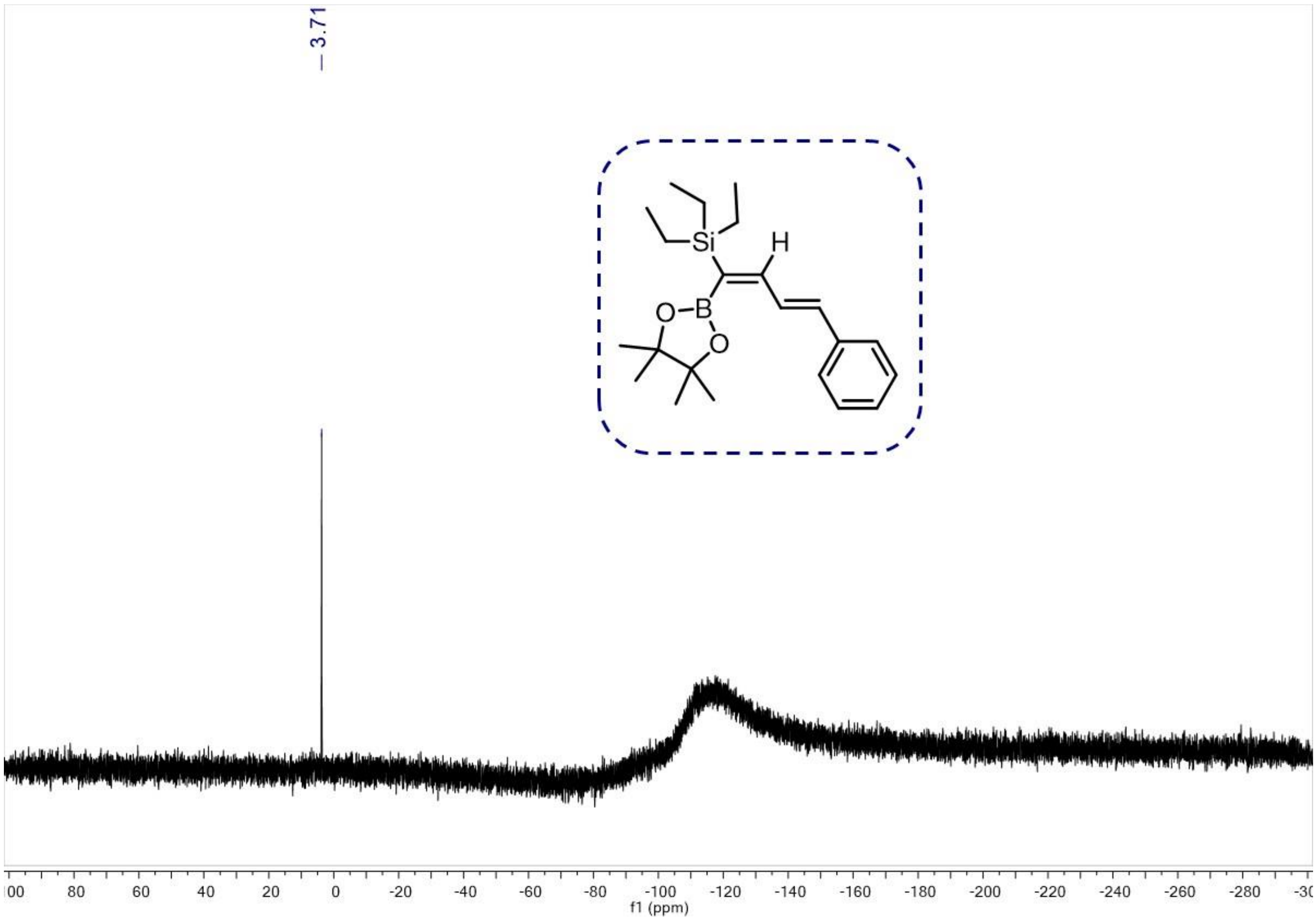

Figure S62. ^{29}Si NMR spectrum of compound **3al**.

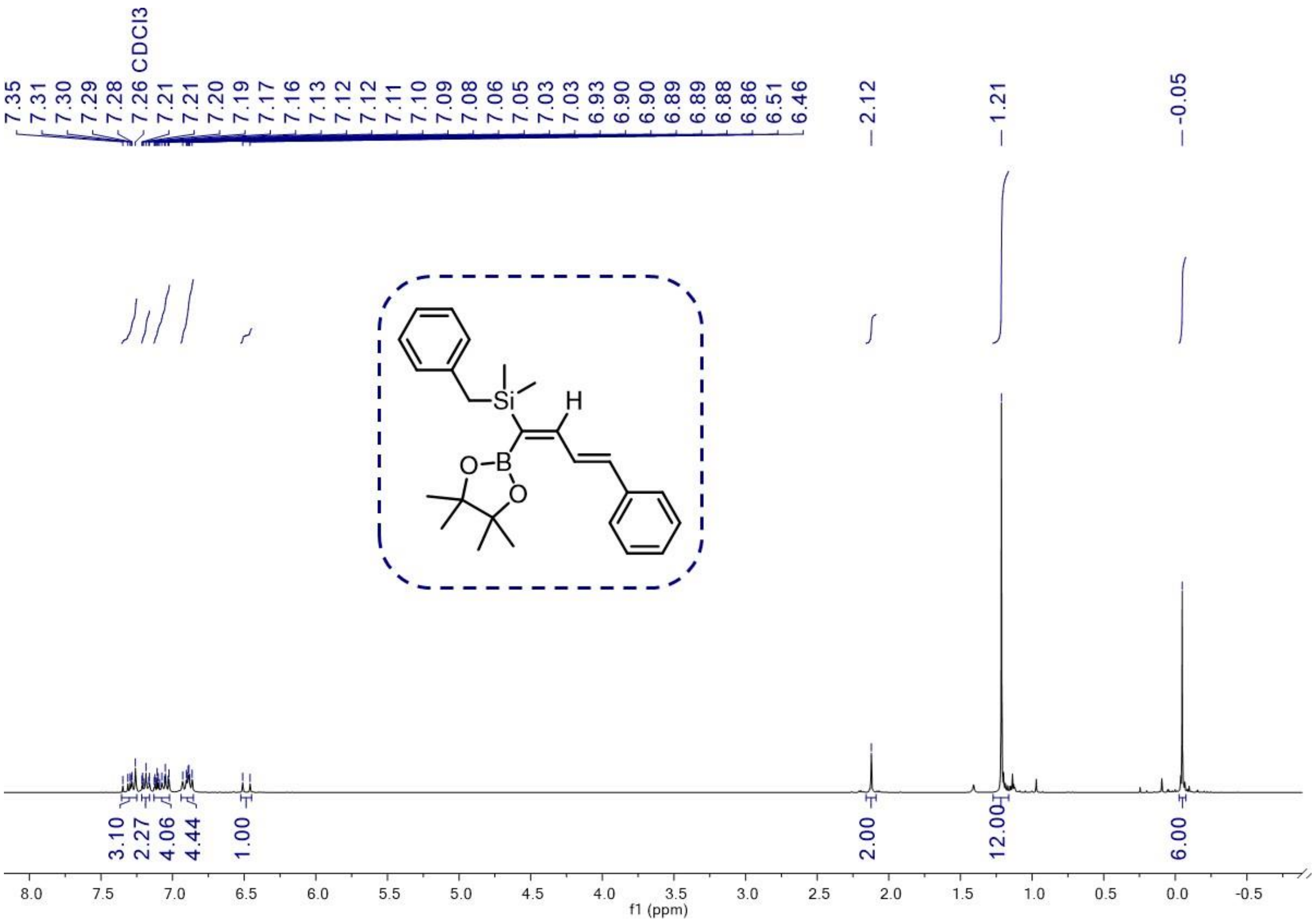

Figure S63. ^{1}H NMR spectrum of compound **3bl**.

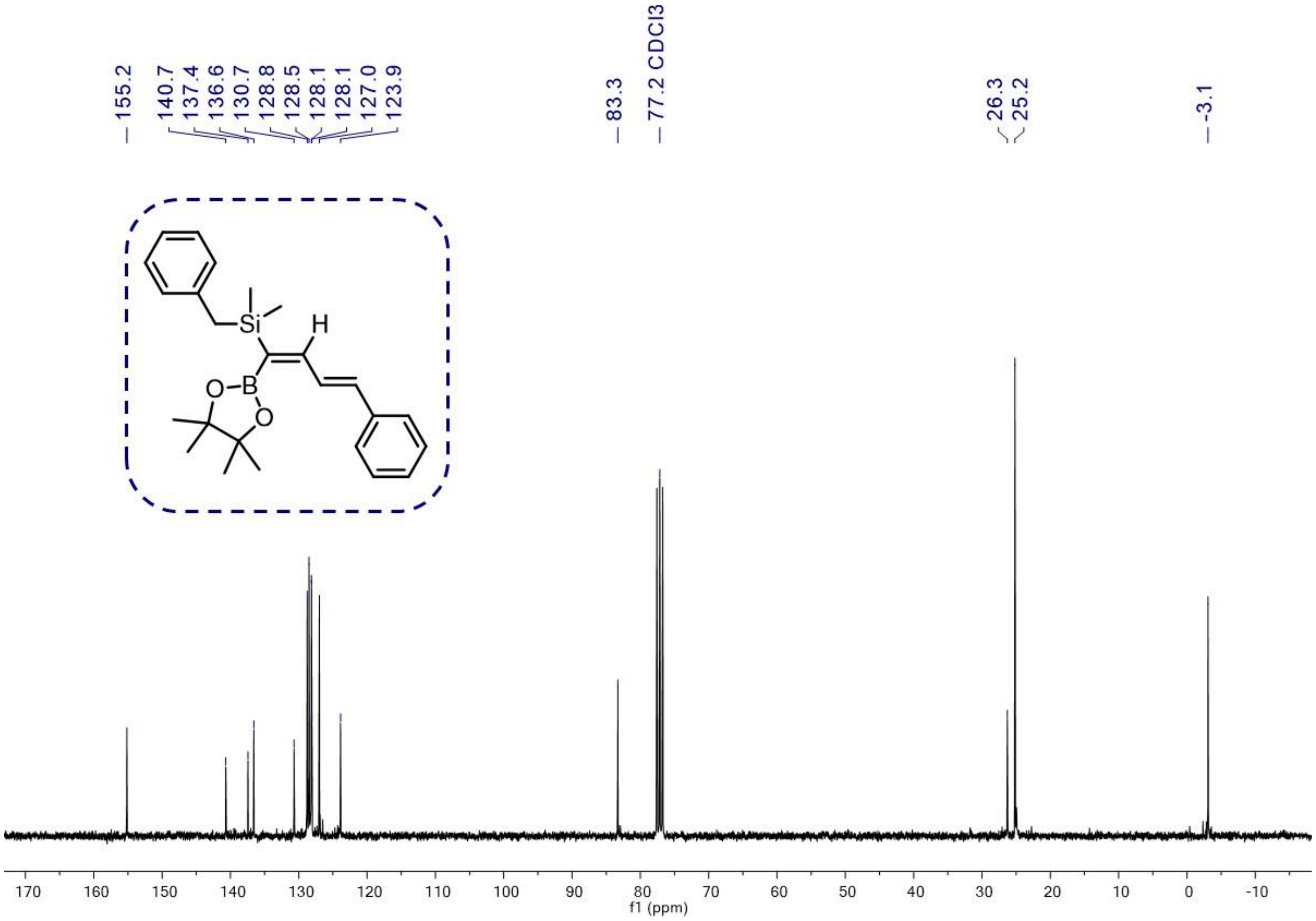

Figure S64. ^{13}C NMR spectrum of compound **3bl**.

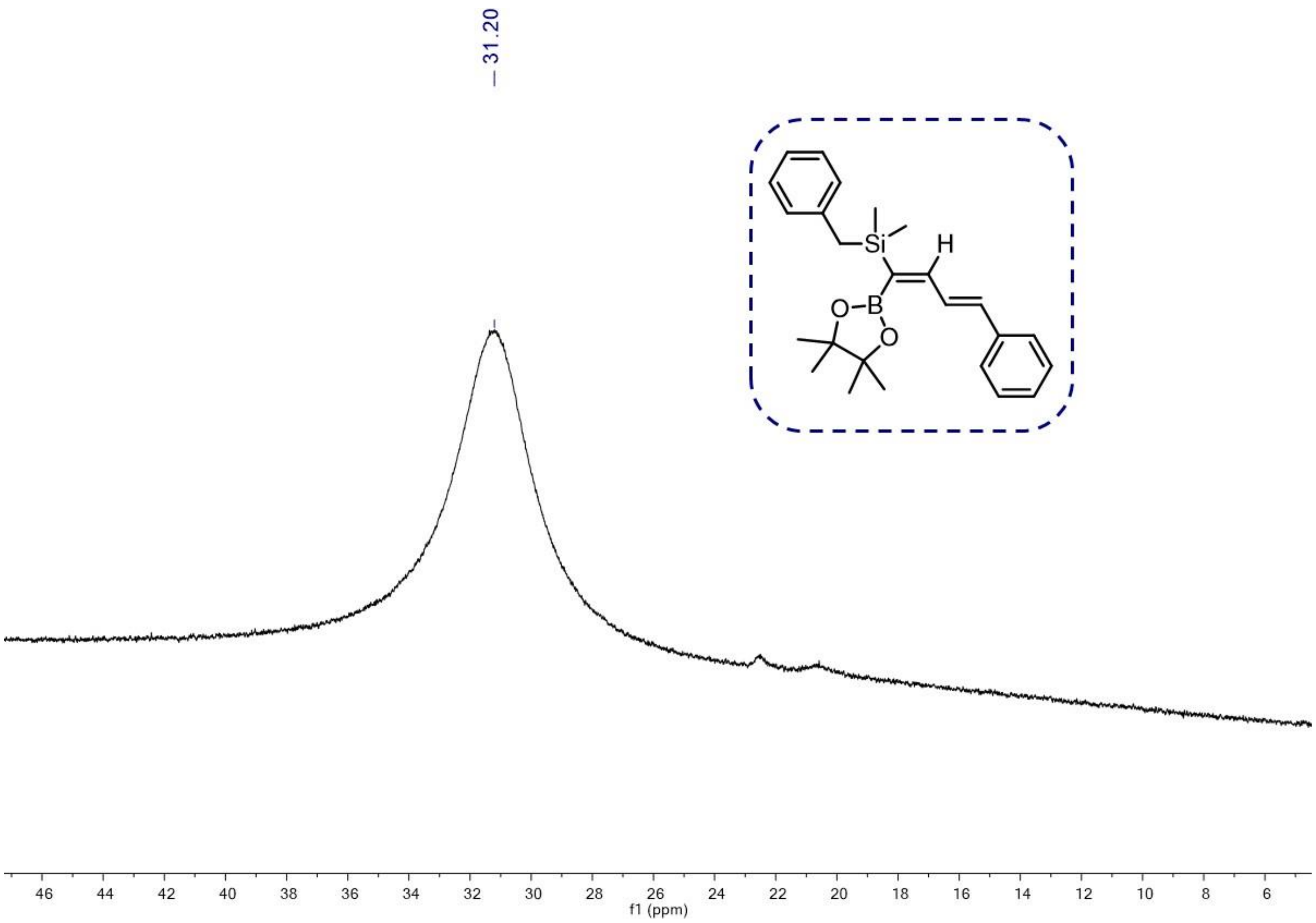

Figure S65. ^{11}B NMR spectrum of compound **3bl**.

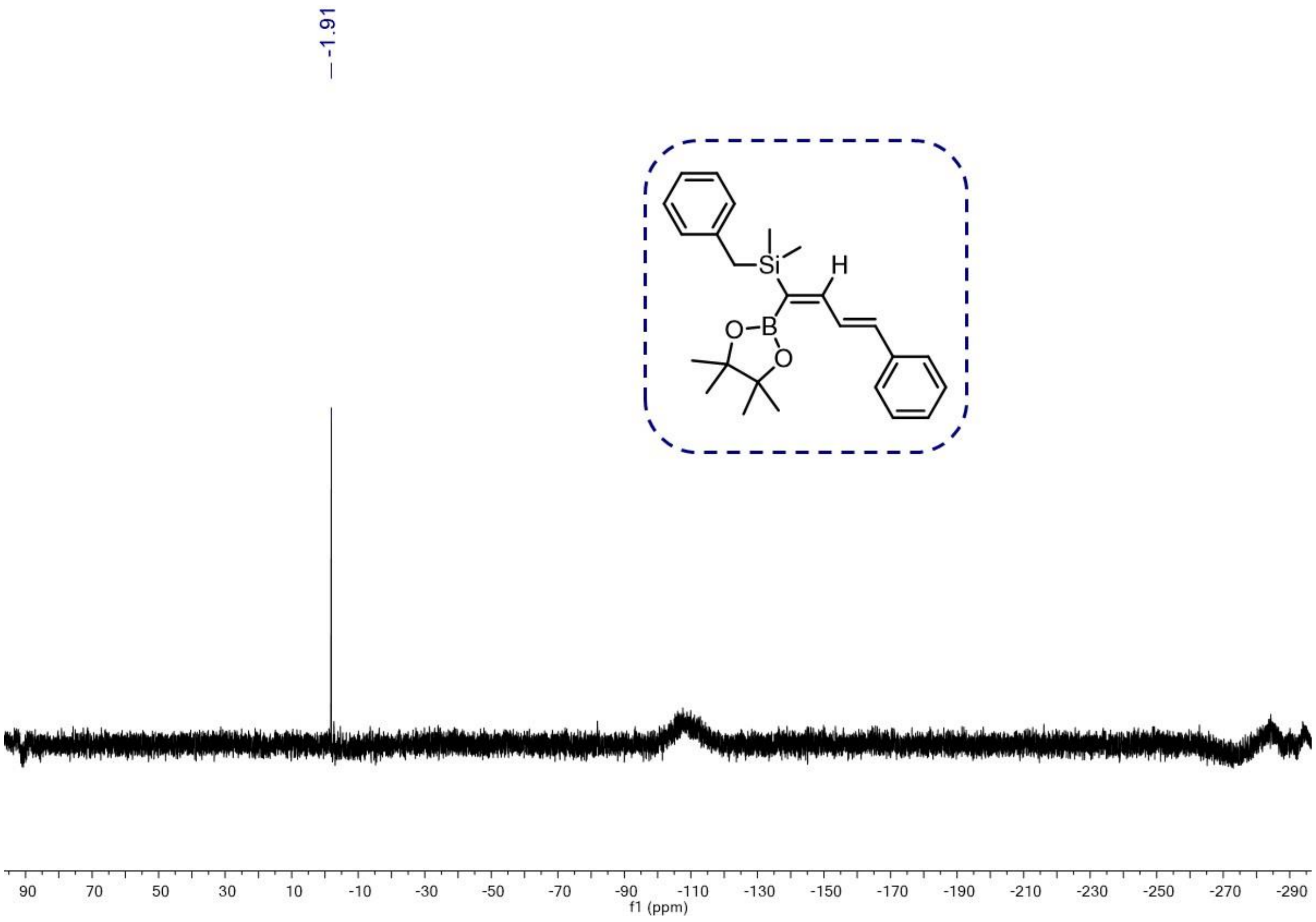

Figure S66. ^{29}Si NMR spectrum of compound **3bl**.

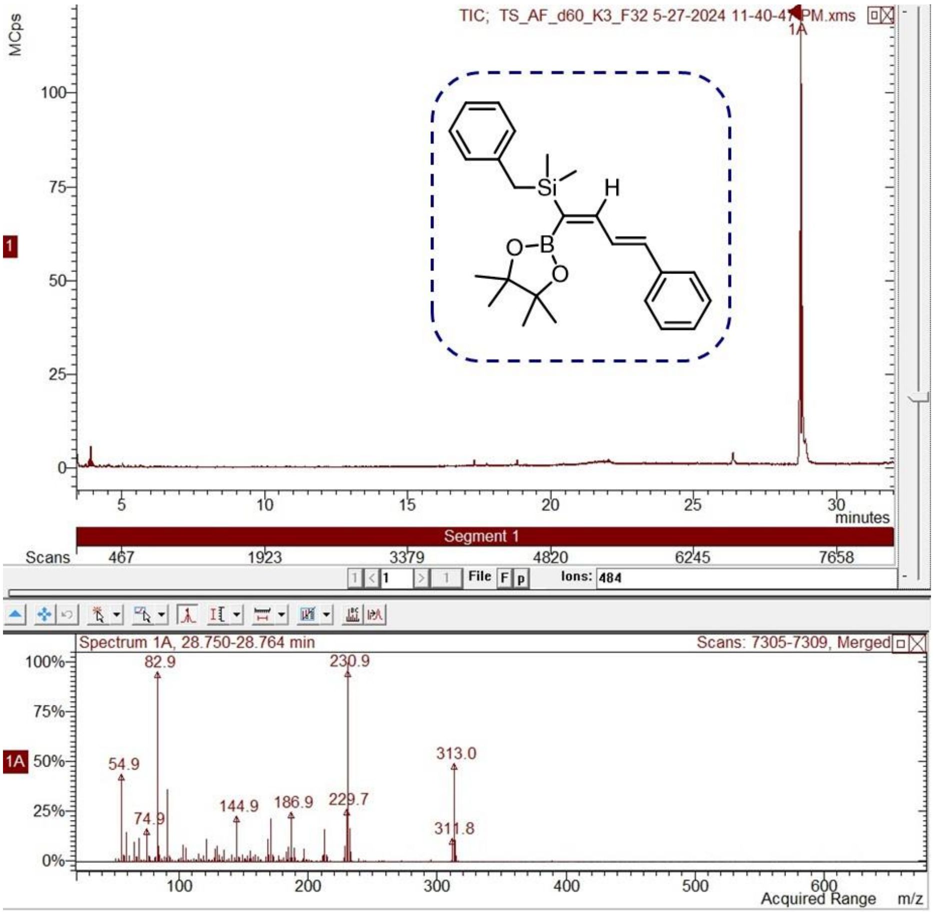

Figure S67. GC-MS image of compound **3bl**.

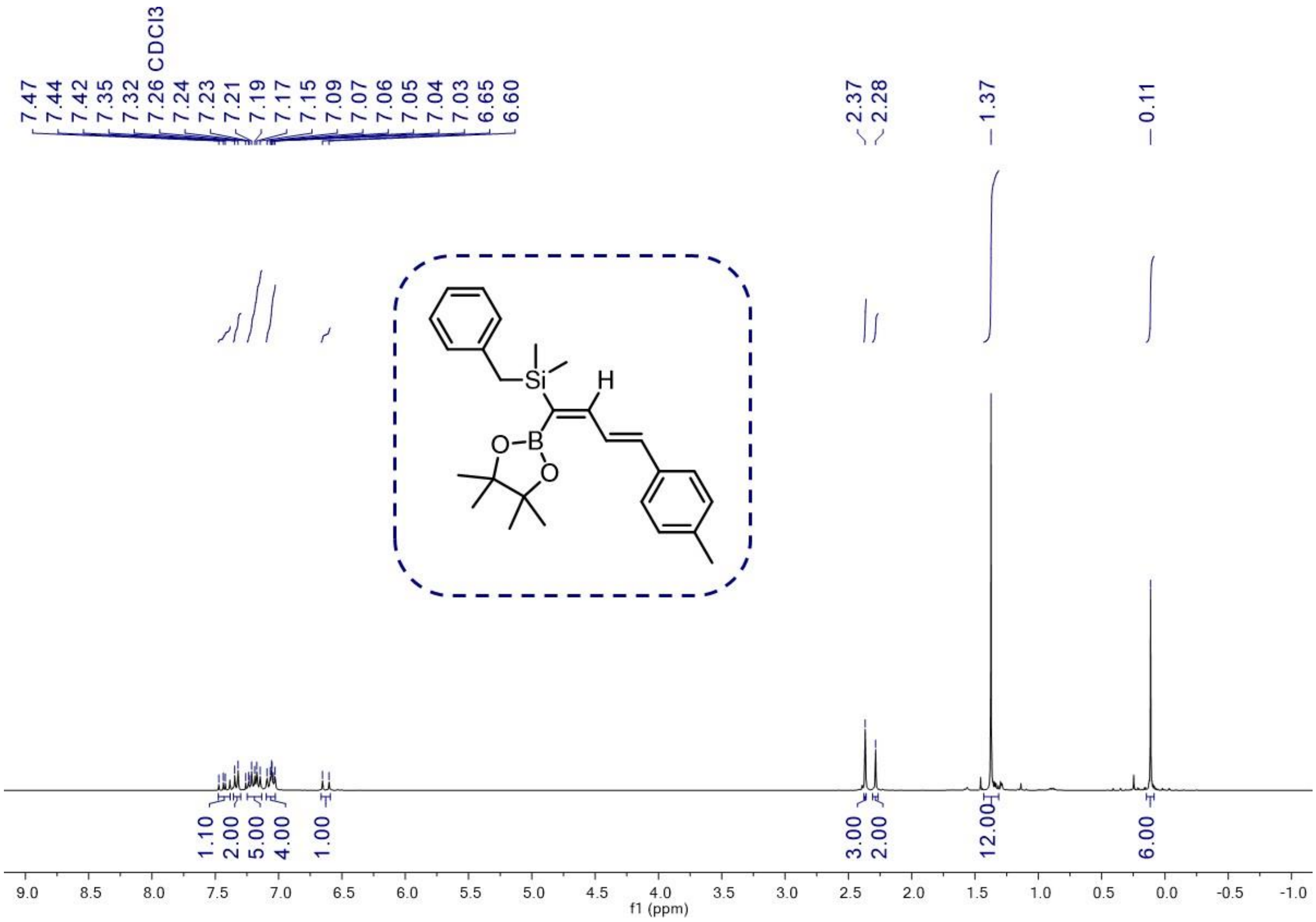

Figure S68. ^{1}H NMR spectrum of compound **3bm**.

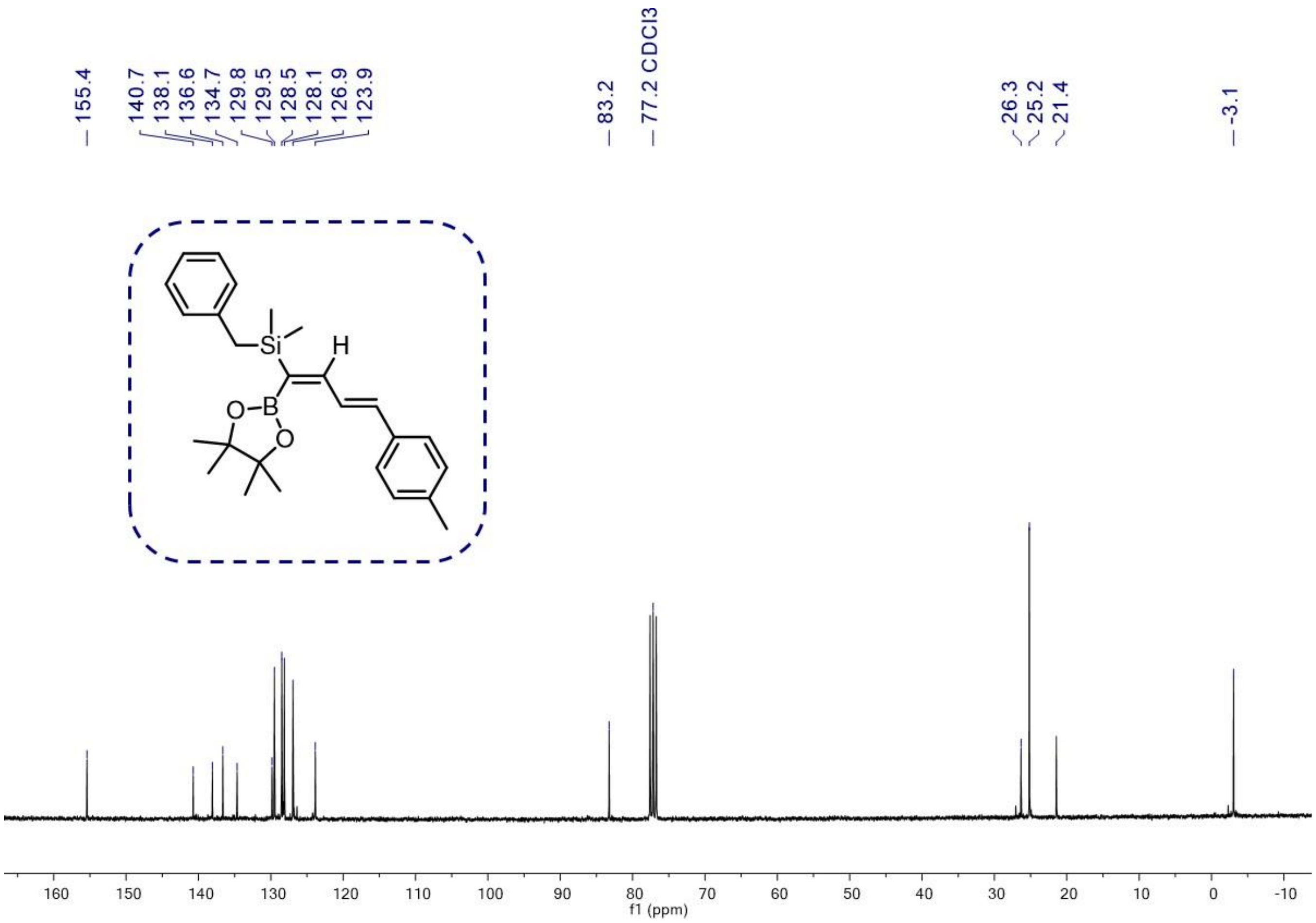

Figure S69. ^{13}C NMR spectrum of compound **3bm**.

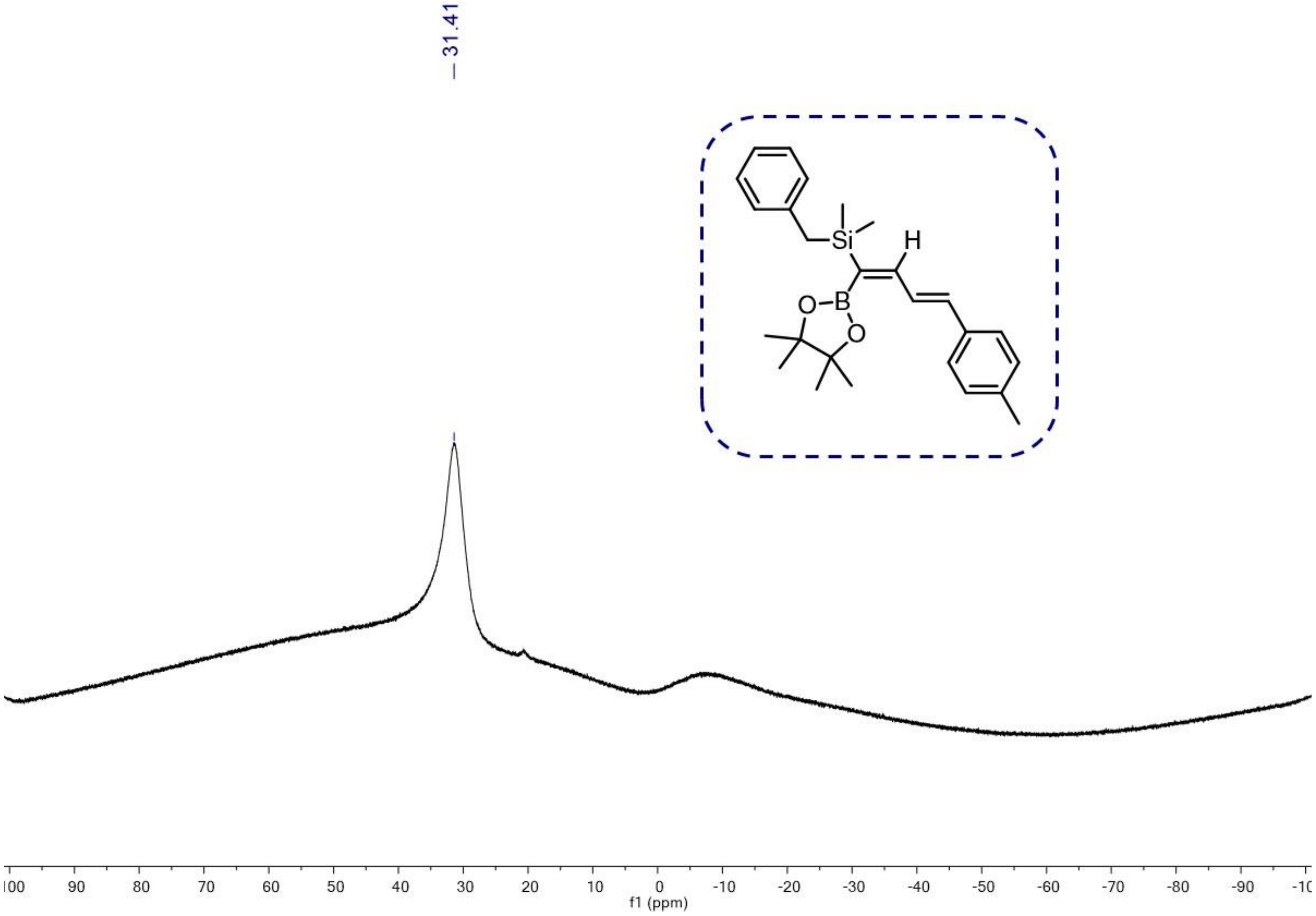

Figure S70. ^{11}B NMR spectrum of compound **3bm**.

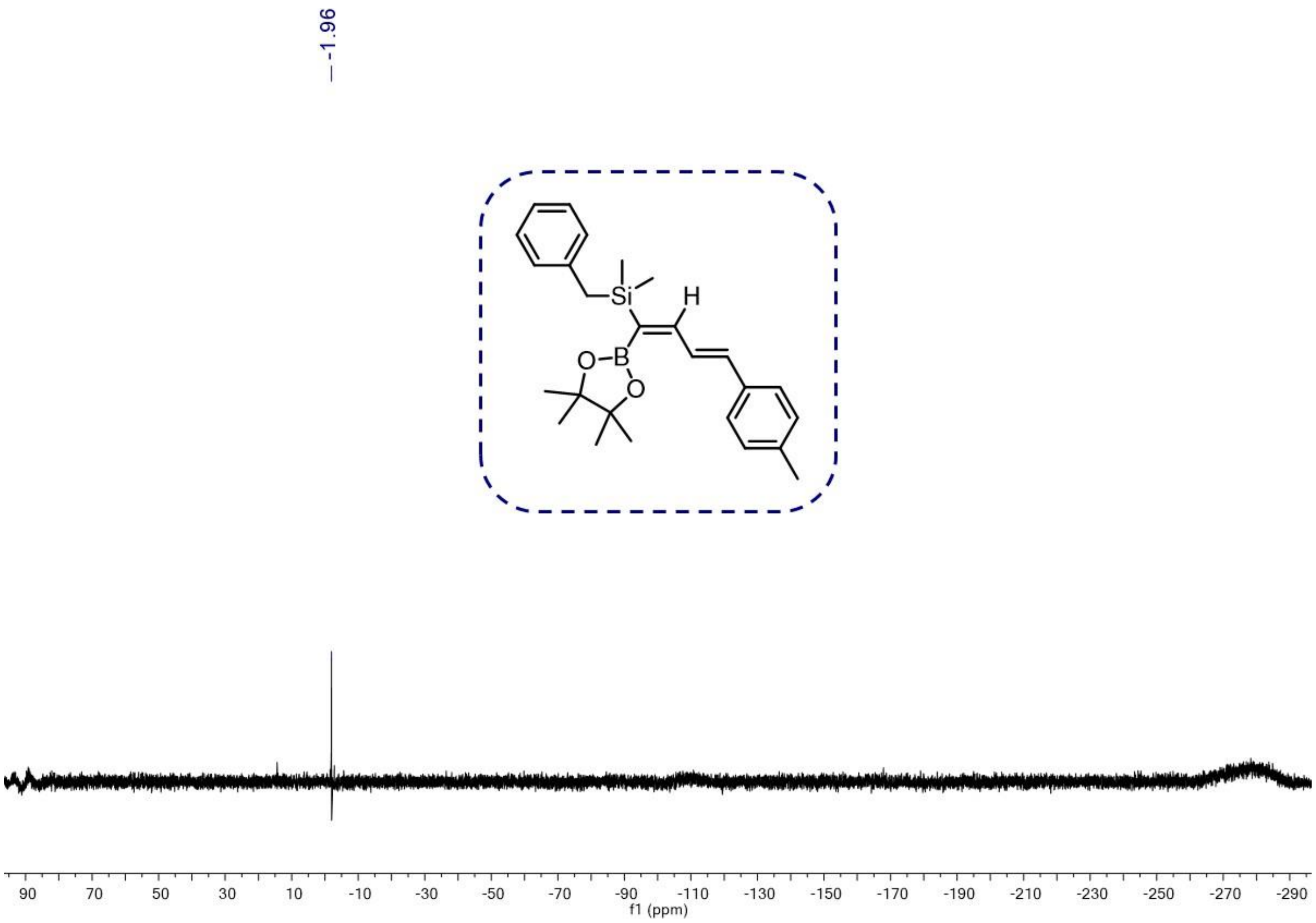

Figure S71. ^{29}Si NMR spectrum of compound **3bm**.

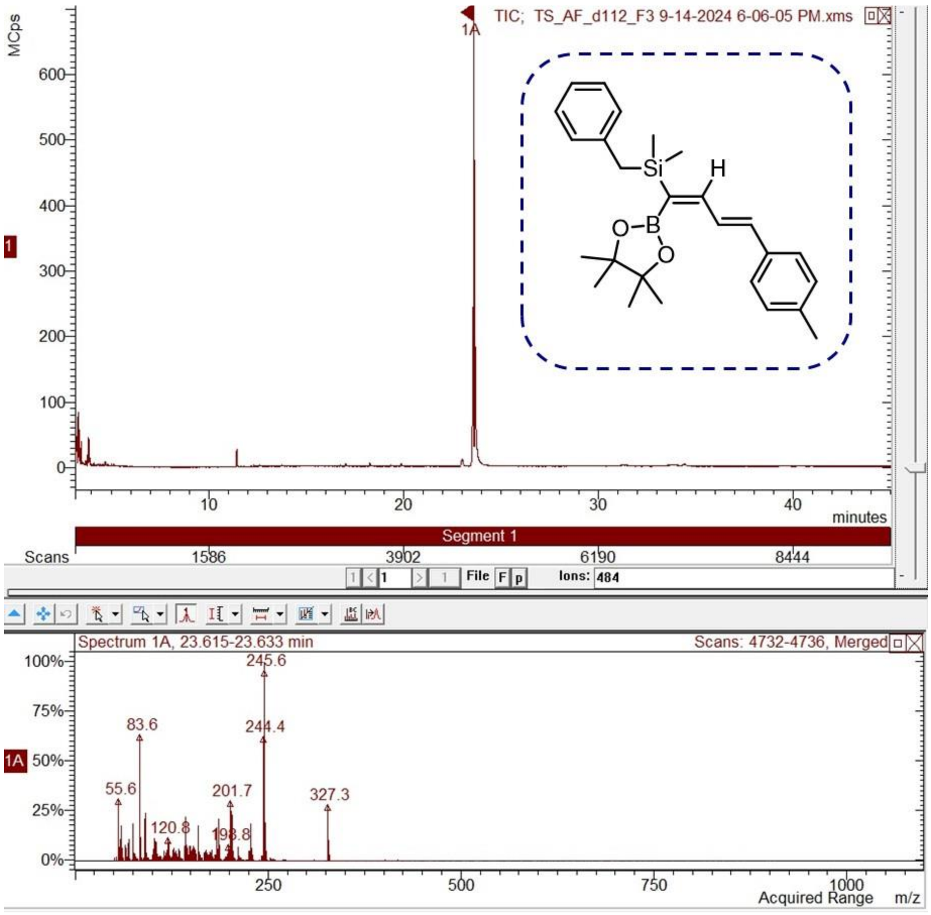

Figure S72. GC-MS image of compound **3bm**.

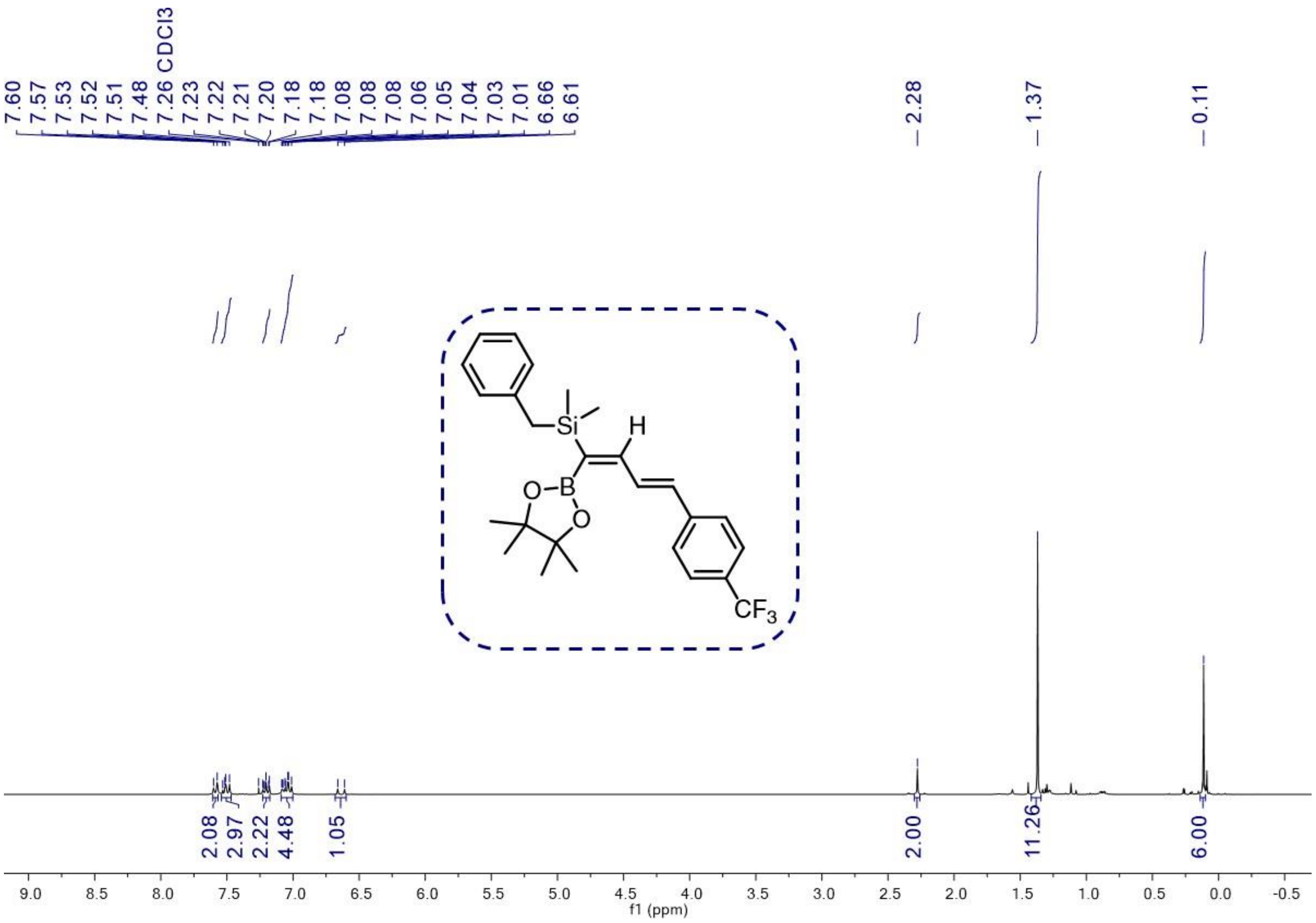

Figure S73. ^{1}H NMR spectrum of compound **3bn**.

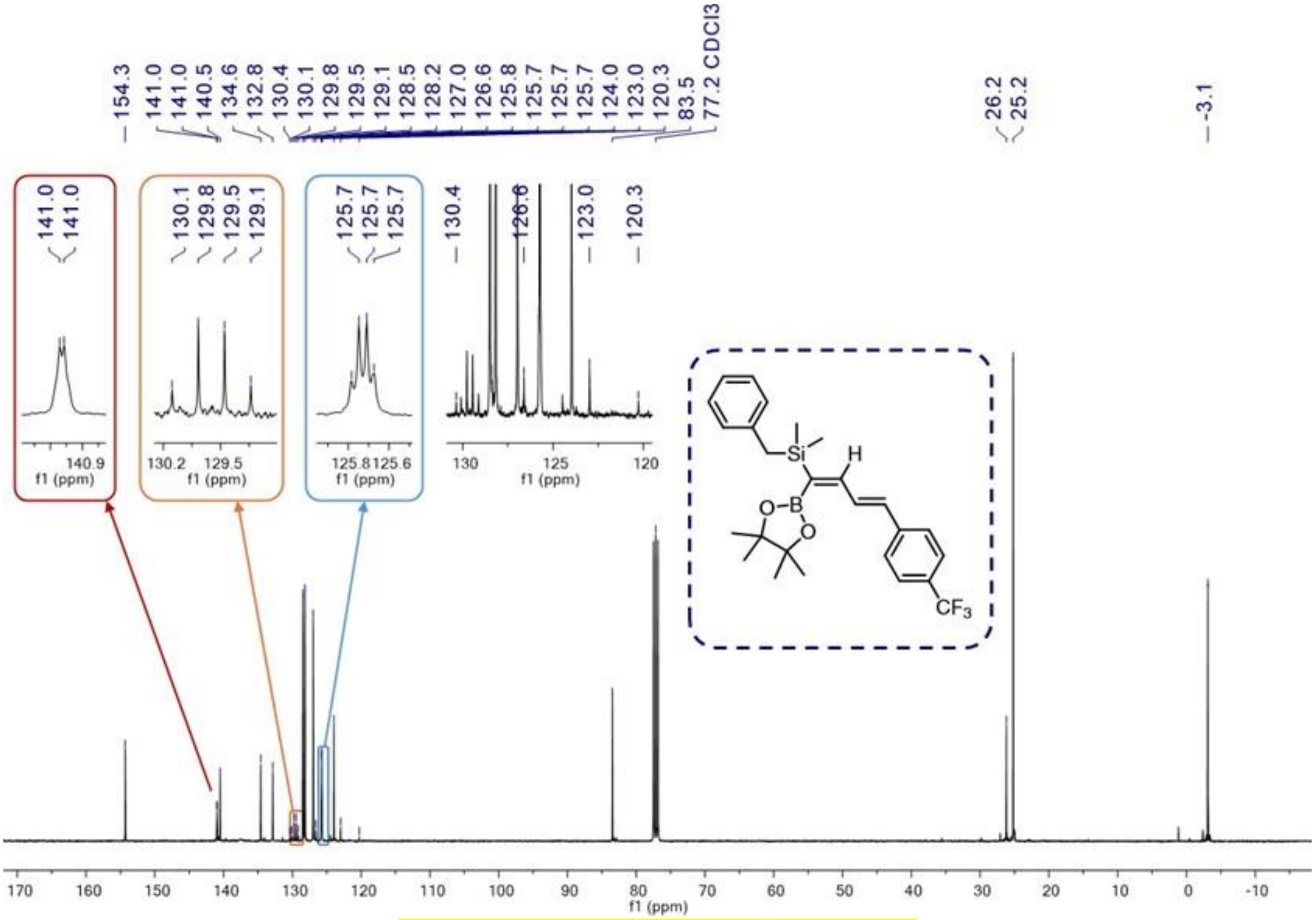

Figure S74. ^{13}C NMR spectrum of compound **3bn**.

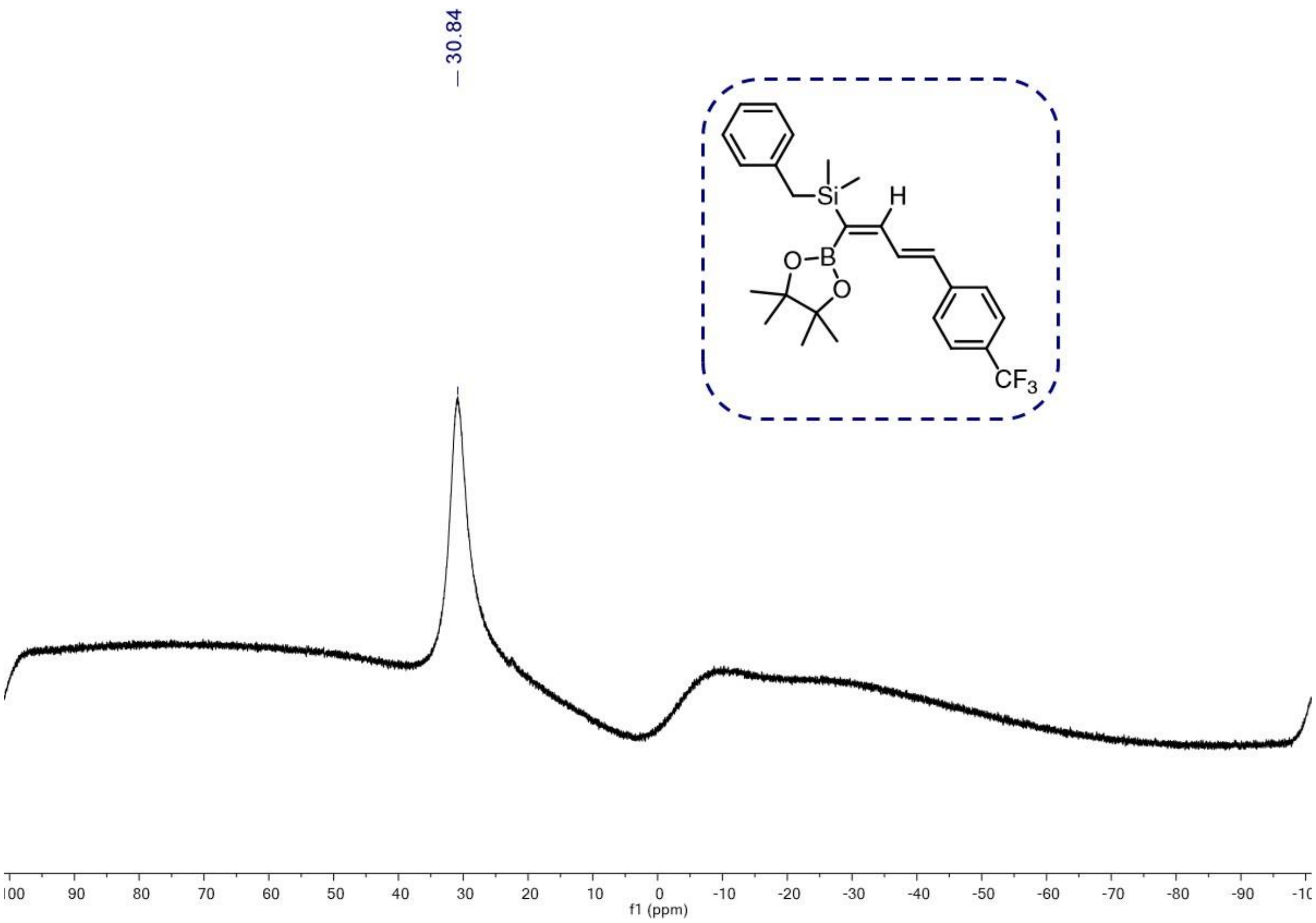

Figure S75. ^{11}B NMR spectrum of compound **3bn**.

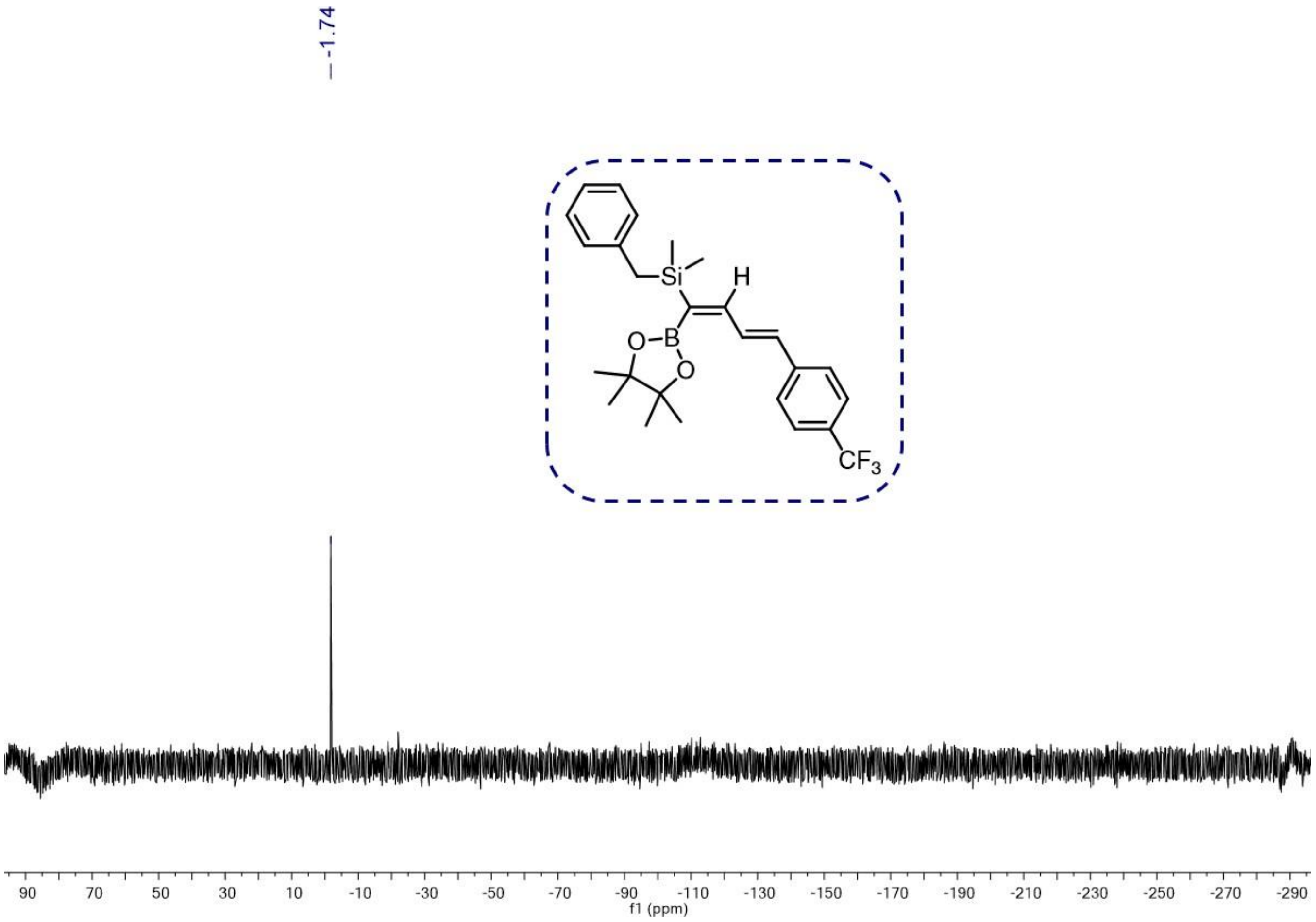

Figure S76. ^{29}Si NMR spectrum of compound **3bn**.

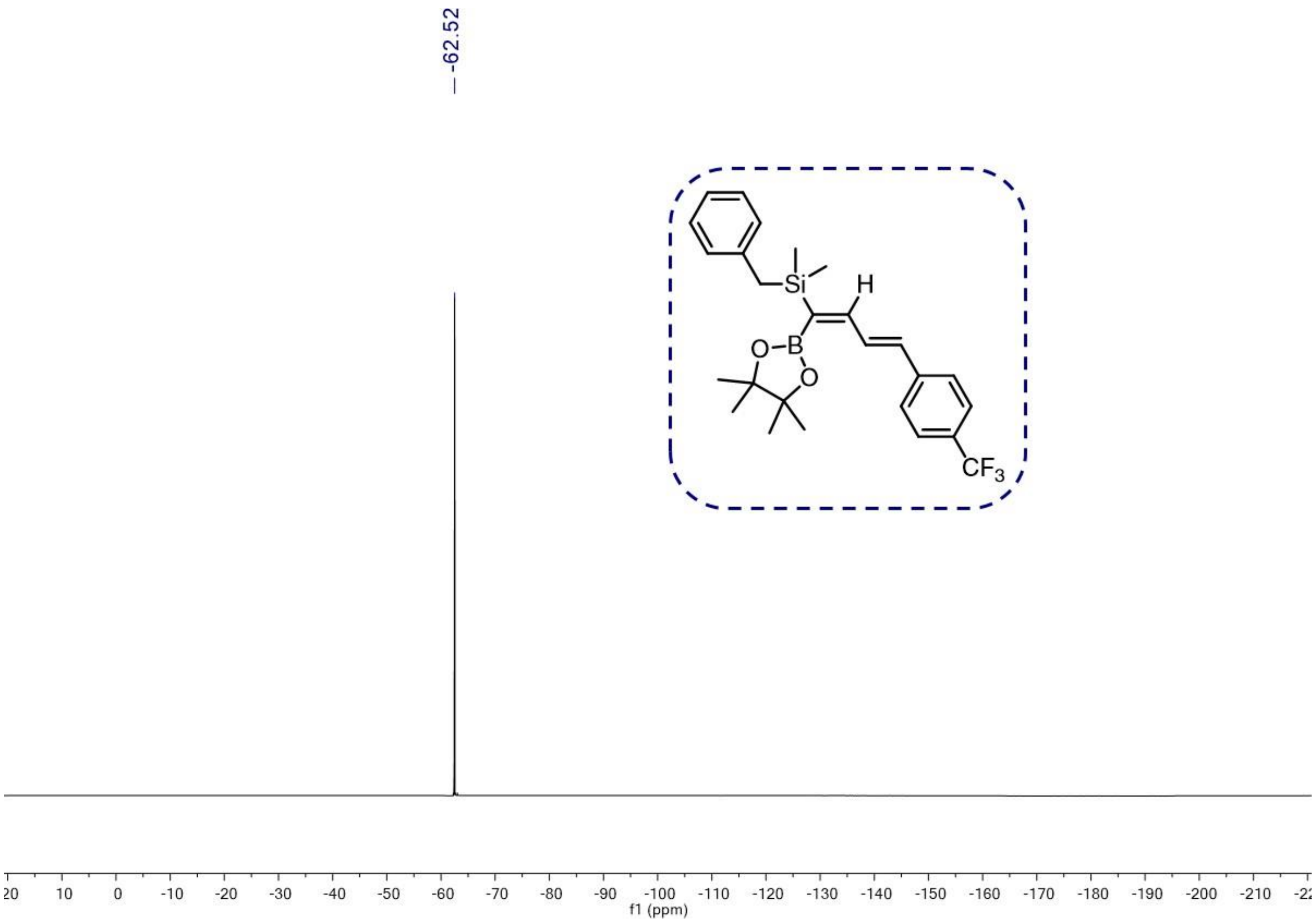

Figure S77. ^{19}F NMR spectrum of compound **3bn**.

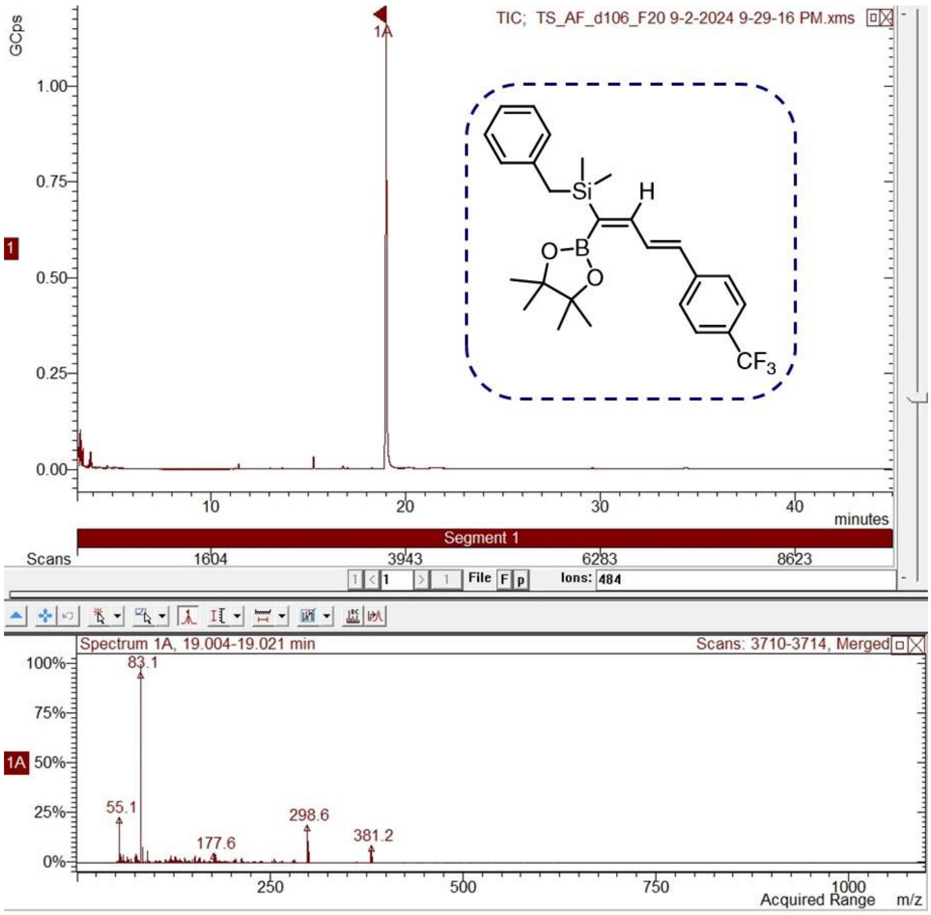

Figure S78. GC-MS image of compound **3bn**.

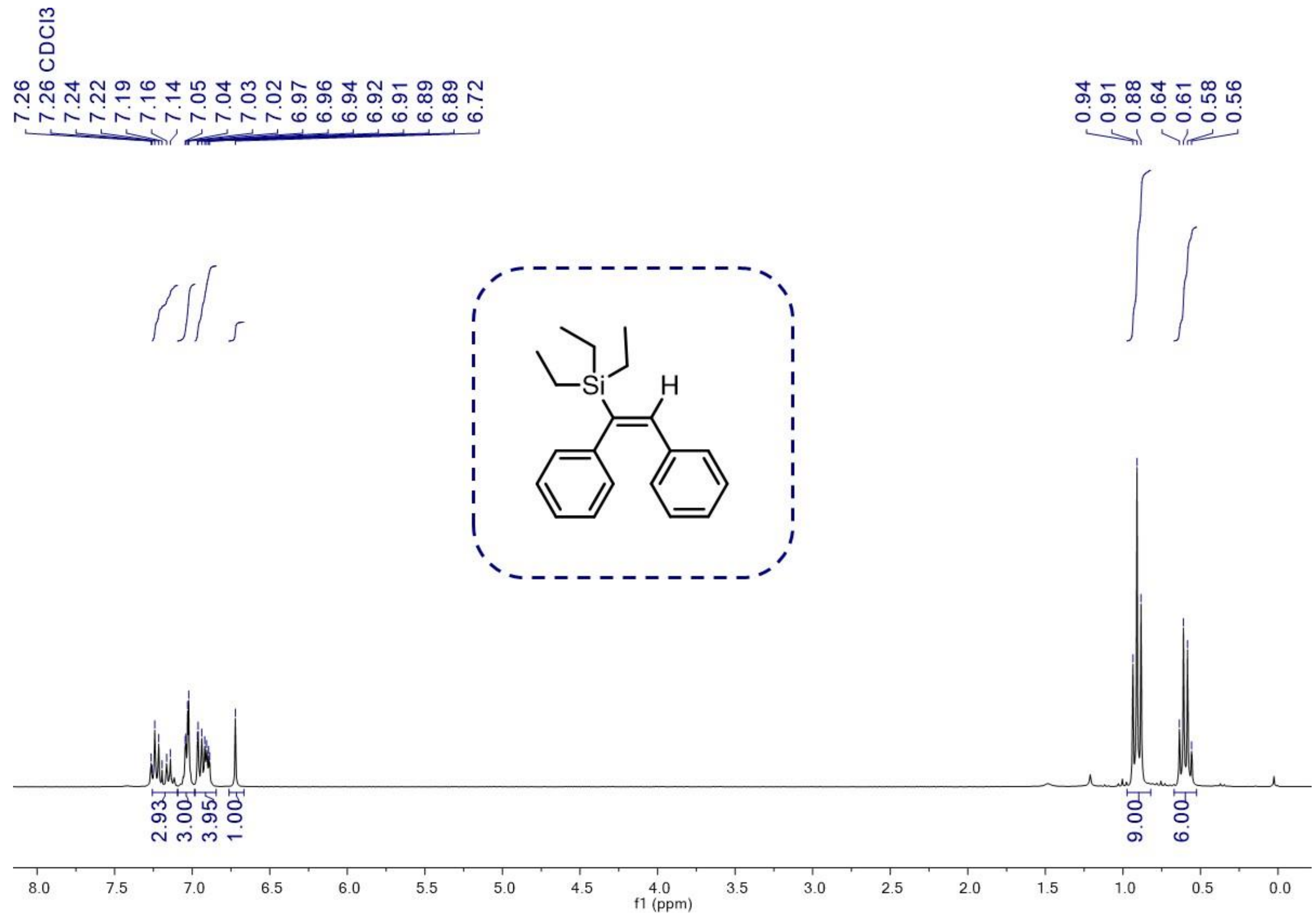

Figure S79. ^{1}H NMR spectrum of compound **4aaa**.

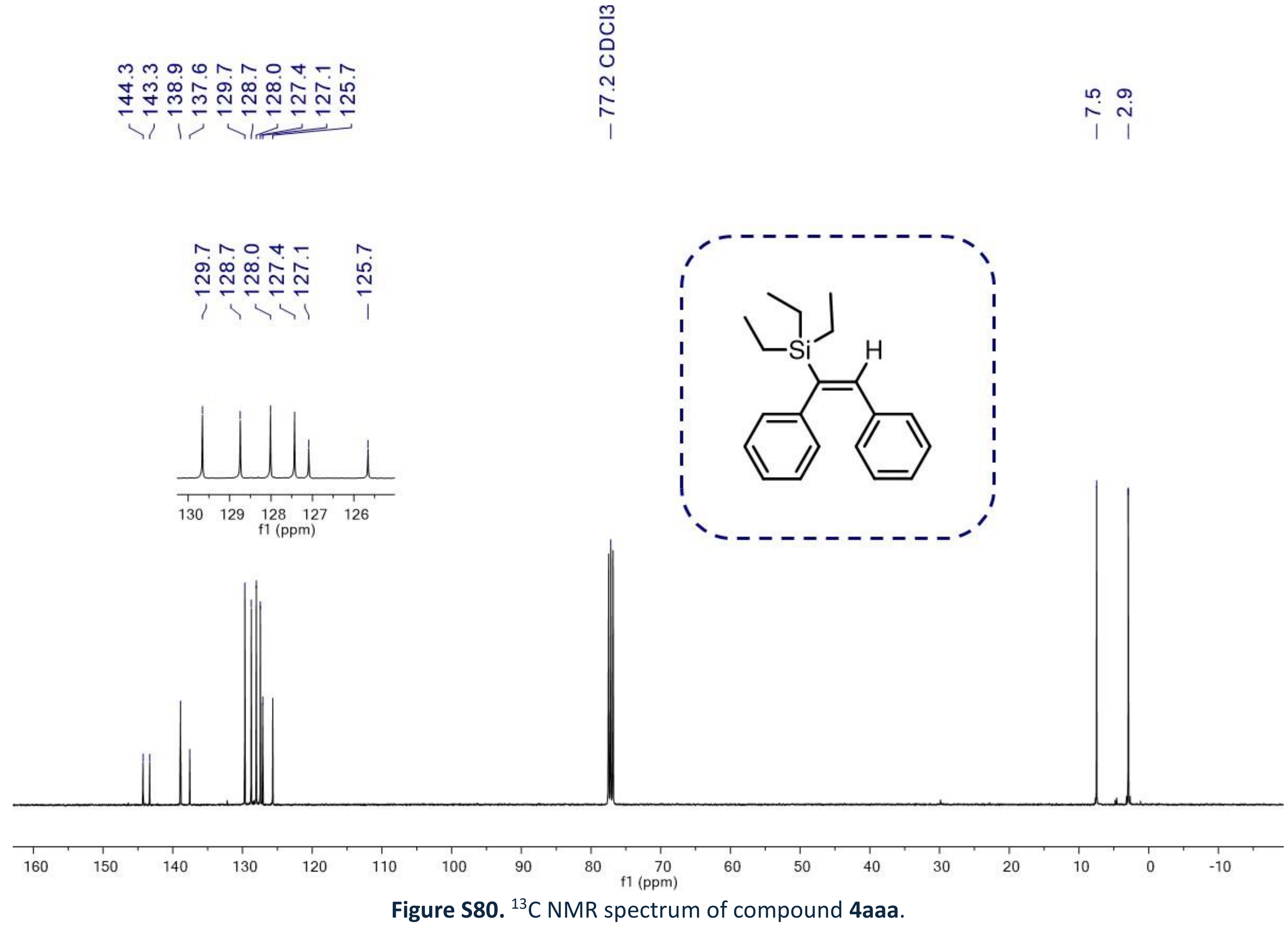

Figure S80. ^{13}C NMR spectrum of compound **4aaa**.

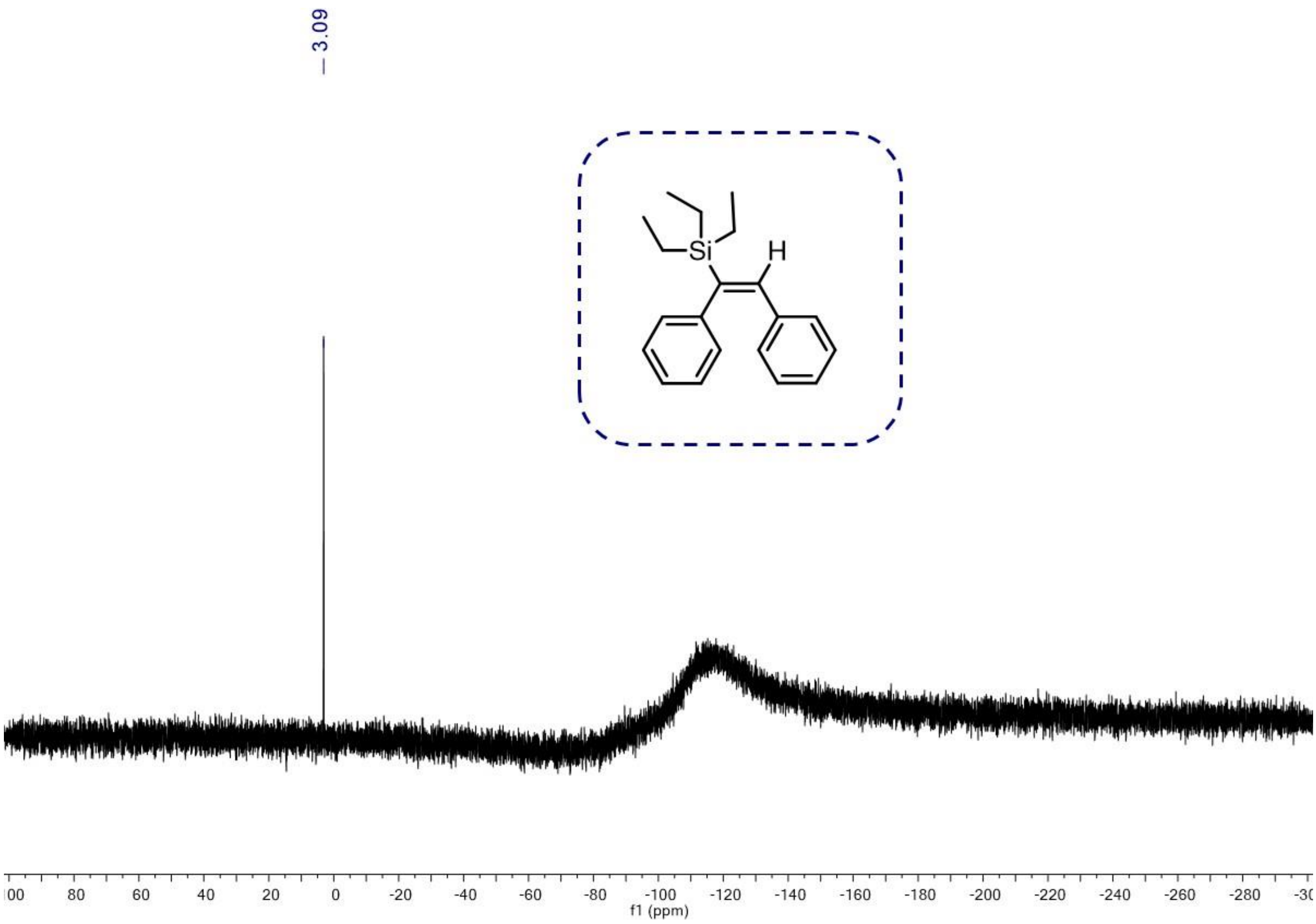

Figure S81. ^{29}Si NMR spectrum of compound **4aaa**.

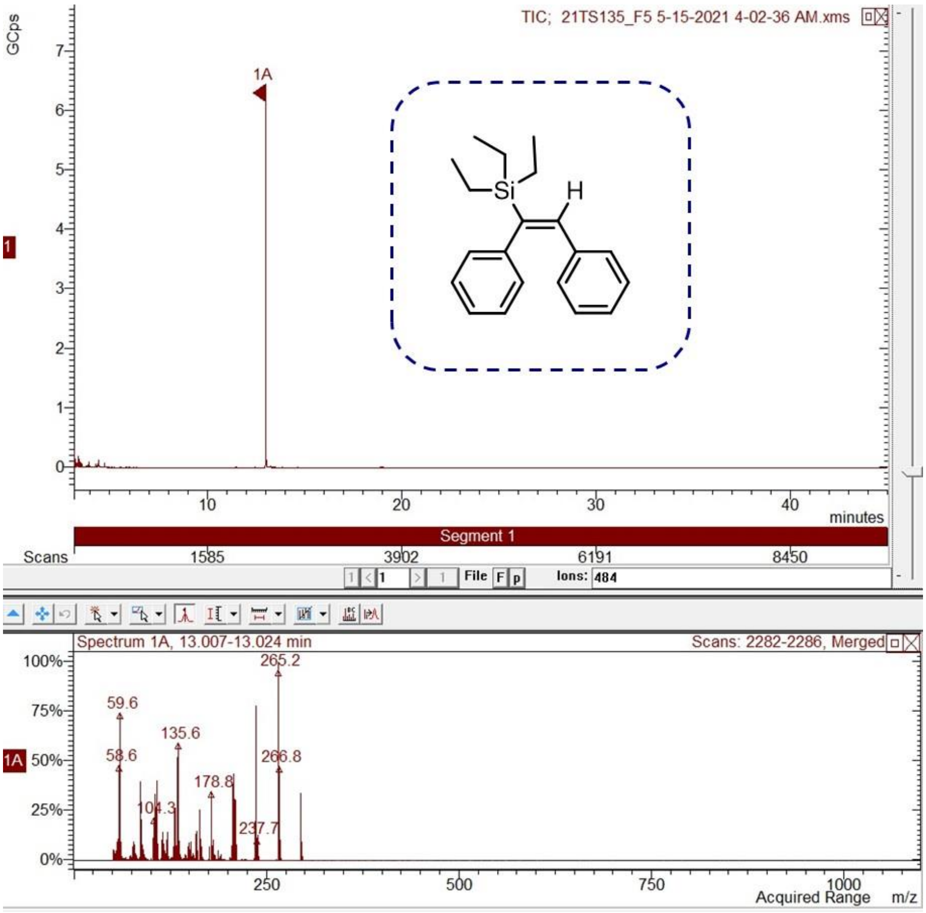

Figure S82. GC-MS image of compound **4aaa**.

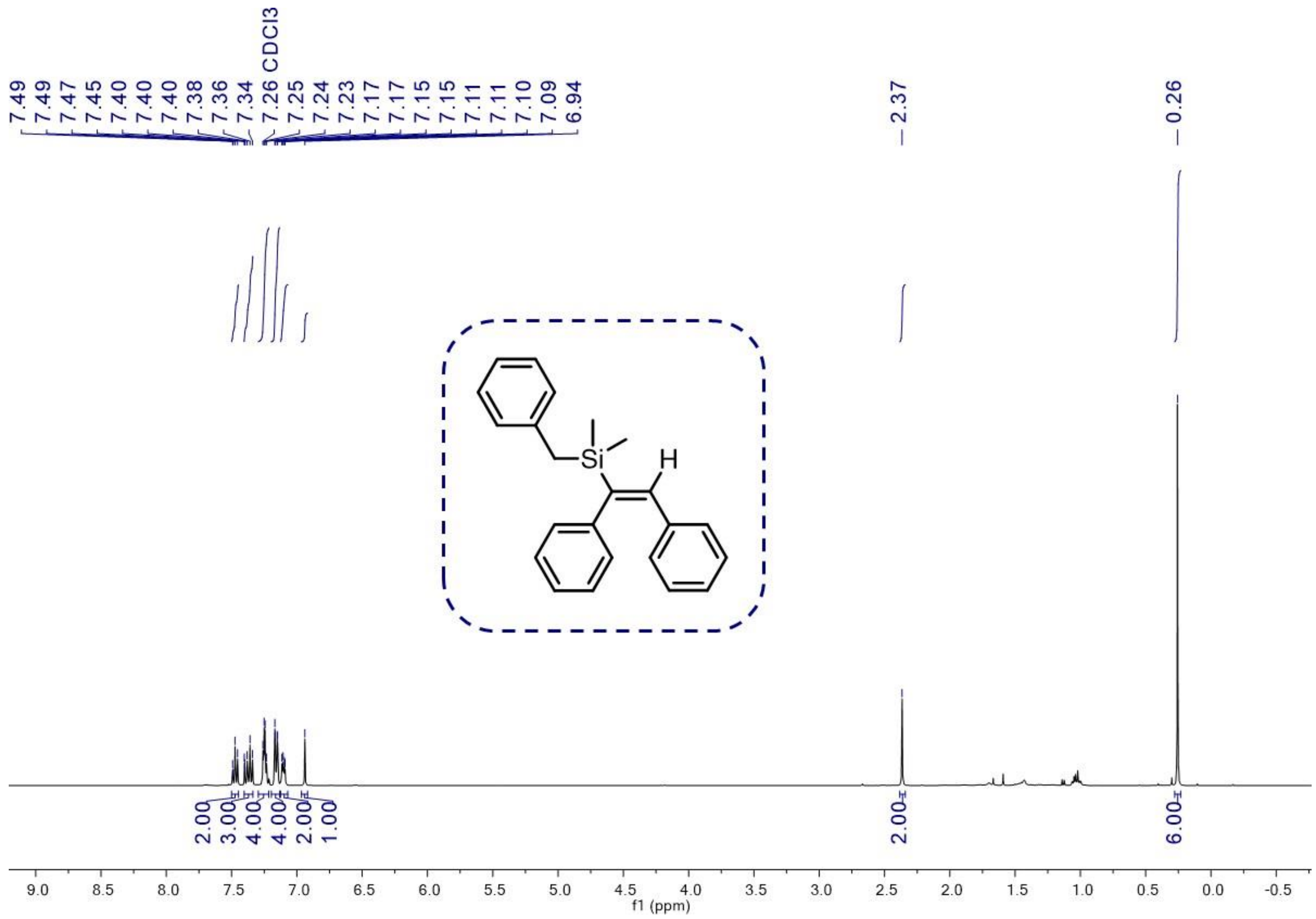

Figure S83. ^{1}H NMR spectrum of compound **4baa**.

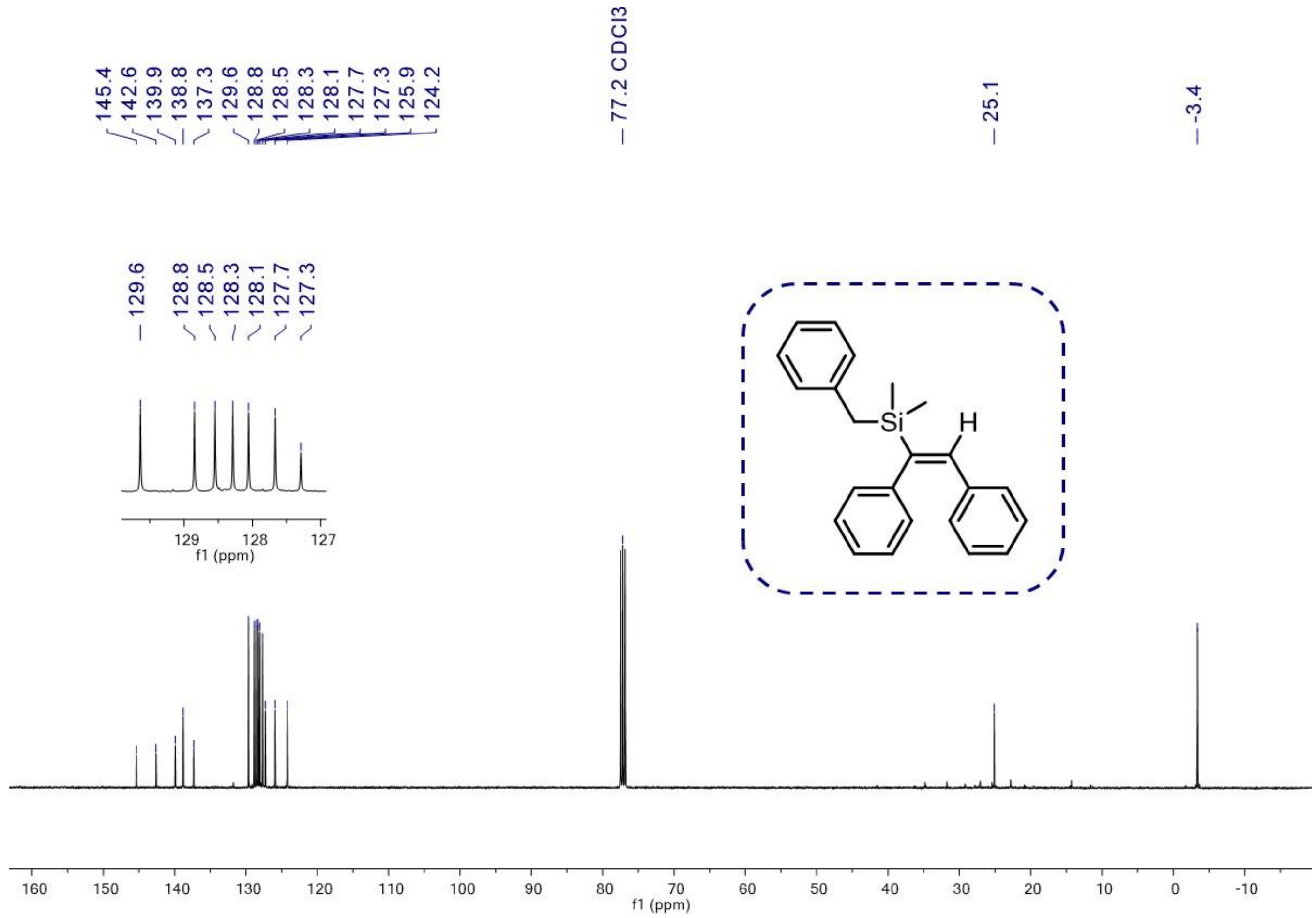

Figure S84. ^{13}C NMR spectrum of compound **4baa**.

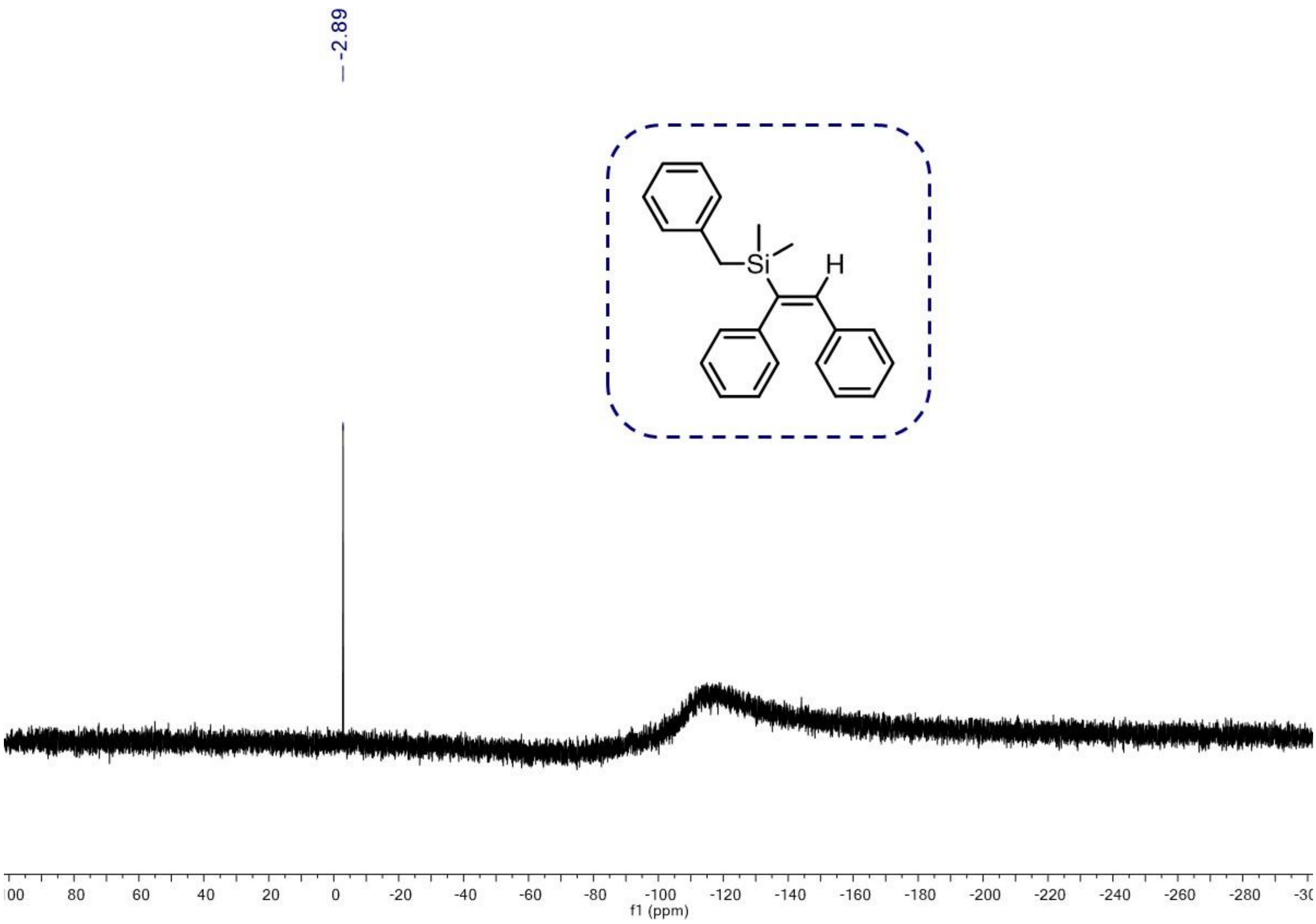

Figure S85. ^{29}Si NMR spectrum of compound **4baa**.

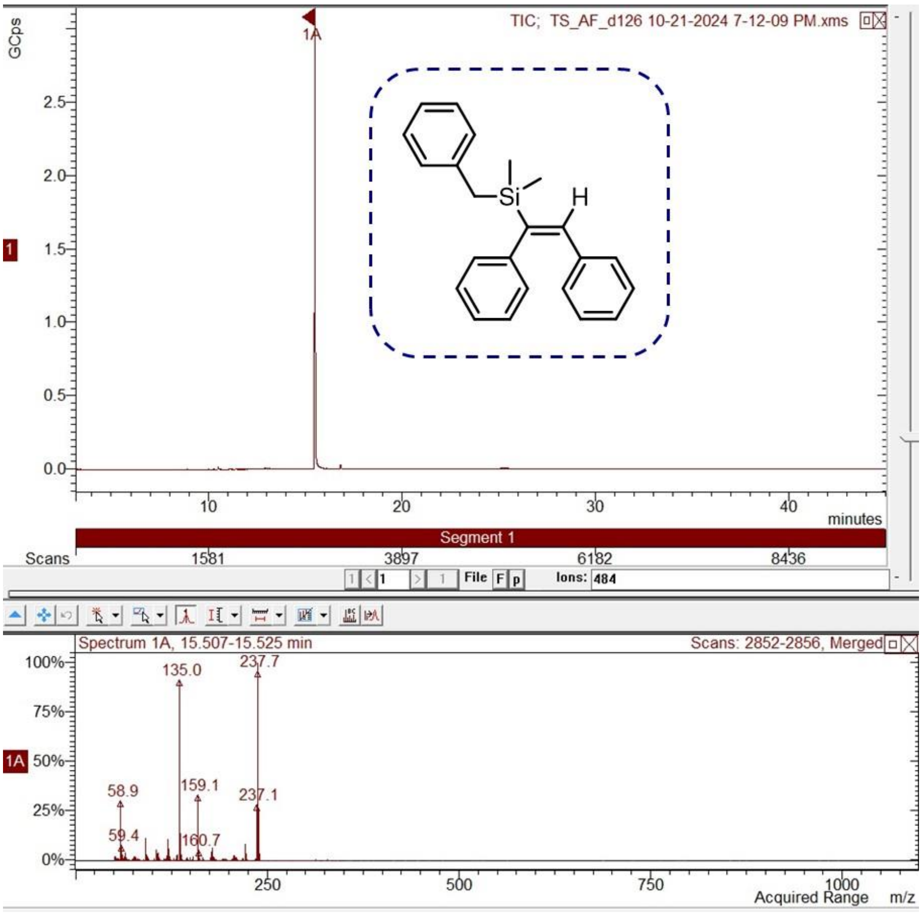

Figure S86. ^{29}Si NMR spectrum of compound **4baa**.

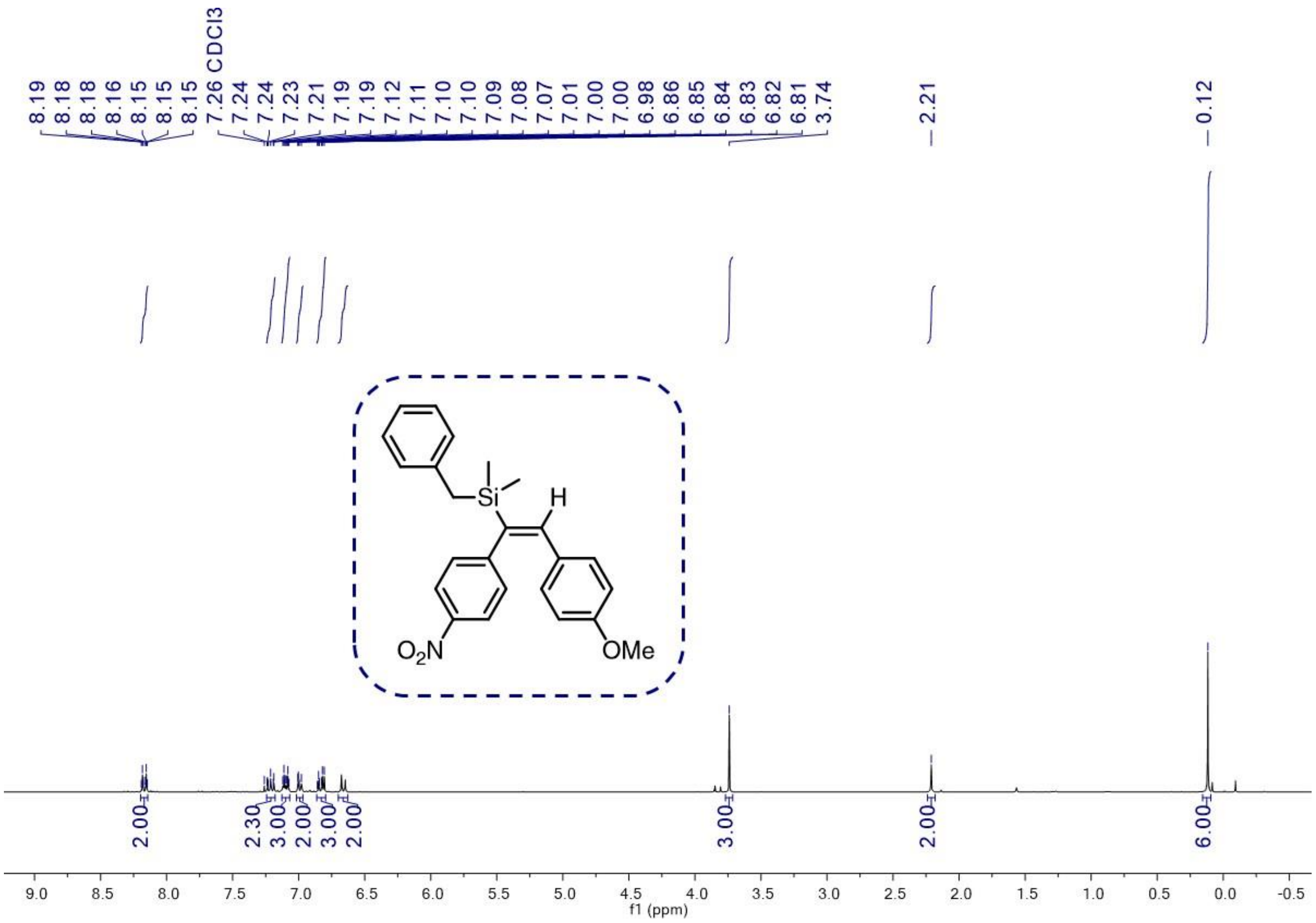

Figure S87. ^{1}H NMR spectrum of compound **4bef**.

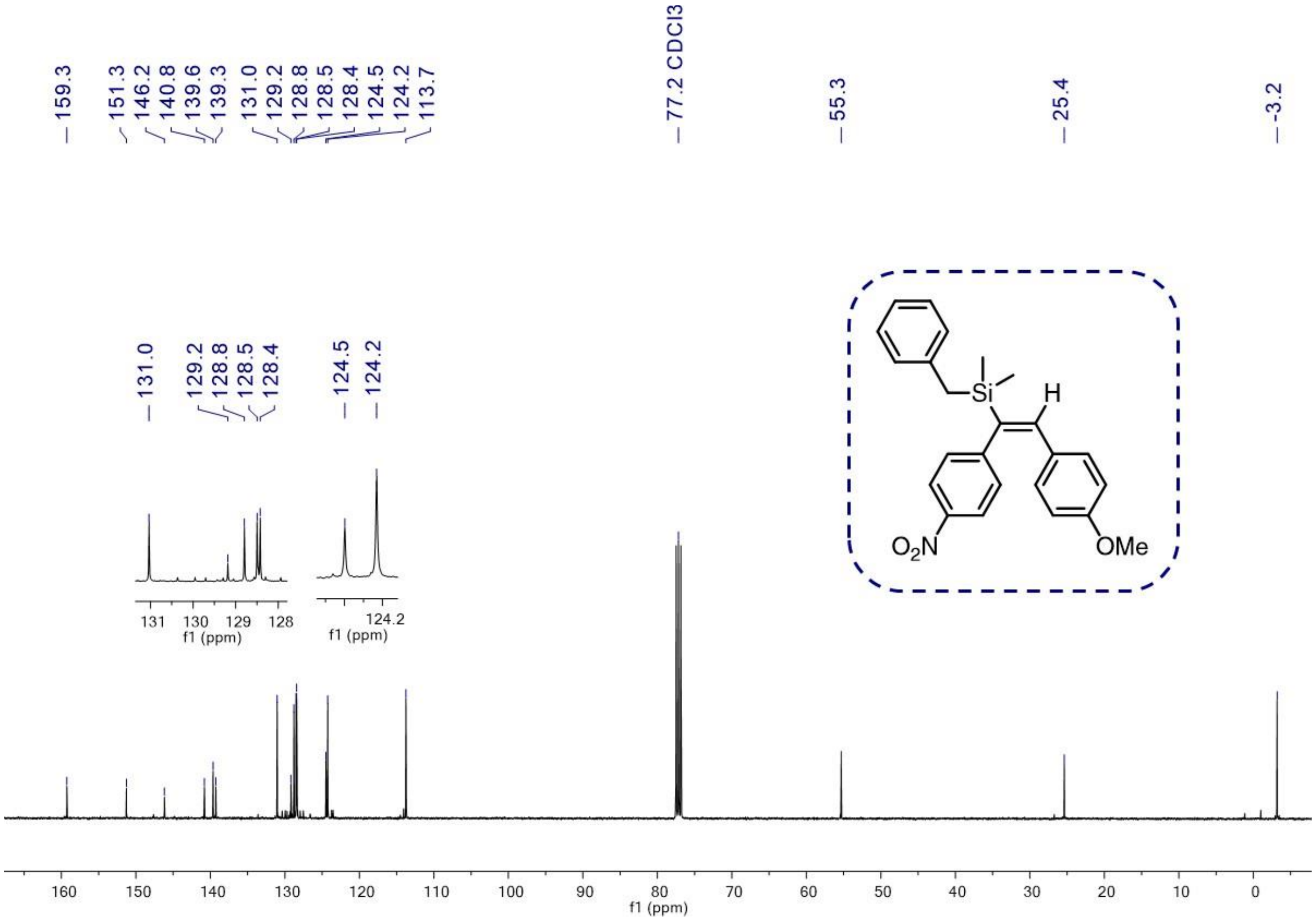

Figure S88. ^{13}C NMR spectrum of compound **4bef**.

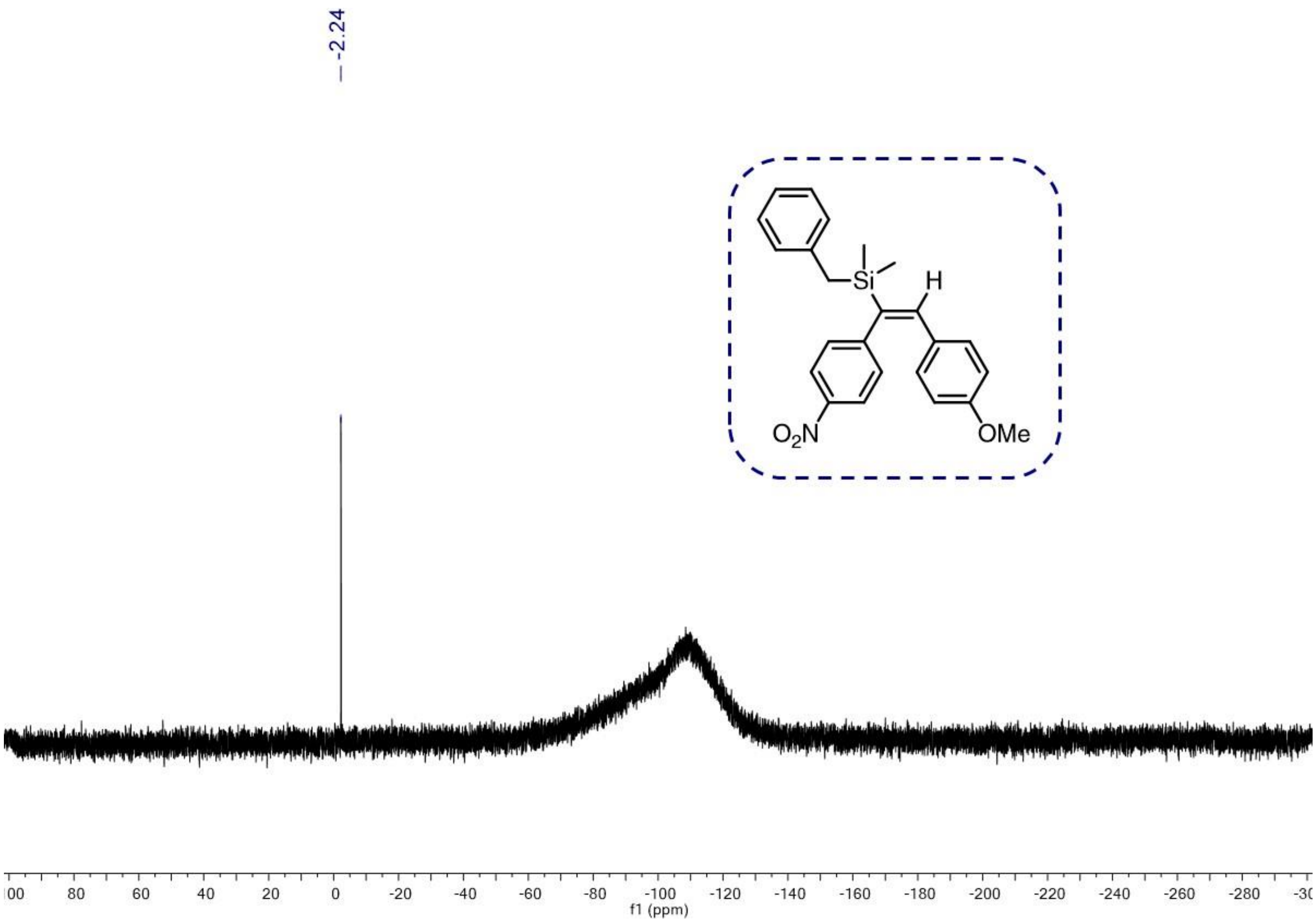

Figure S89. ^{29}Si NMR spectrum of compound **4bef**.

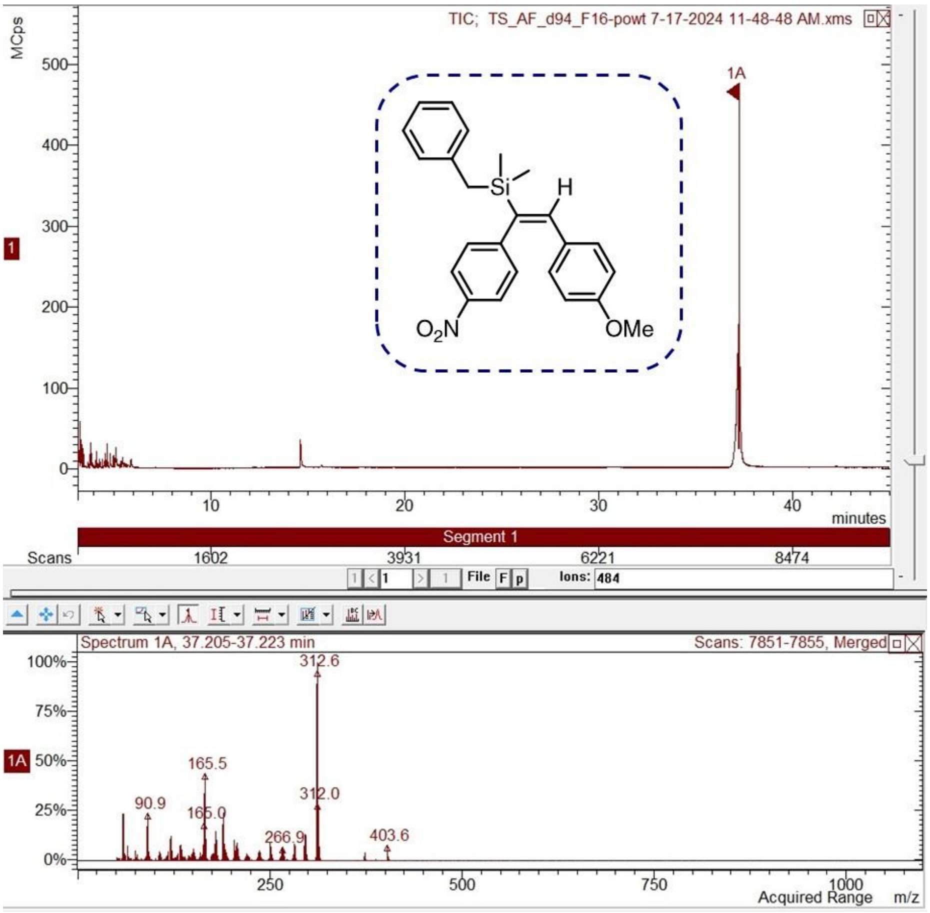

Figure S90. ^{29}Si NMR spectrum of compound **4bef**.

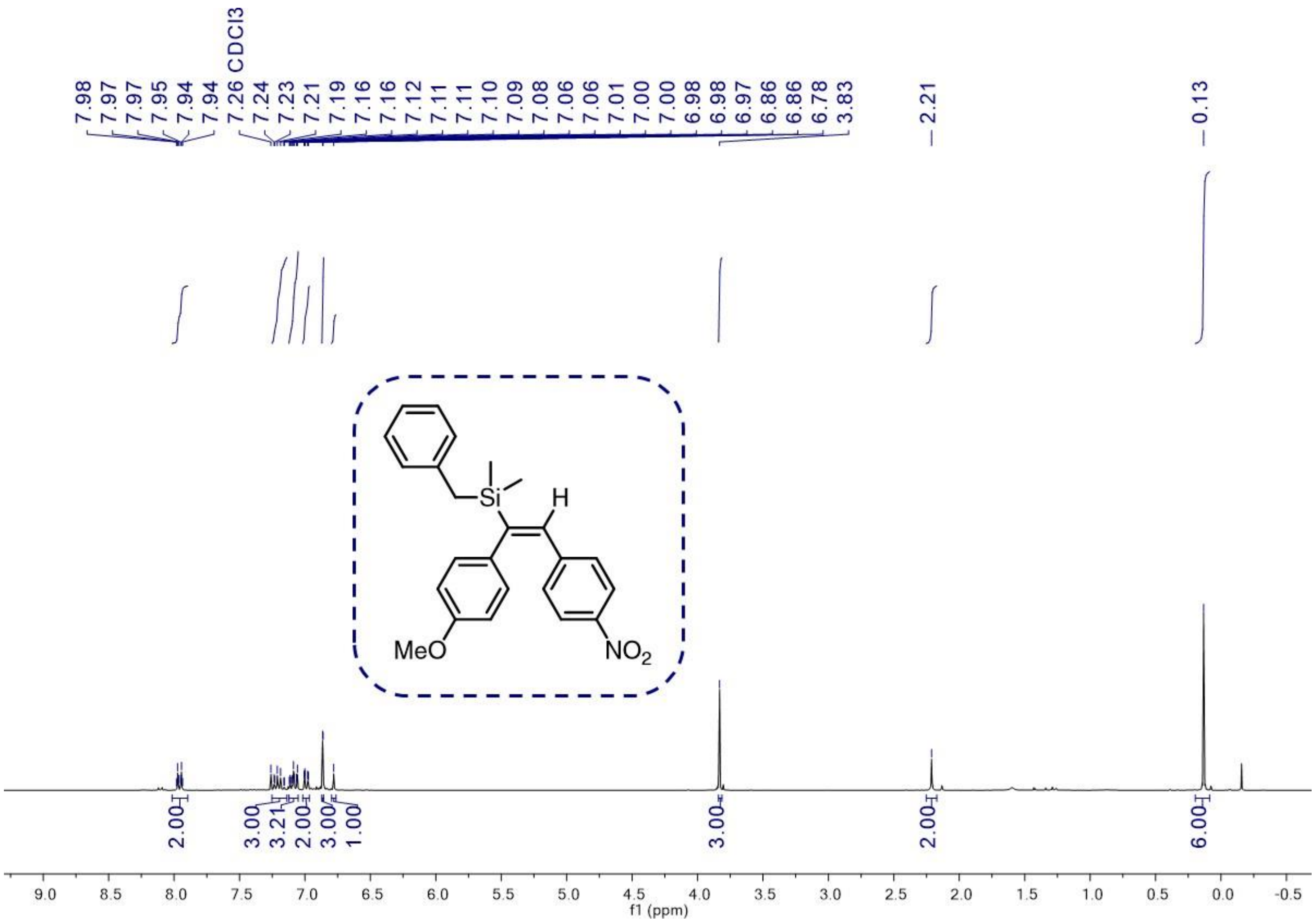

Figure S91. ^{1}H NMR spectrum of compound **4bfe**.

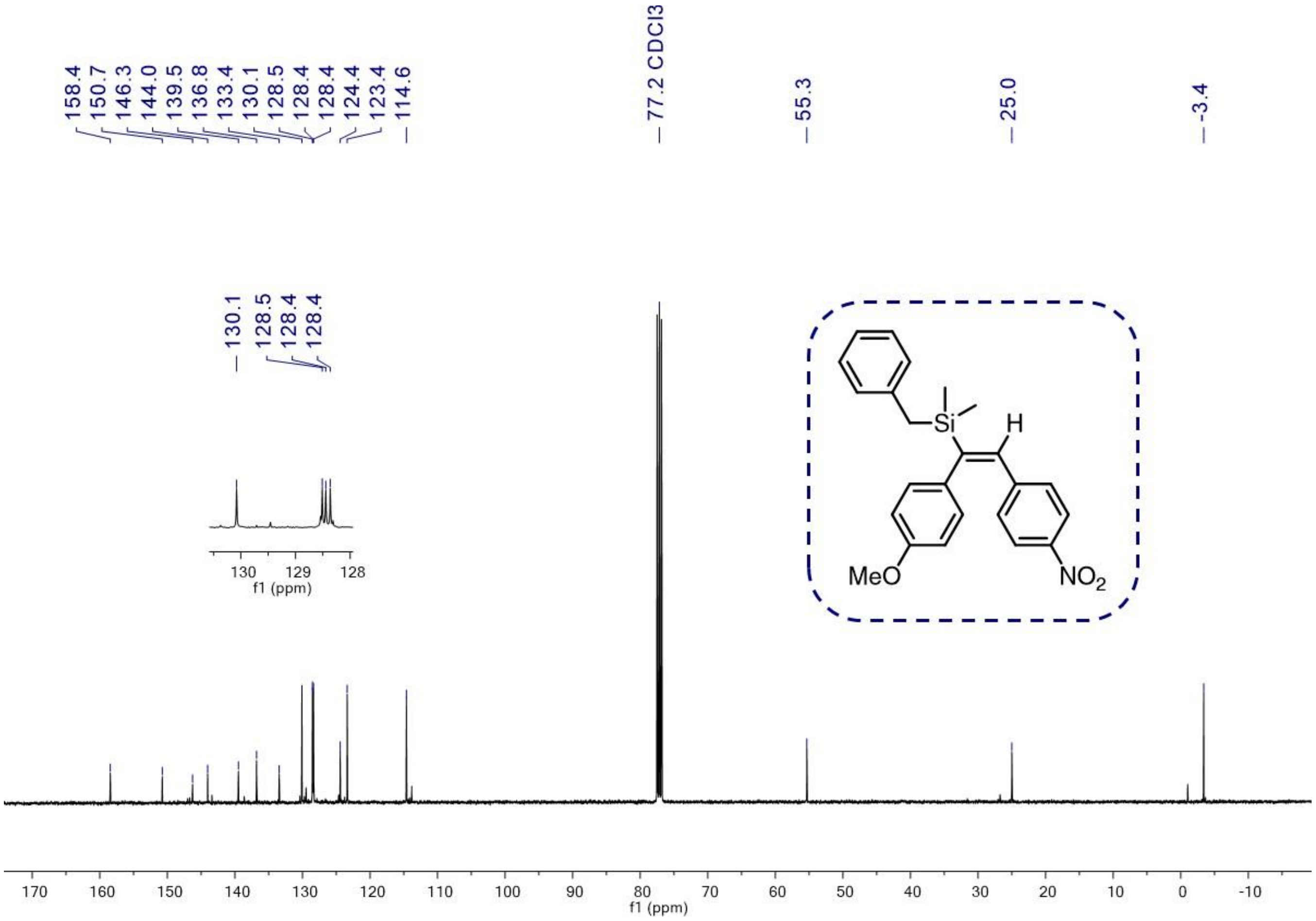

Figure S92. ^{13}C NMR spectrum of compound **4bfe**.

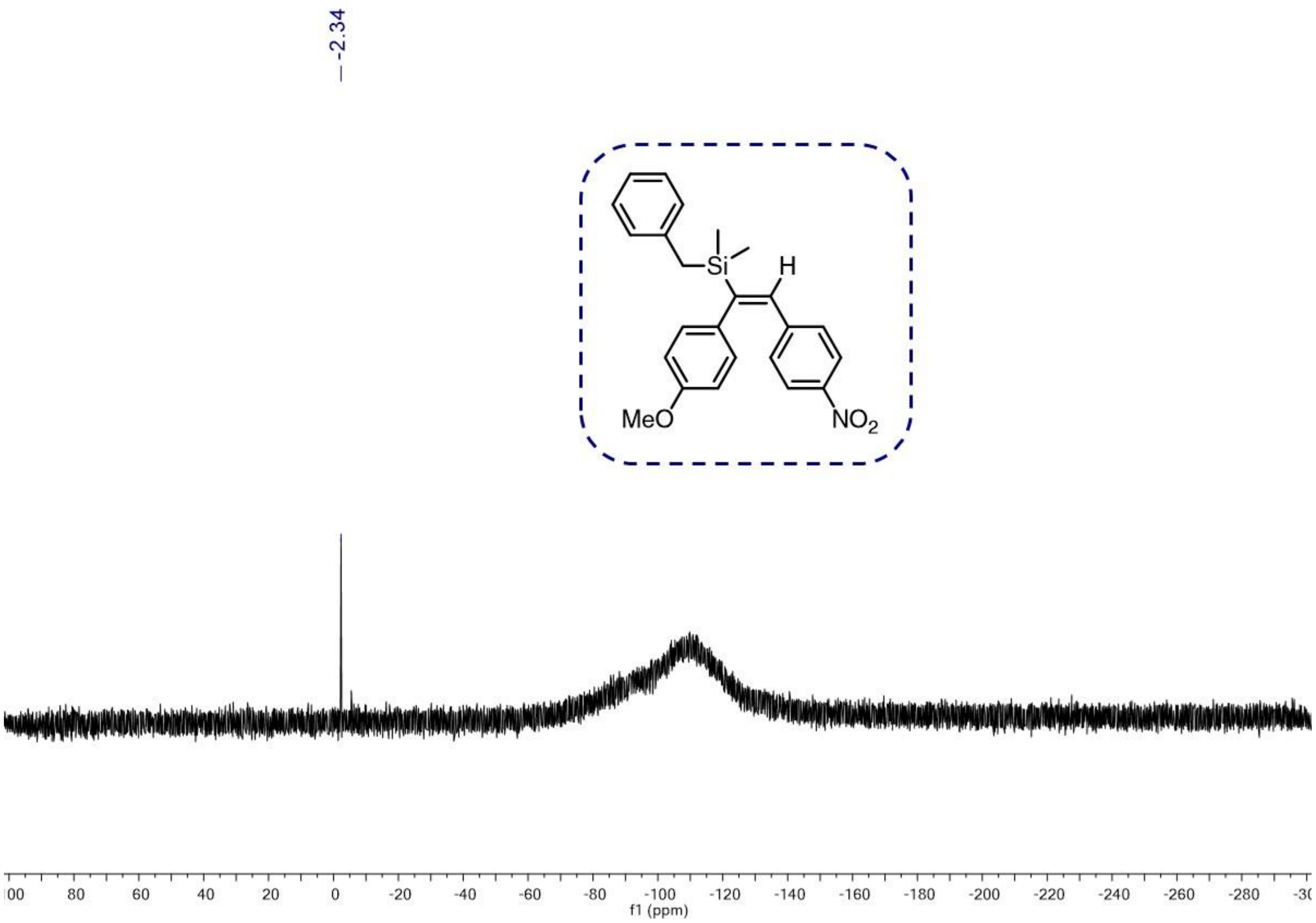

Figure S93. ^{29}Si NMR spectrum of compound **4bfe**.

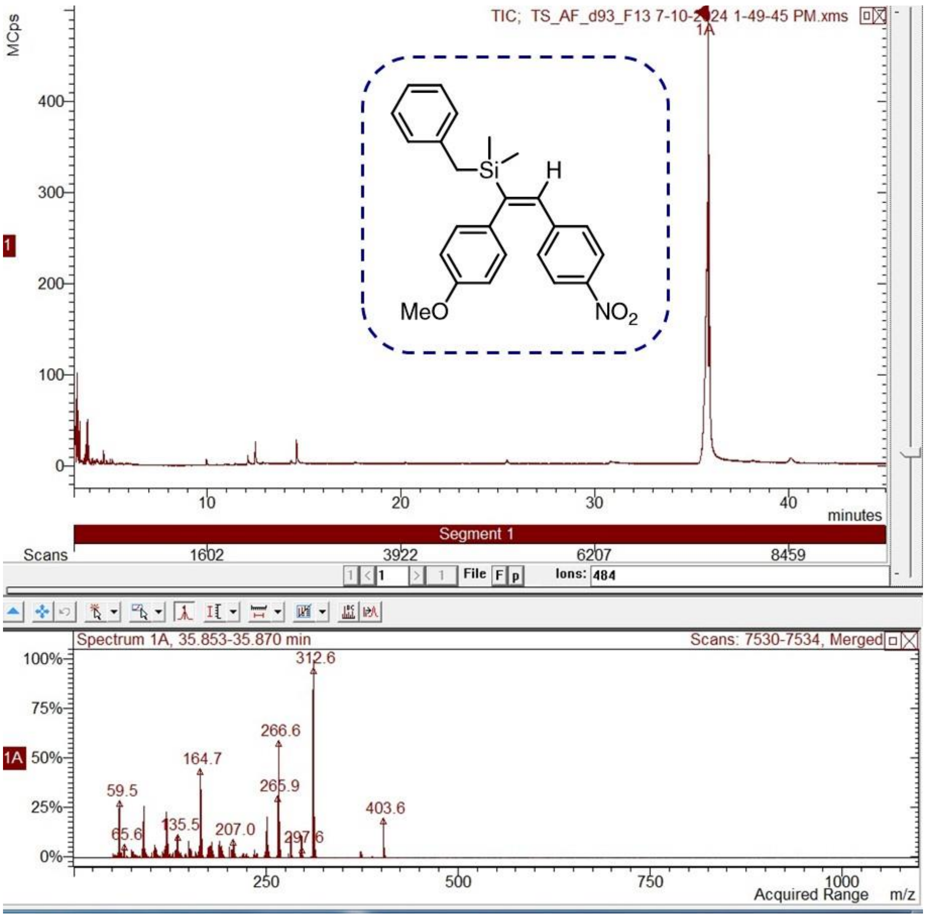

Figure S94. GC-MS image of compound **4bfe**.

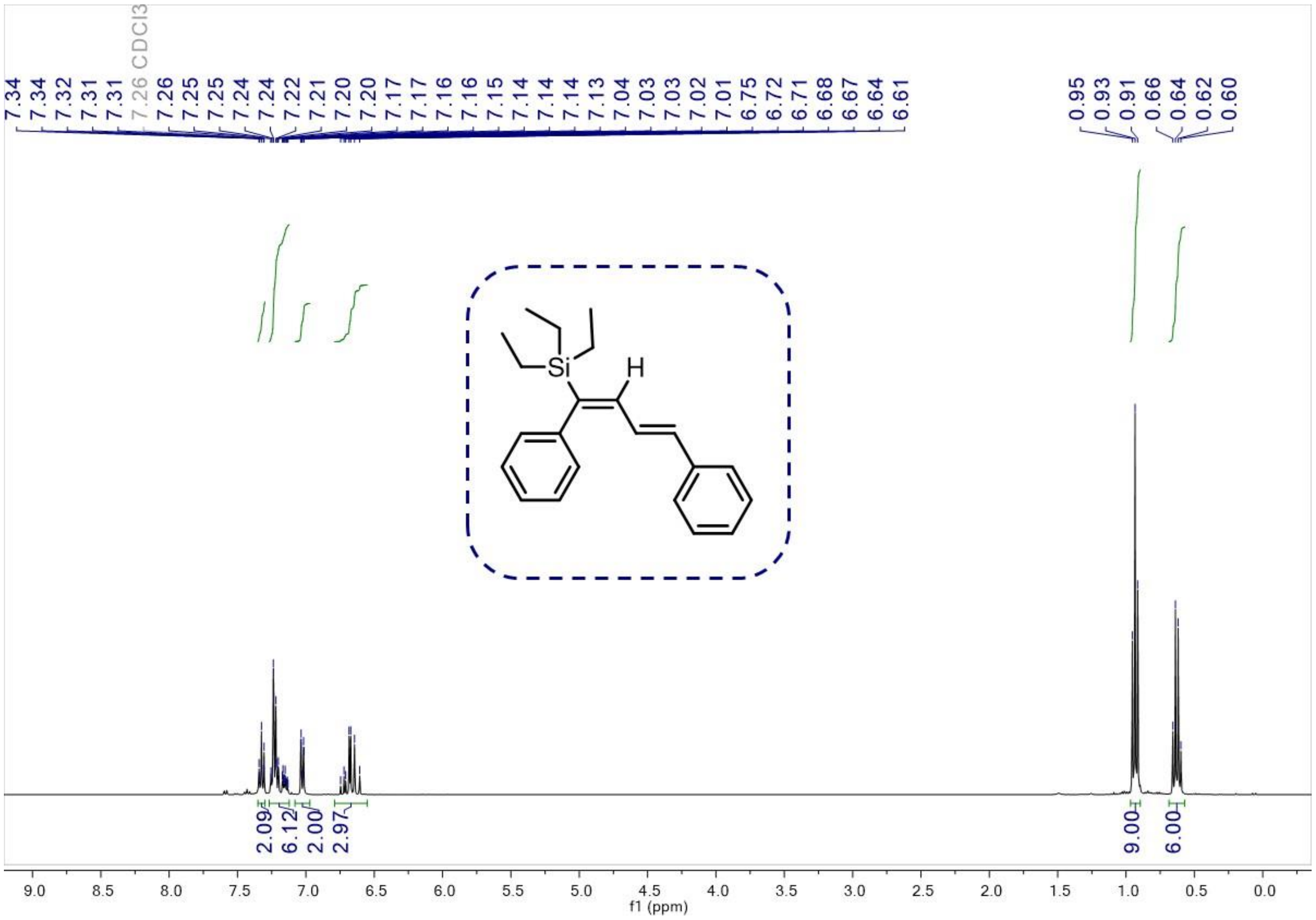

Figure S95. ^{1}H NMR spectrum of compound **4ala**.

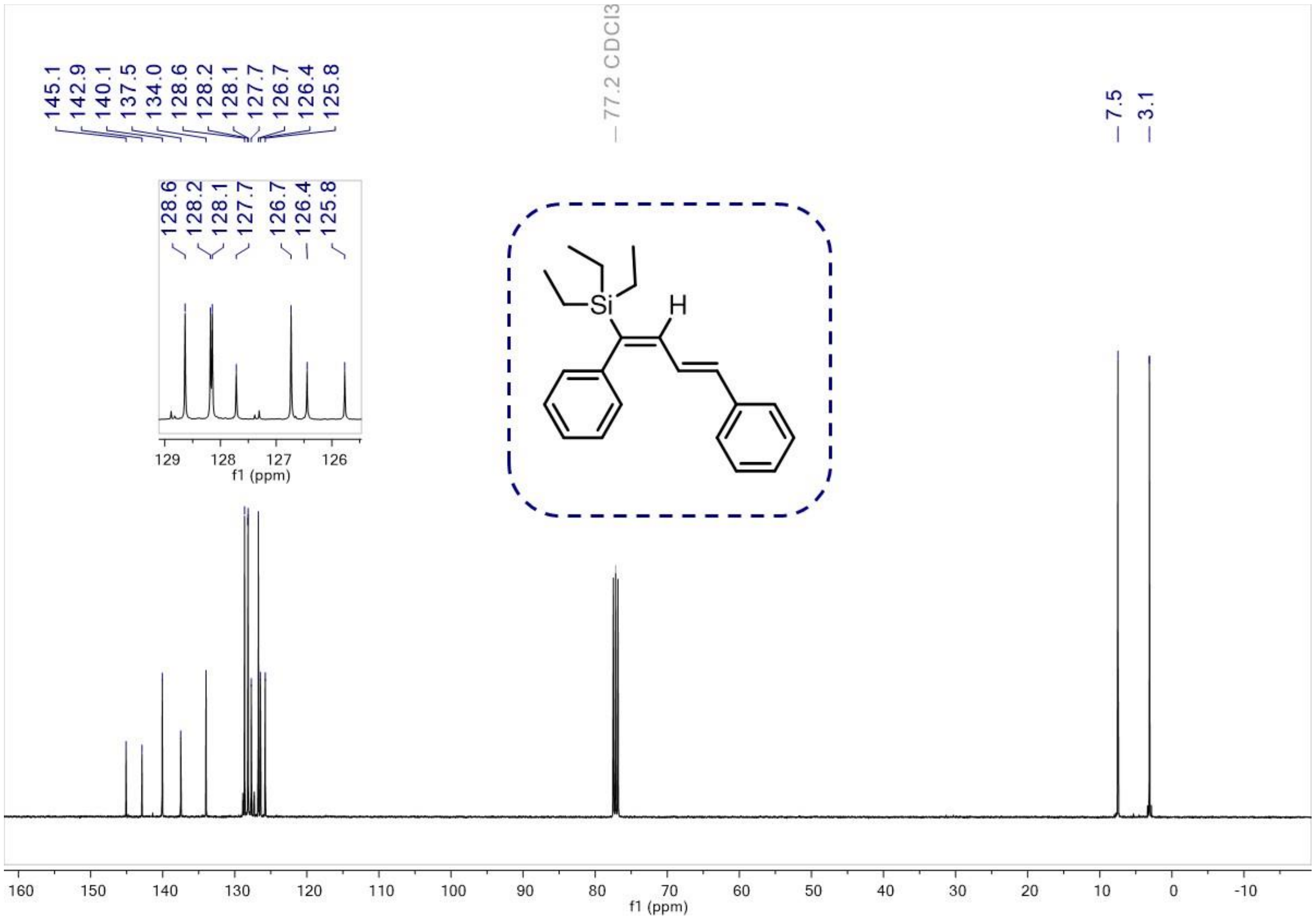

Figure S96. ^{13}C NMR spectrum of compound **4ala**.

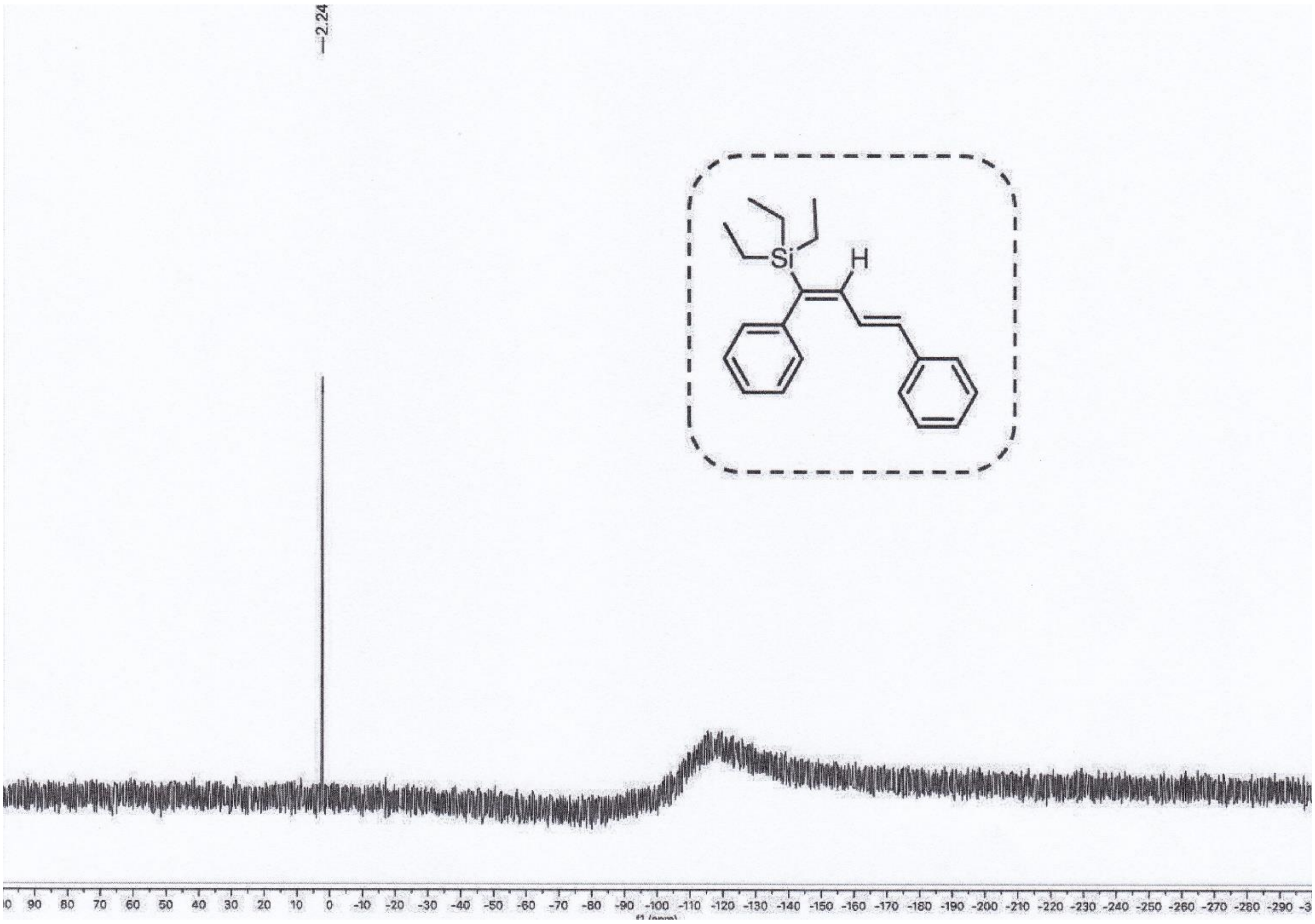

Figure S97. ^{29}Si NMR spectrum of compound **4ala**.

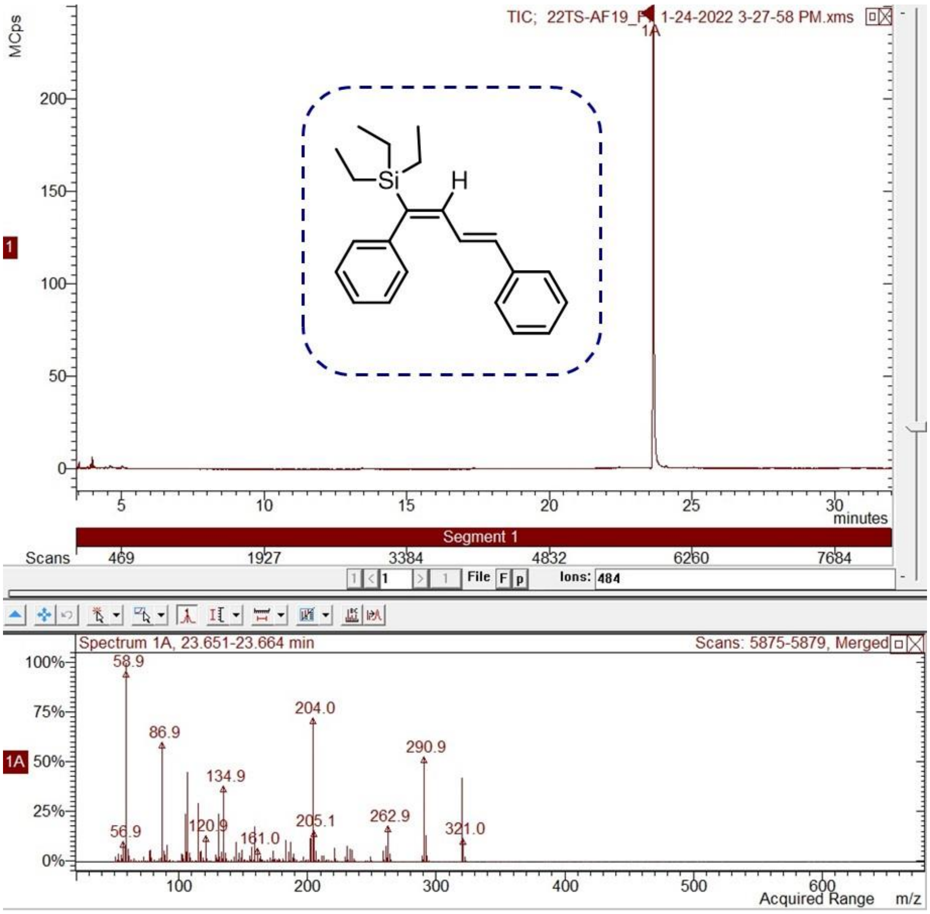

Figure S98. GC-MS image of compound **4ala**.

4. X-ray crystallography

A single crystals of **3bg_acid**, **3bl**, and **4baa** suitable for X-ray structural analysis were obtained by slow evaporation of solvent. The diffraction data were collected at 100 K with an D8 QUEST system equipped with a Microfocus source Incoatec ImS DII (Cu, λ = 1.54184 Å). The frames were integrated with the Bruker SAINT software package using a narrow-frame algorithm [61]. Data were corrected for absorption effects using the Multi-Scan method (SADABS) [62]. The structures were solved by direct methods with the program SHELXT 2018/2 [63] and refined by full-matrix least-squares method on F^2 with SHELXL 2018/3 [64]. The carbon-bound hydrogen atoms were refined as riding on their carriers and their displacement parameters were set equal to 1.5Ueq(C) for the methyl groups and 1.2Ueq(C) for the remaining H atoms.

A summary of the crystallographic data is given in Table S2. Molecular graphics were generated with Olex2 [65]. ORTEP representation of the molecular structures of the reported compounds is presented in Figures S80 - S83.

CCDC 2387733 (**3bg_acid**), 2385268 (**3bl**), and 2385269 (**4baa**) contains the supplementary crystallographic data for this paper. These data can be obtained free of charge via www.ccdc.cam.ac.uk/data_request/cif, or by emailing data_request@ccdc.cam.ac.uk, or by contacting The Cambridge Crystallographic Data Centre, 12 Union Road, Cambridge CB2 1EZ, UK; fax: +44 1223 336033.

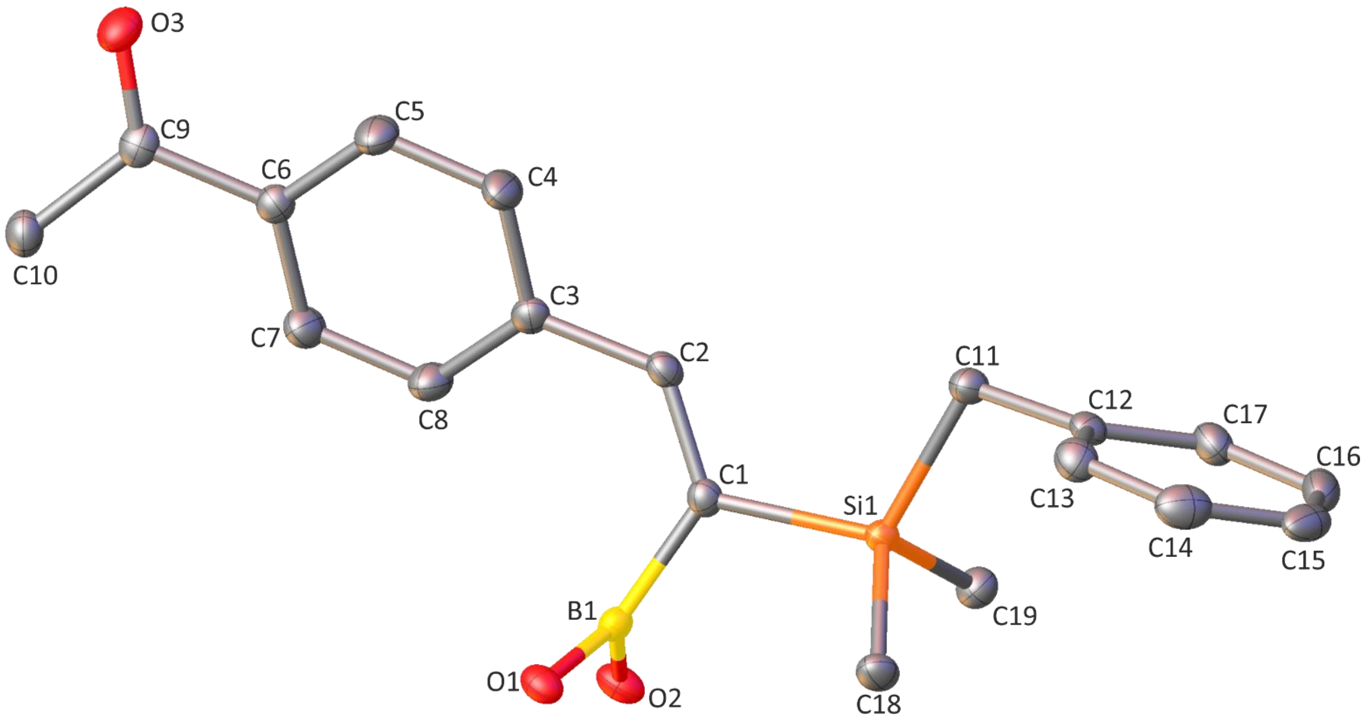

Figure S99. Molecular structure of compound **3bg_acid** and atoms numbering scheme. Hydrogen atoms are omitted for clarity. Displacement ellipsoids are shown at the 50% probability level.

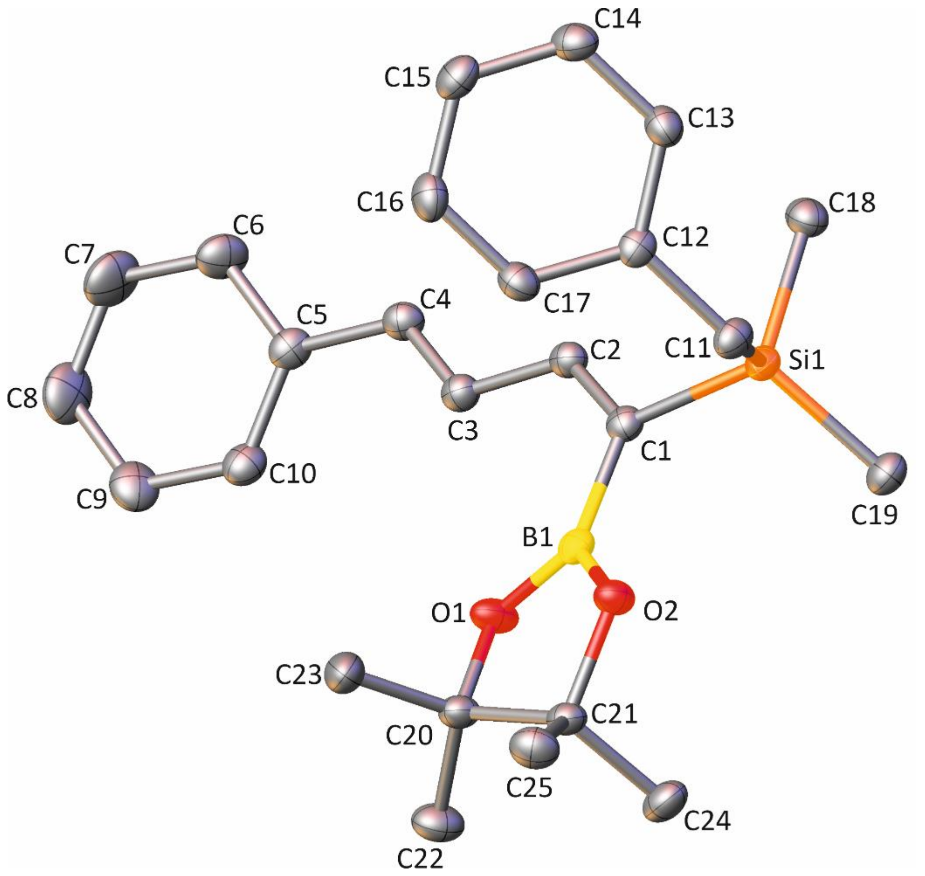

Figure S100. Molecular structure of compound **3bl** and atoms numbering scheme. Hydrogen atoms are omitted for clarity. Displacement ellipsoids are shown at the 50% probability level.

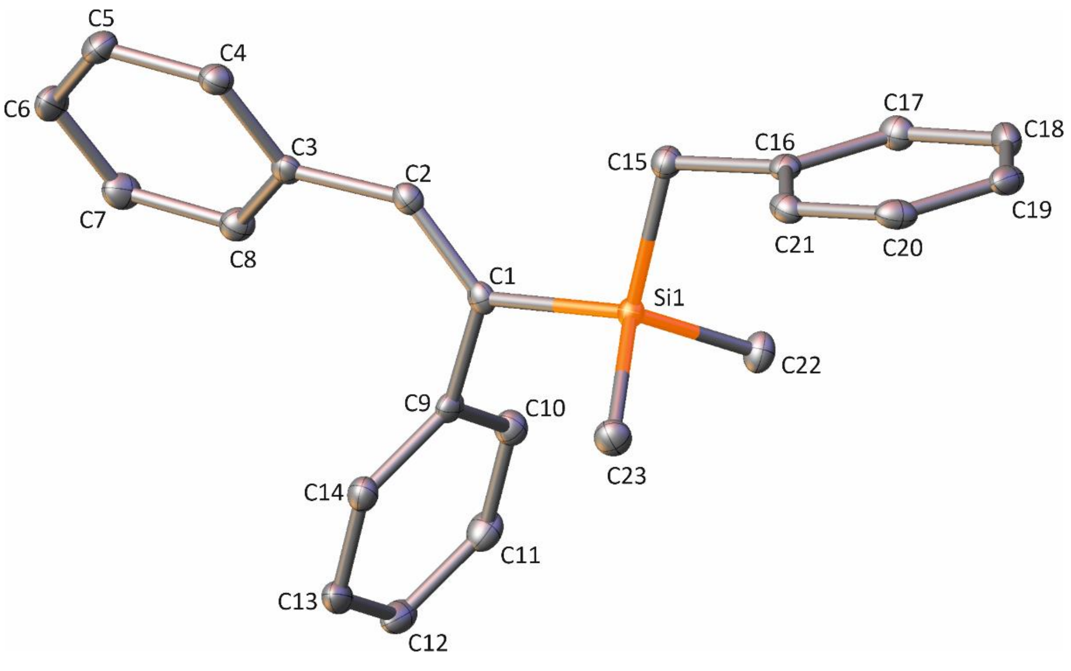

Figure S101. Molecular structure of compound **4baa** and atoms numbering scheme. Hydrogen atoms are omitted for clarity. Displacement ellipsoids are shown at the 50% probability level.

Table S2. Selected crystal data and structure refinement details for **3bg_acid, 3bl**, and **4baa**.

	**3bg_acid**	**3bl**	**4baa**
CCDC number	2387733	2385268	2385269
Chemical formula	$C_{19}H_{23}BO_3Si$	$C_{25}H_{33}BO_2Si$	$C_{23}H_{24}Si$
M_r	338.27	404.41	328.51
Crystal system, space group	Triclinic, $P\bar{1}$	Orthorhombic, *Pbca*	Monoclinic, $P2_1/c$
Temperature (K)		100	
a, *b*, *c* (Å)	11.8512 (4), 11.9282 (5), 13.3864 (4)	8.1671 (3), 13.7307 (5), 42.6059 (17)	10.5454 (2), 12.0637 (2), 14.5360 (3)
α, β, γ (°)	71.741 (2), 80.284 (2), 85.293 (2)		98.923 (1)
V (Å^3)	1770.43 (11)	4777.8 (3)	1826.84 (6)
Z	4	8	4
D_x (Mg m^{-3})	1.269	1.124	1.194
Radiation type		Cu *K*α	
μ (mm^{-1})	1.28	0.99	1.11
Crystal size (mm)	0.18 × 0.14 × 0.13	0.22×0.09×0.07	0.31×0.13×0.08
No. of measured, independent and observed [$I > 2\sigma(I)$] reflections	63782, 6709, 6081	88826, 4731, 4214	33922, 3602, 3320
R_{int}	0.029	0.052	0.032
$R[F^2 > 2\sigma(F^2)]$, $wR(F^2)$, S	0.034, 0.104, 1.07	0.034, 0.084, 1.05	0.030, 0.080, 1.04
No. of parameters	443	268	219
Δ〉max, Δ〉min (e Å^{-3})	0.46, -0.25	0.26, -0.26	0.38, -0.28

Table S3. Selected geometrical parameters (Å, °) in molecules of **3bg_acid, 3bl**, and **4baa**.

**3bg_acid**			
Si1A—C1A	1.8884 (12)	Si1B—C1B	1.8809 (12)
Si1A—C11A	1.9008 (13)	Si1B—C11B	1.9008 (13)
Si1A—C18A	1.8652 (13)	Si1B—C18B	1.8667 (13)
Si1A—C19A	1.8687 (13)	Si1B—C19B	1.8682 (13)
O1A—B1A	1.3576 (17)	O1B—B1B	1.3743 (16)
O2A—B1A	1.3750 (16)	O2B—B1B	1.3597(16)
O1A—B1A—O2A	117.97 (11)	O2B—B1B—O1B	118.19 (11)
Si1A—C11A—C12A—C17A	-85.33 (13)	Si1B—C11B—C12B—C17B	88.01 (13)
C1A—C2A—C3A—C4A	-8.2 (2)	C1B—C2B—C3B—C8B	-22.6 (2)
C2A—C1A—B1A—O2A	92.80 (16)	C2B—C1B—B1B—O1B	-83.20 (16)
**3bl**			
Si1—C1	1.8801 (13)	Si1—C18	1.8689 (14)
Si1—C11	1.8883 (14)	Si1—C19	1.8746 (13)
O2—B1—O1	112.10 (11)		
C1—Si1—C11—C12	-62.62 (11)	C3—C4—C5—C10	-16.5 (2)
C2—C1—B1—O1	16.9 (2)		
**4baa**			
Si1—C1	1.8901 (11)	Si1—C22	1.8670 (12)
Si1—C15	1.8944 (12)	Si1—C23	1.8702 (12)
Si1—C1—C2—C3	-176.91 (9)	C1—C2—C3—C8	-20.26 (19)
Si1—C1—C9—C10	-75.72 (12)	C15—Si1—C1—C9	174.57 (8)
Si1—C15—C16—C21	-88.26 (11)		

5. References

40. Stefanowska, K.; Sokolnicki, T.; Walkowiak, J.; Czapik, A.; Franczyk, A., Directed cis-Hydrosilylation of Borylalkynes to Borylsilylalkenes. *Chem. Commun.* **2022,** 58, (86), 12046-12049.

46. Sha, W.; Changpeng, C.; Xiaoming, Z., Bipyridine Ligand-Promoted cis-Selective Hydroboration of Alkynes with Chromium Catalysis. *Chin. J. Org. Chem.* **2023,** 43, (7), 2447.

59. Alonso, F.; Buitrago, R.; Moglie, Y.; Ruiz-Martínez, J.; Sepúlveda-Escribano, A.; Yus, M., Hydrosilylation of alkynes catalysed by platinum on titania. *J. Organomet. Chem.* **2011,** 696, (1), 368-372.

60. Kim, S.-B.; Lee, C.-H.; Jun, C.-H., Styrylsilane coupling reagents for immobilization of organic functional groups on silica and glass surfaces. *Chem. Commun.* **2018,** 54, (71), 9961-9964.

61. SAINT V8.40B, Bruker AXS LLC, 2019

62. L. Krause, R. Herbst-Irmer, G. M. Sheldrick, D. Stalke, Comparison of silver and molybdenum microfocus X-ray sources for single-crystal structure determination *J. Appl. Cryst*. **2015**, *48*, 3-10.

63. G. M. Sheldrick, SHELXT – Integrated space-group and crystal-structure determination. *Acta Crystallogr*. **2015**, *A71*, 3-8.

64. G. M. Sheldrick, Crystal structure refinement with SHELXL. *Acta Crystallogr*. **2015**, *C71*, 3-8.

65. O. V. Dolomanov, L. J. Bourhis, R. J. Gildea, J. A. K. Howard, H. Puschmann, OLEX2: a complete structure solution, refinement and analysis program. *J. Appl. Cryst* **2009**, *42*, 339—341.
